# Regulatory performance dataset constructed from U.S. soil jurisdictions based on the top 100 concerned pollutants

**DOI:** 10.1016/j.dib.2018.09.049

**Published:** 2018-09-27

**Authors:** Wenbiao Li, Zijian Li, Aaron Jennings

**Affiliations:** a Department of Civil Engineering, Case Western Reserve University, Cleveland, OH 44106, USA; b Parsons Corporation, Chicago, IL 60606, USA

## Abstract

The regulatory performance dataset based on the standard-value-based comparison tool was summarized in this Data in Brief. The dataset includes the identified top 100 concerned soil pollutants, the computed C_2_–C_5_ regulatory performance scores for each state soil jurisdiction, and the reference sources of the soil regulatory guidance values (RGVs). A total of 20 elements, seven cyanides, five halogenated methanes, seven chloroethanes and choroethenes, 12 benzenes, eight phenols, eight carcinogenic PAHs, eight noncarcinogenic PAHs, nine historically used pesticides, 12 currently used pesticides, and nine miscellaneous pollutants were selected as the top 100 concerned pollutants. Four comparison scores simulated from state soil regulations can be directly applied and compared with the U.S. Environmental Protection Agency to quantify the regulatory performance.

**Specifications table**

**Table:** 

Subject area	*Environmental Science*
More specific subject area	*Environmental Policy*
Type of data	*Table and text file*
How data was acquired	*Computational Simulation*
Data format	*Analyzed*
Experimental factors	*Not applicable*
Experimental features	*The standard-value-based comparison tool*
Data source location	*Not applicable*
Data accessibility	*The data are available with this article*
Related research article	*Li, W., Li, Z. and Jennings, A., 2018. A standard-value-based comparison tool to analyze US soil regulations for the top 100 concerned pollutants. The Science of the total environment, 647, pp. 663–675.*

**Value of the data**

• Top 100 concerned soil pollutants were selected based on the environmental occurrence, abundance, and human health effects.
• Regulatory performance scores C_2_–C_5_ were computed and summarized for each state soil regulation.
• Comparison of state regulation scores with the U.S. EPA could help measure and quantify regulatory risks.

## 1 Data

The major chemical classes identified in this study, including 20 elements, seven cyanides, five halogenated methanes, seven chloroethanes and choroethenes, 12 benzenes, eight phenols, eight carcinogenic PAHs, eight noncarcinogenic PAHs, nine historically used pesticides, 12 currently used pesticides, and nine miscellaneous pollutants, were summarized in Table S1 (One hundred contaminants and their CAS number), which were selected based on the environmental occurance, health concerns, and regulatory frequency [1], [2], [3], [4], [5], [6], [7], [8], [9], [10], [11], [12], [13], [14], [15], [16], [17], [18], [19], [20]. The computed regulatory scores based on the top 100 concerned soil pollutants for each U.S. state soil jurisdiction were listed in the Section 3 (C_2_–C_5_ score values and top-concerned pollutant soil RGVs for individual state jurisdictions), which can be directly applied and compared to quantify the performance of state soil regulations.

## 2 Experimental design, materials and methods

The top 100 concerned soil pollutants (Table S1) were selected based upon their use, toxicity, environmental occurrence, abundance, and human effects. All of them can cause cancer or non-cancer adverse health effect via chronic soil exposure route. Therefore, it’s necessary to evaluate whether state regulatory jurisdictions have provided sufficient and safe soil RGVs to protect public health or not. The regulatory performance scores C_2_–C_5_ were derived based on the developed comparison tool, which integrating the median and geometric mean of the worldwide soil RGVs. These scores can be directly used to quantify the performance of each state’s soil regulation, or compared with the U.S. EPA score associated with human health risk uncertainty bounds to assess the regulatory risks.

## 3 C_2_–C_5_ score values and top-concerned pollutant soil RGVs for individual state jurisdictions

### 3.1 Alabama

Alabama has one set of RGVs from the Alabama Department of Environmental Management [21]. Table S2 lists 92 standards for the pollutants considered. The values of C_2_ and C_3_ are 0.054 and 0.15, while the C_4_ and C_5_ values are −36 and −18. The average order of magnitude does not have much difference compared with average median and geometric mean, although about 70% are lower than average median, and 60% data are lower than average geometric mean. Overall, RGVs provides values are generally lower than the test statistics within half order of magnitude.

### 3.2 Alaska

Alaska has 3 sets of RGVs provided by the Alaska Department of Environmental Conservation [22]. First is “Arctic zone” meaning areas north of latitude 68° north. Area south of that latitude are also considered an arctic zone on a site-specific basis based on a demonstration that the site is underlain by continuous permafrost. Second is “under 40-in. zone” indicating that a site receives mean annual precipitation of less than 40 inches each year. Third is “over 40-in. zone” indicating that the mean annual precipitation is 40 or more inches each year.

Table S3 of arctic zone lists 82 standards for the pollutants considered. The C_2_ and C_3_ values are 1.48 and 1.52 RGVs are more than one order of magnitude higher, and about 95% of the RGVs are larger than the average.

Table S3b, under 40-in. zone, also has 82 RGVs, and about 88% are more than one order of magnitude higher than the average, but slightly lower than RGVs from arctic zone.

Table S3C, where over 40-in., has the same number of RGVs as others, and about 91% are more than one order of magnitude higher than the average, but even lower than RGVs from under 40-in. zone.

Overall, Alaska’s RGVs are more than one order of magnitude above the central tendency, but differ a bit based on site hydrology.

### 3.3 Arizona

Arizona has three sets of RGVs listed in the Arizona Administrative Code [23]. The first is based on a 1E-06 cancer risk (see Appendix A) [23]. The second is based on a 1E-05 cancer risk. The third is a list of residential soil remediation levels from Appendix B.

Table S4a presents an analysis of the RGV set based on a 1E-06 cancer risk. The values of C_2_ and C_3_ are 0.47 and 0.63 and are about half order of magnitude higher than the average. The values of C_4_ and C_5_ are 10 and 22 indicating that a substantial number of RGVs higher than the test statistics.

Table S4b presents an analysis of the RGVs based on a 1E-05 cancer risk. The C_2_ and C_3_ values are 0.85 and 1.01 that are about one order of magnitude higher than the average. The C_4_ and C_5_ values are 65 and 81 that about 90% of the data are above the test statistic.

Table S4c presents an analysis of the RGVs from Appendix B. The C_2_ and C_3_ valuess are 0.84 and 0.99 that are about one order of magnitude higher than the average. The C_4_ and C_5_ values are 69 and 71 indicating that about 70% of the RGVs are above the test statistic.

Thus, Arizona is regulating most pollutants with RGVs that are higher than average by about 0.5–1 order of magnitude.

### 3.4 California

California has three sets of RGVs. Two are from California Human Health Screening Levels [24] that contains three tables:

Table S1. Soil Screening Numbers for Nonvolatile Chemicals Based on Total Exposure to Contaminated Soil: Inhalation, Ingestion and Dermal Absorption.
Table S2. Soil-Gas-Screening Numbers for volatile Chemicals below buildings Constructed With Engineered Fill Below Sub-slab Gravel.
Table S3. Soil-Gas-Screening Numbers for Volatile Chemicals below Buildings Constructed Without Engineered Fill Below Sub-slab Gravel.

Another set is from the California Human Health Risk Assessment [25]. Table S5a presents an analysis of values from the California Tables S1 and S2 from California Human Health Screening Levels This RGV set has 41 values for pollutants considered here. Among the missing pollutants are benzenes, phenols, and current use pesticides. The C_2_ and C_3_ values are 0.024 and 0.073, which are close to the average median and geometric mean. The C_4_ and C_5_ values are −9 and −5, which indicates that more values are lower than the test statistics.

Table S5b presents an analysis of the RGVs from the California Tables S1 and S3 from CaHHSL [24]. This RGV set also has 41 pollutants with the same chemicals missing. The C_2_ and C_3_ values of −0.073 and −0.049 are only slightly lower than the test statistics. The C_4_ and C_5_ values are −9 and −5 indicating that the RGV set has more values lower than the average.

Table S5c presents an analysis of the RGVs from the California Human Health Risk Assessment [25]. This RGV set only has 20 values for the pollutants considered here including metals, cyanides, halogenated methanes, and chloroethanes and choroethenes. The C_2_ and C_3_ values are both −0.05, and the C_4_ and C_5_ values are both −6. Although RGVs are close to the average, the number of data is too low to determine the regulating performance.

### 3.5 Colorado

Colorado has one set of RGVs from the Colorado Department of Public Health and Environment [26]. Table S6 lists the 78 RGVs for pollutants considered. The list is missing some current use pesticides and miscellaneous pollutants. The C_2_ and C_3_ scores of 0.46 and 0.55 are about half order of magnitude higher than the test statistics. The C_4_ and C_5_ scores are 2 and 10, indicating that a few more values are higher than the test statistics rather lower. Therefore, Colorado’s RGVs are slightly higher than the average by about half an order of magnitude.

### 3.6 Connecticut

Connecticut has one set of RGVs from the Connecticut Department of Energy and Environmental Protection [27]. Table S7 presents the 60 RGVs considered. The set is missing of some metals, phenols, and pesticides. The C_2_ and C_3_ scores are 0.63 and 0.73, which are no more than one order of magnitude higher than the test statistics. The C_4_ and C_5_ values are 26 and 32, indicating that are about 70% higher than the test statistics. Thus, Connecticut’s RGVs are higher than the central tendencies by about one order of magnitude.

### 3.7 Delaware

Delaware has set of RGVs from the Delaware Department of Natural Resources and Environmental Control [28]. Table S8 presents an analysis of Delaware’s 96 RGVs. The C_2_ and C_3_ values are 0.021 and 0.14, which are close to the test statistics. The C_4_ and C_5_ values are −32 and −20 indicating more values are lower than the test statistics. Overall Delaware’s RGVs are lower than, the test statistics but by less than one order of magnitude.

### 3.8 Florida

Florida has one set of RGVs from the Florida Department of Environmental Protection [29]. Table S9 lists Florida’s 91 RGVs for the pollutants considered here. The C_2_ and C_3_ values are 0.63 and 0.76 that are no more than one order of magnitude higher than the test statistics. The C_4_ and C_5_ values are both 37, indicating that 70% of the RGVs are higher than the test statistics. Overall, Florida’s RGVs are higher than the test statistics by about one order of magnitude.

### 3.9 Georgia

Georgia has one set of RGVs from the Georgia Department of Natural Resources [30]. Table S10 lists 89 RGVs for the pollutants considered. The scores of C_2_ and C_3_ are −0.31 and −0.15 that are slightly lower than the average median and geometric mean. The scores of C_4_ and C_5_ are −15 and −19 that are about 60% of the data are lower than the test statistics. Overall, Georgia’s RGVs are lower than the test statistics within half order of the magnitude.

### 3.10 Hawaii

Hawaii has one set of RGVs from the Hawaii Dept. of Health [31]. Table S11 lists 88 RGVs for the pollutants considered. The C_2_ and C_3_ values are 0.35 and 0.51 that standards are higher than the test statistics. The C_4_ and C_5_ values are −2 and 20, where standards are close to the average median, and about 60% of the values are higher than the average geometric mean. Overall, Hawaii’s RGVs are close to the average median, while slightly higher than the average geometric mean.

### 3.11 Idaho

Idaho has one set of RGVs from the Idaho Department of Environmental Quality [32]. Table S12 presents 85 RGVs for the pollutants considered. The scores of C_2_ and C_3_ are −1.19 and −1.15, which standards are more than one order of magnitude below the central tendencies. The scores of C_4_ and C_5_ are both −67, which about 90% of the values are lower than the test statistics. Overall, Idaho’s RGVs are lower than the test statistics by more than one order of magnitude.

### 3.12 Illinois

Illinois has one set of RGVs from the Illinois Administrative Code [33]. Table S13 lists 95 for the pollutants considered, where 19 of them are calculated from soil screening level equations. The C_2_ and C_3_ values are 0.94 and 1.04 that are about one order of magnitude higher than the average median and geometric mean. The C_4_ and C_5_ values are 43 and 45, which about 70% of the values are higher than the test statistics. Overall, Illinois’s RGVs are higher than the test statistics by one order of magnitude.

### 3.13 Indiana

Indiana has one set of RGVs from the Indiana Department of Environmental Management [34]. Table S14 has 96 RGVs for the pollutants considered. The C_2_ and C_3_ values are 0.89 and 1.03 that are about one order of magnitude higher than the average. The C_4_ and C_5_ values are 72 and 70 that more than 70% of the values are larger than the test statistics. Overall, Indiana’s RGVs are higher than the average median by 0.89 order of magnitude, and higher than the average geometric mean by 1.03 order of magnitude.

### 3.14 Iowa

Iowa has one set of RGVs from the Iowa Department of Natural Resources [35]. Table S15 has 94 RGVs of pollutants. The C_2_ and C_3_ values are 1.11 and 1.21, which standards are more than one order of magnitude higher than the test statistics. The C_4_ and C_5_ values are 60 and 76, where about 82% and 90% of the values are higher than the average. Overall, Iowa’s RGVs are higher than the test statistics by more than one order of magnitude.

### 3.15 Kansas

Kansas has one set of RGVs from the Kansas Department of Health and Environment [36]. Table S16 has 86 RGVs for the pollutants considered. The scores of C_2_ and C_3_ are 0.84 and 1.06 that about one order of magnitude higher than the average.The scores of C_4_ and C_5_ are 64 and 74, which are about 90% of the values higher than the test statistics. Overall, Kansasis’s RGVs are higher than the test statistics by about one order of magnitude.

### 3.16 Louisiana

Louisiana has two sets of RGVs from the Louisiana Department of Environmental Quality [37]. Table S17a presents analysis of values from Table S1 of the LaDEQ indicating screening standards for soil. It has 79 RGVs for the pollutants considered. The scores of C_2_ and C_3_ are 0.10 and 0.14, which are close to the average. The scores of C_4_ and C_5_ are −25 and −15, which more values are lower than the test statistics.

Table S17b presents analysis from Table S2 of the LaDEQ indicating management [37] option standards for soil. It also has 79 RGVs for the pollutants considered. The C_2_ and C_3_ values are 0.76 and 0.78 regulating by one order of magnitude higher than the average median and geometric mean. The C_4_ and C_5_ values are 21 and 27, where more than 60% of the values are higher than the test statistics.

Overall, Louisiana’s RGVs are higher than the test statistics by more than half order of magnitude.

### 3.17 Maine

Maine has one set of RGVs from the Main Department of Environmental Protection [38]. Table S18 has 82 RGVs for the pollutants considered. The scores of C_2_ and C_3_ are 1.34 and 1.35 that are higher than the average by more than one order of magnitude. The scores of C_4_ and C_5_ are 74 and 66 that over 90% of regulated pollutants have higher values than the test statistics. Overall, Maine’s RGVs are higher than the test statistics by more than one order of magnitude.

### 3.18 Maryland

Maryland has one set of RGVs from the Maryland Department of the Environment [39]. Table S19 presents 81 RGVs for the pollutants considered. The scores of C_2_ and C_3_ are 0.33 and 0.38 that standards are slightly higher than the average. The scores of C_4_ and C_5_ are both 9, which more values are higher than the test statistics rather below. Overall, Maryland’s RGVs are higher than the central tendencies within half order of magnitude.

### 3.19 Massachusetts

Massachusetts has seven sets from the Massachusetts Department of Environmental Protection [40]. Massachusetts Contingency Plan (MCP, 310 CMR 40.0000) provides three options for defining risk level. Method 1 uses clear numeric standards for more than 100 common chemicals in soil and groundwater; Method 2 allows for some adjustments in these standards to reflect site-specific conditions; and Method 3 allows cleanup requirement goals to be defined on the basis of a site-specific risk assessment. In addition, category S-1 soils are associated with the highest potential for exposure, and category S-3 soils have the lowest potential for exposure. There are 76 pollutants regulated for each jurisdiction with missing of some metals and current use pesticides.

Table S20a presents analysis based on method 1 for category S-1 soil overlying GW-3. The scores of C_2_ and C_3_ are 0.67 and 0.7. Generally, pollutants are regulated more than half order of magnitude higher than the average median and geometric mean. The scores of C_4_ and C_5_ are both 40. In specific, there are about 76% of the values higher than the average. Overall Massachusetts States has regulated pollutants by about one order of magnitude higher than the test statistics.

Table S20b lists RGVs based on method 1 for category S-2 soil overlying GW-3. The scores of C_2_ and C_3_ are 1.09 and 1.17 that standards are higher than the test statistics by one order of magnitude. C_4_ and C_5_ are 58 and 60 indicating at least 88% of the values are higher than the central tendencies.

Table S20c prensents analysis using method 1 for category S-3 soil overlying GW-3. The C_2_ and C_3_ values are 1.39 and 1.47, which standards are higher than the average by more than one order of magnitude. The C_4_ and C_5_ values are both 60 indicating about 89% of the values are higher the test statistics rather below.

Table S20d lists RGVs using method 2 for category S-1 soil directly contact exposure. The scores of C_2_ and C_3_ are 0.79 and 0.83 that standards are higher than the average by about one order of magnitude. The scores of C_4_ and C_5_ are 50 and 48, where about 82% of the values are higher than the test statistics.

Table S20e presents analysis of RGVs using method 2 for category S-2 soil directly contact exposure. The scores of C_2_ and C_3_ are 1.29 and 1.37 that standards are higher than the average by one order of magnitude. The scores of C_4_ and C_5_ are 68 and 70, which about 95% of the values are higher than the average.

Table S20f lists RGVs based on method 2 for category S-3 soil directly contact exposure. The scores of C_2_ and C_3_ are 1.70 and 1.74, which standards are higher than the test statistics by about two orders of magnitude. The scores of C_4_ and C_5_ are 69 and 71, where about 95% of the values are higher than the central tendencies.

Table S20g lists RGVs based on method 3: upper concentration limits in soil. The scores of C_2_ and C_3_ are 2.39 and 2.45, where standards are higher than the average by two orders of magnitude. The scores of C_4_ and C_5_ are both 74, which about 99% of the values are higher than the test statistics.

Overall, Massachusetts’s RGVs are higher than the test statistics. The range of order of magnitude varies with each method and category. The set of RGVs based on Method 1 has the lowest order of magnitude, and the one based on method 3 has the highest. The set of RGVs for S-1 type of soil has the lowest order of magnitude, and the one for S-3 type of soil has the highest.

### 3.20 Michigan

Michigan has one set of RGVs from the Michigan Department of Environmental Quality [41]. Table S21 has 95 RGVs for the pollutants considered. The scores of C_2_ and C_3_ are 1.77 and 1.82 that standards are higher than the average by about two orders of magnitude. The scores of C_4_ and C_5_ are 91 and 89, where about 97% of the values are higher than the test statistics. Overall, Michigan’s RGV are higher than the central tendencies by about two orders of magnitude.

### 3.21 Minnesota

Minnesota has one set of RGVs from the Minnesota Pollution Control Agency [42]. Table S22 lists 72 RGVs for the pollutants considered. Among them, missing pollutants are carcinogenic PAH, current use pesticides, and miscellaneous pollutants. The scores of C_2_ and C_3_ are 0.61 and 0.67 that standards are higher than the test statistics by about one order of magnitude. The scores of C_4_ and C_5_ are 24 and 44, where about 67% of the values are higher than the average median, and 81% are higher than the average geometric mean. Above all, Minnesota’s RGVs are higher than the central tendencies by about one order of magnitude.

### 3.22 Mississippi

Mississippi has one set of RGVs from the Mississippi Department of Environmental Quality [43]. Table S23 presents 97 RGVs for the pollutants considered, which the max count in this study is. The C_2_ and C_3_ scores are 0.57 and 0.69 that standards are higher than the test statistics by about half order of magnitude. The C_4_ and C_5_ scores are 33 and 31, where about 67% and 69% of the values are higher than the average median and geometric mean. Overall, Mississippi’s RGVs are higher than the test statistics by about half order of magnitude.

### 3.23 Missouri

Missouri has three sets of RGVs from the Missouri Department of Natural Resources [44]. There are three type of soil type—Sandy, Silty, and Clayey for residential land use. There are 92 pollutants that have RGVs for each jurisdiction. Table S24a presents standards based on sandy soil. The values for C_2_ and C_3_ are 1.05 and 1.20 that standards are higher than the average by about one order of magnitude. The values for C_4_ and C_5_ are 68 and 78, where about 87% and 92% of the values are higher than the average median and geometric mean. The other two, Table S24b contains RGVs in silty soil, and Table S24c contains RGVs in Clayey soil, have the same values as the ones in sandy soil.

Therefore, Missouri actually has one set of data for three different types of soils. And about 90% of the standards have been regulated more than one order of magnitude higher than the test statistics.

### 3.24 Nebraska

Nebraska has one set of RGVs from the Nebraska Department of Environmental Quality [45]. Table S25 has 93 RGVs for the pollutants considered. The C_2_ and C_3_ values are 0.26 and 0.28, which standards are slightly higher than the average. The C_4_ and C_5_ values are −19 and 3, where about 60% of the values are are lower than the average median, while about 52% of the values are higher than the average geometric mean. Overall, Nebraksa’s RGVs are lower than the average median, while higher than the average geometric mean, by order of magnitude close to the central tendencies.

### 3.25 Nevada

Nevada has two sets of RGVs from the Nevada Department of Environment Protection [46]. One is based on discovery events, the other is based on comparison level. Table S26a lists the RGVs based on discovery events. It has 93 RGVs for the pollutants considered. The C_2_ and C_3_ values are −0.20 and −0.11 that slightly lower than the test statistics. The C_4_ and C_5_ values are −41 and −23, where about 70% of the values are lower than the average median, and about 62% are lower than the average geometric mean. Overall, Nevada’s RGVs are slightly lower than the test statistics.

Table S26b presents the RGVs based on comparison level with 96 standards for the pollutants considered. The scores of C_2_ and C_3_ are 0.47 and 0.58, which standards are higher than the test statistics. The scores of C_4_ and C_5_ are 22 and 32, where about 61% of the values are higher than the average median, and 67% are higher than the average geometric mean.

Above all, Nevada’s RGVs are higher than the central tendencies by about half order of magnitude.

### 3.26 New Hampshire

New Hampshire has three sets of RGVs. Two are from the New Hampshire Department of Environmental Services [47], the other is from the New Hampshire Code of Administrative Rules [48]. Table S27a presents RGVs based on method 1 from the NhDES, which is a detection method for residential soil. It has 80 RGVs for the pollutants considered. Among missing of pollutants are metals, phenols, and current use pesticides. The scores of C_2_ and C_3_ are −0.1 and 0, which standards are slightly lower than the average median, and has the same order of magnitude as the average geometric mean. The scores of C_4_ and C_5_ are both −10, where about 56% of the values are are lower than the test statistics.

Table S27b lists RGVs based on method 1 for sensitive uses of property and accessible soils. It has 77 RGVs. It has the missing pollutants of metals, phenols, and current use pesticides. The C_2_ and C_3_ values are 0.70 and 0.75, which standards are higher than the average by about one order of magnitude. The C_4_ and C_5_ values are 29 and 33, where about 70% of the values are higher than the test statistics.

Table S27c presents 82 RGVs for the pollutants considered. It has missing pollutants of metals, benzenes, and current use pesticides. The scores of C_2_ and C_3_ are −0.11 and −0.012 that standards are close to the test statistics. The scores of C_4_ and C_5_ are both −12, where about 57% of the values are below the central tendencies.

Therefore, New Hampshire’s RGV are various in methods and categories. Two jurisdiction (Tables S27a and S27c) have the values are close to the average, while the other (Table S27b) has regulated 70% of the values higher than the average by about one order of magnitude.

### 3.27 New Jersey

New Jersey has two sets of RGVs. One (Table S28a) is from the New Jersey Administrative Code [49], the other (Table S28b) is from the New Jersey Department of Environmental Protection [50]. Table S28a has 77 RGVs for the pollutants considered. Among them, missing pollutants are metals, current use pesticides, and miscellaneous pollutants. The scores of C_2_ and C_3_ are 0.68 and 0.77, which standards are higher than the test statistics by about one order of magnitude. The scores of C_4_ and C_5_ are 9 and 25, where 56% of the values are higher than the average median and 66% of the values are higher than the average geometric mean.

Table S28b lists 76 RGVs of pollutants considered. It has missing pollutants of PAH and current use pesticides. The scores of C_2_ and C_3_ are 0.61 and 0.67, which standards are higher than the test statistics by about one order of magnitude. The scores of C_4_ and C_5_ are 18 and 26, where about 62% of the values are higher than the average median, and 67% of the values are higher than the average geometric mean.

Overall, New Jersey’s RGVs are higher than the test statistics by about one order of magnitude.

### 3.28 New Mexico

New Mexico has one set of RGVs from the New Mexico Environment Department [51]. Table S29 lists 78 RGVs of pollutants considered. It has missing pollutants of metals, PAH, current use pesticides, and miscellaneous pollutants. The C_2_ and C_3_ values are 0.96 and 1.04, which standards are higher than the average by one order of magnitude. The C_4_ and C_5_ values are 56 and 58, where about 86% of the values are higher than the average median, and 87% of the values are higher than the average geometric mean. Overall, New Mexico’s RGVs are higher than the test statistics by about one order of magnitude.

### 3.29 New York

New York State has three sets of RGVs from the New York Department of Environmental Conservation [52]. Since first jurisdiction, Supplemental Soil Cleanup Objectives (SSCOs), is a complement of NYCRR375-6.8, which the other two jurisdictions come from, it can also combine to two jurisdictions for New York State. However, due to publish time are different, all update and original values are list separately so that total three sets of data are listed.

Table S30a that lists SSCOs has 31 RGVs for the pollutants considered. The scores of C_2_ and C_3_ are −0.35 and −0.29, which standards are slightly lower than the average. The scores of C_4_ and C_5_ are both −17, where about 77% of the values are lower than the test statistics.

Table S30b has 64 RGVs for pollutants considered based on unrestricted use land. The C_2_ and C_3_ values are −0.81 and −0.83, which standards are lower than the average by about one order of magnitude. The C_4_ and C_5_ values are both −52, where about 90% of the values are lower than the test statistics.

Table S30c has 63 RGVs for the pollutants considered based on restricted residential land. The values of C_2_ and C_3_ are 0.048 and 0.032, which standards are close to the test statistics. The values of C_4_ and C_5_ are −9 and −17, where about 57% of the values are lower than the average median, and 63% are lower than the average geometric mean.

Generally, New York’s RGV are lower than the average. Based on different soil category, the order of magnitude ranges from −0.83 to −0.29.

### 3.30 North Carolina

North Carolina has three sets of RGVs from the North Carolina Environmental Quality [53]. One is based on the “contained-out” soil that the concentrations at which a soil is determined to no longer contain listed hazardous waste; another is the maximum soil contaminant concentration levels (MSCCs), the other is the preliminary soil remediation goals (PSRG). Table S31a that summarized standards for contained-out soil lists 88 RGVs for the pollutants considered. It has some missing values of metals and current use pesticides. The scores of C_2_ and C_3_ are −1.14 and −1.10 that standards are lower than the test statistics by about one order of magnitude. The scores of C_4_ and C_5_ are −66 and −70, where about 88% of the values are lower than the average median, and about 90% of the values are lower than the average geometric mean.

Table S31b that concluded MSCCs lists 52 RGVs for the pollutants considered. Amony some missings of pollutants are metals, benzenes, and phenols. The C_2_ and C_3_ values are 0.81 and 0.85 that pollutants are regulated more than half order of magnitude higher. The C_4_ and C_5_ values are 26 and 34, where about 75% of the values are higher than the average median, and about 83% of the values are higher than the average geometric mean.

Table S31c that summarized PSRG presents 95 RGVs for the pollutants considered. The scores of C_2_ and C_3_ are 0.14 and 0.19 that pollutants are regulated slightly higher than the average. The scores of C_4_ and C_5_ are −25 and −9, where about 63% of the values are lower than the average median, and about 55% of the values are lower than the average geometric mean.

There is no common pattern of values how North Carolina is regulating, where RGVs from each jurisdiction varies.

### 3.31 Ohio

Ohio has two sets of RGVs. One is from the Ohio Administrative Code [54], the other is from the Ohio Environmental Protection Agency [55]. Table S32a summarizes generic numerical standards from OhAC. It lists 68 RGVs and miss some of current use pesticides, metals, methanes, benzenes, and phenols. The scores of C_2_ and C_3_ are 1.13 and 1.22 that are higher than the test statistics by more than one order of magnitude. The scores of C_4_ and C_5_ are 62 and 64, where about 96% and 97% of the values are above the central tendencies.

Table S32b concludes generic numerical standards from OhEPA. It lists 87 RGVs for the pollutants considered. Among missing of pollutants are metals, and current use pesticides. The C_2_ and C_3_ values are 1.33 and 1.39 that are higher than the average by more than one order of magnitude. The C_4_ and C_5_ values are 81 and 79, where about 96% of the values are higher than the test statsistics.

Therefore, Ohio’s RGV provide values are higher than the average by more than one order of magnitude.

### 3.32 Oregon

Oregon State has five sets of RGVs from the Oregon Department of Environmental Quality [56] including soil category of residential, urban residential, hot spot concentration (HSC) residential, HSC urban residential, and catch basin screening. Table S33a summarizes residential soil that lists 65 RGVs for the pollutants considered. It has some missing pollutants of metals, phenols, PAH, pesticides, and miscellaneous pollutants. The scores of C_2_ and C_3_ are 0.55 and 0.57 that are higher than the average by about half order of magnitude. The scores of C_4_ and C_5_ are 11 and 21, where about 58% of the values are higher than the average median and about 66% are higher than the average geometric mean.

Table S33b based on urban residential soil that presents 65 RGVs. The C2 and C3 values are 0.92 and 0.95 that are higher than the test statistics by about one order of magnitude. The C4 and C5 values are 35 and 31, where about 77% of the values are higher than the average median and about 74% are higher than the average geometric mean.

Table S33c summarizes HSC residential soil that has 64 RGVs. The C_2_ and C_3_ values are 2.02 and 2.03 that standards are higher than the average by more than two orders of magnitude. The C_4_ and C_5_ values are both 62, where about 98% of the values are higher than the average.

Table S33d summarizes HSC urban residential soil that has 63 RGVs for the pollutants considered. The scores of C_2_ and C_3_ are 2.33 and 2.38 that are higher than the test statistics by more than two orders of magnitude. The scores of C_4_ and C_5_ are 61 and 63, where about 95% of the values are higher than the average median and 97% are higher than the average geometric mean.

Table S33e summarizes catch basin screening level that has 47 RGVs for the pollutants considered from Oregon Dept. of Environmental Quality [57]. The C_2_ and C_3_ values are −1 and −1.02 that standards are lower than the average by one order of magnitude. The C_4_ and C_5_ values are both −37, where about 89% of the values are lower than the test statistics.

### 3.33 Pennsylvania

Pennsylvania has one set of RGVs from the Pennsylvania Code [58]. It has 95 RGVs for the pollutants considered that combined organic and inorganic chemicals. The C_2_ and C_3_ values are both 1.38 that are higher than the average by more than one order of magnitude. The C_4_ and C_5_ values are both 79, where about 92% of the values are higher than the test statistics (Table S34)

### 3.34 Rhode Island

Rhode Island State has one set of RGVs from the Rhode Island Department of Environmental Management [59]. Table S35 lists 71 RGVs for the pollutants considered. Among missing of pollutants metals and pesticides. The scores of C_2_ and C_3_ are 0.24 and 0.28 that standards are slightly higher than the test statistics. The scores of C_4_ and C_5_ are 3 and 9, where about 53% of the values are higher than the average median and 56% are higher than the average geometric mean.

### 3.35 South Carolina

South Carolina has one set of RGVs from the South Carolina Department of Health and Environmental Control [60]. Table S36 lists only 11 RGVs that most are PAH. The scores of C_2_ and C_3_ are 1.09 and 1.18 that are higher than the test statistics by more than one order of magnitude. The scores of C_4_ and C_5_ are both 5, where are higher than the average.

### 3.36 South Dakota

South Dakota has one set of RGVs from the South Dakota Department of Environment & Natural Resources [61]. Table S37 lists only 6 RGVs that most are benzenes. The scores of C_2_ and C_3_ are both 1.5 that pollutants are regulated higher than the average by more than 1.5 order of magnitude. The scores of C_4_ and C_5_ are both 6, where all the values are higher than the average.

### 3.37 Texas

Texas has two sets of RGVs from the Texas Commission on Environmental Quality [62]. One is 0.5-acre source area, the other is 30-acre source area. They are based on total soil comb that includes inhalation, ingestion, dermal, and vegetable consumption pathways. Table S38a lists 96 RGVs for the pollutants considered based on 0.5-acre source area. The C_2_ and C_3_ values are 0.94 and 1.11 that pollutants are higher than the test statistics by about one order of magnitude. The C_4_ and C_5_ values are 70 and 72, where 86% of the values are higher than the average median and 88% are higher than the average geometric mean.

Table S38b based on 30 acre course area also presents 96 RGVs. The C_2_ and C_3_ values are 0.92 and 1.05 that are higher than the test statistics by about one order of magnitude. The C_4_ and C_5_ values are 68 and 70, where 85% of the values are higher than the average median and 86% are higher than the geometric mean.

### 3.38 Utah

Utah has one set of RGVs from the Utah Department of Environmental Quality [63]. Table S39 lists only 6 RGVs. The scores of C_2_ and C_3_ are −0.33 and −0.5 that standards are slightly lower than the test statistics. The scores of C_4_ and C_5_ are −4 and −2, where most values are lower than the average.

### 3.39 Vermont

Vermont has one set of RGVs from the Vermont Department of Environment [64]. Table S40 lists 96 RGVs for the pollutants of consideration. The C_2_ and C_3_ values are 0.40 and 0.54 that standards are higher than the test statistics by about half order of magnitude. The C_4_ and C_5_ values are −8 and 18, where about 54% of the values are lower than the average median and 59% are higher than the average geometric mean.

### 3.40 Virginia

Virginia has two sets of RGVs from the Virginia Department of Environmental Quality [65]. Table S41a presents analysis for the regional screening level (RSL). It has 76 RGVs for the pollutants considered. Among missing of pollutants are PAH and pesticides. The C_2_ and C_3_ values are 0.18 and 0.33 that are slightly higher than the test statistics. The C_4_ and C_5_ values are 6 and 22, where 54% of the values are higher than the average median and 64% are higher than the average geometric mean.

Table S41b presents analysis based on the Virginia’s Voluntary Remediation Program (VRP) that has 77 RGVs for the pollutants considered. The scores of C_2_ and C_3_ are −0.70 and −0.62 that are lower than the test statistics by about one order of magnitude. The scores of C_4_ and C_5_ are −43 and −51, where about 78% of the values are lower than the average median and 83% are lower than the average geometric mean.
