# Supplementary material for: Regulatory performance dataset constructed from U.S. soil jurisdictions based on the top 100 concerned pollutants
Source: Data Brief. 2018 Sep 27;21:36–49. doi: 10.1016/j.dib.2018.09.049 (PMC6186953; doi:10.1016/j.dib.2018.09.049)
Supplement: Supplementary file 1 — Supplementary material [file mmc1.doc]

# DISCLOSURE OF POTENTIAL CONFLICT-OF-INTEREST

**All accepted articles will be published only after the signed disclosure statements have been completed. The information will be published as a footnote to the article on the Title page.**

| **Please respond to each of the following questions by checking the appropriate boxes.**  **Each author must complete his/her own form.** |
| --- |

| 1. | Did the author of the manuscript receive funding, grants, or in-kind support in support of the research or the preparation of the manuscript? | | | |  |
| --- | --- | --- | --- | --- | --- |
|  | x | NO | | |  |
|  |  |  | | |  |
|  |  | YES, support received from the following persons, agencies, industrial or commercial parties is disclosed below. | | |  |
|  |  | If yes, did the support include contractual or implied restriction on utilization or publication of the data and/or review of the data prior to publication? | | |  |
|  | |  |  | NO | |
|  | |  |  |  | |
|  | |  |  | YES | |
|  |  |  | | |  |
| 2 | Did the author have association or financial involvement (i.e. consultancies/advisory board, stock ownerships/options, equity interest, patents received or pending, royalties/honorary) with any organization or commercial entity having a financial interest in or financial conflict with the subject matter or research presented in the manuscript? | | | |  |
|  | x | NO | | |  |
|  |  |  | | |  |
|  |  | YES, the association or financial involvement of the authors is disclosed below. | | |  |
|  |  |  | | |  |
|  |  |  | | |  |
|  |  |  | | |  |
|  |  | (Use additional sheets if necessary) | | |  |

**Author Name/Signature: Zijian Li**

## Title of Article: Regulatory performance dataset constructed from U.S. soil jurisdictions based on the top 100 concerned pollutants

## Manuscript Number: DIB-D-18-02043

## Date: 8/16/2018
