# Supplementary material for: Regulatory performance dataset constructed from U.S. soil jurisdictions based on the top 100 concerned pollutants
Source: Data Brief. 2018 Sep 27;21:36–49. doi: 10.1016/j.dib.2018.09.049 (PMC6186953; doi:10.1016/j.dib.2018.09.049)
Supplement: Supplementary file 2 — Supplementary material [file mmc2.docx]

Table S1. One hundred contaminants and their CAS number.

| No. | Contaminant | CAS No. |
| --- | --- | --- |
| Elements | | |
| 1 | Antimony | 7440-36-0 |
| 2 | Arsenic | 7440-38-2 |
| 3 | Barium | 7440-39-3 |
| 4 | Beryllium | 7440-41-7 |
| 5 | Cadmium | 7440-43-9 |
| 6 | Chromium (III) | 16065-83-1 |
| 7 | Chromium (VI) | 18540-29-9 |
| 8 | Cobalt | 7440-48-4 |
| 9 | Copper | 7440-50-8 |
| 10 | Lead | 7439-92-1 |
| 11 | Manganese | 7439-96-5 |
| 12 | Mercury | 7439-97-6 |
| 13 | Molybdenum | 7439-98-7 |
| 14 | Nickel | 7440-02-0 |
| 15 | Selenium | 7782-49-2 |
| 16 | Silver | 7440-22-4 |
| 17 | Thallium | 7440-28-0 |
| 18 | Tin | 7440-31-5 |
| 19 | Vanadium | 7440-62-2 |
| 20 | Zinc | 7440-66-6 |
| Cyanides | | |
| 21 | Cyanide | 57-12-5 |
| 22 | Acrylonitril (Vinyl cyanide, 2-Propenenitrile) | 107-13-1 |
| Halogenated Methanes | | |
| 23 | Dichloromethane (methylene chloride) | 75-09-2 |
| 24 | Trichloromethane (chloroform) | 67-66-3 |
| 25 | Tetrachloromethane (carbon tetrachloride) | 56-23-5 |
| 26 | Bromoform | 75-25-2 |
| 27 | Bromomethane (methyl bromide) | 74-83-9 |
| Chloroethanes and Choroethenes | | |
| 28 | 1,2-Dichloroethane | 107-06-2 |
| 29 | 1,1,1-Trichloroethane | 71-55-6 |
| 30 | 1,1,2-Trichloroethane | 79-00-5 |
| 31 | Chloroethene (vinyl chloride) | 75-01-4 |
| 32 | 1,1-Dichloroethene (vinylidene chloride) | 75-35-4 |
| 33 | Trichloroethene (ethylene trichloride) | 79-01-6 |
| 34 | Tetrachloroethene (perchloroethylene) | 127-18-4 |
| Benzenes | | |
| 35 | Benzene | 71-43-2 |
| 36 | Toluene (methylbenzene) | 108-88-3 |
| 37 | Chlorobenzene | 108-90-7 |
| 38 | 1,2-Dichlorobenzene | 95-50-1 |
| 39 | 1,3-Dichlorobenzene | 541-73-1 |
| 40 | 1,4-Dichlorobenzene | 106-46-7 |
| 41 | 1,2,4Trichlorobenzene | 120-82-1 |
| 42 | Hexachlorobenzene | 118-74-1 |
| 43 | Nitrobenzene | 98-95-3 |
| 44 | Ethylbenzene | 100-41-4 |
| 45 | Styrene | 100-42-5 |
| 46 | Xylenes (Dimethyl benzene) | 1330-20-7 |
| Phenol | | |
| 47 | Cresol | 1319-77-3 |
| 48 | Phenol | 108-95-2 |
| 49 | 2-Chlorophenol | 95-57-8 |
| 50 | 2,4-Dichlorophenol | 120-83-2 |
| 51 | 2,4,5-Trichlorophenol | 95-95-4 |
| 52 | 2,4,6-Trichlorophenol | 88-06-2 |
| 53 | 2,3,4,6-Tetrahlorophenol | 58-90-2 |
| 54 | Pentachlorophenol | 87-86-5 |
| Carcinogenic PAH | | |
| 55 | Benz(a)anthracene | 56-55-3 |
| 56 | Benzo(a)pyrene | 50-32-8 |
| 57 | Benzo(b)fluoranthene | 205-99-2 |
| 58 | Benzo(k)fluoranthene | 207-08-9 |
| 59 | Chrysene | 218-01-9 |
| 60 | Dibena(a,h)anthracene | 53-70-3 |
| 61 | Indeno(1,2,3-c,d)pyrebe | 193-39-5 |
| 62 | Naphthalene | 91-20-3 |
| Noncarcinogenic PAH | | |
| 63 | Acenaphehene | 83-32-9 |
| 64 | Acenaphthylene | 208-96-8 |
| 65 | Anthracene | 120-12-7 |
| 66 | Benzo(g,h,i) perylene | 191-24-2 |
| 67 | Flouranthene | 206-44-0 |
| 68 | Flouorene | 86-73-7 |
| 69 | Phenanthrene | 85-01-8 |
| 70 | Pyrene | 129-00-0 |
| Historical Pesticides | | |
| 71 | Aldrin | 309-00-2 |
| 72 | Chlordane | 57-74-9 |
| 73 | DDT (Dichlorodiphenyl-trichloroethane) | 50-29-3 |
| 74 | Dieldrin | 60-57-1 |
| 75 | Endosulfan | 115-29-7 |
| 76 | Endrin | 72-20-8 |
| 77 | Heptachlor | 76-44-8 |
| 78 | Lindane (Hexachlorocyclohexane Gamma-HCH) | 58-89-9 |
| 79 | Toxaphene | 8001-35-2 |
| Current Pesticides | | |
| 80 | 2,4-Dichlorophenoxy Acetic Acid (2,4-D, Weed-B-Gon, Aqua Kleen) | 94-75-7 |
| 81 | Atrazine | 1912-24-9 |
| 82 | Carbaryl (Sevin) | 63-25-2 |
| 83 | Carbofuran (Furadan) | 1563-66-2 |
| 84 | Chloryrifos (Chlorpyrifos) | 2921-88-2 |
| 85 | Diuron (DCMU) | 330-54-1 |
| 86 | Gylphosate (Roundup) | 1071-83-6 |
| 87 | Malathion | 121-75-5 |
| 88 | 2-methyl-4-chlorophenoxy acetic acid (MCPA, Weed-B-Gone) | 94-74-6 |
| 89 | Picloram | 1918-02-1 |
| 90 | Simazine | 122-34-9 |
| 91 | Trifluralin | 1582-09-8 |
| Miscellaneous Pollutants | | |
| 92 | Total PCB | 1336-36-3 |
| 93 | PCB 118 | 31508-00-6 |
| 94 | Total PCDD/PCDF | 1746-01-6 |
| 95 | Methyl ethyl ketone (2-Butanoe, MEK) | 78-93-3 |
| 96 | Methyl isobutyl ketone (MIBX) | 108-10-1 |
| 97 | Dibutyl phthalate | 84-74-2 |
| 98 | Bis(2-ethylhexyl) phthalate (DEHP) | 117-81-7 |
| 99 | Hexachlorobutadine | 87-68-3 |
| 100 | Methyl tert-butyl ether (MTBE) | 1634-04-4 |

**Table S2.** Alabama Dept. of Environmental Management

| Number | Pollutant | CAS. No | RGV (mg/kg) | C_2_ | C_3_ | C_4_ | C_5_ |
| --- | --- | --- | --- | --- | --- | --- | --- |
| 1 | Antimony | 7440-36-0 | 3.1E+00 | -1 | 0 | -1 | -1 |
| 2 | Arsenic | 7440-38-2 | 4.0E-01 | -1 | -1 | -1 | -1 |
| 3 | Barium | 7440-39-3 | 5.4E+02 | 0 | 0 | -1 | -1 |
| 4 | Beryllium | 7440-41-7 | 1.0E+04 | 3 | 3 | 1 | 1 |
| 5 | Cadmium | 7440-43-9 | 1.5E+01 | 1 | 1 | 1 | 1 |
| 6 | Chromium (III) | 16065-83-1 | 1.0E+05 | 3 | 2 | 1 | 1 |
| 7 | Chromium (VI) | 18540-29-9 | 3.0E+01 | 0 | 0 | -1 | -1 |
| 8 | Cobalt | 7440-48-4 | 9.0E+02 | 1 | 1 | 1 | 1 |
| 9 | Copper | 7440-50-8 | 3.1E+02 | 0 | 0 | 1 | 1 |
| 10 | Lead | 7439-92-1 | 4.0E+02 | 0 | 1 | 1 | 1 |
| 11 | Manganese | 7439-96-5 | 1.8E+02 | -1 | -1 | -1 | -1 |
| 12 | Mercury | 7439-97-6 | 2.3E+00 | 0 | 0 | -1 | -1 |
| 14 | Nickel | 7440-02-0 | 1.6E+02 | 0 | 0 | 1 | -1 |
| 15 | Selenium | 7782-49-2 | 3.9E+01 | 0 | 0 | 1 | 1 |
| 16 | Silver | 7440-22-4 | 3.9E+01 | 0 | 0 | -1 | -1 |
| 17 | Thallium | 7440-28-0 | 5.2E-01 | -1 | -1 | -1 | -1 |
| 18 | Tin | 7440-31-5 | 4.7E+03 | 2 | 1 | 1 | 1 |
| 19 | Vanadium | 7440-62-2 | 7.8E+00 | -1 | -1 | -1 | -1 |
| 20 | Zinc | 7440-66-6 | 2.3E+03 | 1 | 1 | 1 | 1 |
| 21 | Cyanide | 57-12-5 | 1.2E+02 | 1 | 1 | 1 | 1 |
| 22 | Acrylonitril | 107-13-1 | 2.1E-01 | -1 | -1 | -1 | -1 |
| 23 | Dichloromethane | 75-09-2 | 9.1E+00 | 0 | 0 | -1 | -1 |
| 24 | Trichloromethane | 67-66-3 | 2.2E-01 | 0 | -1 | -1 | -1 |
| 25 | Tetrachloromethane | 56-23-5 | 2.5E-01 | 0 | 0 | -1 | -1 |
| 26 | Bromoform | 75-25-2 | 6.2E+01 | 0 | 0 | -1 | 1 |
| 27 | Bromomethane | 74-83-9 | 3.9E-01 | -1 | -1 | -1 | -1 |
| 28 | 1,2-Dichloroethane | 107-06-2 | 2.8E-01 | -1 | 0 | -1 | -1 |
| 29 | 1,1,1-Trichloroethane | 71-55-6 | 1.2E+03 | 1 | 1 | 1 | 1 |
| 30 | 1,1,2-Trichloroethane | 79-00-5 | 7.3E-01 | 0 | 0 | -1 | -1 |
| 31 | Chloroethene | 75-01-4 | 3.0E+00 | 1 | 1 | 1 | 1 |
| 32 | 1,1-Dichloroethene | 75-35-4 | 1.2E+01 | 0 | 0 | -1 | 1 |
| 33 | Trichloroethene | 79-01-6 | 5.3E-02 | -2 | -2 | -1 | -1 |
| 34 | Tetrachloroethene | 127-18-4 | 4.8E-01 | -1 | -1 | -1 | -1 |
| 35 | Benzene | 71-43-2 | 6.4E-01 | 0 | 0 | -1 | -1 |
| 36 | Toluene | 108-88-3 | 5.2E+02 | 1 | 1 | 1 | 1 |
| 37 | Chlorobenzene | 108-90-7 | 1.5E+01 | 0 | 0 | -1 | -1 |
| 38 | 1,2-Dichlorobenzene | 95-50-1 | 6.0E+02 | 1 | 1 | 1 | 1 |
| 39 | 1,3-Dichlorobenzene | 541-73-1 | 5.3E+01 | 0 | 1 | 1 | 1 |
| 40 | 1,4-Dichlorobenzene | 106-46-7 | 3.4E+00 | 0 | 0 | -1 | -1 |
| 41 | 1,2,4Trichlorobenzene | 120-82-1 | 6.2E+00 | 0 | 0 | -1 | -1 |
| 42 | Hexachlorobenzene | 118-74-1 | 3.0E-01 | 0 | 0 | -1 | -1 |
| 43 | Nitrobenzene | 98-95-3 | 2.0E+00 | -1 | -1 | -1 | -1 |
| 44 | Ethylbenzene | 100-41-4 | 4.0E+02 | 1 | 1 | 1 | 1 |
| 45 | Styrene | 100-42-5 | 1.7E+03 | 2 | 2 | 1 | 1 |
| 46 | Xylenes | 1330-20-7 | 2.7E+01 | -1 | 0 | -1 | -1 |
| 48 | Phenol | 108-95-2 | 1.8E+03 | 1 | 1 | 1 | 1 |
| 49 | 2-Chlorophenol | 95-57-8 | 6.3E+00 | -1 | 0 | -1 | -1 |
| 50 | 2,4-Dichlorophenol | 120-83-2 | 1.8E+01 | 0 | 0 | -1 | 1 |
| 51 | 2,4,5-Trichlorophenol | 95-95-4 | 6.1E+02 | 0 | 1 | -1 | 1 |
| 52 | 2,4,6-Trichlorophenol | 88-06-2 | 6.1E-01 | -1 | -1 | -1 | -1 |
| 53 | 2,3,4,6-Tetrahlorophenol | 58-90-2 | 1.8E+02 | 0 | 1 | -1 | 1 |
| 54 | Pentachlorophenol | 87-86-5 | 3.0E+00 | 0 | 0 | -1 | -1 |
| 55 | Benz(a)anthracene | 56-55-3 | 6.2E-01 | 0 | 0 | -1 | -1 |
| 56 | Benzo(a)pyrene | 50-32-8 | 6.2E-02 | -1 | -1 | -1 | -1 |
| 57 | Benzo(b)fluoranthene | 205-99-2 | 6.2E-01 | 0 | 0 | -1 | -1 |
| 58 | Benzo(k)fluoranthene | 207-08-9 | 6.2E+00 | 0 | 0 | -1 | -1 |
| 59 | Chrysene | 218-01-9 | 6.2E+01 | 1 | 1 | 1 | 1 |
| 60 | Dibena(a,h)anthracene | 53-70-3 | 6.2E-02 | -1 | -1 | -1 | -1 |
| 61 | Indeno(1,2,3-c,d)pyrebe | 193-39-5 | 6.2E-01 | 0 | -1 | -1 | -1 |
| 62 | Naphthalene | 91-20-3 | 5.6E+00 | 0 | 0 | -1 | 1 |
| 63 | Acenaphehene | 83-32-9 | 3.7E+02 | 1 | 1 | 1 | 1 |
| 65 | Anthracene | 120-12-7 | 2.2E+03 | 2 | 1 | 1 | 1 |
| 66 | Benzo(g,h,i) perylene | 191-24-2 | 2.3E+02 | 1 | 1 | 1 | 1 |
| 67 | Flouranthene | 206-44-0 | 2.3E+02 | 2 | 1 | 1 | 1 |
| 68 | Flouorene | 86-73-7 | 2.7E+02 | 1 | 1 | 1 | 1 |
| 69 | Phenanthrene | 85-01-8 | 2.0E+02 | 1 | 1 | 1 | 1 |
| 70 | Pyrene | 129-00-0 | 2.3E+02 | 0 | 0 | 1 | 1 |
| 71 | Aldrin | 309-00-2 | 2.9E-02 | 0 | -1 | -1 | -1 |
| 72 | Chlordane | 57-74-9 | 1.6E+00 | 0 | 0 | -1 | -1 |
| 73 | DDT | 50-29-3 | 1.7E+00 | 0 | 0 | -1 | -1 |
| 74 | Dieldrin | 60-57-1 | 3.0E-02 | 0 | -1 | -1 | -1 |
| 75 | Endosulfan | 115-29-7 | 3.7E+01 | 0 | 1 | -1 | 1 |
| 76 | Endrin | 72-20-8 | 1.8E+00 | 0 | 0 | -1 | -1 |
| 77 | Heptachlor | 76-44-8 | 1.1E-01 | 0 | 0 | -1 | -1 |
| 78 | Lindane | 58-89-9 | 4.4E-01 | 0 | 0 | -1 | -1 |
| 79 | Toxaphene | 8001-35-2 | 4.4E-01 | 0 | 0 | -1 | -1 |
| 80 | 2,4-D | 94-75-7 | 6.9E+01 | -1 | 0 | -1 | -1 |
| 81 | Atrazine | 1912-24-9 | 2.2E+00 | 0 | 0 | -1 | -1 |
| 83 | Carbofuran | 1563-66-2 | 3.1E+01 | -1 | 0 | -1 | 1 |
| 84 | Chloryrifos | 2921-88-2 | 1.8E+01 | -1 | 0 | -1 | -1 |
| 86 | Gylphosate | 1071-83-6 | 6.1E+02 | -1 | 0 | -1 | -1 |
| 87 | Malathion | 121-75-5 | 1.2E+02 | -1 | 0 | -1 | -1 |
| 89 | Picloram | 1918-02-1 | 4.3E+02 | -1 | 0 | -1 | -1 |
| 90 | Simazine | 122-34-9 | 4.1E+00 | 0 | 0 | -1 | -1 |
| 92 | Total PCB | 1336-36-3 | 2.2E-01 | 0 | 0 | -1 | -1 |
| 94 | Total PCDD/PCDF | 1746-01-6 | 1.0E-03 | 1 | 1 | 1 | 1 |
| 95 | Methyl ethyl ketone | 78-93-3 | 2.2E+03 | 0 | 0 | -1 | 1 |
| 96 | Methyl isobutyl ketone | 108-10-1 | 5.3E+02 | -1 | 0 | -1 | -1 |
| 97 | Dibutyl phthalate | 84-74-2 | 6.1E+02 | -1 | 0 | -1 | -1 |
| 98 | DEHP | 117-81-7 | 3.5E+01 | 0 | 0 | -1 | -1 |
| 99 | Hexachlorobutadine | 87-68-3 | 6.2E+00 | 0 | 0 | -1 | 1 |
| 100 | Methyl tert-butyl ether | 1634-04-4 | 3.2E+01 | 0 | 0 | -1 | -1 |
|  |  | Overall | C1 | C2 | C3 | C4 | C5 |
|  |  | Scores | 92 | 0.054 | 0.15 | -36 | -18 |
|  |  |  |  |  | No. >0 | 28 | 37 |
|  |  |  |  |  | No. <0 | 64 | 55 |

**Table S3a** Alaska Dept. of Environmental Conservation arctic zone

| Number | Pollutant | CAS. No | RGV (mg/kg) | C_2_ | C_3_ | C_4_ | C_5_ |
| --- | --- | --- | --- | --- | --- | --- | --- |
| 1 | Antimony | 7440-36-0 | 5.50E+01 | 1 | 1 | 1 | 1 |
| 2 | Arsenic | 7440-38-2 | 6.10E+00 | 0 | 0 | -1 | -1 |
| 3 | Barium | 7440-39-3 | 2.74E+04 | 2 | 2 | 1 | 1 |
| 4 | Beryllium | 7440-41-7 | 2.70E+02 | 1 | 1 | 1 | 1 |
| 5 | Cadmium | 7440-43-9 | 1.10E+02 | 1 | 1 | 1 | 1 |
| 6 | Chromium (III) | 16065-83-1 | 2.05E+05 | 3 | 3 | 1 | 1 |
| 7 | Chromium (VI) | 18540-29-9 | 4.10E+02 | 1 | 1 | 1 | 1 |
| 9 | Copper | 7440-50-8 | 5.50E+03 | 2 | 2 | 1 | 1 |
| 10 | Lead | 7439-92-1 | 4.00E+02 | 0 | 1 | 1 | 1 |
| 12 | Mercury | 7439-97-6 | 4.10E+01 | 1 | 1 | 1 | 1 |
| 14 | Nickel | 7440-02-0 | 2.70E+03 | 2 | 1 | 1 | 1 |
| 15 | Selenium | 7782-49-2 | 6.80E+02 | 2 | 2 | 1 | 1 |
| 16 | Silver | 7440-22-4 | 6.80E+02 | 1 | 1 | 1 | 1 |
| 17 | Thallium | 7440-28-0 | 1.10E+01 | 1 | 1 | 1 | 1 |
| 19 | Vanadium | 7440-62-2 | 9.60E+02 | 1 | 1 | 1 | 1 |
| 20 | Zinc | 7440-66-6 | 4.11E+04 | 2 | 2 | 1 | 1 |
| 21 | Cyanide | 57-12-5 | 2.70E+03 | 2 | 2 | 1 | 1 |
| 23 | Dichloromethane | 75-09-2 | 1.50E+03 | 2 | 2 | 1 | 1 |
| 24 | Trichloromethane | 67-66-3 | 1.40E+03 | 3 | 3 | 1 | 1 |
| 25 | Tetrachloromethane | 56-23-5 | 8.60E+01 | 2 | 2 | 1 | 1 |
| 26 | Bromoform | 75-25-2 | 1.40E+03 | 2 | 2 | 1 | 1 |
| 27 | Bromomethane | 74-83-9 | 1.90E+02 | 2 | 2 | 1 | 1 |
| 28 | 1,2-Dichloroethane | 107-06-2 | 1.20E+02 | 2 | 2 | 1 | 1 |
| 29 | 1,1,1-Trichloroethane | 71-55-6 | 2.74E+04 | 2 | 2 | 1 | 1 |
| 30 | 1,1,2-Trichloroethane | 79-00-5 | 2.00E+02 | 2 | 2 | 1 | 1 |
| 31 | Chloroethene | 75-01-4 | 7.50E+00 | 2 | 2 | 1 | 1 |
| 32 | 1,1-Dichloroethene | 75-35-4 | 1.90E+01 | 0 | 1 | 1 | 1 |
| 33 | Trichloroethene | 79-01-6 | 2.80E+01 | 1 | 1 | 1 | 1 |
| 34 | Tetrachloroethene | 127-18-4 | 2.10E+01 | 1 | 1 | 1 | 1 |
| 35 | Benzene | 71-43-2 | 2.00E+02 | 3 | 3 | 1 | 1 |
| 36 | Toluene | 108-88-3 | 1.10E+04 | 2 | 2 | 1 | 1 |
| 37 | Chlorobenzene | 108-90-7 | 2.70E+03 | 2 | 2 | 1 | 1 |
| 38 | 1,2-Dichlorobenzene | 95-50-1 | 1.23E+04 | 2 | 2 | 1 | 1 |
| 39 | 1,3-Dichlorobenzene | 541-73-1 | 1.23E+04 | 3 | 3 | 1 | 1 |
| 40 | 1,4-Dichlorobenzene | 106-46-7 | 4.70E+02 | 2 | 2 | 1 | 1 |
| 41 | 1,2,4Trichlorobenzene | 120-82-1 | 1.40E+03 | 2 | 2 | 1 | 1 |
| 42 | Hexachlorobenzene | 118-74-1 | 4.30E+00 | 1 | 1 | 1 | 1 |
| 43 | Nitrobenzene | 98-95-3 | 6.80E+01 | 1 | 1 | 1 | 1 |
| 44 | Ethylbenzene | 100-41-4 | 1.37E+04 | 3 | 3 | 1 | 1 |
| 45 | Styrene | 100-42-5 | 2.74E+04 | 3 | 3 | 1 | 1 |
| 46 | Xylenes | 1330-20-7 | 2.74E+04 | 2 | 3 | 1 | 1 |
| 47 | Cresol | 1319-77-3 | 4.80E+02 | 1 | 1 | 1 | 1 |
| 48 | Phenol | 108-95-2 | 3.13E+04 | 3 | 2 | 1 | 1 |
| 49 | 2-Chlorophenol | 95-57-8 | 6.80E+02 | 1 | 2 | 1 | 1 |
| 50 | 2,4-Dichlorophenol | 120-83-2 | 3.10E+02 | 1 | 2 | 1 | 1 |
| 51 | 2,4,5-Trichlorophenol | 95-95-4 | 8.80E+03 | 1 | 2 | 1 | 1 |
| 52 | 2,4,6-Trichlorophenol | 88-06-2 | 6.20E+02 | 2 | 2 | 1 | 1 |
| 54 | Pentachlorophenol | 87-86-5 | 5.20E+01 | 1 | 1 | 1 | 1 |
| 55 | Benz(a)anthracene | 56-55-3 | 6.60E+00 | 1 | 1 | 1 | 1 |
| 56 | Benzo(a)pyrene | 50-32-8 | 6.60E-01 | 0 | 0 | -1 | -1 |
| 57 | Benzo(b)fluoranthene | 205-99-2 | 6.60E+00 | 1 | 1 | 1 | 1 |
| 58 | Benzo(k)fluoranthene | 207-08-9 | 6.60E+01 | 1 | 1 | 1 | 1 |
| 59 | Chrysene | 218-01-9 | 6.60E+02 | 2 | 2 | 1 | 1 |
| 60 | Dibena(a,h)anthracene | 53-70-3 | 6.60E-01 | 0 | 0 | 1 | -1 |
| 61 | Indeno(1,2,3-c,d)pyrebe | 193-39-5 | 6.60E+00 | 1 | 1 | 1 | 1 |
| 62 | Naphthalene | 91-20-3 | 1.90E+03 | 2 | 3 | 1 | 1 |
| 63 | Acenaphehene | 83-32-9 | 3.80E+03 | 2 | 2 | 1 | 1 |
| 64 | Acenaphthylene | 208-96-8 | 3.80E+03 | 2 | 2 | 1 | 1 |
| 65 | Anthracene | 120-12-7 | 2.78E+04 | 3 | 2 | 1 | 1 |
| 66 | Benzo(g,h,i) perylene | 191-24-2 | 1.90E+03 | 2 | 2 | 1 | 1 |
| 67 | Flouranthene | 206-44-0 | 2.50E+03 | 3 | 2 | 1 | 1 |
| 68 | Flouorene | 86-73-7 | 3.20E+03 | 2 | 2 | 1 | 1 |
| 69 | Phenanthrene | 85-01-8 | 2.78E+04 | 3 | 3 | 1 | 1 |
| 70 | Pyrene | 129-00-0 | 1.90E+03 | 1 | 1 | 1 | 1 |
| 71 | Aldrin | 309-00-2 | 4.00E-01 | 1 | 0 | 1 | 1 |
| 72 | Chlordane | 57-74-9 | 2.60E+01 | 1 | 1 | 1 | 1 |
| 73 | DDT | 50-29-3 | 2.90E+01 | 1 | 1 | 1 | 1 |
| 74 | Dieldrin | 60-57-1 | 4.30E-01 | 1 | 0 | 1 | 1 |
| 75 | Endosulfan | 115-29-7 | 8.20E+02 | 2 | 2 | 1 | 1 |
| 76 | Endrin | 72-20-8 | 2.70E+00 | 0 | 0 | -1 | 1 |
| 77 | Heptachlor | 76-44-8 | 1.70E+00 | 1 | 1 | 1 | 1 |
| 78 | Lindane | 58-89-9 | 7.60E+00 | 1 | 1 | 1 | 1 |
| 79 | Toxaphene | 8001-35-2 | 1.00E+01 | 1 | 1 | 1 | 1 |
| 80 | 2,4-D | 94-75-7 | 1.20E+03 | 0 | 1 | 1 | 1 |
| 92 | Total PCB | 1336-36-3 | 1.00E+00 | 0 | 0 | 1 | 1 |
| 94 | Total PCDD/PCDF | 1746-01-6 | 6.30E-05 | 0 | 0 | -1 | -1 |
| 95 | Methyl ethyl ketone | 78-93-3 | 8.21E+04 | 1 | 2 | 1 | 1 |
| 96 | Methyl isobutyl ketone | 108-10-1 | 1.10E+04 | 1 | 1 | 1 | 1 |
| 97 | Dibutyl phthalate | 84-74-2 | 1.07E+04 | 0 | 1 | 1 | 1 |
| 98 | DEHP | 117-81-7 | 3.00E+02 | 1 | 1 | 1 | 1 |
| 99 | Hexachlorobutadine | 87-68-3 | 1.80E+01 | 1 | 1 | 1 | 1 |
| 100 | Methyl tert-butyl ether | 1634-04-4 | 6.20E+03 | 2 | 2 | 1 | 1 |
|  |  | Overall | C1 | C2 | C3 | C4 | C5 |
|  |  | Scores | 82 | 1.48 | 1.52 | 74 | 74 |
|  |  |  |  |  | No. >0 | 78 | 78 |
|  |  |  |  |  | No. <0 | 4 | 4 |

**Table 3Sb** Alaska Dept. of Environmental Conservation < 40in.

| Number | Pollutant | CAS. No | RGV (mg/kg) | C_2_ | C_3_ | C_4_ | C_5_ |
| --- | --- | --- | --- | --- | --- | --- | --- |
| 1 | Antimony | 7440-36-0 | 4.10E+01 | 1 | 1 | 1 | 1 |
| 2 | Arsenic | 7440-38-2 | 4.50E+00 | 0 | 0 | -1 | -1 |
| 3 | Barium | 7440-39-3 | 2.03E+04 | 2 | 2 | 1 | 1 |
| 4 | Beryllium | 7440-41-7 | 2.00E+02 | 1 | 1 | 1 | 1 |
| 5 | Cadmium | 7440-43-9 | 7.90E+01 | 1 | 1 | 1 | 1 |
| 6 | Chromium (III) | 16065-83-1 | 1.52E+05 | 3 | 2 | 1 | 1 |
| 7 | Chromium (VI) | 18540-29-9 | 3.00E+02 | 1 | 1 | 1 | 1 |
| 9 | Copper | 7440-50-8 | 4.10E+03 | 2 | 1 | 1 | 1 |
| 10 | Lead | 7439-92-1 | 4.00E+02 | 0 | 1 | 1 | 1 |
| 12 | Mercury | 7439-97-6 | 3.00E+01 | 1 | 1 | 1 | 1 |
| 14 | Nickel | 7440-02-0 | 2.00E+03 | 1 | 1 | 1 | 1 |
| 15 | Selenium | 7782-49-2 | 5.10E+02 | 1 | 2 | 1 | 1 |
| 16 | Silver | 7440-22-4 | 5.10E+02 | 1 | 1 | 1 | 1 |
| 17 | Thallium | 7440-28-0 | 8.10E+00 | 1 | 1 | 1 | 1 |
| 19 | Vanadium | 7440-62-2 | 7.10E+02 | 1 | 1 | 1 | 1 |
| 20 | Zinc | 7440-66-6 | 3.04E+04 | 2 | 2 | 1 | 1 |
| 21 | Cyanide | 57-12-5 | 2.00E+03 | 2 | 2 | 1 | 1 |
| 23 | Dichloromethane | 75-09-2 | 1.10E+03 | 2 | 2 | 1 | 1 |
| 24 | Trichloromethane | 67-66-3 | 1.00E+03 | 3 | 3 | 1 | 1 |
| 25 | Tetrachloromethane | 56-23-5 | 6.40E+01 | 2 | 2 | 1 | 1 |
| 26 | Bromoform | 75-25-2 | 1.10E+03 | 1 | 2 | 1 | 1 |
| 27 | Bromomethane | 74-83-9 | 1.40E+02 | 2 | 2 | 1 | 1 |
| 28 | 1,2-Dichloroethane | 107-06-2 | 9.10E+01 | 2 | 2 | 1 | 1 |
| 29 | 1,1,1-Trichloroethane | 71-55-6 | 2.03E+04 | 2 | 2 | 1 | 1 |
| 30 | 1,1,2-Trichloroethane | 79-00-5 | 1.50E+02 | 2 | 2 | 1 | 1 |
| 31 | Chloroethene | 75-01-4 | 5.50E+00 | 2 | 2 | 1 | 1 |
| 32 | 1,1-Dichloroethene | 75-35-4 | 1.40E+01 | 0 | 0 | 1 | 1 |
| 33 | Trichloroethene | 79-01-6 | 2.10E+01 | 1 | 1 | 1 | 1 |
| 34 | Tetrachloroethene | 127-18-4 | 1.50E+01 | 1 | 1 | 1 | 1 |
| 35 | Benzene | 71-43-2 | 1.50E+02 | 3 | 2 | 1 | 1 |
| 36 | Toluene | 108-88-3 | 8.10E+03 | 2 | 2 | 1 | 1 |
| 37 | Chlorobenzene | 108-90-7 | 2.00E+03 | 2 | 2 | 1 | 1 |
| 38 | 1,2-Dichlorobenzene | 95-50-1 | 9.10E+03 | 2 | 2 | 1 | 1 |
| 39 | 1,3-Dichlorobenzene | 541-73-1 | 9.10E+03 | 3 | 3 | 1 | 1 |
| 40 | 1,4-Dichlorobenzene | 106-46-7 | 3.50E+02 | 2 | 2 | 1 | 1 |
| 41 | 1,2,4Trichlorobenzene | 120-82-1 | 1.00E+03 | 2 | 2 | 1 | 1 |
| 42 | Hexachlorobenzene | 118-74-1 | 3.20E+00 | 1 | 1 | 1 | 1 |
| 43 | Nitrobenzene | 98-95-3 | 5.10E+01 | 1 | 1 | 1 | 1 |
| 44 | Ethylbenzene | 100-41-4 | 1.01E+04 | 3 | 3 | 1 | 1 |
| 45 | Styrene | 100-42-5 | 2.03E+04 | 3 | 3 | 1 | 1 |
| 46 | Xylenes | 1330-20-7 | 2.03E+04 | 2 | 2 | 1 | 1 |
| 47 | Cresol | 1319-77-3 | 3.50E+02 | 1 | 1 | 1 | 1 |
| 48 | Phenol | 108-95-2 | 2.32E+04 | 2 | 2 | 1 | 1 |
| 49 | 2-Chlorophenol | 95-57-8 | 5.10E+02 | 1 | 2 | 1 | 1 |
| 50 | 2,4-Dichlorophenol | 120-83-2 | 2.30E+02 | 1 | 2 | 1 | 1 |
| 51 | 2,4,5-Trichlorophenol | 95-95-4 | 6.50E+03 | 1 | 2 | 1 | 1 |
| 52 | 2,4,6-Trichlorophenol | 88-06-2 | 4.60E+02 | 2 | 2 | 1 | 1 |
| 54 | Pentachlorophenol | 87-86-5 | 3.90E+01 | 1 | 1 | 1 | 1 |
| 55 | Benz(a)anthracene | 56-55-3 | 4.90E+00 | 1 | 0 | 1 | 1 |
| 56 | Benzo(a)pyrene | 50-32-8 | 4.90E-01 | 0 | 0 | -1 | -1 |
| 57 | Benzo(b)fluoranthene | 205-99-2 | 4.90E+00 | 1 | 1 | 1 | 1 |
| 58 | Benzo(k)fluoranthene | 207-08-9 | 4.90E+01 | 1 | 1 | 1 | 1 |
| 59 | Chrysene | 218-01-9 | 4.90E+02 | 1 | 1 | 1 | 1 |
| 60 | Dibena(a,h)anthracene | 53-70-3 | 4.90E-01 | 0 | 0 | -1 | -1 |
| 61 | Indeno(1,2,3-c,d)pyrebe | 193-39-5 | 4.90E+00 | 0 | 0 | 1 | 1 |
| 62 | Naphthalene | 91-20-3 | 1.40E+03 | 2 | 3 | 1 | 1 |
| 63 | Acenaphehene | 83-32-9 | 2.80E+03 | 2 | 2 | 1 | 1 |
| 64 | Acenaphthylene | 208-96-8 | 2.80E+03 | 2 | 2 | 1 | 1 |
| 65 | Anthracene | 120-12-7 | 2.06E+04 | 3 | 2 | 1 | 1 |
| 66 | Benzo(g,h,i) perylene | 191-24-2 | 1.40E+03 | 2 | 2 | 1 | 1 |
| 67 | Flouranthene | 206-44-0 | 1.90E+03 | 3 | 1 | 1 | 1 |
| 68 | Flouorene | 86-73-7 | 2.30E+03 | 1 | 1 | 1 | 1 |
| 69 | Phenanthrene | 85-01-8 | 2.06E+04 | 3 | 3 | 1 | 1 |
| 70 | Pyrene | 129-00-0 | 1.40E+03 | 1 | 1 | 1 | 1 |
| 71 | Aldrin | 309-00-2 | 3.00E-01 | 1 | 0 | 1 | 1 |
| 72 | Chlordane | 57-74-9 | 1.90E+01 | 1 | 1 | 1 | 1 |
| 73 | DDT | 50-29-3 | 2.10E+01 | 1 | 1 | 1 | 1 |
| 74 | Dieldrin | 60-57-1 | 3.20E-01 | 1 | 0 | 1 | 1 |
| 75 | Endosulfan | 115-29-7 | 6.10E+02 | 1 | 2 | 1 | 1 |
| 76 | Endrin | 72-20-8 | 2.00E+00 | 0 | 0 | -1 | -1 |
| 77 | Heptachlor | 76-44-8 | 1.30E+00 | 1 | 1 | 1 | 1 |
| 78 | Lindane | 58-89-9 | 5.60E+00 | 1 | 1 | 1 | 1 |
| 79 | Toxaphene | 8001-35-2 | 7.50E+00 | 1 | 1 | 1 | 1 |
| 80 | 2,4-D | 94-75-7 | 8.60E+02 | 0 | 1 | 1 | 1 |
| 92 | Total PCB | 1336-36-3 | 1.00E+00 | 0 | 0 | 1 | 1 |
| 94 | Total PCDD/PCDF | 1746-01-6 | 4.70E-05 | 0 | 0 | -1 | -1 |
| 95 | Methyl ethyl ketone | 78-93-3 | 6.08E+04 | 1 | 2 | 1 | 1 |
| 96 | Methyl isobutyl ketone | 108-10-1 | 8.10E+03 | 1 | 1 | 1 | 1 |
| 97 | Dibutyl phthalate | 84-74-2 | 7.90E+03 | 0 | 1 | 1 | 1 |
| 98 | DEHP | 117-81-7 | 2.20E+02 | 1 | 0 | 1 | 1 |
| 99 | Hexachlorobutadine | 87-68-3 | 1.30E+01 | 1 | 1 | 1 | 1 |
| 100 | Methyl tert-butyl ether | 1634-04-4 | 4.60E+03 | 2 | 2 | 1 | 1 |
|  |  | Overall | C1 | C2 | C3 | C4 | C5 |
|  |  | Scores | 82 | 1.38 | 1.39 | 72 | 72 |
|  |  |  |  |  | No. >0 | 77 | 77 |
|  |  |  |  |  | No. <0 | 5 | 5 |

**Table S3c** Alaska Dept. of Environmental Conservation >40in.

| Number | Pollutant | CAS. No | RGV (mg/kg) | C_2_ | C_3_ | C_4_ | C_5_ |
| --- | --- | --- | --- | --- | --- | --- | --- |
| 1 | Antimony | 7440-36-0 | 3.30E+01 | 0 | 1 | 1 | 1 |
| 2 | Arsenic | 7440-38-2 | 3.70E+00 | 0 | 0 | -1 | -1 |
| 3 | Barium | 7440-39-3 | 1.66E+04 | 2 | 1 | 1 | 1 |
| 4 | Beryllium | 7440-41-7 | 1.70E+02 | 1 | 1 | 1 | 1 |
| 5 | Cadmium | 7440-43-9 | 6.50E+01 | 1 | 1 | 1 | 1 |
| 6 | Chromium (III) | 16065-83-1 | 1.24E+05 | 3 | 2 | 1 | 1 |
| 7 | Chromium (VI) | 18540-29-9 | 2.50E+02 | 1 | 1 | 1 | 1 |
| 9 | Copper | 7440-50-8 | 3.30E+03 | 1 | 1 | 1 | 1 |
| 10 | Lead | 7439-92-1 | 4.00E+02 | 0 | 1 | 1 | 1 |
| 12 | Mercury | 7439-97-6 | 2.50E+01 | 1 | 1 | 1 | 1 |
| 14 | Nickel | 7440-02-0 | 1.70E+03 | 1 | 1 | 1 | 1 |
| 15 | Selenium | 7782-49-2 | 4.10E+02 | 1 | 1 | 1 | 1 |
| 16 | Silver | 7440-22-4 | 4.10E+02 | 1 | 1 | 1 | 1 |
| 17 | Thallium | 7440-28-0 | 6.60E+00 | 1 | 1 | 1 | 1 |
| 19 | Vanadium | 7440-62-2 | 5.80E+02 | 1 | 1 | 1 | 1 |
| 20 | Zinc | 7440-66-6 | 2.49E+04 | 2 | 2 | 1 | 1 |
| 21 | Cyanide | 57-12-5 | 1.70E+03 | 2 | 2 | 1 | 1 |
| 23 | Dichloromethane | 75-09-2 | 9.10E+02 | 2 | 2 | 1 | 1 |
| 24 | Trichloromethane | 67-66-3 | 8.30E+02 | 3 | 3 | 1 | 1 |
| 25 | Tetrachloromethane | 56-23-5 | 5.20E+01 | 2 | 2 | 1 | 1 |
| 26 | Bromoform | 75-25-2 | 8.60E+02 | 1 | 1 | 1 | 1 |
| 27 | Bromomethane | 74-83-9 | 1.20E+02 | 1 | 1 | 1 | 1 |
| 28 | 1,2-Dichloroethane | 107-06-2 | 7.50E+01 | 2 | 2 | 1 | 1 |
| 29 | 1,1,1-Trichloroethane | 71-55-6 | 1.66E+04 | 2 | 2 | 1 | 1 |
| 30 | 1,1,2-Trichloroethane | 79-00-5 | 1.20E+02 | 2 | 2 | 1 | 1 |
| 31 | Chloroethene | 75-01-4 | 4.50E+00 | 2 | 1 | 1 | 1 |
| 32 | 1,1-Dichloroethene | 75-35-4 | 1.10E+01 | 0 | 0 | -1 | 1 |
| 33 | Trichloroethene | 79-01-6 | 1.70E+01 | 1 | 1 | 1 | 1 |
| 34 | Tetrachloroethene | 127-18-4 | 1.30E+01 | 1 | 1 | 1 | 1 |
| 35 | Benzene | 71-43-2 | 1.20E+02 | 2 | 2 | 1 | 1 |
| 36 | Toluene | 108-88-3 | 6.60E+03 | 2 | 2 | 1 | 1 |
| 37 | Chlorobenzene | 108-90-7 | 1.70E+03 | 2 | 2 | 1 | 1 |
| 38 | 1,2-Dichlorobenzene | 95-50-1 | 7.50E+03 | 2 | 2 | 1 | 1 |
| 39 | 1,3-Dichlorobenzene | 541-73-1 | 7.50E+03 | 3 | 3 | 1 | 1 |
| 40 | 1,4-Dichlorobenzene | 106-46-7 | 2.80E+02 | 2 | 2 | 1 | 1 |
| 41 | 1,2,4Trichlorobenzene | 120-82-1 | 8.30E+02 | 2 | 2 | 1 | 1 |
| 42 | Hexachlorobenzene | 118-74-1 | 2.60E+00 | 1 | 1 | 1 | 1 |
| 43 | Nitrobenzene | 98-95-3 | 4.10E+01 | 1 | 1 | 1 | 1 |
| 44 | Ethylbenzene | 100-41-4 | 8.30E+03 | 3 | 3 | 1 | 1 |
| 45 | Styrene | 100-42-5 | 1.66E+04 | 3 | 3 | 1 | 1 |
| 46 | Xylenes | 1330-20-7 | 1.66E+04 | 2 | 2 | 1 | 1 |
| 47 | Cresol | 1319-77-3 | 2.90E+02 | 0 | 1 | 1 | 1 |
| 48 | Phenol | 108-95-2 | 1.90E+04 | 2 | 2 | 1 | 1 |
| 49 | 2-Chlorophenol | 95-57-8 | 4.10E+02 | 1 | 2 | 1 | 1 |
| 50 | 2,4-Dichlorophenol | 120-83-2 | 1.90E+02 | 1 | 1 | 1 | 1 |
| 51 | 2,4,5-Trichlorophenol | 95-95-4 | 5.30E+03 | 1 | 2 | 1 | 1 |
| 52 | 2,4,6-Trichlorophenol | 88-06-2 | 3.80E+02 | 2 | 2 | 1 | 1 |
| 54 | Pentachlorophenol | 87-86-5 | 3.20E+01 | 1 | 1 | 1 | 1 |
| 55 | Benz(a)anthracene | 56-55-3 | 4.00E+00 | 1 | 0 | 1 | 1 |
| 56 | Benzo(a)pyrene | 50-32-8 | 4.00E-01 | 0 | 0 | -1 | -1 |
| 57 | Benzo(b)fluoranthene | 205-99-2 | 4.00E+00 | 1 | 0 | 1 | 1 |
| 58 | Benzo(k)fluoranthene | 207-08-9 | 4.00E+01 | 1 | 1 | 1 | 1 |
| 59 | Chrysene | 218-01-9 | 4.00E+02 | 1 | 1 | 1 | 1 |
| 60 | Dibena(a,h)anthracene | 53-70-3 | 4.00E-01 | 0 | 0 | -1 | -1 |
| 61 | Indeno(1,2,3-c,d)pyrebe | 193-39-5 | 4.00E+00 | 0 | 0 | 1 | 1 |
| 62 | Naphthalene | 91-20-3 | 1.10E+03 | 2 | 3 | 1 | 1 |
| 63 | Acenaphehene | 83-32-9 | 2.30E+03 | 2 | 1 | 1 | 1 |
| 64 | Acenaphthylene | 208-96-8 | 2.30E+03 | 2 | 2 | 1 | 1 |
| 65 | Anthracene | 120-12-7 | 1.68E+04 | 2 | 2 | 1 | 1 |
| 66 | Benzo(g,h,i) perylene | 191-24-2 | 1.10E+03 | 2 | 1 | 1 | 1 |
| 67 | Flouranthene | 206-44-0 | 1.50E+03 | 2 | 1 | 1 | 1 |
| 68 | Flouorene | 86-73-7 | 1.90E+03 | 1 | 1 | 1 | 1 |
| 69 | Phenanthrene | 85-01-8 | 1.68E+04 | 3 | 3 | 1 | 1 |
| 70 | Pyrene | 129-00-0 | 1.10E+03 | 1 | 1 | 1 | 1 |
| 71 | Aldrin | 309-00-2 | 2.40E-01 | 1 | 0 | 1 | -1 |
| 72 | Chlordane | 57-74-9 | 1.50E+01 | 1 | 1 | 1 | 1 |
| 73 | DDT | 50-29-3 | 1.80E+01 | 1 | 1 | 1 | 1 |
| 74 | Dieldrin | 60-57-1 | 2.60E-01 | 0 | 0 | 1 | -1 |
| 75 | Endosulfan | 115-29-7 | 5.00E+02 | 1 | 2 | 1 | 1 |
| 76 | Endrin | 72-20-8 | 1.70E+00 | 0 | 0 | -1 | -1 |
| 77 | Heptachlor | 76-44-8 | 1.00E+00 | 1 | 1 | 1 | 1 |
| 78 | Lindane | 58-89-9 | 4.60E+00 | 1 | 1 | 1 | 1 |
| 79 | Toxaphene | 8001-35-2 | 6.20E+00 | 1 | 1 | 1 | 1 |
| 80 | 2,4-D | 94-75-7 | 7.10E+02 | 0 | 1 | 1 | 1 |
| 92 | Total PCB | 1336-36-3 | 1.00E+00 | 0 | 0 | 1 | 1 |
| 94 | Total PCDD/PCDF | 1746-01-6 | 3.80E-05 | 0 | 0 | -1 | -1 |
| 95 | Methyl ethyl ketone | 78-93-3 | 4.98E+04 | 1 | 2 | 1 | 1 |
| 96 | Methyl isobutyl ketone | 108-10-1 | 6.60E+03 | 1 | 1 | 1 | 1 |
| 97 | Dibutyl phthalate | 84-74-2 | 6.50E+03 | 0 | 1 | 1 | 1 |
| 98 | DEHP | 117-81-7 | 1.80E+02 | 1 | 0 | 1 | 1 |
| 99 | Hexachlorobutadine | 87-68-3 | 1.10E+01 | 0 | 1 | 1 | 1 |
| 100 | Methyl tert-butyl ether | 1634-04-4 | 3.80E+03 | 2 | 2 | 1 | 1 |
|  |  | Overall | C1 | C2 | C3 | C4 | C5 |
|  |  | Scores | 82 | 1.27 | 1.28 | 70 | 68 |
|  |  |  |  |  | No. >0 | 76 | 75 |
|  |  |  |  |  | No. <0 | 6 | 7 |

**Table S4a** Arizona Administrative Code Appendix A-1E-06 non-carcinogen

| Number | Pollutant | CAS. No | RGV (mg/kg) | C_2_ | C_3_ | C_4_ | C_5_ |
| --- | --- | --- | --- | --- | --- | --- | --- |
| 1 | Antimony | 7440-36-0 | 3.10E+01 | 0 | 1 | 1 | 1 |
| 2 | Arsenic | 7440-38-2 | 1.00E+01 | 0 | 0 | -1 | -1 |
| 3 | Barium | 7440-39-3 | 1.50E+04 | 1 | 1 | 1 | 1 |
| 4 | Beryllium | 7440-41-7 | 1.50E+02 | 1 | 1 | 1 | 1 |
| 5 | Cadmium | 7440-43-9 | 3.90E+01 | 1 | 1 | 1 | 1 |
| 6 | Chromium (III) | 16065-83-1 | 1.20E+05 | 3 | 2 | 1 | 1 |
| 7 | Chromium (VI) | 18540-29-9 | 3.00E+01 | 0 | 0 | -1 | -1 |
| 8 | Cobalt | 7440-48-4 | 9.00E+02 | 1 | 1 | 1 | 1 |
| 9 | Copper | 7440-50-8 | 3.10E+03 | 1 | 1 | 1 | 1 |
| 10 | Lead | 7439-92-1 | 4.00E+02 | 0 | 1 | 1 | 1 |
| 11 | Manganese | 7439-96-5 | 3.30E+03 | 1 | 1 | 1 | 1 |
| 12 | Mercury | 7439-97-6 | 2.30E+01 | 1 | 1 | 1 | 1 |
| 13 | Molybdenum | 7439-98-7 | 3.90E+02 | 1 | 1 | 1 | 1 |
| 14 | Nickel | 7440-02-0 | 1.60E+03 | 1 | 1 | 1 | 1 |
| 15 | Selenium | 7782-49-2 | 3.90E+02 | 1 | 1 | 1 | 1 |
| 16 | Silver | 7440-22-4 | 3.90E+02 | 1 | 1 | 1 | 1 |
| 17 | Thallium | 7440-28-0 | 5.20E+00 | 0 | 0 | 1 | 1 |
| 18 | Tin | 7440-31-5 | 4.70E+04 | 3 | 2 | 1 | 1 |
| 19 | Vanadium | 7440-62-2 | 7.80E+01 | 0 | 0 | -1 | -1 |
| 20 | Zinc | 7440-66-6 | 2.30E+04 | 2 | 2 | 1 | 1 |
| 21 | Cyanide | 57-12-5 | 1.20E+03 | 2 | 2 | 1 | 1 |
| 22 | Acrylonitril | 107-13-1 | 2.10E-01 | -1 | -1 | -1 | -1 |
| 23 | Dichloromethane | 75-09-2 | 9.30E+00 | 0 | 0 | -1 | -1 |
| 24 | Trichloromethane | 67-66-3 | 9.40E-01 | 0 | 0 | -1 | -1 |
| 25 | Tetrachloromethane | 56-23-5 | 2.50E-01 | 0 | 0 | -1 | -1 |
| 26 | Bromoform | 75-25-2 | 6.90E+01 | 0 | 0 | 1 | 1 |
| 27 | Bromomethane | 74-83-9 | 3.90E+00 | 0 | 0 | -1 | -1 |
| 28 | 1,2-Dichloroethane | 107-06-2 | 2.80E-01 | -1 | 0 | -1 | -1 |
| 29 | 1,1,1-Trichloroethane | 71-55-6 | 1.20E+03 | 1 | 1 | 1 | 1 |
| 30 | 1,1,2-Trichloroethane | 79-00-5 | 7.40E-01 | 0 | 0 | -1 | -1 |
| 31 | Chloroethene | 75-01-4 | 8.50E-02 | 0 | 0 | -1 | -1 |
| 32 | 1,1-Dichloroethene | 75-35-4 | 1.20E+02 | 1 | 1 | 1 | 1 |
| 33 | Trichloroethene | 79-01-6 | 3.00E+00 | 0 | 0 | -1 | -1 |
| 34 | Tetrachloroethene | 127-18-4 | 5.10E-01 | -1 | -1 | -1 | -1 |
| 35 | Benzene | 71-43-2 | 6.50E-01 | 0 | 0 | -1 | -1 |
| 36 | Toluene | 108-88-3 | 6.50E+02 | 1 | 1 | 1 | 1 |
| 37 | Chlorobenzene | 108-90-7 | 1.50E+02 | 1 | 1 | 1 | 1 |
| 38 | 1,2-Dichlorobenzene | 95-50-1 | 6.00E+02 | 1 | 1 | 1 | 1 |
| 39 | 1,3-Dichlorobenzene | 541-73-1 | 5.30E+02 | 1 | 2 | 1 | 1 |
| 40 | 1,4-Dichlorobenzene | 106-46-7 | 3.50E+00 | 0 | 0 | -1 | -1 |
| 41 | 1,2,4Trichlorobenzene | 120-82-1 | 6.20E+01 | 1 | 1 | 1 | 1 |
| 42 | Hexachlorobenzene | 118-74-1 | 3.40E-01 | 0 | 0 | -1 | -1 |
| 43 | Nitrobenzene | 98-95-3 | 2.00E+01 | 0 | 0 | 1 | 1 |
| 44 | Ethylbenzene | 100-41-4 | 4.00E+02 | 1 | 1 | 1 | 1 |
| 45 | Styrene | 100-42-5 | 1.50E+03 | 1 | 1 | 1 | 1 |
| 46 | Xylenes | 1330-20-7 | 2.70E+02 | 0 | 1 | 1 | 1 |
| 47 | Cresol | 1319-77-3 | 3.10E+02 | 0 | 1 | 1 | 1 |
| 48 | Phenol | 108-95-2 | 1.80E+04 | 2 | 2 | 1 | 1 |
| 49 | 2-Chlorophenol | 95-57-8 | 6.30E+01 | 0 | 1 | 1 | 1 |
| 50 | 2,4-Dichlorophenol | 120-83-2 | 1.80E+02 | 1 | 1 | 1 | 1 |
| 51 | 2,4,5-Trichlorophenol | 95-95-4 | 6.10E+03 | 1 | 2 | 1 | 1 |
| 52 | 2,4,6-Trichlorophenol | 88-06-2 | 6.10E+00 | 0 | 0 | -1 | -1 |
| 53 | 2,3,4,6-Tetrahlorophenol | 58-90-2 | 1.80E+03 | 1 | 2 | 1 | 1 |
| 54 | Pentachlorophenol | 87-86-5 | 3.20E+00 | 0 | 0 | -1 | -1 |
| 55 | Benz(a)anthracene | 56-55-3 | 6.90E-01 | 0 | 0 | -1 | -1 |
| 56 | Benzo(a)pyrene | 50-32-8 | 6.90E-02 | -1 | -1 | -1 | -1 |
| 57 | Benzo(b)fluoranthene | 205-99-2 | 6.90E-01 | 0 | 0 | -1 | -1 |
| 58 | Benzo(k)fluoranthene | 207-08-9 | 6.90E+00 | 0 | 0 | -1 | -1 |
| 59 | Chrysene | 218-01-9 | 6.80E+01 | 1 | 1 | 1 | 1 |
| 60 | Dibena(a,h)anthracene | 53-70-3 | 6.90E-02 | -1 | -1 | -1 | -1 |
| 61 | Indeno(1,2,3-c,d)pyrebe | 193-39-5 | 6.90E-01 | 0 | 0 | -1 | -1 |
| 62 | Naphthalene | 91-20-3 | 5.60E+01 | 1 | 1 | 1 | 1 |
| 63 | Acenaphehene | 83-32-9 | 3.70E+03 | 2 | 2 | 1 | 1 |
| 65 | Anthracene | 120-12-7 | 2.20E+04 | 3 | 2 | 1 | 1 |
| 67 | Flouranthene | 206-44-0 | 2.30E+03 | 3 | 2 | 1 | 1 |
| 68 | Flouorene | 86-73-7 | 2.70E+03 | 2 | 2 | 1 | 1 |
| 70 | Pyrene | 129-00-0 | 2.30E+03 | 1 | 1 | 1 | 1 |
| 71 | Aldrin | 309-00-2 | 3.20E-02 | 0 | -1 | -1 | -1 |
| 72 | Chlordane | 57-74-9 | 1.90E+00 | 0 | 0 | -1 | -1 |
| 73 | DDT | 50-29-3 | 2.00E+00 | 0 | 0 | -1 | -1 |
| 74 | Dieldrin | 60-57-1 | 3.40E-02 | 0 | -1 | -1 | -1 |
| 75 | Endosulfan | 115-29-7 | 3.70E+02 | 1 | 2 | 1 | 1 |
| 76 | Endrin | 72-20-8 | 1.80E+01 | 1 | 1 | 1 | 1 |
| 77 | Heptachlor | 76-44-8 | 1.20E-01 | 0 | 0 | -1 | -1 |
| 78 | Lindane | 58-89-9 | 5.00E-01 | 0 | 0 | -1 | -1 |
| 79 | Toxaphene | 8001-35-2 | 5.00E-01 | 0 | 0 | -1 | -1 |
| 80 | 2,4-D | 94-75-7 | 6.90E+02 | 0 | 1 | -1 | 1 |
| 81 | Atrazine | 1912-24-9 | 2.50E+00 | 0 | 0 | -1 | -1 |
| 82 | Carbaryl | 63-25-2 | 6.10E+03 | 0 | 2 | 1 | 1 |
| 83 | Carbofuran | 1563-66-2 | 3.10E+02 | 0 | 1 | 1 | 1 |
| 84 | Chloryrifos | 2921-88-2 | 1.80E+02 | 0 | 1 | 1 | 1 |
| 85 | Diuron | 330-54-1 | 1.20E+02 | 0 | 1 | -1 | 1 |
| 86 | Gylphosate | 1071-83-6 | 6.10E+03 | 0 | 1 | -1 | 1 |
| 87 | Malathion | 121-75-5 | 1.20E+03 | 0 | 1 | -1 | 1 |
| 88 | MCPA | 94-74-6 | 3.10E+01 | 0 | 0 | -1 | 1 |
| 89 | Picloram | 1918-02-1 | 4.30E+03 | 0 | 1 | -1 | 1 |
| 90 | Simazine | 122-34-9 | 4.60E+00 | 0 | 0 | 1 | -1 |
| 91 | Trifluralin | 1582-09-8 | 7.10E+01 | 0 | 0 | -1 | -1 |
| 92 | Total PCB | 1336-36-3 | 1.10E+00 | 0 | 0 | 1 | 1 |
| 94 | Total PCDD/PCDF | 1746-01-6 | 4.50E-06 | -1 | -1 | -1 | -1 |
| 95 | Methyl ethyl ketone | 78-93-3 | 2.30E+04 | 1 | 1 | 1 | 1 |
| 96 | Methyl isobutyl ketone | 108-10-1 | 5.30E+03 | 0 | 1 | 1 | 1 |
| 97 | Dibutyl phthalate | 84-74-2 | 6.10E+03 | 0 | 1 | -1 | 1 |
| 98 | DEHP | 117-81-7 | 3.90E+01 | 0 | 0 | -1 | -1 |
| 99 | Hexachlorobutadine | 87-68-3 | 7.00E+00 | 0 | 0 | 1 | 1 |
| 100 | Methyl tert-butyl ether | 1634-04-4 | 3.20E+01 | 0 | 0 | -1 | -1 |
|  |  | Overall | C1 | C2 | C3 | C4 | C5 |
|  |  | Scores | 96 | 0.47 | 0.63 | 10 | 22 |
|  |  |  |  |  | No. >0 | 53 | 59 |
|  |  |  |  |  | No. <0 | 43 | 37 |

**Table S4b** Arizona Administrative Code Appendix A-1E-05 non-carcinogen

| Number | Pollutant | CAS. No | RGV (mg/kg) | C_2_ | C_3_ | C_4_ | C_5_ |
| --- | --- | --- | --- | --- | --- | --- | --- |
| 1 | Antimony | 7440-36-0 | 3.10E+01 | 0 | 1 | 1 | 1 |
| 2 | Arsenic | 7440-38-2 | 1.00E+01 | 0 | 0 | -1 | -1 |
| 3 | Barium | 7440-39-3 | 1.50E+04 | 1 | 1 | 1 | 1 |
| 4 | Beryllium | 7440-41-7 | 1.50E+02 | 1 | 1 | 1 | 1 |
| 5 | Cadmium | 7440-43-9 | 3.90E+01 | 1 | 1 | 1 | 1 |
| 6 | Chromium (III) | 16065-83-1 | 1.20E+05 | 3 | 2 | 1 | 1 |
| 8 | Cobalt | 7440-48-4 | 1.40E+03 | 2 | 2 | 1 | 1 |
| 9 | Copper | 7440-50-8 | 3.10E+03 | 1 | 1 | 1 | 1 |
| 10 | Lead | 7439-92-1 | 4.00E+02 | 0 | 1 | 1 | 1 |
| 11 | Manganese | 7439-96-5 | 3.30E+03 | 1 | 1 | 1 | 1 |
| 12 | Mercury | 7439-97-6 | 2.30E+01 | 1 | 1 | 1 | 1 |
| 13 | Molybdenum | 7439-98-7 | 3.90E+02 | 1 | 1 | 1 | 1 |
| 14 | Nickel | 7440-02-0 | 1.60E+03 | 1 | 1 | 1 | 1 |
| 15 | Selenium | 7782-49-2 | 3.90E+02 | 1 | 1 | 1 | 1 |
| 16 | Silver | 7440-22-4 | 3.90E+02 | 1 | 1 | 1 | 1 |
| 17 | Thallium | 7440-28-0 | 5.20E+00 | 0 | 0 | 1 | 1 |
| 18 | Tin | 7440-31-5 | 4.70E+04 | 3 | 2 | 1 | 1 |
| 19 | Vanadium | 7440-62-2 | 7.80E+01 | 0 | 0 | -1 | -1 |
| 20 | Zinc | 7440-66-6 | 2.30E+04 | 2 | 2 | 1 | 1 |
| 21 | Cyanide | 57-12-5 | 1.20E+03 | 2 | 2 | 1 | 1 |
| 22 | Acrylonitril | 107-13-1 | 2.10E+00 | 0 | 0 | -1 | 1 |
| 23 | Dichloromethane | 75-09-2 | 9.30E+01 | 1 | 1 | 1 | 1 |
| 24 | Trichloromethane | 67-66-3 | 9.40E+00 | 1 | 1 | 1 | 1 |
| 25 | Tetrachloromethane | 56-23-5 | 2.20E+00 | 1 | 1 | 1 | 1 |
| 26 | Bromoform | 75-25-2 | 6.90E+02 | 1 | 1 | 1 | 1 |
| 27 | Bromomethane | 74-83-9 | 3.90E+00 | 0 | 0 | -1 | -1 |
| 28 | 1,2-Dichloroethane | 107-06-2 | 2.80E+00 | 0 | 1 | 1 | 1 |
| 29 | 1,1,1-Trichloroethane | 71-55-6 | 1.20E+03 | 1 | 1 | 1 | 1 |
| 30 | 1,1,2-Trichloroethane | 79-00-5 | 7.40E+00 | 1 | 1 | 1 | 1 |
| 32 | 1,1-Dichloroethene | 75-35-4 | 1.20E+02 | 1 | 1 | 1 | 1 |
| 33 | Trichloroethene | 79-01-6 | 1.70E+01 | 1 | 1 | 1 | 1 |
| 34 | Tetrachloroethene | 127-18-4 | 5.10E+00 | 0 | 0 | 1 | 1 |
| 36 | Toluene | 108-88-3 | 6.50E+02 | 1 | 1 | 1 | 1 |
| 37 | Chlorobenzene | 108-90-7 | 1.50E+02 | 1 | 1 | 1 | 1 |
| 38 | 1,2-Dichlorobenzene | 95-50-1 | 6.00E+02 | 1 | 1 | 1 | 1 |
| 39 | 1,3-Dichlorobenzene | 541-73-1 | 5.30E+02 | 1 | 2 | 1 | 1 |
| 40 | 1,4-Dichlorobenzene | 106-46-7 | 3.50E+01 | 1 | 1 | 1 | 1 |
| 41 | 1,2,4Trichlorobenzene | 120-82-1 | 6.20E+01 | 1 | 1 | 1 | 1 |
| 42 | Hexachlorobenzene | 118-74-1 | 3.40E+00 | 1 | 1 | 1 | 1 |
| 43 | Nitrobenzene | 98-95-3 | 2.00E+01 | 0 | 0 | 1 | 1 |
| 44 | Ethylbenzene | 100-41-4 | 4.00E+02 | 1 | 1 | 1 | 1 |
| 45 | Styrene | 100-42-5 | 1.50E+03 | 1 | 1 | 1 | 1 |
| 46 | Xylenes | 1330-20-7 | 2.70E+02 | 0 | 1 | 1 | 1 |
| 47 | Cresol | 1319-77-3 | 3.10E+02 | 0 | 1 | 1 | 1 |
| 48 | Phenol | 108-95-2 | 1.80E+04 | 2 | 2 | 1 | 1 |
| 49 | 2-Chlorophenol | 95-57-8 | 6.30E+01 | 0 | 1 | 1 | 1 |
| 50 | 2,4-Dichlorophenol | 120-83-2 | 1.80E+02 | 1 | 1 | 1 | 1 |
| 51 | 2,4,5-Trichlorophenol | 95-95-4 | 6.10E+03 | 1 | 2 | 1 | 1 |
| 52 | 2,4,6-Trichlorophenol | 88-06-2 | 6.10E+00 | 0 | 0 | -1 | -1 |
| 53 | 2,3,4,6-Tetrahlorophenol | 58-90-2 | 1.80E+03 | 1 | 2 | 1 | 1 |
| 54 | Pentachlorophenol | 87-86-5 | 3.20E+01 | 1 | 1 | 1 | 1 |
| 55 | Benz(a)anthracene | 56-55-3 | 6.90E+00 | 1 | 1 | 1 | 1 |
| 56 | Benzo(a)pyrene | 50-32-8 | 6.90E-01 | 0 | 0 | -1 | 1 |
| 57 | Benzo(b)fluoranthene | 205-99-2 | 6.90E+00 | 1 | 1 | 1 | 1 |
| 58 | Benzo(k)fluoranthene | 207-08-9 | 6.90E+01 | 1 | 1 | 1 | 1 |
| 59 | Chrysene | 218-01-9 | 6.80E+02 | 2 | 2 | 1 | 1 |
| 60 | Dibena(a,h)anthracene | 53-70-3 | 6.90E-01 | 0 | 0 | 1 | -1 |
| 61 | Indeno(1,2,3-c,d)pyrebe | 193-39-5 | 6.90E+00 | 1 | 1 | 1 | 1 |
| 62 | Naphthalene | 91-20-3 | 5.60E+01 | 1 | 1 | 1 | 1 |
| 63 | Acenaphehene | 83-32-9 | 3.70E+03 | 2 | 2 | 1 | 1 |
| 65 | Anthracene | 120-12-7 | 2.20E+04 | 3 | 2 | 1 | 1 |
| 67 | Flouranthene | 206-44-0 | 2.30E+03 | 3 | 2 | 1 | 1 |
| 68 | Flouorene | 86-73-7 | 2.70E+03 | 2 | 2 | 1 | 1 |
| 70 | Pyrene | 129-00-0 | 2.30E+03 | 1 | 1 | 1 | 1 |
| 71 | Aldrin | 309-00-2 | 3.20E-01 | 1 | 0 | 1 | 1 |
| 72 | Chlordane | 57-74-9 | 1.90E+01 | 1 | 1 | 1 | 1 |
| 73 | DDT | 50-29-3 | 2.00E+01 | 1 | 1 | 1 | 1 |
| 74 | Dieldrin | 60-57-1 | 3.40E-01 | 1 | 0 | 1 | 1 |
| 75 | Endosulfan | 115-29-7 | 3.70E+02 | 1 | 2 | 1 | 1 |
| 76 | Endrin | 72-20-8 | 1.80E+01 | 1 | 1 | 1 | 1 |
| 77 | Heptachlor | 76-44-8 | 1.20E+00 | 1 | 1 | 1 | 1 |
| 78 | Lindane | 58-89-9 | 5.00E+00 | 1 | 1 | 1 | 1 |
| 79 | Toxaphene | 8001-35-2 | 5.00E+00 | 1 | 1 | 1 | 1 |
| 80 | 2,4-D | 94-75-7 | 6.90E+02 | 0 | 1 | -1 | 1 |
| 81 | Atrazine | 1912-24-9 | 2.50E+01 | 1 | 1 | 1 | 1 |
| 82 | Carbaryl | 63-25-2 | 6.10E+03 | 0 | 2 | 1 | 1 |
| 83 | Carbofuran | 1563-66-2 | 3.10E+02 | 0 | 1 | 1 | 1 |
| 84 | Chloryrifos | 2921-88-2 | 1.80E+02 | 0 | 1 | 1 | 1 |
| 85 | Diuron | 330-54-1 | 1.20E+02 | 0 | 1 | -1 | 1 |
| 86 | Gylphosate | 1071-83-6 | 6.10E+03 | 0 | 1 | -1 | 1 |
| 87 | Malathion | 121-75-5 | 1.20E+03 | 0 | 1 | -1 | 1 |
| 88 | MCPA | 94-74-6 | 3.10E+01 | 0 | 0 | -1 | 1 |
| 89 | Picloram | 1918-02-1 | 4.30E+03 | 0 | 1 | -1 | 1 |
| 90 | Simazine | 122-34-9 | 4.60E+01 | 1 | 1 | 1 | 1 |
| 91 | Trifluralin | 1582-09-8 | 7.10E+02 | 1 | 1 | 1 | 1 |
| 92 | Total PCB | 1336-36-3 | 1.10E+00 | 0 | 0 | 1 | 1 |
| 94 | Total PCDD/PCDF | 1746-01-6 | 4.50E-05 | 0 | 0 | -1 | -1 |
| 95 | Methyl ethyl ketone | 78-93-3 | 2.30E+04 | 1 | 1 | 1 | 1 |
| 96 | Methyl isobutyl ketone | 108-10-1 | 5.30E+03 | 0 | 1 | 1 | 1 |
| 97 | Dibutyl phthalate | 84-74-2 | 6.10E+03 | 0 | 1 | -1 | 1 |
| 98 | DEHP | 117-81-7 | 3.90E+02 | 1 | 1 | 1 | 1 |
| 99 | Hexachlorobutadine | 87-68-3 | 1.80E+01 | 1 | 1 | 1 | 1 |
| 100 | Methyl tert-butyl ether | 1634-04-4 | 3.20E+02 | 1 | 1 | 1 | 1 |
|  |  | Overall | C1 | C2 | C3 | C4 | C5 |
|  |  | Scores | 93 | 0.85 | 1.01 | 65 | 81 |
|  |  |  |  |  | No. >0 | 79 | 87 |
|  |  |  |  |  | No. <0 | 14 | 6 |

**Table S4c** Arizona Administrative Code Appendix B

| Number | Pollutant | CAS. No | RGV (mg/kg) | C_2_ | C_3_ | C_4_ | C_5_ |
| --- | --- | --- | --- | --- | --- | --- | --- |
| 1 | Antimony | 7440-36-0 | 3.10E+01 | 0 | 1 | 1 | 1 |
| 2 | Arsenic | 7440-38-2 | 1.00E+01 | 0 | 0 | -1 | -1 |
| 3 | Barium | 7440-39-3 | 5.30E+03 | 1 | 1 | 1 | 1 |
| 4 | Beryllium | 7440-41-7 | 1.40E+00 | -1 | -1 | -1 | -1 |
| 5 | Cadmium | 7440-43-9 | 3.80E+01 | 1 | 1 | 1 | 1 |
| 6 | Chromium (III) | 16065-83-1 | 7.70E+04 | 3 | 2 | 1 | 1 |
| 7 | Chromium (VI) | 18540-29-9 | 3.00E+01 | 0 | 0 | -1 | -1 |
| 8 | Cobalt | 7440-48-4 | 4.60E+03 | 2 | 2 | 1 | 1 |
| 9 | Copper | 7440-50-8 | 2.80E+03 | 1 | 1 | 1 | 1 |
| 10 | Lead | 7439-92-1 | 4.00E+02 | 0 | 1 | 1 | 1 |
| 11 | Manganese | 7439-96-5 | 3.20E+03 | 1 | 1 | 1 | 1 |
| 12 | Mercury | 7439-97-6 | 6.70E+00 | 1 | 1 | 1 | 1 |
| 13 | Molybdenum | 7439-98-7 | 3.80E+02 | 1 | 1 | 1 | 1 |
| 14 | Nickel | 7440-02-0 | 1.50E+03 | 1 | 1 | 1 | 1 |
| 15 | Selenium | 7782-49-2 | 3.80E+02 | 1 | 1 | 1 | 1 |
| 16 | Silver | 7440-22-4 | 3.80E+02 | 1 | 1 | 1 | 1 |
| 18 | Tin | 7440-31-5 | 4.60E+04 | 3 | 2 | 1 | 1 |
| 19 | Vanadium | 7440-62-2 | 5.40E+02 | 1 | 1 | 1 | 1 |
| 20 | Zinc | 7440-66-6 | 2.30E+04 | 2 | 2 | 1 | 1 |
| 21 | Cyanide | 57-12-5 | 1.30E+03 | 2 | 2 | 1 | 1 |
| 22 | Acrylonitril | 107-13-1 | 1.90E+00 | 0 | 0 | -1 | -1 |
| 23 | Dichloromethane | 75-09-2 | 7.70E+01 | 1 | 1 | 1 | 1 |
| 24 | Trichloromethane | 67-66-3 | 2.50E+00 | 1 | 0 | 1 | 1 |
| 25 | Tetrachloromethane | 56-23-5 | 1.60E+00 | 0 | 0 | 1 | 1 |
| 26 | Bromoform | 75-25-2 | 5.60E+02 | 1 | 1 | 1 | 1 |
| 27 | Bromomethane | 74-83-9 | 6.80E+00 | 0 | 0 | -1 | -1 |
| 28 | 1,2-Dichloroethane | 107-06-2 | 2.50E+00 | 0 | 0 | 1 | 1 |
| 29 | 1,1,1-Trichloroethane | 71-55-6 | 1.20E+03 | 1 | 1 | 1 | 1 |
| 30 | 1,1,2-Trichloroethane | 79-00-5 | 6.50E+00 | 0 | 1 | 1 | 1 |
| 31 | Chloroethene | 75-01-4 | 8.50E-02 | 0 | 0 | -1 | -1 |
| 32 | 1,1-Dichloroethene | 75-35-4 | 3.60E-01 | -1 | -1 | -1 | -1 |
| 33 | Trichloroethene | 79-01-6 | 2.70E+01 | 1 | 1 | 1 | 1 |
| 34 | Tetrachloroethene | 127-18-4 | 5.30E+01 | 1 | 1 | 1 | 1 |
| 35 | Benzene | 71-43-2 | 6.20E-01 | 0 | 0 | -1 | -1 |
| 36 | Toluene | 108-88-3 | 7.90E+02 | 1 | 1 | 1 | 1 |
| 37 | Chlorobenzene | 108-90-7 | 6.50E+01 | 0 | 1 | 1 | 1 |
| 38 | 1,2-Dichlorobenzene | 95-50-1 | 1.10E+03 | 1 | 1 | 1 | 1 |
| 39 | 1,3-Dichlorobenzene | 541-73-1 | 5.00E+02 | 1 | 2 | 1 | 1 |
| 40 | 1,4-Dichlorobenzene | 106-46-7 | 1.90E+02 | 2 | 2 | 1 | 1 |
| 41 | 1,2,4Trichlorobenzene | 120-82-1 | 5.70E+02 | 2 | 2 | 1 | 1 |
| 42 | Hexachlorobenzene | 118-74-1 | 2.80E+00 | 1 | 1 | 1 | 1 |
| 43 | Nitrobenzene | 98-95-3 | 1.80E+01 | 0 | 0 | -1 | 1 |
| 44 | Ethylbenzene | 100-41-4 | 4.00E+02 | 1 | 1 | 1 | 1 |
| 45 | Styrene | 100-42-5 | 1.50E+03 | 1 | 1 | 1 | 1 |
| 46 | Xylenes | 1330-20-7 | 2.80E+03 | 1 | 2 | 1 | 1 |
| 47 | Cresol | 1319-77-3 | 3.30E+02 | 1 | 1 | 1 | 1 |
| 48 | Phenol | 108-95-2 | 3.90E+04 | 3 | 2 | 1 | 1 |
| 49 | 2-Chlorophenol | 95-57-8 | 9.10E+01 | 1 | 1 | 1 | 1 |
| 50 | 2,4-Dichlorophenol | 120-83-2 | 2.00E+02 | 1 | 2 | 1 | 1 |
| 51 | 2,4,5-Trichlorophenol | 95-95-4 | 6.50E+03 | 1 | 2 | 1 | 1 |
| 52 | 2,4,6-Trichlorophenol | 88-06-2 | 4.00E+02 | 2 | 2 | 1 | 1 |
| 53 | 2,3,4,6-Tetrahlorophenol | 58-90-2 | 2.00E+03 | 1 | 2 | 1 | 1 |
| 54 | Pentachlorophenol | 87-86-5 | 2.50E+01 | 1 | 1 | 1 | 1 |
| 55 | Benz(a)anthracene | 56-55-3 | 6.10E+00 | 1 | 1 | 1 | 1 |
| 56 | Benzo(a)pyrene | 50-32-8 | 6.10E-01 | 0 | 0 | -1 | -1 |
| 57 | Benzo(b)fluoranthene | 205-99-2 | 6.10E+00 | 1 | 1 | 1 | 1 |
| 58 | Benzo(k)fluoranthene | 207-08-9 | 6.10E+01 | 1 | 1 | 1 | 1 |
| 59 | Chrysene | 218-01-9 | 6.10E+02 | 1 | 2 | 1 | 1 |
| 60 | Dibena(a,h)anthracene | 53-70-3 | 6.10E-01 | 0 | 0 | 1 | -1 |
| 61 | Indeno(1,2,3-c,d)pyrebe | 193-39-5 | 6.10E+00 | 1 | 0 | 1 | 1 |
| 62 | Naphthalene | 91-20-3 | 2.60E+03 | 2 | 3 | 1 | 1 |
| 63 | Acenaphehene | 83-32-9 | 3.90E+03 | 2 | 2 | 1 | 1 |
| 65 | Anthracene | 120-12-7 | 2.00E+04 | 3 | 2 | 1 | 1 |
| 67 | Flouranthene | 206-44-0 | 2.60E+03 | 3 | 2 | 1 | 1 |
| 68 | Flouorene | 86-73-7 | 2.60E+03 | 2 | 2 | 1 | 1 |
| 70 | Pyrene | 129-00-0 | 2.00E+03 | 1 | 1 | 1 | 1 |
| 71 | Aldrin | 309-00-2 | 2.60E-01 | 1 | 0 | 1 | 1 |
| 72 | Chlordane | 57-74-9 | 3.40E+00 | 0 | 0 | 1 | 1 |
| 73 | DDT | 50-29-3 | 1.30E+01 | 1 | 1 | 1 | 1 |
| 74 | Dieldrin | 60-57-1 | 2.80E-01 | 1 | 0 | 1 | -1 |
| 75 | Endosulfan | 115-29-7 | 3.90E+02 | 1 | 2 | 1 | 1 |
| 76 | Endrin | 72-20-8 | 2.00E+01 | 1 | 1 | 1 | 1 |
| 77 | Heptachlor | 76-44-8 | 9.90E-01 | 1 | 1 | 1 | 1 |
| 78 | Lindane | 58-89-9 | 3.40E+00 | 1 | 1 | 1 | 1 |
| 79 | Toxaphene | 8001-35-2 | 4.00E+00 | 1 | 1 | 1 | 1 |
| 80 | 2,4-D | 94-75-7 | 6.50E+02 | 0 | 1 | -1 | 1 |
| 81 | Atrazine | 1912-24-9 | 2.00E+01 | 1 | 1 | 1 | 1 |
| 82 | Carbaryl | 63-25-2 | 6.50E+03 | 0 | 2 | 1 | 1 |
| 83 | Carbofuran | 1563-66-2 | 3.30E+02 | 0 | 1 | 1 | 1 |
| 84 | Chloryrifos | 2921-88-2 | 2.00E+02 | 0 | 1 | 1 | 1 |
| 85 | Diuron | 330-54-1 | 1.30E+02 | 0 | 1 | 1 | 1 |
| 86 | Gylphosate | 1071-83-6 | 6.50E+03 | 0 | 1 | 1 | 1 |
| 87 | Malathion | 121-75-5 | 1.30E+03 | 0 | 1 | 1 | 1 |
| 88 | MCPA | 94-74-6 | 3.30E+01 | 0 | 0 | 1 | 1 |
| 89 | Picloram | 1918-02-1 | 4.60E+03 | 0 | 1 | 1 | 1 |
| 90 | Simazine | 122-34-9 | 3.70E+01 | 1 | 1 | 1 | 1 |
| 91 | Trifluralin | 1582-09-8 | 4.90E+02 | 1 | 1 | 1 | 1 |
| 92 | Total PCB | 1336-36-3 | 2.50E+00 | 1 | 1 | 1 | 1 |
| 94 | Total PCDD/PCDF | 1746-01-6 | 3.80E-05 | 0 | 0 | -1 | -1 |
| 95 | Methyl ethyl ketone | 78-93-3 | 7.10E+03 | 0 | 1 | 1 | 1 |
| 96 | Methyl isobutyl ketone | 108-10-1 | 7.70E+02 | 0 | 0 | -1 | 1 |
| 97 | Dibutyl phthalate | 84-74-2 | 6.50E+03 | -1 | 0 | 1 | 1 |
| 98 | DEHP | 117-81-7 | 3.20E+02 | 1 | 1 | 1 | 1 |
| 99 | Hexachlorobutadine | 87-68-3 | 1.30E+01 | 1 | 1 | 1 | 1 |
| 100 | Methyl tert-butyl ether | 1634-04-4 | 3.20E+02 | 1 | 1 | 1 | 1 |
|  |  | Overall | C1 | C2 | C3 | C4 | C5 |
|  |  | Scores | 95 | 0.84 | 0.99 | 69 | 71 |
|  |  |  |  |  | No. >0 | 82 | 83 |
|  |  |  |  |  | No. <0 | 13 | 12 |

**Table S5a** California Human Health Screening Levels - Table S1&S2

| Number | Pollutant | CAS. No | RGV (mg/kg) | C_2_ | C_3_ | C_4_ | C_5_ |
| --- | --- | --- | --- | --- | --- | --- | --- |
| 1 | Antimony | 7440-36-0 | 3.00E+01 | 0 | 1 | 1 | 1 |
| 2 | Arsenic | 7440-38-2 | 7.00E-02 | -2 | -2 | -1 | -1 |
| 3 | Barium | 7440-39-3 | 5.20E+03 | 1 | 1 | 1 | 1 |
| 4 | Beryllium | 7440-41-7 | 1.60E+01 | 0 | 0 | -1 | -1 |
| 5 | Cadmium | 7440-43-9 | 1.70E+00 | 0 | 0 | -1 | -1 |
| 6 | Chromium (III) | 16065-83-1 | 1.00E+05 | 3 | 2 | 1 | 1 |
| 7 | Chromium (VI) | 18540-29-9 | 1.70E+01 | -1 | 0 | -1 | -1 |
| 8 | Cobalt | 7440-48-4 | 6.60E+02 | 1 | 1 | 1 | 1 |
| 9 | Copper | 7440-50-8 | 3.00E+03 | 1 | 1 | 1 | 1 |
| 10 | Lead | 7439-92-1 | 8.00E+01 | 0 | 0 | -1 | -1 |
| 12 | Mercury | 7439-97-6 | 2.00E-01 | -1 | -1 | -1 | -1 |
| 13 | Molybdenum | 7439-98-7 | 3.80E+02 | 1 | 1 | 1 | 1 |
| 14 | Nickel | 7440-02-0 | 1.60E+03 | 1 | 1 | 1 | 1 |
| 15 | Selenium | 7782-49-2 | 3.80E+02 | 1 | 1 | 1 | 1 |
| 16 | Silver | 7440-22-4 | 3.80E+02 | 1 | 1 | 1 | 1 |
| 17 | Thallium | 7440-28-0 | 5.00E+00 | 0 | 0 | 1 | 1 |
| 19 | Vanadium | 7440-62-2 | 5.30E+02 | 1 | 1 | 1 | 1 |
| 20 | Zinc | 7440-66-6 | 2.30E+04 | 2 | 2 | 1 | 1 |
| 25 | Tetrachloromethane | 56-23-5 | 6.30E-02 | -1 | -1 | -1 | -1 |
| 28 | 1,2-Dichloroethane | 107-06-2 | 1.10E-01 | -1 | -1 | -1 | -1 |
| 29 | 1,1,1-Trichloroethane | 71-55-6 | 2.50E+03 | 1 | 1 | 1 | 1 |
| 31 | Chloroethene | 75-01-4 | 2.80E-02 | -1 | -1 | -1 | -1 |
| 35 | Benzene | 71-43-2 | 8.50E-02 | -1 | -1 | -1 | -1 |
| 36 | Toluene | 108-88-3 | 3.20E+02 | 1 | 1 | 1 | 1 |
| 44 | Ethylbenzene | 100-41-4 | 1.10E+00 | -1 | -1 | -1 | -1 |
| 46 | Xylenes | 1330-20-7 | 7.40E+02 | 1 | 1 | 1 | 1 |
| 54 | Pentachlorophenol | 87-86-5 | 4.40E+00 | 0 | 0 | -1 | 1 |
| 56 | Benzo(a)pyrene | 50-32-8 | 3.80E-02 | -1 | -1 | -1 | -1 |
| 62 | Naphthalene | 91-20-3 | 9.30E-02 | -2 | -1 | -1 | -1 |
| 71 | Aldrin | 309-00-2 | 3.30E-02 | 0 | -1 | -1 | -1 |
| 72 | Chlordane | 57-74-9 | 4.30E-01 | -1 | -1 | -1 | -1 |
| 73 | DDT | 50-29-3 | 1.60E+00 | 0 | 0 | -1 | -1 |
| 74 | Dieldrin | 60-57-1 | 3.50E-02 | 0 | -1 | -1 | -1 |
| 76 | Endrin | 72-20-8 | 2.10E+01 | 1 | 1 | 1 | 1 |
| 77 | Heptachlor | 76-44-8 | 1.30E-01 | 0 | 0 | -1 | -1 |
| 78 | Lindane | 58-89-9 | 5.00E-01 | 0 | 0 | -1 | -1 |
| 79 | Toxaphene | 8001-35-2 | 4.60E-01 | 0 | 0 | -1 | -1 |
| 80 | 2,4-D | 94-75-7 | 6.90E+02 | 0 | 1 | -1 | 1 |
| 92 | Total PCB | 1336-36-3 | 8.90E-02 | -1 | -1 | -1 | -1 |
| 94 | Total PCDD/PCDF | 1746-01-6 | 4.60E-06 | -1 | -1 | -1 | -1 |
| 100 | Methyl tert-butyl ether | 1634-04-4 | 8.60E+00 | -1 | 0 | -1 | -1 |
|  |  | Overall | C1 | C2 | C3 | C4 | C5 |
|  |  | Scores | 41 | 0.024 | 0.073 | -9 | -5 |
|  |  |  |  |  | No. >0 | 16 | 18 |
|  |  |  |  |  | No. <0 | 25 | 23 |

**Table S5b** California Human Health Screening Levels - Table S1&S3

| Number | Pollutant | CAS. No | RGV (mg/kg) | C_2_ | C_3_ | C_4_ | C_5_ |
| --- | --- | --- | --- | --- | --- | --- | --- |
| 1 | Antimony | 7440-36-0 | 3.00E+01 | 0 | 1 | 1 | 1 |
| 2 | Arsenic | 7440-38-2 | 7.00E-02 | -2 | -2 | -1 | -1 |
| 3 | Barium | 7440-39-3 | 5.20E+03 | 1 | 1 | 1 | 1 |
| 4 | Beryllium | 7440-41-7 | 1.60E+01 | 0 | 0 | -1 | -1 |
| 5 | Cadmium | 7440-43-9 | 1.70E+00 | 0 | 0 | -1 | -1 |
| 6 | Chromium (III) | 16065-83-1 | 1.00E+05 | 3 | 2 | 1 | 1 |
| 7 | Chromium (VI) | 18540-29-9 | 1.70E+01 | -1 | 0 | -1 | -1 |
| 8 | Cobalt | 7440-48-4 | 6.60E+02 | 1 | 1 | 1 | 1 |
| 9 | Copper | 7440-50-8 | 3.00E+03 | 1 | 1 | 1 | 1 |
| 10 | Lead | 7439-92-1 | 8.00E+01 | 0 | 0 | -1 | -1 |
| 12 | Mercury | 7439-97-6 | 4.50E-02 | -2 | -2 | -1 | -1 |
| 13 | Molybdenum | 7439-98-7 | 3.80E+02 | 1 | 1 | 1 | 1 |
| 14 | Nickel | 7440-02-0 | 1.60E+03 | 1 | 1 | 1 | 1 |
| 15 | Selenium | 7782-49-2 | 3.80E+02 | 1 | 1 | 1 | 1 |
| 16 | Silver | 7440-22-4 | 3.80E+02 | 1 | 1 | 1 | 1 |
| 17 | Thallium | 7440-28-0 | 5.00E+00 | 0 | 0 | 1 | 1 |
| 19 | Vanadium | 7440-62-2 | 5.30E+02 | 1 | 1 | 1 | 1 |
| 20 | Zinc | 7440-66-6 | 2.30E+04 | 2 | 2 | 1 | 1 |
| 25 | Tetrachloromethane | 56-23-5 | 2.50E-02 | -1 | -1 | -1 | -1 |
| 28 | 1,2-Dichloroethane | 107-06-2 | 5.00E-02 | -1 | -1 | -1 | -1 |
| 29 | 1,1,1-Trichloroethane | 71-55-6 | 9.90E+02 | 1 | 1 | 1 | 1 |
| 31 | Chloroethene | 75-01-4 | 1.30E-02 | -1 | -1 | -1 | -1 |
| 35 | Benzene | 71-43-2 | 3.60E-02 | -1 | -1 | -1 | -1 |
| 36 | Toluene | 108-88-3 | 1.40E+02 | 0 | 0 | 1 | 1 |
| 44 | Ethylbenzene | 100-41-4 | 4.20E-01 | -2 | -2 | -1 | -1 |
| 46 | Xylenes | 1330-20-7 | 3.20E+02 | 1 | 1 | 1 | 1 |
| 54 | Pentachlorophenol | 87-86-5 | 4.40E+00 | 0 | 0 | -1 | 1 |
| 56 | Benzo(a)pyrene | 50-32-8 | 3.80E-02 | -1 | -1 | -1 | -1 |
| 62 | Naphthalene | 91-20-3 | 3.20E-02 | -3 | -2 | -1 | -1 |
| 71 | Aldrin | 309-00-2 | 3.30E-02 | 0 | -1 | -1 | -1 |
| 72 | Chlordane | 57-74-9 | 4.30E-01 | -1 | -1 | -1 | -1 |
| 73 | DDT | 50-29-3 | 1.60E+00 | 0 | 0 | -1 | -1 |
| 74 | Dieldrin | 60-57-1 | 3.50E-02 | 0 | -1 | -1 | -1 |
| 76 | Endrin | 72-20-8 | 2.10E+01 | 1 | 1 | 1 | 1 |
| 77 | Heptachlor | 76-44-8 | 1.30E-01 | 0 | 0 | -1 | -1 |
| 78 | Lindane | 58-89-9 | 5.00E-01 | 0 | 0 | -1 | -1 |
| 79 | Toxaphene | 8001-35-2 | 4.60E-01 | 0 | 0 | -1 | -1 |
| 80 | 2,4-D | 94-75-7 | 6.90E+02 | 0 | 1 | -1 | 1 |
| 92 | Total PCB | 1336-36-3 | 8.90E-02 | -1 | -1 | -1 | -1 |
| 94 | Total PCDD/PCDF | 1746-01-6 | 4.60E-06 | -1 | -1 | -1 | -1 |
| 100 | Methyl tert-butyl ether | 1634-04-4 | 4.00E+00 | -1 | -1 | -1 | -1 |
|  |  | Overall | C1 | C2 | C3 | C4 | C5 |
|  |  | Scores | 41 | -0.073 | -0.049 | -9 | -5 |
|  |  |  |  |  | No. >0 | 16 | 18 |
|  |  |  |  |  | No. <0 | 25 | 23 |

**Table S5c** California Human Health Risk Assessment

| Number | Pollutant | CAS. No | RGV (mg/kg) | C_2_ | C_3_ | C_4_ | C_5_ |
| --- | --- | --- | --- | --- | --- | --- | --- |
| 2 | Arsenic | 7440-38-2 | 6.70E-02 | -2 | -2 | -1 | -1 |
| 4 | Beryllium | 7440-41-7 | 1.50E+01 | 0 | 0 | -1 | -1 |
| 5 | Cadmium | 7440-43-9 | 5.20E+00 | 0 | 0 | -1 | -1 |
| 6 | Chromium (III) | 16065-83-1 | 3.60E+04 | 2 | 2 | 1 | 1 |
| 11 | Manganese | 7439-96-5 | 1.80E+03 | 0 | 0 | 1 | 1 |
| 12 | Mercury | 7439-97-6 | 1.00E+00 | 0 | 0 | -1 | -1 |
| 14 | Nickel | 7440-02-0 | 4.90E+02 | 1 | 1 | 1 | 1 |
| 16 | Silver | 7440-22-4 | 3.90E+02 | 1 | 1 | 1 | 1 |
| 22 | Acrylonitril | 107-13-1 | 6.80E-02 | -1 | -1 | -1 | -1 |
| 23 | Dichloromethane | 75-09-2 | 1.90E+00 | -1 | -1 | -1 | -1 |
| 25 | Tetrachloromethane | 56-23-5 | 9.90E-02 | -1 | -1 | -1 | -1 |
| 26 | Bromoform | 75-25-2 | 2.00E+01 | 0 | 0 | -1 | -1 |
| 29 | 1,1,1-Trichloroethane | 71-55-6 | 1.70E+03 | 1 | 1 | 1 | 1 |
| 31 | Chloroethene | 75-01-4 | 8.80E-03 | -1 | -1 | -1 | -1 |
| 34 | Tetrachloroethene | 127-18-4 | 6.00E-01 | -1 | -1 | -1 | -1 |
| 35 | Benzene | 71-43-2 | 3.30E-01 | 0 | 0 | -1 | -1 |
| 36 | Toluene | 108-88-3 | 1.10E+03 | 1 | 1 | 1 | 1 |
| 39 | 1,3-Dichlorobenzene | 541-73-1 | 2.40E+02 | 1 | 1 | 1 | 1 |
| 72 | Chlordane | 57-74-9 | 4.30E-01 | -1 | -1 | -1 | -1 |
| 99 | Hexachlorobutadine | 87-68-3 | 1.20E+00 | 0 | 0 | -1 | -1 |
|  |  | Overall | C1 | C2 | C3 | C4 | C5 |
|  |  | Scores | 20 | -0.05 | -0.05 | -6 | -6 |
|  |  |  |  |  | No. >0 | 7 | 7 |
|  |  |  |  |  | No. <0 | 13 | 13 |

**Table S6** Colorado Dept. of Public Health and Environment

| Number | Pollutant | CAS. No | RGV (mg/kg) | C_2_ | C_3_ | C_4_ | C_5_ |
| --- | --- | --- | --- | --- | --- | --- | --- |
| 1 | Antimony | 7440-36-0 | 3.10E+01 | 0 | 1 | 1 | 1 |
| 2 | Arsenic | 7440-38-2 | 3.90E-01 | -1 | -1 | -1 | -1 |
| 3 | Barium | 7440-39-3 | 1.50E+04 | 1 | 1 | 1 | 1 |
| 4 | Beryllium | 7440-41-7 | 1.60E+02 | 1 | 1 | 1 | 1 |
| 5 | Cadmium | 7440-43-9 | 7.00E+01 | 1 | 1 | 1 | 1 |
| 6 | Chromium (III) | 16065-83-1 | 1.20E+05 | 3 | 2 | 1 | 1 |
| 7 | Chromium (VI) | 18540-29-9 | 1.20E+00 | -2 | -1 | -1 | -1 |
| 8 | Cobalt | 7440-48-4 | 2.30E+01 | 0 | 0 | -1 | -1 |
| 9 | Copper | 7440-50-8 | 3.10E+03 | 1 | 1 | 1 | 1 |
| 10 | Lead | 7439-92-1 | 4.00E+02 | 0 | 1 | 1 | 1 |
| 11 | Manganese | 7439-96-5 | 9.20E+03 | 1 | 1 | 1 | 1 |
| 12 | Mercury | 7439-97-6 | 1.30E+01 | 1 | 1 | 1 | 1 |
| 14 | Nickel | 7440-02-0 | 1.50E+03 | 1 | 1 | 1 | 1 |
| 15 | Selenium | 7782-49-2 | 3.90E+02 | 1 | 1 | 1 | 1 |
| 16 | Silver | 7440-22-4 | 3.90E+02 | 1 | 1 | 1 | 1 |
| 19 | Vanadium | 7440-62-2 | 3.90E+02 | 1 | 1 | 1 | 1 |
| 20 | Zinc | 7440-66-6 | 2.30E+04 | 2 | 2 | 1 | 1 |
| 21 | Cyanide | 57-12-5 | 1.60E+03 | 2 | 2 | 1 | 1 |
| 23 | Dichloromethane | 75-09-2 | 1.20E+01 | 0 | 0 | 1 | -1 |
| 24 | Trichloromethane | 67-66-3 | 2.90E-01 | 0 | -1 | -1 | -1 |
| 25 | Tetrachloromethane | 56-23-5 | 2.40E-01 | 0 | 0 | -1 | -1 |
| 26 | Bromoform | 75-25-2 | 2.50E+01 | 0 | 0 | -1 | -1 |
| 27 | Bromomethane | 74-83-9 | 1.00E+01 | 0 | 0 | 1 | 1 |
| 28 | 1,2-Dichloroethane | 107-06-2 | 4.50E-01 | 0 | 0 | -1 | -1 |
| 29 | 1,1,1-Trichloroethane | 71-55-6 | 9.00E+03 | 1 | 2 | 1 | 1 |
| 30 | 1,1,2-Trichloroethane | 79-00-5 | 1.10E+00 | 0 | 0 | -1 | -1 |
| 31 | Chloroethene | 75-01-4 | 9.00E-02 | 0 | 0 | -1 | -1 |
| 32 | 1,1-Dichloroethene | 75-35-4 | 7.10E+00 | 0 | 0 | -1 | -1 |
| 33 | Trichloroethene | 79-01-6 | 5.20E-02 | -2 | -2 | -1 | -1 |
| 34 | Tetrachloroethene | 127-18-4 | 5.20E-01 | -1 | -1 | -1 | -1 |
| 35 | Benzene | 71-43-2 | 1.20E+00 | 0 | 0 | 1 | 1 |
| 36 | Toluene | 108-88-3 | 4.70E+03 | 2 | 2 | 1 | 1 |
| 37 | Chlorobenzene | 108-90-7 | 3.30E+02 | 1 | 1 | 1 | 1 |
| 38 | 1,2-Dichlorobenzene | 95-50-1 | 2.00E+03 | 1 | 2 | 1 | 1 |
| 40 | 1,4-Dichlorobenzene | 106-46-7 | 2.60E+00 | 0 | 0 | -1 | -1 |
| 41 | 1,2,4Trichlorobenzene | 120-82-1 | 2.00E+01 | 0 | 0 | -1 | -1 |
| 42 | Hexachlorobenzene | 118-74-1 | 3.00E-01 | 0 | 0 | -1 | -1 |
| 43 | Nitrobenzene | 98-95-3 | 4.60E+00 | 0 | 0 | -1 | -1 |
| 44 | Ethylbenzene | 100-41-4 | 6.00E+00 | 0 | 0 | -1 | -1 |
| 45 | Styrene | 100-42-5 | 6.70E+03 | 2 | 2 | 1 | 1 |
| 46 | Xylenes | 1330-20-7 | 7.10E+02 | 1 | 1 | 1 | 1 |
| 47 | Cresol | 1319-77-3 | 3.10E+02 | 0 | 1 | 1 | 1 |
| 48 | Phenol | 108-95-2 | 1.80E+04 | 2 | 2 | 1 | 1 |
| 49 | 2-Chlorophenol | 95-57-8 | 3.60E+02 | 1 | 1 | 1 | 1 |
| 50 | 2,4-Dichlorophenol | 120-83-2 | 1.80E+02 | 1 | 1 | 1 | 1 |
| 51 | 2,4,5-Trichlorophenol | 95-95-4 | 6.10E+03 | 1 | 2 | 1 | 1 |
| 52 | 2,4,6-Trichlorophenol | 88-06-2 | 4.40E+01 | 1 | 1 | 1 | 1 |
| 54 | Pentachlorophenol | 87-86-5 | 3.00E+00 | 0 | 0 | -1 | -1 |
| 55 | Benz(a)anthracene | 56-55-3 | 2.20E-01 | -1 | -1 | -1 | -1 |
| 56 | Benzo(a)pyrene | 50-32-8 | 2.20E-02 | -1 | -1 | -1 | -1 |
| 57 | Benzo(b)fluoranthene | 205-99-2 | 2.20E-01 | -1 | -1 | -1 | -1 |
| 58 | Benzo(k)fluoranthene | 207-08-9 | 2.20E+00 | 0 | 0 | -1 | -1 |
| 59 | Chrysene | 218-01-9 | 2.20E+01 | 0 | 0 | -1 | -1 |
| 60 | Dibena(a,h)anthracene | 53-70-3 | 2.20E-02 | -1 | -1 | -1 | -1 |
| 61 | Indeno(1,2,3-c,d)pyrebe | 193-39-5 | 2.20E-01 | -1 | -1 | -1 | -1 |
| 62 | Naphthalene | 91-20-3 | 1.40E+03 | 2 | 3 | 1 | 1 |
| 63 | Acenaphehene | 83-32-9 | 4.30E+03 | 2 | 2 | 1 | 1 |
| 65 | Anthracene | 120-12-7 | 2.20E+04 | 3 | 2 | 1 | 1 |
| 67 | Flouranthene | 206-44-0 | 2.40E+03 | 3 | 2 | 1 | 1 |
| 68 | Flouorene | 86-73-7 | 2.90E+03 | 2 | 2 | 1 | 1 |
| 70 | Pyrene | 129-00-0 | 1.80E+03 | 1 | 1 | 1 | 1 |
| 71 | Aldrin | 309-00-2 | 2.90E-02 | 0 | -1 | -1 | -1 |
| 72 | Chlordane | 57-74-9 | 1.60E+00 | 0 | 0 | -1 | -1 |
| 73 | DDT | 50-29-3 | 1.70E+00 | 0 | 0 | -1 | -1 |
| 74 | Dieldrin | 60-57-1 | 3.00E-02 | 0 | -1 | -1 | -1 |
| 75 | Endosulfan | 115-29-7 | 3.70E+02 | 1 | 2 | 1 | 1 |
| 76 | Endrin | 72-20-8 | 1.80E+01 | 1 | 1 | 1 | 1 |
| 77 | Heptachlor | 76-44-8 | 1.10E-01 | 0 | 0 | -1 | -1 |
| 78 | Lindane | 58-89-9 | 5.20E-01 | 0 | 0 | -1 | 1 |
| 79 | Toxaphene | 8001-35-2 | 4.40E-01 | 0 | 0 | -1 | -1 |
| 80 | 2,4-D | 94-75-7 | 6.90E+02 | 0 | 1 | -1 | 1 |
| 88 | MCPA | 94-74-6 | 3.10E+01 | 0 | 0 | -1 | 1 |
| 92 | Total PCB | 1336-36-3 | 2.20E-01 | 0 | 0 | -1 | -1 |
| 95 | Methyl ethyl ketone | 78-93-3 | 2.80E+04 | 1 | 2 | 1 | 1 |
| 96 | Methyl isobutyl ketone | 108-10-1 | 5.00E+03 | 0 | 1 | 1 | 1 |
| 97 | Dibutyl phthalate | 84-74-2 | 6.10E+03 | 0 | 1 | -1 | 1 |
| 98 | DEHP | 117-81-7 | 3.50E+01 | 0 | 0 | -1 | -1 |
| 99 | Hexachlorobutadine | 87-68-3 | 6.20E+00 | 0 | 0 | -1 | 1 |
|  |  | Overall | C1 | C2 | C3 | C4 | C5 |
|  |  | Scores | 78 | 0.46 | 0.55 | 2 | 10 |
|  |  |  |  |  | No. >0 | 40 | 44 |
|  |  |  |  |  | No. <0 | 38 | 34 |

**Table S7** Connecticut Dept. of Energy and Environmental Protection

| Number | Pollutant | CAS. No | RGV (mg/kg) | C_2_ | C_3_ | C_4_ | C_5_ |
| --- | --- | --- | --- | --- | --- | --- | --- |
| 1 | Antimony | 7440-36-0 | 3.10E+01 | 0 | 1 | 1 | 1 |
| 2 | Arsenic | 7440-38-2 | 3.90E-01 | -1 | -1 | -1 | -1 |
| 3 | Barium | 7440-39-3 | 1.50E+04 | 1 | 1 | 1 | 1 |
| 4 | Beryllium | 7440-41-7 | 1.60E+02 | 1 | 1 | 1 | 1 |
| 5 | Cadmium | 7440-43-9 | 7.00E+01 | 1 | 1 | 1 | 1 |
| 6 | Chromium (III) | 16065-83-1 | 1.20E+05 | 3 | 2 | 1 | 1 |
| 7 | Chromium (VI) | 18540-29-9 | 1.20E+00 | -2 | -1 | -1 | -1 |
| 8 | Cobalt | 7440-48-4 | 2.30E+01 | 0 | 0 | -1 | -1 |
| 9 | Copper | 7440-50-8 | 3.10E+03 | 1 | 1 | 1 | 1 |
| 10 | Lead | 7439-92-1 | 4.00E+02 | 0 | 1 | 1 | 1 |
| 11 | Manganese | 7439-96-5 | 9.20E+03 | 1 | 1 | 1 | 1 |
| 12 | Mercury | 7439-97-6 | 1.30E+01 | 1 | 1 | 1 | 1 |
| 14 | Nickel | 7440-02-0 | 1.50E+03 | 1 | 1 | 1 | 1 |
| 15 | Selenium | 7782-49-2 | 3.90E+02 | 1 | 1 | 1 | 1 |
| 16 | Silver | 7440-22-4 | 3.90E+02 | 1 | 1 | 1 | 1 |
| 19 | Vanadium | 7440-62-2 | 3.90E+02 | 1 | 1 | 1 | 1 |
| 20 | Zinc | 7440-66-6 | 2.30E+04 | 2 | 2 | 1 | 1 |
| 21 | Cyanide | 57-12-5 | 1.60E+03 | 2 | 2 | 1 | 1 |
| 23 | Dichloromethane | 75-09-2 | 1.20E+01 | 0 | 0 | 1 | -1 |
| 24 | Trichloromethane | 67-66-3 | 2.90E-01 | 0 | -1 | -1 | -1 |
| 25 | Tetrachloromethane | 56-23-5 | 2.40E-01 | 0 | 0 | -1 | -1 |
| 26 | Bromoform | 75-25-2 | 2.50E+01 | 0 | 0 | -1 | -1 |
| 27 | Bromomethane | 74-83-9 | 1.00E+01 | 0 | 0 | 1 | 1 |
| 28 | 1,2-Dichloroethane | 107-06-2 | 4.50E-01 | 0 | 0 | -1 | -1 |
| 29 | 1,1,1-Trichloroethane | 71-55-6 | 9.00E+03 | 1 | 2 | 1 | 1 |
| 30 | 1,1,2-Trichloroethane | 79-00-5 | 1.10E+00 | 0 | 0 | -1 | -1 |
| 31 | Chloroethene | 75-01-4 | 9.00E-02 | 0 | 0 | -1 | -1 |
| 32 | 1,1-Dichloroethene | 75-35-4 | 7.10E+00 | 0 | 0 | -1 | -1 |
| 33 | Trichloroethene | 79-01-6 | 5.20E-02 | -2 | -2 | -1 | -1 |
| 34 | Tetrachloroethene | 127-18-4 | 5.20E-01 | -1 | -1 | -1 | -1 |
| 35 | Benzene | 71-43-2 | 1.20E+00 | 0 | 0 | 1 | 1 |
| 36 | Toluene | 108-88-3 | 4.70E+03 | 2 | 2 | 1 | 1 |
| 37 | Chlorobenzene | 108-90-7 | 3.30E+02 | 1 | 1 | 1 | 1 |
| 38 | 1,2-Dichlorobenzene | 95-50-1 | 2.00E+03 | 1 | 2 | 1 | 1 |
| 40 | 1,4-Dichlorobenzene | 106-46-7 | 2.60E+00 | 0 | 0 | -1 | -1 |
| 41 | 1,2,4Trichlorobenzene | 120-82-1 | 2.00E+01 | 0 | 0 | -1 | -1 |
| 42 | Hexachlorobenzene | 118-74-1 | 3.00E-01 | 0 | 0 | -1 | -1 |
| 43 | Nitrobenzene | 98-95-3 | 4.60E+00 | 0 | 0 | -1 | -1 |
| 44 | Ethylbenzene | 100-41-4 | 6.00E+00 | 0 | 0 | -1 | -1 |
| 45 | Styrene | 100-42-5 | 6.70E+03 | 2 | 2 | 1 | 1 |
| 46 | Xylenes | 1330-20-7 | 7.10E+02 | 1 | 1 | 1 | 1 |
| 47 | Cresol | 1319-77-3 | 3.10E+02 | 0 | 1 | 1 | 1 |
| 48 | Phenol | 108-95-2 | 1.80E+04 | 2 | 2 | 1 | 1 |
| 49 | 2-Chlorophenol | 95-57-8 | 3.60E+02 | 1 | 1 | 1 | 1 |
| 50 | 2,4-Dichlorophenol | 120-83-2 | 1.80E+02 | 1 | 1 | 1 | 1 |
| 51 | 2,4,5-Trichlorophenol | 95-95-4 | 6.10E+03 | 1 | 2 | 1 | 1 |
| 52 | 2,4,6-Trichlorophenol | 88-06-2 | 4.40E+01 | 1 | 1 | 1 | 1 |
| 54 | Pentachlorophenol | 87-86-5 | 3.00E+00 | 0 | 0 | -1 | -1 |
| 55 | Benz(a)anthracene | 56-55-3 | 2.20E-01 | -1 | -1 | -1 | -1 |
| 56 | Benzo(a)pyrene | 50-32-8 | 2.20E-02 | -1 | -1 | -1 | -1 |
| 57 | Benzo(b)fluoranthene | 205-99-2 | 2.20E-01 | -1 | -1 | -1 | -1 |
| 58 | Benzo(k)fluoranthene | 207-08-9 | 2.20E+00 | 0 | 0 | -1 | -1 |
| 59 | Chrysene | 218-01-9 | 2.20E+01 | 0 | 0 | -1 | -1 |
| 60 | Dibena(a,h)anthracene | 53-70-3 | 2.20E-02 | -1 | -1 | -1 | -1 |
| 61 | Indeno(1,2,3-c,d)pyrebe | 193-39-5 | 2.20E-01 | -1 | -1 | -1 | -1 |
| 62 | Naphthalene | 91-20-3 | 1.40E+03 | 2 | 3 | 1 | 1 |
| 63 | Acenaphehene | 83-32-9 | 4.30E+03 | 2 | 2 | 1 | 1 |
| 65 | Anthracene | 120-12-7 | 2.20E+04 | 3 | 2 | 1 | 1 |
| 67 | Flouranthene | 206-44-0 | 2.40E+03 | 3 | 2 | 1 | 1 |
| 68 | Flouorene | 86-73-7 | 2.90E+03 | 2 | 2 | 1 | 1 |
| 70 | Pyrene | 129-00-0 | 1.80E+03 | 1 | 1 | 1 | 1 |
| 71 | Aldrin | 309-00-2 | 2.90E-02 | 0 | -1 | -1 | -1 |
| 72 | Chlordane | 57-74-9 | 1.60E+00 | 0 | 0 | -1 | -1 |
| 73 | DDT | 50-29-3 | 1.70E+00 | 0 | 0 | -1 | -1 |
| 74 | Dieldrin | 60-57-1 | 3.00E-02 | 0 | -1 | -1 | -1 |
| 75 | Endosulfan | 115-29-7 | 3.70E+02 | 1 | 2 | 1 | 1 |
| 76 | Endrin | 72-20-8 | 1.80E+01 | 1 | 1 | 1 | 1 |
| 77 | Heptachlor | 76-44-8 | 1.10E-01 | 0 | 0 | -1 | -1 |
| 78 | Lindane | 58-89-9 | 5.20E-01 | 0 | 0 | -1 | 1 |
| 79 | Toxaphene | 8001-35-2 | 4.40E-01 | 0 | 0 | -1 | -1 |
| 80 | 2,4-D | 94-75-7 | 6.90E+02 | 0 | 1 | -1 | 1 |
| 88 | MCPA | 94-74-6 | 3.10E+01 | 0 | 0 | -1 | 1 |
| 92 | Total PCB | 1336-36-3 | 2.20E-01 | 0 | 0 | -1 | -1 |
| 95 | Methyl ethyl ketone | 78-93-3 | 2.80E+04 | 1 | 2 | 1 | 1 |
| 96 | Methyl isobutyl ketone | 108-10-1 | 5.00E+03 | 0 | 1 | 1 | 1 |
| 97 | Dibutyl phthalate | 84-74-2 | 6.10E+03 | 0 | 1 | -1 | 1 |
| 98 | DEHP | 117-81-7 | 3.50E+01 | 0 | 0 | -1 | -1 |
| 99 | Hexachlorobutadine | 87-68-3 | 6.20E+00 | 0 | 0 | -1 | 1 |
|  |  | Overall | C1 | C2 | C3 | C4 | C5 |
|  |  | Scores | 60 | 0.63 | 0.73 | 26 | 32 |
|  |  |  |  |  | No. >0 | 43 | 46 |
|  |  |  |  |  | No. <0 | 17 | 14 |

**Table S8** Delaware Dept. of Natural Resources and Environmental Control

| Number | Pollutant | CAS. No | RGV (mg/kg) | C_2_ | C_3_ | C_4_ | C_5_ |
| --- | --- | --- | --- | --- | --- | --- | --- |
| 1 | Antimony | 7440-36-0 | 3.10E+00 | -1 | 0 | -1 | -1 |
| 2 | Arsenic | 7440-38-2 | 1.10E+01 | 0 | 0 | -1 | -1 |
| 3 | Barium | 7440-39-3 | 1.50E+03 | 0 | 0 | 1 | 1 |
| 4 | Beryllium | 7440-41-7 | 1.60E+01 | 0 | 0 | -1 | -1 |
| 5 | Cadmium | 7440-43-9 | 7.10E+00 | 0 | 0 | 1 | -1 |
| 6 | Chromium (III) | 16065-83-1 | 1.20E+04 | 2 | 1 | 1 | 1 |
| 7 | Chromium (VI) | 18540-29-9 | 3.00E-01 | -2 | -2 | -1 | -1 |
| 8 | Cobalt | 7440-48-4 | 3.40E+01 | 0 | 0 | -1 | -1 |
| 9 | Copper | 7440-50-8 | 3.10E+02 | 0 | 0 | 1 | 1 |
| 10 | Lead | 7439-92-1 | 4.00E+02 | 0 | 1 | 1 | 1 |
| 11 | Manganese | 7439-96-5 | 2.10E+03 | 0 | 0 | 1 | 1 |
| 12 | Mercury | 7439-97-6 | 1.10E+00 | 0 | 0 | -1 | -1 |
| 13 | Molybdenum | 7439-98-7 | 3.90E+01 | 0 | 0 | -1 | -1 |
| 14 | Nickel | 7440-02-0 | 1.50E+02 | 0 | 0 | 1 | -1 |
| 15 | Selenium | 7782-49-2 | 3.90E+01 | 0 | 0 | 1 | 1 |
| 16 | Silver | 7440-22-4 | 3.90E+01 | 0 | 0 | -1 | -1 |
| 17 | Thallium | 7440-28-0 | 7.80E-02 | -1 | -1 | -1 | -1 |
| 18 | Tin | 7440-31-5 | 4.70E+03 | 2 | 1 | 1 | 1 |
| 19 | Vanadium | 7440-62-2 | 1.34E+02 | 0 | 0 | -1 | -1 |
| 20 | Zinc | 7440-66-6 | 2.30E+03 | 1 | 1 | 1 | 1 |
| 21 | Cyanide | 57-12-5 | 2.30E+00 | -1 | -1 | -1 | -1 |
| 22 | Acrylonitril | 107-13-1 | 8.10E+01 | 2 | 2 | 1 | 1 |
| 23 | Dichloromethane | 75-09-2 | 3.50E+01 | 1 | 1 | 1 | 1 |
| 24 | Trichloromethane | 67-66-3 | 3.20E-01 | 0 | -1 | -1 | -1 |
| 25 | Tetrachloromethane | 56-23-5 | 6.50E-01 | 0 | 0 | -1 | -1 |
| 26 | Bromoform | 75-25-2 | 1.90E+01 | 0 | 0 | -1 | -1 |
| 27 | Bromomethane | 74-83-9 | 6.80E-01 | -1 | -1 | -1 | -1 |
| 28 | 1,2-Dichloroethane | 107-06-2 | 4.60E-01 | 0 | 0 | -1 | -1 |
| 29 | 1,1,1-Trichloroethane | 71-55-6 | 8.10E+02 | 0 | 1 | 1 | 1 |
| 30 | 1,1,2-Trichloroethane | 79-00-5 | 1.50E-01 | -1 | -1 | -1 | -1 |
| 32 | 1,1-Dichloroethene | 75-35-4 | 2.30E+01 | 1 | 1 | 1 | 1 |
| 33 | Trichloroethene | 79-01-6 | 4.10E-01 | -1 | -1 | -1 | -1 |
| 34 | Tetrachloroethene | 127-18-4 | 8.10E+00 | 1 | 1 | 1 | 1 |
| 35 | Benzene | 71-43-2 | 1.20E+00 | 0 | 0 | 1 | 1 |
| 36 | Toluene | 108-88-3 | 4.90E+02 | 1 | 1 | 1 | 1 |
| 37 | Chlorobenzene | 108-90-7 | 2.80E+01 | 0 | 0 | -1 | 1 |
| 38 | 1,2-Dichlorobenzene | 95-50-1 | 1.80E+02 | 0 | 1 | -1 | 1 |
| 40 | 1,4-Dichlorobenzene | 106-46-7 | 2.60E+00 | 0 | 0 | -1 | -1 |
| 41 | 1,2,4Trichlorobenzene | 120-82-1 | 5.80E+00 | 0 | 0 | -1 | -1 |
| 42 | Hexachlorobenzene | 118-74-1 | 2.10E-01 | 0 | 0 | -1 | -1 |
| 43 | Nitrobenzene | 98-95-3 | 5.10E+00 | 0 | 0 | -1 | -1 |
| 44 | Ethylbenzene | 100-41-4 | 5.80E+00 | 0 | 0 | -1 | -1 |
| 45 | Styrene | 100-42-5 | 6.00E+02 | 1 | 1 | 1 | 1 |
| 46 | Xylenes | 1330-20-7 | 5.80E+01 | 0 | 0 | -1 | -1 |
| 47 | Cresol | 1319-77-3 | 6.30E+02 | 1 | 1 | 1 | 1 |
| 48 | Phenol | 108-95-2 | 1.90E+03 | 1 | 1 | 1 | 1 |
| 49 | 2-Chlorophenol | 95-57-8 | 3.90E+01 | 0 | 0 | -1 | 1 |
| 50 | 2,4-Dichlorophenol | 120-83-2 | 1.90E+01 | 0 | 0 | 1 | 1 |
| 51 | 2,4,5-Trichlorophenol | 95-95-4 | 6.30E+02 | 0 | 1 | 1 | 1 |
| 52 | 2,4,6-Trichlorophenol | 88-06-2 | 6.30E+00 | 0 | 0 | -1 | -1 |
| 53 | 2,3,4,6-Tetrahlorophenol | 58-90-2 | 1.90E+02 | 0 | 1 | 1 | 1 |
| 54 | Pentachlorophenol | 87-86-5 | 1.00E+00 | 0 | 0 | -1 | -1 |
| 55 | Benz(a)anthracene | 56-55-3 | 8.20E-01 | 0 | 0 | -1 | -1 |
| 56 | Benzo(a)pyrene | 50-32-8 | 2.40E-01 | 0 | 0 | -1 | -1 |
| 57 | Benzo(b)fluoranthene | 205-99-2 | 1.11E+00 | 0 | 0 | -1 | -1 |
| 58 | Benzo(k)fluoranthene | 207-08-9 | 1.60E+00 | 0 | 0 | -1 | -1 |
| 59 | Chrysene | 218-01-9 | 1.60E+01 | 0 | 0 | -1 | -1 |
| 60 | Dibena(a,h)anthracene | 53-70-3 | 1.70E-01 | 0 | 0 | -1 | -1 |
| 61 | Indeno(1,2,3-c,d)pyrebe | 193-39-5 | 1.30E+00 | 0 | 0 | -1 | -1 |
| 62 | Naphthalene | 91-20-3 | 3.80E+00 | 0 | 0 | -1 | 1 |
| 63 | Acenaphehene | 83-32-9 | 3.60E+02 | 1 | 1 | 1 | 1 |
| 65 | Anthracene | 120-12-7 | 1.80E+03 | 1 | 1 | 1 | 1 |
| 67 | Flouranthene | 206-44-0 | 2.40E+02 | 2 | 1 | 1 | 1 |
| 68 | Flouorene | 86-73-7 | 2.40E+02 | 1 | 0 | 1 | 1 |
| 69 | Phenanthrene | 85-01-8 | 1.80E+02 | 1 | 1 | 1 | 1 |
| 70 | Pyrene | 129-00-0 | 1.80E+02 | 0 | 0 | 1 | 1 |
| 71 | Aldrin | 309-00-2 | 3.90E-02 | 0 | -1 | -1 | -1 |
| 72 | Chlordane | 57-74-9 | 1.70E+00 | 0 | 0 | -1 | -1 |
| 73 | DDT | 50-29-3 | 1.90E+00 | 0 | 0 | -1 | -1 |
| 74 | Dieldrin | 60-57-1 | 3.40E-02 | 0 | -1 | -1 | -1 |
| 75 | Endosulfan | 115-29-7 | 4.70E+01 | 0 | 1 | 1 | 1 |
| 76 | Endrin | 72-20-8 | 1.90E+00 | 0 | 0 | -1 | -1 |
| 77 | Heptachlor | 76-44-8 | 1.30E-01 | 0 | 0 | -1 | -1 |
| 78 | Lindane | 58-89-9 | 5.70E-01 | 0 | 0 | 1 | 1 |
| 79 | Toxaphene | 8001-35-2 | 4.90E-01 | 0 | 0 | -1 | -1 |
| 80 | 2,4-D | 94-75-7 | 7.00E+01 | -1 | 0 | -1 | -1 |
| 81 | Atrazine | 1912-24-9 | 2.40E+00 | 0 | 0 | -1 | -1 |
| 82 | Carbaryl | 63-25-2 | 6.30E+02 | -1 | 1 | -1 | 1 |
| 83 | Carbofuran | 1563-66-2 | 3.20E+01 | -1 | 0 | -1 | 1 |
| 84 | Chloryrifos | 2921-88-2 | 6.30E+00 | -1 | -1 | -1 | -1 |
| 85 | Diuron | 330-54-1 | 1.30E+01 | -1 | 0 | -1 | -1 |
| 86 | Gylphosate | 1071-83-6 | 6.30E+02 | -1 | 0 | -1 | -1 |
| 87 | Malathion | 121-75-5 | 1.30E+02 | -1 | 0 | -1 | -1 |
| 88 | MCPA | 94-74-6 | 3.20E+00 | -1 | -1 | -1 | -1 |
| 89 | Picloram | 1918-02-1 | 4.40E+02 | -1 | 0 | -1 | -1 |
| 90 | Simazine | 122-34-9 | 4.50E+00 | 0 | 0 | 1 | -1 |
| 91 | Trifluralin | 1582-09-8 | 5.90E+01 | 0 | 0 | -1 | -1 |
| 92 | Total PCB | 1336-36-3 | 2.30E-01 | 0 | 0 | -1 | -1 |
| 93 | PCB 118 | 31508-00-6 | 1.20E-01 | 0 | 0 | -1 | -1 |
| 94 | Total PCDD/PCDF | 1746-01-6 | 4.80E-06 | -1 | -1 | -1 | -1 |
| 95 | Methyl ethyl ketone | 78-93-3 | 2.70E+03 | 0 | 1 | -1 | 1 |
| 96 | Methyl isobutyl ketone | 108-10-1 | 3.30E+03 | 0 | 1 | -1 | 1 |
| 97 | Dibutyl phthalate | 84-74-2 | 6.30E+02 | 0 | 1 | -1 | -1 |
| 98 | DEHP | 117-81-7 | 3.90E+01 | 0 | 0 | -1 | -1 |
| 99 | Hexachlorobutadine | 87-68-3 | 1.20E+00 | 0 | 0 | -1 | -1 |
| 100 | Methyl tert-butyl ether | 1634-04-4 | 4.70E+01 | 0 | 0 | -1 | 1 |
|  |  | Overall | C1 | C2 | C3 | C4 | C5 |
|  |  | Scores | 96 | 0.021 | 0.14 | -32 | -20 |
|  |  |  |  |  | No. >0 | 32 | 38 |
|  |  |  |  |  | No. <0 | 64 | 58 |

**Table S9** Florida Dept. of Environmental Protection

| Number | Pollutant | CAS. No | RGV (mg/kg) | C_2_ | C_3_ | C_4_ | C_5_ |
| --- | --- | --- | --- | --- | --- | --- | --- |
| 1 | Antimony | 7440-36-0 | 2.70E+01 | 0 | 0 | 1 | 1 |
| 2 | Arsenic | 7440-38-2 | 2.10E+00 | -1 | -1 | -1 | -1 |
| 3 | Barium | 7440-39-3 | 1.20E+02 | -1 | -1 | -1 | -1 |
| 4 | Beryllium | 7440-41-7 | 1.20E+02 | 1 | 1 | 1 | 1 |
| 5 | Cadmium | 7440-43-9 | 8.20E+01 | 1 | 1 | 1 | 1 |
| 6 | Chromium (III) | 16065-83-1 | 1.10E+05 | 3 | 2 | 1 | 1 |
| 7 | Chromium (VI) | 18540-29-9 | 2.10E+02 | 1 | 1 | 1 | 1 |
| 8 | Cobalt | 7440-48-4 | 1.70E+03 | 2 | 2 | 1 | 1 |
| 9 | Copper | 7440-50-8 | 1.50E+02 | 0 | 0 | -1 | -1 |
| 10 | Lead | 7439-92-1 | 4.00E+02 | 0 | 1 | 1 | 1 |
| 11 | Manganese | 7439-96-5 | 3.50E+03 | 1 | 1 | 1 | 1 |
| 12 | Mercury | 7439-97-6 | 3.00E+00 | 0 | 0 | -1 | -1 |
| 13 | Molybdenum | 7439-98-7 | 4.40E+02 | 1 | 1 | 1 | 1 |
| 14 | Nickel | 7440-02-0 | 3.40E+02 | 1 | 1 | 1 | 1 |
| 15 | Selenium | 7782-49-2 | 4.40E+02 | 1 | 1 | 1 | 1 |
| 16 | Silver | 7440-22-4 | 4.10E+02 | 1 | 1 | 1 | 1 |
| 17 | Thallium | 7440-28-0 | 6.10E+00 | 1 | 1 | 1 | 1 |
| 18 | Tin | 7440-31-5 | 4.70E+04 | 3 | 2 | 1 | 1 |
| 19 | Vanadium | 7440-62-2 | 6.70E+01 | 0 | 0 | -1 | -1 |
| 20 | Zinc | 7440-66-6 | 2.60E+04 | 2 | 2 | 1 | 1 |
| 21 | Cyanide | 57-12-5 | 3.40E+01 | 0 | 0 | -1 | -1 |
| 22 | Acrylonitril | 107-13-1 | 3.00E-01 | -1 | -1 | -1 | -1 |
| 23 | Dichloromethane | 75-09-2 | 1.70E+01 | 0 | 0 | 1 | 1 |
| 24 | Trichloromethane | 67-66-3 | 4.00E-01 | 0 | -1 | -1 | -1 |
| 25 | Tetrachloromethane | 56-23-5 | 5.00E-01 | 0 | 0 | -1 | -1 |
| 26 | Bromoform | 75-25-2 | 4.80E+01 | 0 | 0 | -1 | -1 |
| 27 | Bromomethane | 74-83-9 | 3.10E+00 | 0 | 0 | -1 | -1 |
| 28 | 1,2-Dichloroethane | 107-06-2 | 5.00E-01 | 0 | 0 | -1 | -1 |
| 29 | 1,1,1-Trichloroethane | 71-55-6 | 7.30E+02 | 0 | 1 | 1 | 1 |
| 30 | 1,1,2-Trichloroethane | 79-00-5 | 1.40E+00 | 0 | 0 | -1 | -1 |
| 32 | 1,1-Dichloroethene | 75-35-4 | 9.50E+01 | 1 | 1 | 1 | 1 |
| 33 | Trichloroethene | 79-01-6 | 6.40E+00 | 0 | 1 | 1 | 1 |
| 34 | Tetrachloroethene | 127-18-4 | 8.80E+00 | 1 | 1 | 1 | 1 |
| 35 | Benzene | 71-43-2 | 1.20E+00 | 0 | 0 | 1 | 1 |
| 36 | Toluene | 108-88-3 | 7.50E+03 | 2 | 2 | 1 | 1 |
| 37 | Chlorobenzene | 108-90-7 | 1.20E+02 | 1 | 1 | 1 | 1 |
| 38 | 1,2-Dichlorobenzene | 95-50-1 | 8.80E+02 | 1 | 1 | 1 | 1 |
| 39 | 1,3-Dichlorobenzene | 541-73-1 | 3.80E+02 | 1 | 2 | 1 | 1 |
| 40 | 1,4-Dichlorobenzene | 106-46-7 | 6.40E+00 | 0 | 0 | -1 | -1 |
| 41 | 1,2,4Trichlorobenzene | 120-82-1 | 6.60E+02 | 2 | 2 | 1 | 1 |
| 42 | Hexachlorobenzene | 118-74-1 | 4.00E-01 | 0 | 0 | -1 | -1 |
| 43 | Nitrobenzene | 98-95-3 | 1.80E+01 | 0 | 0 | -1 | 1 |
| 44 | Ethylbenzene | 100-41-4 | 1.50E+03 | 2 | 2 | 1 | 1 |
| 45 | Styrene | 100-42-5 | 3.60E+03 | 2 | 2 | 1 | 1 |
| 46 | Xylenes | 1330-20-7 | 1.30E+02 | 0 | 0 | -1 | 1 |
| 47 | Cresol | 1319-77-3 | 3.00E+02 | 0 | 1 | 1 | 1 |
| 48 | Phenol | 108-95-2 | 5.00E+02 | 1 | 1 | 1 | 1 |
| 49 | 2-Chlorophenol | 95-57-8 | 1.30E+02 | 1 | 1 | 1 | 1 |
| 50 | 2,4-Dichlorophenol | 120-83-2 | 1.90E+02 | 1 | 1 | 1 | 1 |
| 51 | 2,4,5-Trichlorophenol | 95-95-4 | 7.70E+03 | 1 | 2 | 1 | 1 |
| 52 | 2,4,6-Trichlorophenol | 88-06-2 | 7.00E+01 | 1 | 1 | 1 | 1 |
| 53 | 2,3,4,6-Tetrahlorophenol | 58-90-2 | 2.10E+03 | 1 | 2 | 1 | 1 |
| 54 | Pentachlorophenol | 87-86-5 | 7.20E+00 | 0 | 0 | 1 | 1 |
| 56 | Benzo(a)pyrene | 50-32-8 | 1.00E-01 | -1 | -1 | -1 | -1 |
| 62 | Naphthalene | 91-20-3 | 5.50E+01 | 1 | 1 | 1 | 1 |
| 63 | Acenaphehene | 83-32-9 | 2.40E+03 | 2 | 1 | 1 | 1 |
| 64 | Acenaphthylene | 208-96-8 | 1.80E+03 | 2 | 2 | 1 | 1 |
| 65 | Anthracene | 120-12-7 | 2.10E+04 | 3 | 2 | 1 | 1 |
| 66 | Benzo(g,h,i) perylene | 191-24-2 | 2.50E+03 | 2 | 2 | 1 | 1 |
| 67 | Flouranthene | 206-44-0 | 3.20E+03 | 3 | 2 | 1 | 1 |
| 68 | Flouorene | 86-73-7 | 2.60E+03 | 2 | 2 | 1 | 1 |
| 69 | Phenanthrene | 85-01-8 | 2.20E+03 | 2 | 2 | 1 | 1 |
| 70 | Pyrene | 129-00-0 | 2.40E+03 | 1 | 2 | 1 | 1 |
| 71 | Aldrin | 309-00-2 | 6.00E-02 | 0 | 0 | -1 | -1 |
| 72 | Chlordane | 57-74-9 | 2.80E+00 | 0 | 0 | -1 | -1 |
| 73 | DDT | 50-29-3 | 2.90E+00 | 0 | 0 | 1 | -1 |
| 74 | Dieldrin | 60-57-1 | 6.00E-02 | 0 | 0 | -1 | -1 |
| 75 | Endosulfan | 115-29-7 | 4.50E+02 | 1 | 2 | 1 | 1 |
| 76 | Endrin | 72-20-8 | 2.50E+01 | 1 | 1 | 1 | 1 |
| 77 | Heptachlor | 76-44-8 | 2.00E-01 | 0 | 0 | -1 | -1 |
| 78 | Lindane | 58-89-9 | 7.00E-01 | 0 | 0 | 1 | 1 |
| 79 | Toxaphene | 8001-35-2 | 9.00E-01 | 0 | 0 | 1 | -1 |
| 80 | 2,4-D | 94-75-7 | 7.70E+02 | 0 | 1 | 1 | 1 |
| 81 | Atrazine | 1912-24-9 | 4.30E+00 | 0 | 0 | 1 | -1 |
| 82 | Carbaryl | 63-25-2 | 7.70E+03 | 0 | 2 | 1 | 1 |
| 83 | Carbofuran | 1563-66-2 | 1.30E+02 | 0 | 1 | -1 | 1 |
| 84 | Chloryrifos | 2921-88-2 | 2.50E+02 | 1 | 1 | 1 | 1 |
| 85 | Diuron | 330-54-1 | 1.50E+02 | 0 | 1 | 1 | 1 |
| 86 | Gylphosate | 1071-83-6 | 8.80E+03 | 0 | 1 | 1 | 1 |
| 87 | Malathion | 121-75-5 | 1.50E+03 | 0 | 1 | 1 | 1 |
| 88 | MCPA | 94-74-6 | 3.50E+01 | 0 | 0 | 1 | 1 |
| 90 | Simazine | 122-34-9 | 7.80E+00 | 0 | 0 | 1 | 1 |
| 91 | Trifluralin | 1582-09-8 | 9.20E+01 | 0 | 0 | 1 | -1 |
| 92 | Total PCB | 1336-36-3 | 5.00E-01 | 0 | 0 | -1 | -1 |
| 94 | Total PCDD/PCDF | 1746-01-6 | 7.00E-06 | -1 | -1 | -1 | -1 |
| 95 | Methyl ethyl ketone | 78-93-3 | 1.60E+04 | 1 | 1 | 1 | 1 |
| 96 | Methyl isobutyl ketone | 108-10-1 | 4.30E+03 | 0 | 1 | 1 | 1 |
| 97 | Dibutyl phthalate | 84-74-2 | 8.20E+03 | 0 | 1 | 1 | 1 |
| 98 | DEHP | 117-81-7 | 7.20E+01 | 0 | 0 | -1 | -1 |
| 99 | Hexachlorobutadine | 87-68-3 | 6.20E+00 | 0 | 0 | -1 | 1 |
| 100 | Methyl tert-butyl ether | 1634-04-4 | 4.40E+03 | 2 | 2 | 1 | 1 |
|  |  | Overall | C1 | C2 | C3 | C4 | C5 |
|  |  | Scores | 91 | 0.63 | 0.76 | 37 | 37 |
|  |  |  |  |  | No. >0 | 64 | 64 |
|  |  |  |  |  | No. <0 | 27 | 27 |

**Table S10** Georgia Dept. of Natural Resources

| Number | Pollutant | CAS. No | RGV (mg/kg) | C_2_ | C_3_ | C_4_ | C_5_ |
| --- | --- | --- | --- | --- | --- | --- | --- |
| 1 | Antimony | 7440-36-0 | 1.00E+01 | 0 | 0 | -1 | -1 |
| 2 | Arsenic | 7440-38-2 | 4.10E+01 | 1 | 1 | 1 | 1 |
| 3 | Barium | 7440-39-3 | 5.00E+02 | 0 | 0 | -1 | -1 |
| 4 | Beryllium | 7440-41-7 | 3.00E+00 | -1 | -1 | -1 | -1 |
| 5 | Cadmium | 7440-43-9 | 3.90E+01 | 1 | 1 | 1 | 1 |
| 6 | Chromium (III) | 16065-83-1 | 1.20E+03 | 1 | 0 | 1 | 1 |
| 8 | Cobalt | 7440-48-4 | 2.50E+01 | 0 | 0 | -1 | -1 |
| 9 | Copper | 7440-50-8 | 1.50E+03 | 1 | 1 | 1 | 1 |
| 10 | Lead | 7439-92-1 | 4.00E+02 | 0 | 1 | 1 | 1 |
| 11 | Manganese | 7439-96-5 | 2.50E+01 | -2 | -2 | -1 | -1 |
| 12 | Mercury | 7439-97-6 | 1.70E+01 | 1 | 1 | 1 | 1 |
| 14 | Nickel | 7440-02-0 | 4.20E+02 | 1 | 1 | 1 | 1 |
| 15 | Selenium | 7782-49-2 | 3.60E+01 | 0 | 0 | 1 | 1 |
| 16 | Silver | 7440-22-4 | 1.00E+01 | 0 | 0 | -1 | -1 |
| 17 | Thallium | 7440-28-0 | 1.00E+01 | 1 | 1 | 1 | 1 |
| 19 | Vanadium | 7440-62-2 | 1.00E+02 | 0 | 0 | -1 | -1 |
| 20 | Zinc | 7440-66-6 | 2.80E+03 | 1 | 1 | 1 | 1 |
| 21 | Cyanide | 57-12-5 | 1.00E+01 | 0 | 0 | -1 | -1 |
| 22 | Acrylonitril | 107-13-1 | 1.37E+00 | 0 | 0 | -1 | -1 |
| 23 | Dichloromethane | 75-09-2 | 8.00E-02 | -2 | -2 | -1 | -1 |
| 24 | Trichloromethane | 67-66-3 | 6.80E-01 | 0 | 0 | -1 | -1 |
| 25 | Tetrachloromethane | 56-23-5 | 1.70E-01 | -1 | -1 | -1 | -1 |
| 26 | Bromoform | 75-25-2 | 1.00E+00 | -2 | -1 | -1 | -1 |
| 27 | Bromomethane | 74-83-9 | 8.00E-01 | -1 | -1 | -1 | -1 |
| 28 | 1,2-Dichloroethane | 107-06-2 | 2.00E-02 | -2 | -2 | -1 | -1 |
| 29 | 1,1,1-Trichloroethane | 71-55-6 | 5.44E+00 | -2 | -1 | -1 | -1 |
| 30 | 1,1,2-Trichloroethane | 79-00-5 | 5.00E-01 | -1 | -1 | -1 | -1 |
| 31 | Chloroethene | 75-01-4 | 4.00E-02 | 0 | -1 | -1 | -1 |
| 32 | 1,1-Dichloroethene | 75-35-4 | 3.60E-01 | -1 | -1 | -1 | -1 |
| 33 | Trichloroethene | 79-01-6 | 1.30E-01 | -1 | -1 | -1 | -1 |
| 34 | Tetrachloroethene | 127-18-4 | 1.80E-01 | -1 | -1 | -1 | -1 |
| 35 | Benzene | 71-43-2 | 2.00E-02 | -1 | -1 | -1 | -1 |
| 36 | Toluene | 108-88-3 | 1.44E+01 | -1 | -1 | -1 | -1 |
| 37 | Chlorobenzene | 108-90-7 | 4.18E+00 | -1 | -1 | -1 | -1 |
| 38 | 1,2-Dichlorobenzene | 95-50-1 | 2.50E+01 | -1 | 0 | -1 | -1 |
| 39 | 1,3-Dichlorobenzene | 541-73-1 | 2.22E+00 | -1 | -1 | -1 | -1 |
| 40 | 1,4-Dichlorobenzene | 106-46-7 | 6.84E+00 | 0 | 0 | 1 | -1 |
| 41 | 1,2,4Trichlorobenzene | 120-82-1 | 1.08E+01 | 0 | 0 | -1 | -1 |
| 42 | Hexachlorobenzene | 118-74-1 | 2.14E+00 | 1 | 1 | 1 | 1 |
| 43 | Nitrobenzene | 98-95-3 | 7.00E-01 | -1 | -1 | -1 | -1 |
| 44 | Ethylbenzene | 100-41-4 | 2.00E+01 | 0 | 0 | -1 | -1 |
| 45 | Styrene | 100-42-5 | 1.40E+01 | -1 | -1 | -1 | -1 |
| 46 | Xylenes | 1330-20-7 | 2.00E+01 | -1 | -1 | -1 | -1 |
| 47 | Cresol | 1319-77-3 | 3.80E+00 | -1 | -1 | -1 | -1 |
| 48 | Phenol | 108-95-2 | 5.00E+01 | 0 | 0 | -1 | -1 |
| 49 | 2-Chlorophenol | 95-57-8 | 6.80E-01 | -2 | -1 | -1 | -1 |
| 50 | 2,4-Dichlorophenol | 120-83-2 | 9.60E-01 | -1 | -1 | -1 | -1 |
| 51 | 2,4,5-Trichlorophenol | 95-95-4 | 4.56E+00 | -2 | -1 | -1 | -1 |
| 52 | 2,4,6-Trichlorophenol | 88-06-2 | 6.60E-01 | -1 | -1 | -1 | -1 |
| 53 | 2,3,4,6-Tetrahlorophenol | 58-90-2 | 2.50E+01 | -1 | 0 | -1 | -1 |
| 54 | Pentachlorophenol | 87-86-5 | 3.30E+00 | 0 | 0 | -1 | -1 |
| 55 | Benz(a)anthracene | 56-55-3 | 5.00E+00 | 1 | 1 | 1 | 1 |
| 56 | Benzo(a)pyrene | 50-32-8 | 1.64E+00 | 1 | 1 | 1 | 1 |
| 57 | Benzo(b)fluoranthene | 205-99-2 | 5.00E+00 | 1 | 1 | 1 | 1 |
| 58 | Benzo(k)fluoranthene | 207-08-9 | 5.00E+00 | 0 | 0 | -1 | -1 |
| 59 | Chrysene | 218-01-9 | 5.00E+00 | -1 | 0 | -1 | -1 |
| 60 | Dibena(a,h)anthracene | 53-70-3 | 5.00E+00 | 1 | 1 | 1 | 1 |
| 61 | Indeno(1,2,3-c,d)pyrebe | 193-39-5 | 5.00E+00 | 0 | 0 | 1 | 1 |
| 62 | Naphthalene | 91-20-3 | 1.00E+02 | 1 | 2 | 1 | 1 |
| 63 | Acenaphehene | 83-32-9 | 3.00E+02 | 1 | 1 | 1 | 1 |
| 64 | Acenaphthylene | 208-96-8 | 1.30E+02 | 0 | 1 | 1 | 1 |
| 65 | Anthracene | 120-12-7 | 5.00E+02 | 1 | 1 | 1 | 1 |
| 66 | Benzo(g,h,i) perylene | 191-24-2 | 5.00E+02 | 1 | 1 | 1 | 1 |
| 67 | Flouranthene | 206-44-0 | 5.00E+02 | 2 | 1 | 1 | 1 |
| 68 | Flouorene | 86-73-7 | 3.60E+02 | 1 | 1 | 1 | 1 |
| 69 | Phenanthrene | 85-01-8 | 1.10E+02 | 1 | 0 | 1 | 1 |
| 70 | Pyrene | 129-00-0 | 5.00E+02 | 1 | 1 | 1 | 1 |
| 71 | Aldrin | 309-00-2 | 6.60E-01 | 1 | 1 | 1 | 1 |
| 72 | Chlordane | 57-74-9 | 9.20E+00 | 1 | 1 | 1 | 1 |
| 73 | DDT | 50-29-3 | 6.60E-01 | 0 | 0 | -1 | -1 |
| 74 | Dieldrin | 60-57-1 | 6.60E-01 | 1 | 1 | 1 | 1 |
| 75 | Endosulfan | 115-29-7 | 3.30E+00 | -1 | 0 | -1 | -1 |
| 76 | Endrin | 72-20-8 | 1.00E+01 | 1 | 1 | 1 | 1 |
| 77 | Heptachlor | 76-44-8 | 6.60E-01 | 1 | 0 | 1 | 1 |
| 78 | Lindane | 58-89-9 | 6.60E-01 | 0 | 0 | 1 | 1 |
| 79 | Toxaphene | 8001-35-2 | 1.09E+01 | 1 | 1 | 1 | 1 |
| 80 | 2,4-D | 94-75-7 | 1.16E+00 | -3 | -2 | -1 | -1 |
| 82 | Carbaryl | 63-25-2 | 1.00E+00 | -4 | -2 | -1 | -1 |
| 83 | Carbofuran | 1563-66-2 | 8.00E-01 | -2 | -1 | -1 | -1 |
| 84 | Chloryrifos | 2921-88-2 | 1.00E+00 | -2 | -2 | -1 | -1 |
| 85 | Diuron | 330-54-1 | 1.00E+03 | 1 | 2 | 1 | 1 |
| 87 | Malathion | 121-75-5 | 1.00E+00 | -3 | -2 | -1 | -1 |
| 92 | Total PCB | 1336-36-3 | 1.55E+00 | 1 | 1 | 1 | 1 |
| 94 | Total PCDD/PCDF | 1746-01-6 | 8.00E-05 | 0 | 0 | 1 | -1 |
| 95 | Methyl ethyl ketone | 78-93-3 | 7.90E-01 | -4 | -3 | -1 | -1 |
| 96 | Methyl isobutyl ketone | 108-10-1 | 3.30E+00 | -3 | -2 | -1 | -1 |
| 97 | Dibutyl phthalate | 84-74-2 | 1.37E+01 | -3 | -2 | -1 | -1 |
| 98 | DEHP | 117-81-7 | 5.00E+01 | 0 | 0 | -1 | -1 |
| 99 | Hexachlorobutadine | 87-68-3 | 1.75E+01 | 1 | 1 | 1 | 1 |
|  |  | Overall | C1 | C2 | C3 | C4 | C5 |
|  |  | Scores | 89 | -0.31 | -0.15 | -15 | -19 |
|  |  |  |  |  | No. >0 | 37 | 35 |
|  |  |  |  |  | No. <0 | 52 | 54 |

**Table S11** Hawaii Dept. of Health

| Number | Pollutant | CAS. No | RGV (mg/kg) | C_2_ | C_3_ | C_4_ | C_5_ |
| --- | --- | --- | --- | --- | --- | --- | --- |
| 1 | Antimony | 7440-36-0 | 1.60E+00 | -1 | -1 | -1 | -1 |
| 2 | Arsenic | 7440-38-2 | 2.30E+01 | 0 | 1 | 1 | 1 |
| 3 | Barium | 7440-39-3 | 3.10E+03 | 1 | 1 | 1 | 1 |
| 4 | Beryllium | 7440-41-7 | 3.10E+01 | 0 | 0 | 1 | 1 |
| 5 | Cadmium | 7440-43-9 | 1.40E+01 | 1 | 1 | 1 | 1 |
| 6 | Chromium (III) | 16065-83-1 | 2.30E+04 | 2 | 2 | 1 | 1 |
| 7 | Chromium (VI) | 18540-29-9 | 2.90E+01 | 0 | 0 | -1 | -1 |
| 8 | Cobalt | 7440-48-4 | 4.70E+00 | -1 | -1 | -1 | -1 |
| 9 | Copper | 7440-50-8 | 6.30E+02 | 1 | 1 | 1 | 1 |
| 10 | Lead | 7439-92-1 | 2.00E+02 | 0 | 0 | -1 | 1 |
| 12 | Mercury | 7439-97-6 | 4.70E+00 | 0 | 0 | 1 | 1 |
| 13 | Molybdenum | 7439-98-7 | 7.80E+01 | 0 | 0 | 1 | 1 |
| 14 | Nickel | 7440-02-0 | 7.60E+02 | 1 | 1 | 1 | 1 |
| 15 | Selenium | 7782-49-2 | 7.80E+01 | 1 | 1 | 1 | 1 |
| 16 | Silver | 7440-22-4 | 7.80E+01 | 0 | 0 | 1 | 1 |
| 17 | Thallium | 7440-28-0 | 7.80E-01 | 0 | 0 | -1 | -1 |
| 19 | Vanadium | 7440-62-2 | 7.80E+01 | 0 | 0 | -1 | -1 |
| 20 | Zinc | 7440-66-6 | 4.70E+03 | 1 | 1 | 1 | 1 |
| 21 | Cyanide | 57-12-5 | 3.10E+02 | 1 | 1 | 1 | 1 |
| 23 | Dichloromethane | 75-09-2 | 1.10E+01 | 0 | 0 | -1 | -1 |
| 24 | Trichloromethane | 67-66-3 | 3.00E-01 | 0 | -1 | -1 | -1 |
| 25 | Tetrachloromethane | 56-23-5 | 6.20E-01 | 0 | 0 | -1 | -1 |
| 26 | Bromoform | 75-25-2 | 6.10E+01 | 0 | 0 | -1 | 1 |
| 27 | Bromomethane | 74-83-9 | 1.60E+00 | 0 | 0 | -1 | -1 |
| 28 | 1,2-Dichloroethane | 107-06-2 | 4.40E-01 | 0 | 0 | -1 | -1 |
| 29 | 1,1,1-Trichloroethane | 71-55-6 | 6.40E+02 | 0 | 1 | 1 | 1 |
| 30 | 1,1,2-Trichloroethane | 79-00-5 | 3.20E-01 | -1 | -1 | -1 | -1 |
| 31 | Chloroethene | 75-01-4 | 7.20E-02 | 0 | 0 | -1 | -1 |
| 32 | 1,1-Dichloroethene | 75-35-4 | 4.90E+01 | 1 | 1 | 1 | 1 |
| 33 | Trichloroethene | 79-01-6 | 2.80E+00 | 0 | 0 | -1 | -1 |
| 34 | Tetrachloroethene | 127-18-4 | 5.60E-01 | -1 | -1 | -1 | -1 |
| 35 | Benzene | 71-43-2 | 1.10E+00 | 0 | 0 | 1 | 1 |
| 36 | Toluene | 108-88-3 | 8.20E+02 | 1 | 1 | 1 | 1 |
| 37 | Chlorobenzene | 108-90-7 | 5.90E+01 | 0 | 1 | -1 | 1 |
| 38 | 1,2-Dichlorobenzene | 95-50-1 | 1.90E+02 | 0 | 1 | -1 | 1 |
| 39 | 1,3-Dichlorobenzene | 541-73-1 | 1.90E+02 | 1 | 1 | 1 | 1 |
| 40 | 1,4-Dichlorobenzene | 106-46-7 | 2.50E+02 | 2 | 2 | 1 | 1 |
| 41 | 1,2,4Trichlorobenzene | 120-82-1 | 6.60E+00 | 0 | 0 | -1 | -1 |
| 42 | Hexachlorobenzene | 118-74-1 | 3.00E-01 | 0 | 0 | -1 | -1 |
| 43 | Nitrobenzene | 98-95-3 | 4.80E+00 | 0 | 0 | -1 | -1 |
| 44 | Ethylbenzene | 100-41-4 | 5.50E+01 | 1 | 0 | 1 | 1 |
| 45 | Styrene | 100-42-5 | 8.70E+02 | 1 | 1 | 1 | 1 |
| 46 | Xylenes | 1330-20-7 | 1.20E+02 | 0 | 0 | -1 | -1 |
| 48 | Phenol | 108-95-2 | 3.70E+03 | 2 | 1 | 1 | 1 |
| 49 | 2-Chlorophenol | 95-57-8 | 6.70E+01 | 0 | 1 | 1 | 1 |
| 50 | 2,4-Dichlorophenol | 120-83-2 | 3.70E+01 | 1 | 1 | 1 | 1 |
| 51 | 2,4,5-Trichlorophenol | 95-95-4 | 1.20E+03 | 1 | 1 | 1 | 1 |
| 52 | 2,4,6-Trichlorophenol | 88-06-2 | 1.20E+01 | 0 | 0 | -1 | -1 |
| 53 | 2,3,4,6-Tetrahlorophenol | 58-90-2 | 3.70E+02 | 1 | 1 | 1 | 1 |
| 54 | Pentachlorophenol | 87-86-5 | 8.90E-01 | -1 | 0 | -1 | -1 |
| 55 | Benz(a)anthracene | 56-55-3 | 1.50E+00 | 0 | 0 | -1 | -1 |
| 56 | Benzo(a)pyrene | 50-32-8 | 1.50E-01 | 0 | 0 | -1 | -1 |
| 57 | Benzo(b)fluoranthene | 205-99-2 | 1.50E+00 | 0 | 0 | -1 | -1 |
| 58 | Benzo(k)fluoranthene | 207-08-9 | 1.50E+01 | 0 | 1 | 1 | 1 |
| 59 | Chrysene | 218-01-9 | 1.50E+02 | 1 | 1 | 1 | 1 |
| 60 | Dibena(a,h)anthracene | 53-70-3 | 1.50E-01 | 0 | 0 | -1 | -1 |
| 61 | Indeno(1,2,3-c,d)pyrebe | 193-39-5 | 1.50E+00 | 0 | 0 | -1 | -1 |
| 62 | Naphthalene | 91-20-3 | 2.80E+01 | 0 | 1 | 1 | 1 |
| 63 | Acenaphehene | 83-32-9 | 6.20E+02 | 1 | 1 | 1 | 1 |
| 64 | Acenaphthylene | 208-96-8 | 3.20E+02 | 1 | 1 | 1 | 1 |
| 65 | Anthracene | 120-12-7 | 3.30E+03 | 2 | 1 | 1 | 1 |
| 66 | Benzo(g,h,i) perylene | 191-24-2 | 4.60E+02 | 1 | 1 | 1 | 1 |
| 67 | Flouranthene | 206-44-0 | 4.60E+02 | 2 | 1 | 1 | 1 |
| 68 | Flouorene | 86-73-7 | 4.40E+02 | 1 | 1 | 1 | 1 |
| 69 | Phenanthrene | 85-01-8 | 4.40E+02 | 1 | 1 | 1 | 1 |
| 70 | Pyrene | 129-00-0 | 3.40E+02 | 1 | 1 | 1 | 1 |
| 71 | Aldrin | 309-00-2 | 9.20E-01 | 1 | 1 | 1 | 1 |
| 72 | Chlordane | 57-74-9 | 1.60E+01 | 1 | 1 | 1 | 1 |
| 73 | DDT | 50-29-3 | 1.70E+00 | 0 | 0 | -1 | -1 |
| 74 | Dieldrin | 60-57-1 | 1.50E+00 | 1 | 1 | 1 | 1 |
| 75 | Endosulfan | 115-29-7 | 7.30E+01 | 1 | 1 | 1 | 1 |
| 76 | Endrin | 72-20-8 | 3.70E+00 | 0 | 0 | -1 | 1 |
| 77 | Heptachlor | 76-44-8 | 1.10E-01 | 0 | 0 | -1 | -1 |
| 78 | Lindane | 58-89-9 | 5.20E-01 | 0 | 0 | -1 | 1 |
| 79 | Toxaphene | 8001-35-2 | 4.40E-01 | 0 | 0 | -1 | -1 |
| 80 | 2,4-D | 94-75-7 | 1.40E+02 | 0 | 0 | -1 | -1 |
| 81 | Atrazine | 1912-24-9 | 2.10E+00 | 0 | 0 | -1 | -1 |
| 85 | Diuron | 330-54-1 | 2.40E+01 | 0 | 0 | -1 | -1 |
| 86 | Gylphosate | 1071-83-6 | 1.20E+03 | 0 | 0 | -1 | 1 |
| 90 | Simazine | 122-34-9 | 4.00E+00 | 0 | 0 | -1 | -1 |
| 91 | Trifluralin | 1582-09-8 | 6.30E+01 | 0 | 0 | -1 | -1 |
| 92 | Total PCB | 1336-36-3 | 1.10E+00 | 0 | 0 | 1 | 1 |
| 94 | Total PCDD/PCDF | 1746-01-6 | 2.40E-04 | 1 | 1 | 1 | 1 |
| 95 | Methyl ethyl ketone | 78-93-3 | 5.60E+03 | 0 | 1 | -1 | 1 |
| 96 | Methyl isobutyl ketone | 108-10-1 | 1.10E+03 | 0 | 0 | -1 | 1 |
| 98 | DEHP | 117-81-7 | 3.50E+01 | 0 | 0 | -1 | -1 |
| 99 | Hexachlorobutadine | 87-68-3 | 6.20E+00 | 0 | 0 | -1 | 1 |
| 100 | Methyl tert-butyl ether | 1634-04-4 | 4.40E+01 | 0 | 0 | -1 | 1 |
|  |  | Overall | C1 | C2 | C3 | C4 | C5 |
|  |  | Scores | 88 | 0.35 | 0.41 | -2 | 20 |
|  |  |  |  |  | No. >0 | 43 | 54 |
|  |  |  |  |  | No. <0 | 45 | 34 |

**Table S12** Idaho Dept. of Environmental Quality

| Number | Pollutant | CAS. No | RGV (mg/kg) | C_2_ | C_3_ | C_4_ | C_5_ |
| --- | --- | --- | --- | --- | --- | --- | --- |
| 1 | Antimony | 7440-36-0 | 4.77E+00 | 0 | 0 | -1 | -1 |
| 2 | Arsenic | 7440-38-2 | 3.91E-01 | -1 | -1 | -1 | -1 |
| 3 | Barium | 7440-39-3 | 8.96E+02 | 0 | 0 | 1 | -1 |
| 4 | Beryllium | 7440-41-7 | 1.63E+00 | -1 | -1 | -1 | -1 |
| 5 | Cadmium | 7440-43-9 | 1.35E+00 | 0 | -1 | -1 | -1 |
| 6 | Chromium (III) | 16065-83-1 | 2.13E+03 | 1 | 1 | 1 | 1 |
| 7 | Chromium (VI) | 18540-29-9 | 7.90E+00 | -1 | -1 | -1 | -1 |
| 9 | Copper | 7440-50-8 | 9.21E+02 | 1 | 1 | 1 | 1 |
| 10 | Lead | 7439-92-1 | 4.96E+01 | 0 | 0 | -1 | -1 |
| 11 | Manganese | 7439-96-5 | 2.23E+02 | -1 | -1 | -1 | -1 |
| 12 | Mercury | 7439-97-6 | 5.09E-03 | -3 | -3 | -1 | -1 |
| 14 | Nickel | 7440-02-0 | 5.91E+01 | 0 | 0 | -1 | -1 |
| 15 | Selenium | 7782-49-2 | 2.03E+00 | -1 | -1 | -1 | -1 |
| 16 | Silver | 7440-22-4 | 1.89E-01 | -2 | -2 | -1 | -1 |
| 17 | Thallium | 7440-28-0 | 1.55E+00 | 0 | 0 | -1 | -1 |
| 20 | Zinc | 7440-66-6 | 8.86E+02 | 0 | 0 | 1 | -1 |
| 21 | Cyanide | 57-12-5 | 3.68E-01 | -2 | -2 | -1 | -1 |
| 22 | Acrylonitril | 107-13-1 | 1.94E-04 | -4 | -4 | -1 | -1 |
| 24 | Trichloromethane | 67-66-3 | 5.64E-03 | -2 | -2 | -1 | -1 |
| 25 | Tetrachloromethane | 56-23-5 | 1.14E-02 | -2 | -2 | -1 | -1 |
| 26 | Bromoform | 75-25-2 | 2.92E-02 | -3 | -3 | -1 | -1 |
| 27 | Bromomethane | 74-83-9 | 5.01E-02 | -2 | -2 | -1 | -1 |
| 28 | 1,2-Dichloroethane | 107-06-2 | 7.67E-03 | -2 | -2 | -1 | -1 |
| 29 | 1,1,1-Trichloroethane | 71-55-6 | 2.00E+00 | -2 | -2 | -1 | -1 |
| 30 | 1,1,2-Trichloroethane | 79-00-5 | 1.41E-02 | -2 | -2 | -1 | -1 |
| 32 | 1,1-Dichloroethene | 75-35-4 | 3.88E-02 | -2 | -2 | -1 | -1 |
| 34 | Tetrachloroethene | 127-18-4 | 2.88E-02 | -2 | -2 | -1 | -1 |
| 35 | Benzene | 71-43-2 | 1.78E-02 | -1 | -2 | -1 | -1 |
| 36 | Toluene | 108-88-3 | 4.89E+00 | -1 | -1 | -1 | -1 |
| 37 | Chlorobenzene | 108-90-7 | 6.18E-01 | -2 | -1 | -1 | -1 |
| 38 | 1,2-Dichlorobenzene | 95-50-1 | 5.25E+00 | -1 | -1 | -1 | -1 |
| 39 | 1,3-Dichlorobenzene | 541-73-1 | 2.29E-01 | -2 | -2 | -1 | -1 |
| 40 | 1,4-Dichlorobenzene | 106-46-7 | 7.55E-02 | -2 | -2 | -1 | -1 |
| 41 | 1,2,4Trichlorobenzene | 120-82-1 | 6.92E-01 | -1 | -1 | -1 | -1 |
| 42 | Hexachlorobenzene | 118-74-1 | 4.27E-02 | -1 | -1 | -1 | -1 |
| 43 | Nitrobenzene | 98-95-3 | 2.18E-02 | -3 | -3 | -1 | -1 |
| 44 | Ethylbenzene | 100-41-4 | 1.02E+01 | 0 | 0 | -1 | -1 |
| 45 | Styrene | 100-42-5 | 1.83E+00 | -1 | -1 | -1 | -1 |
| 46 | Xylenes | 1330-20-7 | 1.67E+00 | -2 | -2 | -1 | -1 |
| 47 | Cresol | 1319-77-3 | 1.41E-01 | -3 | -2 | -1 | -1 |
| 48 | Phenol | 108-95-2 | 7.36E+00 | -1 | -1 | -1 | -1 |
| 49 | 2-Chlorophenol | 95-57-8 | 3.65E-01 | -2 | -2 | -1 | -1 |
| 50 | 2,4-Dichlorophenol | 120-83-2 | 9.78E-02 | -2 | -2 | -1 | -1 |
| 51 | 2,4,5-Trichlorophenol | 95-95-4 | 7.38E+00 | -2 | -1 | -1 | -1 |
| 52 | 2,4,6-Trichlorophenol | 88-06-2 | 4.36E-03 | -3 | -3 | -1 | -1 |
| 54 | Pentachlorophenol | 87-86-5 | 9.07E-03 | -3 | -2 | -1 | -1 |
| 55 | Benz(a)anthracene | 56-55-3 | 4.22E-01 | 0 | -1 | -1 | -1 |
| 56 | Benzo(a)pyrene | 50-32-8 | 4.22E-02 | -1 | -1 | -1 | -1 |
| 57 | Benzo(b)fluoranthene | 205-99-2 | 4.22E-01 | 0 | -1 | -1 | -1 |
| 58 | Benzo(k)fluoranthene | 207-08-9 | 4.22E+00 | 0 | 0 | -1 | -1 |
| 59 | Chrysene | 218-01-9 | 3.34E+01 | 0 | 0 | -1 | 1 |
| 60 | Dibena(a,h)anthracene | 53-70-3 | 4.22E-02 | -1 | -1 | -1 | -1 |
| 61 | Indeno(1,2,3-c,d)pyrebe | 193-39-5 | 4.22E-01 | -1 | -1 | -1 | -1 |
| 62 | Naphthalene | 91-20-3 | 1.14E+00 | -1 | 0 | -1 | -1 |
| 63 | Acenaphehene | 83-32-9 | 5.23E+01 | 0 | 0 | -1 | -1 |
| 64 | Acenaphthylene | 208-96-8 | 7.80E+01 | 0 | 0 | -1 | 1 |
| 65 | Anthracene | 120-12-7 | 1.04E+03 | 1 | 1 | 1 | 1 |
| 66 | Benzo(g,h,i) perylene | 191-24-2 | 1.18E+03 | 2 | 2 | 1 | 1 |
| 67 | Flouranthene | 206-44-0 | 3.64E+02 | 2 | 1 | 1 | 1 |
| 68 | Flouorene | 86-73-7 | 5.48E+01 | 0 | 0 | -1 | -1 |
| 69 | Phenanthrene | 85-01-8 | 7.90E+01 | 1 | 0 | 1 | 1 |
| 70 | Pyrene | 129-00-0 | 3.59E+02 | 1 | 1 | 1 | 1 |
| 71 | Aldrin | 309-00-2 | 2.11E-02 | 0 | -1 | -1 | -1 |
| 72 | Chlordane | 57-74-9 | 1.53E+00 | 0 | 0 | -1 | -1 |
| 73 | DDT | 50-29-3 | 4.03E-01 | 0 | -1 | -1 | -1 |
| 74 | Dieldrin | 60-57-1 | 1.33E-03 | -2 | -2 | -1 | -1 |
| 75 | Endosulfan | 115-29-7 | 2.49E+00 | -1 | -1 | -1 | -1 |
| 76 | Endrin | 72-20-8 | 3.35E-01 | -1 | -1 | -1 | -1 |
| 77 | Heptachlor | 76-44-8 | 1.06E-03 | -2 | -2 | -1 | -1 |
| 79 | Toxaphene | 8001-35-2 | 3.26E-01 | 0 | 0 | -1 | -1 |
| 80 | 2,4-D | 94-75-7 | 1.84E+00 | -2 | -2 | -1 | -1 |
| 81 | Atrazine | 1912-24-9 | 1.39E-02 | -2 | -2 | -1 | -1 |
| 83 | Carbofuran | 1563-66-2 | 9.42E-02 | -3 | -2 | -1 | -1 |
| 84 | Chloryrifos | 2921-88-2 | 2.84E+00 | -1 | -1 | -1 | -1 |
| 85 | Diuron | 330-54-1 | 2.16E-01 | -3 | -2 | -1 | -1 |
| 86 | Gylphosate | 1071-83-6 | 4.48E+01 | -2 | -1 | -1 | -1 |
| 89 | Picloram | 1918-02-1 | 2.95E+00 | -3 | -2 | -1 | -1 |
| 90 | Simazine | 122-34-9 | 1.08E-02 | -2 | -2 | -1 | -1 |
| 94 | Total PCDD/PCDF | 1746-01-6 | 3.91E-06 | -1 | -1 | -1 | -1 |
| 95 | Methyl ethyl ketone | 78-93-3 | 1.18E+01 | -3 | -2 | -1 | -1 |
| 96 | Methyl isobutyl ketone | 108-10-1 | 1.76E+01 | -2 | -1 | -1 | -1 |
| 97 | Dibutyl phthalate | 84-74-2 | 3.10E+01 | -2 | -2 | -1 | -1 |
| 98 | DEHP | 117-81-7 | 1.18E+01 | -1 | -1 | -1 | -1 |
| 99 | Hexachlorobutadine | 87-68-3 | 3.78E-02 | -2 | -2 | -1 | -1 |
| 100 | Methyl tert-butyl ether | 1634-04-4 | 3.64E-02 | -3 | -3 | -1 | -1 |
|  |  | Overall | C1 | C2 | C3 | C4 | C5 |
|  |  | Scores | 85 | -1.19 | -1.15 | -67 | -67 |
|  |  |  |  |  | No. >0 | 9 | 9 |
|  |  |  |  |  | No. <0 | 76 | 76 |

**Table 13** Illinois Administrative Code

| Number | Pollutant | CAS. No | RGV (mg/kg) | C_2_ | C_3_ | C_4_ | C_5_ |
| --- | --- | --- | --- | --- | --- | --- | --- |
| 1 | Antimony | 7440-36-0 | 3.10E+01 | 0 | 1 | 1 | 1 |
| 2 | Arsenic | 7440-38-2 | 7.50E+02 | 2 | 2 | 1 | 1 |
| 3 | Barium | 7440-39-3 | 5.50E+03 | 1 | 1 | 1 | 1 |
| 4 | Beryllium | 7440-41-7 | 1.60E+02 | 1 | 1 | 1 | 1 |
| 5 | Cadmium | 7440-43-9 | 7.80E+01 | 1 | 1 | 1 | 1 |
| 6 | Chromium (III) | 16065-83-1 | 2.30E+02 | 0 | 0 | -1 | -1 |
| 7 | Chromium (VI) | 18540-29-9 | 2.30E+02 | 1 | 1 | 1 | 1 |
| 8 | Cobalt | 7440-48-4 | 4.70E+03 | 2 | 2 | 1 | 1 |
| 9 | Copper | 7440-50-8 | 2.90E+03 | 1 | 1 | 1 | 1 |
| 10 | Lead | 7439-92-1 | 4.00E+02 | 0 | 1 | 1 | 1 |
| 11 | Manganese | 7439-96-5 | 1.60E+03 | 0 | 0 | 1 | -1 |
| 12 | Mercury | 7439-97-6 | 2.30E+01 | 1 | 1 | 1 | 1 |
| 13 | Molybdenum | 7439-98-7 | 2.38E+03 | 2 | 2 | 1 | 1 |
| 14 | Nickel | 7440-02-0 | 1.60E+03 | 1 | 1 | 1 | 1 |
| 15 | Selenium | 7782-49-2 | 3.90E+02 | 1 | 1 | 1 | 1 |
| 16 | Silver | 7440-22-4 | 3.90E+02 | 1 | 1 | 1 | 1 |
| 17 | Thallium | 7440-28-0 | 6.30E+00 | 1 | 1 | 1 | 1 |
| 18 | Tin | 7440-31-5 | 2.85E+05 | 3 | 3 | 1 | 1 |
| 19 | Vanadium | 7440-62-2 | 5.50E+02 | 1 | 1 | 1 | 1 |
| 20 | Zinc | 7440-66-6 | 2.30E+04 | 2 | 2 | 1 | 1 |
| 21 | Cyanide | 57-12-5 | 1.60E+03 | 2 | 2 | 1 | 1 |
| 22 | Acrylonitril | 107-13-1 | 6.18E-01 | 0 | 0 | -1 | -1 |
| 23 | Dichloromethane | 75-09-2 | 1.30E+01 | 0 | 0 | 1 | 1 |
| 24 | Trichloromethane | 67-66-3 | 1.00E+02 | 2 | 2 | 1 | 1 |
| 25 | Tetrachloromethane | 56-23-5 | 3.00E-01 | 0 | 0 | -1 | -1 |
| 26 | Bromoform | 75-25-2 | 5.30E+01 | 0 | 0 | -1 | -1 |
| 27 | Bromomethane | 74-83-9 | 1.00E+01 | 0 | 0 | 1 | 1 |
| 28 | 1,2-Dichloroethane | 107-06-2 | 4.00E-01 | 0 | 0 | -1 | -1 |
| 29 | 1,1,1-Trichloroethane | 71-55-6 | 1.20E+03 | 1 | 1 | 1 | 1 |
| 30 | 1,1,2-Trichloroethane | 79-00-5 | 3.10E+02 | 2 | 2 | 1 | 1 |
| 31 | Chloroethene | 75-01-4 | 2.80E-01 | 0 | 0 | 1 | -1 |
| 32 | 1,1-Dichloroethene | 75-35-4 | 2.90E+02 | 2 | 2 | 1 | 1 |
| 33 | Trichloroethene | 79-01-6 | 5.00E+00 | 0 | 0 | -1 | 1 |
| 34 | Tetrachloroethene | 127-18-4 | 1.10E+01 | 1 | 1 | 1 | 1 |
| 35 | Benzene | 71-43-2 | 8.00E-01 | 0 | 0 | -1 | -1 |
| 36 | Toluene | 108-88-3 | 6.50E+02 | 1 | 1 | 1 | 1 |
| 37 | Chlorobenzene | 108-90-7 | 1.30E+02 | 1 | 1 | 1 | 1 |
| 38 | 1,2-Dichlorobenzene | 95-50-1 | 5.60E+02 | 1 | 1 | 1 | 1 |
| 40 | 1,4-Dichlorobenzene | 106-46-7 | 1.10E+04 | 3 | 3 | 1 | 1 |
| 41 | 1,2,4Trichlorobenzene | 120-82-1 | 7.80E+02 | 2 | 2 | 1 | 1 |
| 42 | Hexachlorobenzene | 118-74-1 | 4.00E-01 | 0 | 0 | -1 | -1 |
| 43 | Nitrobenzene | 98-95-3 | 3.90E+01 | 1 | 1 | 1 | 1 |
| 44 | Ethylbenzene | 100-41-4 | 4.00E+02 | 1 | 1 | 1 | 1 |
| 45 | Styrene | 100-42-5 | 1.50E+03 | 1 | 1 | 1 | 1 |
| 46 | Xylenes | 1330-20-7 | 3.20E+02 | 1 | 1 | 1 | 1 |
| 47 | Cresol | 1319-77-3 | 4.76E+04 | 3 | 3 | 1 | 1 |
| 48 | Phenol | 108-95-2 | 2.30E+04 | 2 | 2 | 1 | 1 |
| 49 | 2-Chlorophenol | 95-57-8 | 2.38E+03 | 2 | 2 | 1 | 1 |
| 50 | 2,4-Dichlorophenol | 120-83-2 | 1.43E+03 | 2 | 2 | 1 | 1 |
| 51 | 2,4,5-Trichlorophenol | 95-95-4 | 7.80E+03 | 1 | 2 | 1 | 1 |
| 52 | 2,4,6-Trichlorophenol | 88-06-2 | 5.80E+01 | 1 | 1 | 1 | 1 |
| 53 | 2,3,4,6-Tetrahlorophenol | 58-90-2 | 1.43E+04 | 2 | 2 | 1 | 1 |
| 54 | Pentachlorophenol | 87-86-5 | 3.00E+00 | 0 | 0 | -1 | -1 |
| 55 | Benz(a)anthracene | 56-55-3 | 9.00E-01 | 0 | 0 | -1 | -1 |
| 56 | Benzo(a)pyrene | 50-32-8 | 9.00E-02 | -1 | -1 | -1 | -1 |
| 57 | Benzo(b)fluoranthene | 205-99-2 | 9.00E-01 | 0 | 0 | -1 | -1 |
| 58 | Benzo(k)fluoranthene | 207-08-9 | 9.00E+00 | 0 | 0 | 1 | 1 |
| 59 | Chrysene | 218-01-9 | 8.80E+01 | 1 | 1 | 1 | 1 |
| 60 | Dibena(a,h)anthracene | 53-70-3 | 9.00E-02 | -1 | -1 | -1 | -1 |
| 61 | Indeno(1,2,3-c,d)pyrebe | 193-39-5 | 9.00E-01 | 0 | 0 | -1 | -1 |
| 62 | Naphthalene | 91-20-3 | 1.70E+02 | 1 | 2 | 1 | 1 |
| 63 | Acenaphehene | 83-32-9 | 4.70E+03 | 2 | 2 | 1 | 1 |
| 65 | Anthracene | 120-12-7 | 2.30E+04 | 3 | 2 | 1 | 1 |
| 67 | Flouranthene | 206-44-0 | 3.10E+03 | 3 | 2 | 1 | 1 |
| 68 | Flouorene | 86-73-7 | 3.10E+03 | 2 | 2 | 1 | 1 |
| 70 | Pyrene | 129-00-0 | 2.30E+03 | 1 | 1 | 1 | 1 |
| 71 | Aldrin | 309-00-2 | 4.00E-02 | 0 | -1 | -1 | -1 |
| 72 | Chlordane | 57-74-9 | 1.80E+00 | 0 | 0 | -1 | -1 |
| 73 | DDT | 50-29-3 | 2.04E+01 | 1 | 1 | 1 | 1 |
| 74 | Dieldrin | 60-57-1 | 4.00E-02 | 0 | -1 | -1 | -1 |
| 75 | Endosulfan | 115-29-7 | 4.70E+02 | 1 | 2 | 1 | 1 |
| 76 | Endrin | 72-20-8 | 2.30E+01 | 1 | 1 | 1 | 1 |
| 77 | Heptachlor | 76-44-8 | 1.00E-01 | 0 | 0 | -1 | -1 |
| 78 | Lindane | 58-89-9 | 5.00E-01 | 0 | 0 | -1 | -1 |
| 79 | Toxaphene | 8001-35-2 | 6.00E-01 | 0 | 0 | -1 | -1 |
| 80 | 2,4-D | 94-75-7 | 7.80E+02 | 0 | 1 | 1 | 1 |
| 81 | Atrazine | 1912-24-9 | 2.70E+03 | 3 | 3 | 1 | 1 |
| 82 | Carbaryl | 63-25-2 | 4.76E+04 | 1 | 2 | 1 | 1 |
| 83 | Carbofuran | 1563-66-2 | 3.90E+02 | 0 | 1 | 1 | 1 |
| 85 | Diuron | 330-54-1 | 9.52E+02 | 1 | 1 | 1 | 1 |
| 86 | Gylphosate | 1071-83-6 | 4.76E+04 | 1 | 2 | 1 | 1 |
| 87 | Malathion | 121-75-5 | 9.52E+03 | 1 | 2 | 1 | 1 |
| 88 | MCPA | 94-74-6 | 2.38E+02 | 1 | 1 | 1 | 1 |
| 89 | Picloram | 1918-02-1 | 5.50E+03 | 0 | 1 | 1 | 1 |
| 90 | Simazine | 122-34-9 | 3.90E+02 | 2 | 2 | 1 | 1 |
| 91 | Trifluralin | 1582-09-8 | 4.34E+01 | 0 | 0 | -1 | -1 |
| 92 | Total PCB | 1336-36-3 | 1.00E+00 | 0 | 0 | 1 | 1 |
| 93 | PCB 118 | 31508-00-6 | 8.56E-02 | 0 | 0 | -1 | -1 |
| 94 | Total PCDD/PCDF | 1746-01-6 | 2.57E-06 | -1 | -1 | -1 | -1 |
| 95 | Methyl ethyl ketone | 78-93-3 | 2.85E+05 | 2 | 3 | 1 | 1 |
| 96 | Methyl isobutyl ketone | 108-10-1 | 2.02E+06 | 3 | 4 | 1 | 1 |
| 97 | Dibutyl phthalate | 84-74-2 | 2.30E+03 | 3 | 4 | -1 | 1 |
| 98 | DEHP | 117-81-7 | 4.60E+01 | 0 | 0 | -1 | -1 |
| 99 | Hexachlorobutadine | 87-68-3 | 4.28E+00 | 0 | 0 | -1 | 1 |
| 100 | Methyl tert-butyl ether | 1634-04-4 | 1.85E+02 | 1 | 1 | 1 | 1 |
|  |  | Overall | C1 | C2 | C3 | C4 | C5 |
|  |  | Scores | 95 | 0.94 | 1.04 | 43 | 45 |
|  |  |  |  |  | No. >0 | 69 | 70 |
|  |  |  |  |  | No. <0 | 26 | 25 |

**Table S14** Indiana Dept. of Environmental Management

| Number | Pollutant | CAS. No | RGV (mg/kg) | C_2_ | C_3_ | C_4_ | C_5_ |
| --- | --- | --- | --- | --- | --- | --- | --- |
| 1 | Antimony | 7440-36-0 | 4.30E+01 | 1 | 1 | 1 | 1 |
| 2 | Arsenic | 7440-38-2 | 9.50E+00 | 0 | 0 | -1 | -1 |
| 3 | Barium | 7440-39-3 | 2.10E+04 | 2 | 2 | 1 | 1 |
| 4 | Beryllium | 7440-41-7 | 2.20E+02 | 1 | 1 | 1 | 1 |
| 5 | Cadmium | 7440-43-9 | 9.90E+01 | 1 | 1 | 1 | 1 |
| 6 | Chromium (III) | 16065-83-1 | 1.00E+05 | 3 | 2 | 1 | 1 |
| 7 | Chromium (VI) | 18540-29-9 | 4.20E+00 | -1 | -1 | -1 | -1 |
| 8 | Cobalt | 7440-48-4 | 3.20E+01 | 0 | 0 | -1 | -1 |
| 9 | Copper | 7440-50-8 | 4.30E+03 | 2 | 1 | 1 | 1 |
| 10 | Lead | 7439-92-1 | 4.00E+02 | 0 | 1 | 1 | 1 |
| 11 | Manganese | 7439-96-5 | 2.50E+03 | 0 | 0 | 1 | 1 |
| 12 | Mercury | 7439-97-6 | 3.10E+00 | 0 | 0 | -1 | -1 |
| 13 | Molybdenum | 7439-98-7 | 5.50E+02 | 1 | 1 | 1 | 1 |
| 14 | Nickel | 7440-02-0 | 2.10E+03 | 2 | 1 | 1 | 1 |
| 15 | Selenium | 7782-49-2 | 5.50E+02 | 1 | 2 | 1 | 1 |
| 16 | Silver | 7440-22-4 | 5.50E+02 | 1 | 1 | 1 | 1 |
| 17 | Thallium | 7440-28-0 | 1.10E+00 | 0 | 0 | -1 | -1 |
| 18 | Tin | 7440-31-5 | 6.60E+04 | 3 | 2 | 1 | 1 |
| 19 | Vanadium | 7440-62-2 | 5.50E+02 | 1 | 1 | 1 | 1 |
| 20 | Zinc | 7440-66-6 | 3.20E+04 | 2 | 2 | 1 | 1 |
| 21 | Cyanide | 57-12-5 | 3.80E+00 | -1 | -1 | -1 | -1 |
| 22 | Acrylonitril | 107-13-1 | 3.50E+00 | 0 | 0 | 1 | 1 |
| 23 | Dichloromethane | 75-09-2 | 4.90E+02 | 2 | 2 | 1 | 1 |
| 24 | Trichloromethane | 67-66-3 | 4.50E+00 | 1 | 1 | 1 | 1 |
| 25 | Tetrachloromethane | 56-23-5 | 9.10E+00 | 1 | 1 | 1 | 1 |
| 26 | Bromoform | 75-25-2 | 2.70E+02 | 1 | 1 | 1 | 1 |
| 27 | Bromomethane | 74-83-9 | 9.50E+00 | 0 | 0 | 1 | 1 |
| 28 | 1,2-Dichloroethane | 107-06-2 | 6.40E+00 | 1 | 1 | 1 | 1 |
| 29 | 1,1,1-Trichloroethane | 71-55-6 | 6.40E+02 | 0 | 1 | 1 | 1 |
| 30 | 1,1,2-Trichloroethane | 79-00-5 | 2.10E+00 | 0 | 0 | -1 | -1 |
| 31 | Chloroethene | 75-01-4 | 8.30E-01 | 1 | 1 | 1 | 1 |
| 32 | 1,1-Dichloroethene | 75-35-4 | 3.20E+02 | 2 | 2 | 1 | 1 |
| 33 | Trichloroethene | 79-01-6 | 5.70E+00 | 0 | 1 | 1 | 1 |
| 34 | Tetrachloroethene | 127-18-4 | 1.10E+02 | 2 | 2 | 1 | 1 |
| 35 | Benzene | 71-43-2 | 1.70E+01 | 2 | 1 | 1 | 1 |
| 36 | Toluene | 108-88-3 | 8.20E+02 | 1 | 1 | 1 | 1 |
| 37 | Chlorobenzene | 108-90-7 | 3.90E+02 | 1 | 1 | 1 | 1 |
| 38 | 1,2-Dichlorobenzene | 95-50-1 | 3.80E+02 | 1 | 1 | 1 | 1 |
| 40 | 1,4-Dichlorobenzene | 106-46-7 | 3.60E+01 | 1 | 1 | 1 | 1 |
| 41 | 1,2,4Trichlorobenzene | 120-82-1 | 8.10E+01 | 1 | 1 | 1 | 1 |
| 42 | Hexachlorobenzene | 118-74-1 | 2.90E+00 | 1 | 1 | 1 | 1 |
| 43 | Nitrobenzene | 98-95-3 | 7.10E+01 | 1 | 1 | 1 | 1 |
| 44 | Ethylbenzene | 100-41-4 | 8.10E+01 | 1 | 1 | 1 | 1 |
| 45 | Styrene | 100-42-5 | 8.70E+02 | 1 | 1 | 1 | 1 |
| 46 | Xylenes | 1330-20-7 | 2.60E+02 | 0 | 1 | 1 | 1 |
| 47 | Cresol | 1319-77-3 | 8.80E+03 | 2 | 2 | 1 | 1 |
| 48 | Phenol | 108-95-2 | 2.70E+04 | 3 | 2 | 1 | 1 |
| 49 | 2-Chlorophenol | 95-57-8 | 5.50E+02 | 1 | 2 | 1 | 1 |
| 50 | 2,4-Dichlorophenol | 120-83-2 | 2.70E+02 | 1 | 2 | 1 | 1 |
| 51 | 2,4,5-Trichlorophenol | 95-95-4 | 8.80E+03 | 1 | 2 | 1 | 1 |
| 52 | 2,4,6-Trichlorophenol | 88-06-2 | 8.80E+01 | 1 | 1 | 1 | 1 |
| 53 | 2,3,4,6-Tetrahlorophenol | 58-90-2 | 2.70E+03 | 1 | 2 | 1 | 1 |
| 54 | Pentachlorophenol | 87-86-5 | 1.40E+01 | 1 | 1 | 1 | 1 |
| 55 | Benz(a)anthracene | 56-55-3 | 2.20E+00 | 0 | 0 | 1 | -1 |
| 56 | Benzo(a)pyrene | 50-32-8 | 2.20E-01 | 0 | 0 | -1 | -1 |
| 57 | Benzo(b)fluoranthene | 205-99-2 | 2.20E+00 | 0 | 0 | 1 | -1 |
| 58 | Benzo(k)fluoranthene | 207-08-9 | 2.20E+01 | 1 | 1 | 1 | 1 |
| 59 | Chrysene | 218-01-9 | 2.20E+02 | 1 | 1 | 1 | 1 |
| 60 | Dibena(a,h)anthracene | 53-70-3 | 2.20E-01 | 0 | 0 | -1 | -1 |
| 61 | Indeno(1,2,3-c,d)pyrebe | 193-39-5 | 2.20E+00 | 0 | 0 | -1 | -1 |
| 62 | Naphthalene | 91-20-3 | 5.30E+01 | 1 | 1 | 1 | 1 |
| 63 | Acenaphehene | 83-32-9 | 5.00E+03 | 2 | 2 | 1 | 1 |
| 65 | Anthracene | 120-12-7 | 2.50E+04 | 3 | 2 | 1 | 1 |
| 67 | Flouranthene | 206-44-0 | 3.40E+03 | 3 | 2 | 1 | 1 |
| 68 | Flouorene | 86-73-7 | 3.40E+03 | 2 | 2 | 1 | 1 |
| 70 | Pyrene | 129-00-0 | 2.50E+03 | 1 | 2 | 1 | 1 |
| 71 | Aldrin | 309-00-2 | 5.50E-01 | 1 | 1 | 1 | 1 |
| 72 | Chlordane | 57-74-9 | 2.40E+01 | 1 | 1 | 1 | 1 |
| 73 | DDT | 50-29-3 | 2.70E+01 | 1 | 1 | 1 | 1 |
| 74 | Dieldrin | 60-57-1 | 4.80E-01 | 1 | 0 | 1 | 1 |
| 75 | Endosulfan | 115-29-7 | 6.60E+02 | 1 | 2 | 1 | 1 |
| 76 | Endrin | 72-20-8 | 2.70E+01 | 1 | 1 | 1 | 1 |
| 77 | Heptachlor | 76-44-8 | 1.80E+00 | 1 | 1 | 1 | 1 |
| 78 | Lindane | 58-89-9 | 8.00E+00 | 1 | 1 | 1 | 1 |
| 79 | Toxaphene | 8001-35-2 | 6.90E+00 | 1 | 1 | 1 | 1 |
| 80 | 2,4-D | 94-75-7 | 9.80E+02 | 0 | 1 | 1 | 1 |
| 81 | Atrazine | 1912-24-9 | 3.40E+01 | 1 | 1 | 1 | 1 |
| 82 | Carbaryl | 63-25-2 | 8.80E+03 | 0 | 2 | 1 | 1 |
| 83 | Carbofuran | 1563-66-2 | 4.50E+02 | 1 | 2 | 1 | 1 |
| 84 | Chloryrifos | 2921-88-2 | 8.80E+01 | 0 | 0 | -1 | 1 |
| 85 | Diuron | 330-54-1 | 1.80E+02 | 0 | 1 | 1 | 1 |
| 86 | Gylphosate | 1071-83-6 | 8.80E+03 | 0 | 1 | 1 | 1 |
| 87 | Malathion | 121-75-5 | 1.80E+03 | 0 | 1 | 1 | 1 |
| 88 | MCPA | 94-74-6 | 4.50E+01 | 0 | 1 | 1 | 1 |
| 89 | Picloram | 1918-02-1 | 6.20E+03 | 0 | 1 | 1 | 1 |
| 90 | Simazine | 122-34-9 | 6.30E+01 | 1 | 1 | 1 | 1 |
| 91 | Trifluralin | 1582-09-8 | 8.30E+02 | 1 | 1 | 1 | 1 |
| 92 | Total PCB | 1336-36-3 | 3.20E+00 | 1 | 1 | 1 | 1 |
| 93 | PCB 118 | 31508-00-6 | 1.70E+00 | 1 | 1 | 1 | 1 |
| 94 | Total PCDD/PCDF | 1746-01-6 | 6.70E-05 | 0 | 0 | 1 | -1 |
| 95 | Methyl ethyl ketone | 78-93-3 | 2.80E+04 | 1 | 2 | 1 | 1 |
| 96 | Methyl isobutyl ketone | 108-10-1 | 3.40E+03 | 0 | 1 | -1 | 1 |
| 97 | Dibutyl phthalate | 84-74-2 | 8.80E+03 | 0 | 1 | 1 | 1 |
| 98 | DEHP | 117-81-7 | 5.50E+02 | 1 | 1 | 1 | 1 |
| 99 | Hexachlorobutadine | 87-68-3 | 1.70E+01 | 1 | 1 | 1 | 1 |
| 100 | Methyl tert-butyl ether | 1634-04-4 | 6.60E+02 | 1 | 1 | 1 | 1 |
|  |  | Overall | C1 | C2 | C3 | C4 | C5 |
|  |  | Scores | 96 | 0.89 | 1.03 | 72 | 70 |
|  |  |  |  |  | No. >0 | 84 | 83 |
|  |  |  |  |  | No. <0 | 12 | 13 |

**Table S15** Iowa Dept. of Natural Resources

| Number | Pollutant | CAS. No | RGV (mg/kg) | C_2_ | C_3_ | C_4_ | C_5_ |
| --- | --- | --- | --- | --- | --- | --- | --- |
| 1 | Antimony | 7440-36-0 | 3.10E+01 | 0 | 1 | 1 | 1 |
| 2 | Arsenic | 7440-38-2 | 1.70E+01 | 0 | 0 | -1 | 1 |
| 3 | Barium | 7440-39-3 | 1.50E+04 | 1 | 1 | 1 | 1 |
| 4 | Beryllium | 7440-41-7 | 1.10E+02 | 1 | 1 | 1 | 1 |
| 5 | Cadmium | 7440-43-9 | 7.00E+01 | 1 | 1 | 1 | 1 |
| 6 | Chromium (III) | 16065-83-1 | 9.70E+04 | 3 | 2 | 1 | 1 |
| 7 | Chromium (VI) | 18540-29-9 | 2.10E+02 | 1 | 1 | 1 | 1 |
| 8 | Cobalt | 7440-48-4 | 3.10E+01 | 0 | 0 | -1 | -1 |
| 9 | Copper | 7440-50-8 | 1.50E+04 | 2 | 2 | 1 | 1 |
| 10 | Lead | 7439-92-1 | 4.00E+02 | 0 | 1 | 1 | 1 |
| 11 | Manganese | 7439-96-5 | 1.00E+04 | 1 | 1 | 1 | 1 |
| 12 | Mercury | 7439-97-6 | 2.30E+01 | 1 | 1 | 1 | 1 |
| 13 | Molybdenum | 7439-98-7 | 3.90E+02 | 1 | 1 | 1 | 1 |
| 14 | Nickel | 7440-02-0 | 1.50E+03 | 1 | 1 | 1 | 1 |
| 15 | Selenium | 7782-49-2 | 3.90E+02 | 1 | 1 | 1 | 1 |
| 16 | Silver | 7440-22-4 | 3.70E+02 | 1 | 1 | 1 | 1 |
| 17 | Thallium | 7440-28-0 | 7.80E-01 | 0 | 0 | -1 | -1 |
| 18 | Tin | 7440-31-5 | 4.70E+04 | 3 | 2 | 1 | 1 |
| 19 | Vanadium | 7440-62-2 | 3.50E+02 | 1 | 1 | 1 | 1 |
| 20 | Zinc | 7440-66-6 | 2.30E+04 | 2 | 2 | 1 | 1 |
| 21 | Cyanide | 57-12-5 | 4.60E+01 | 0 | 0 | -1 | 1 |
| 22 | Acrylonitril | 107-13-1 | 5.70E+00 | 1 | 1 | 1 | 1 |
| 23 | Dichloromethane | 75-09-2 | 1.50E+03 | 2 | 2 | 1 | 1 |
| 25 | Tetrachloromethane | 56-23-5 | 4.40E+01 | 2 | 2 | 1 | 1 |
| 26 | Bromoform | 75-25-2 | 3.90E+02 | 1 | 1 | 1 | 1 |
| 27 | Bromomethane | 74-83-9 | 1.10E+02 | 1 | 1 | 1 | 1 |
| 28 | 1,2-Dichloroethane | 107-06-2 | 3.40E+01 | 1 | 2 | 1 | 1 |
| 29 | 1,1,1-Trichloroethane | 71-55-6 | 1.50E+05 | 3 | 3 | 1 | 1 |
| 30 | 1,1,2-Trichloroethane | 79-00-5 | 5.40E+01 | 1 | 1 | 1 | 1 |
| 32 | 1,1-Dichloroethene | 75-35-4 | 3.80E+02 | 2 | 2 | 1 | 1 |
| 33 | Trichloroethene | 79-01-6 | 6.70E+01 | 1 | 2 | 1 | 1 |
| 34 | Tetrachloroethene | 127-18-4 | 1.50E+03 | 3 | 3 | 1 | 1 |
| 35 | Benzene | 71-43-2 | 5.60E+01 | 2 | 2 | 1 | 1 |
| 36 | Toluene | 108-88-3 | 6.10E+03 | 2 | 2 | 1 | 1 |
| 37 | Chlorobenzene | 108-90-7 | 1.50E+03 | 2 | 2 | 1 | 1 |
| 38 | 1,2-Dichlorobenzene | 95-50-1 | 5.50E+03 | 2 | 2 | 1 | 1 |
| 39 | 1,3-Dichlorobenzene | 541-73-1 | 6.80E+03 | 3 | 3 | 1 | 1 |
| 40 | 1,4-Dichlorobenzene | 106-46-7 | 7.60E+02 | 2 | 2 | 1 | 1 |
| 41 | 1,2,4Trichlorobenzene | 120-82-1 | 7.60E+02 | 2 | 2 | 1 | 1 |
| 42 | Hexachlorobenzene | 118-74-1 | 1.50E+00 | 1 | 0 | 1 | 1 |
| 44 | Ethylbenzene | 100-41-4 | 7.60E+03 | 3 | 3 | 1 | 1 |
| 45 | Styrene | 100-42-5 | 1.50E+04 | 2 | 2 | 1 | 1 |
| 46 | Xylenes | 1330-20-7 | 1.50E+04 | 2 | 2 | 1 | 1 |
| 47 | Cresol | 1319-77-3 | 3.10E+03 | 1 | 2 | 1 | 1 |
| 48 | Phenol | 108-95-2 | 1.80E+04 | 2 | 2 | 1 | 1 |
| 49 | 2-Chlorophenol | 95-57-8 | 3.10E+02 | 1 | 1 | 1 | 1 |
| 50 | 2,4-Dichlorophenol | 120-83-2 | 1.80E+02 | 1 | 1 | 1 | 1 |
| 51 | 2,4,5-Trichlorophenol | 95-95-4 | 6.10E+03 | 1 | 2 | 1 | 1 |
| 52 | 2,4,6-Trichlorophenol | 88-06-2 | 2.20E+02 | 2 | 1 | 1 | 1 |
| 54 | Pentachlorophenol | 87-86-5 | 4.50E+00 | 0 | 0 | -1 | 1 |
| 55 | Benz(a)anthracene | 56-55-3 | 3.10E+00 | 0 | 0 | 1 | 1 |
| 56 | Benzo(a)pyrene | 50-32-8 | 3.10E-01 | 0 | 0 | -1 | -1 |
| 57 | Benzo(b)fluoranthene | 205-99-2 | 3.10E+00 | 0 | 0 | 1 | 1 |
| 58 | Benzo(k)fluoranthene | 207-08-9 | 3.10E+01 | 1 | 1 | 1 | 1 |
| 59 | Chrysene | 218-01-9 | 3.10E+02 | 1 | 1 | 1 | 1 |
| 60 | Dibena(a,h)anthracene | 53-70-3 | 3.10E-01 | 0 | 0 | -1 | -1 |
| 61 | Indeno(1,2,3-c,d)pyrebe | 193-39-5 | 3.10E+00 | 0 | 0 | 1 | -1 |
| 62 | Naphthalene | 91-20-3 | 1.10E+03 | 2 | 3 | 1 | 1 |
| 63 | Acenaphehene | 83-32-9 | 3.40E+03 | 2 | 2 | 1 | 1 |
| 64 | Acenaphthylene | 208-96-8 | 1.70E+03 | 2 | 2 | 1 | 1 |
| 65 | Anthracene | 120-12-7 | 1.70E+04 | 2 | 2 | 1 | 1 |
| 66 | Benzo(g,h,i) perylene | 191-24-2 | 1.70E+02 | 1 | 1 | 1 | 1 |
| 67 | Flouranthene | 206-44-0 | 2.30E+03 | 3 | 2 | 1 | 1 |
| 68 | Flouorene | 86-73-7 | 2.30E+03 | 1 | 1 | 1 | 1 |
| 69 | Phenanthrene | 85-01-8 | 1.70E+03 | 2 | 2 | 1 | 1 |
| 70 | Pyrene | 129-00-0 | 1.70E+03 | 1 | 1 | 1 | 1 |
| 71 | Aldrin | 309-00-2 | 1.40E-01 | 0 | 0 | 1 | -1 |
| 73 | DDT | 50-29-3 | 8.60E+00 | 1 | 1 | 1 | 1 |
| 74 | Dieldrin | 60-57-1 | 1.50E-01 | 0 | 0 | -1 | -1 |
| 75 | Endosulfan | 115-29-7 | 3.70E+02 | 1 | 2 | 1 | 1 |
| 76 | Endrin | 72-20-8 | 1.80E+01 | 1 | 1 | 1 | 1 |
| 77 | Heptachlor | 76-44-8 | 5.40E-01 | 1 | 0 | 1 | 1 |
| 78 | Lindane | 58-89-9 | 2.60E+00 | 1 | 1 | 1 | 1 |
| 79 | Toxaphene | 8001-35-2 | 2.20E+00 | 1 | 0 | 1 | 1 |
| 80 | 2,4-D | 94-75-7 | 6.90E+02 | 0 | 1 | -1 | 1 |
| 81 | Atrazine | 1912-24-9 | 2.10E+03 | 3 | 3 | 1 | 1 |
| 82 | Carbaryl | 63-25-2 | 2.80E+03 | 0 | 1 | -1 | 1 |
| 83 | Carbofuran | 1563-66-2 | 3.10E+02 | 0 | 1 | 1 | 1 |
| 84 | Chloryrifos | 2921-88-2 | 1.80E+01 | -1 | 0 | -1 | -1 |
| 85 | Diuron | 330-54-1 | 1.30E+02 | 0 | 1 | 1 | 1 |
| 86 | Gylphosate | 1071-83-6 | 6.10E+03 | 0 | 1 | -1 | 1 |
| 87 | Malathion | 121-75-5 | 1.20E+03 | 0 | 1 | -1 | 1 |
| 88 | MCPA | 94-74-6 | 3.10E+01 | 0 | 0 | -1 | 1 |
| 89 | Picloram | 1918-02-1 | 4.30E+03 | 0 | 1 | -1 | 1 |
| 90 | Simazine | 122-34-9 | 3.10E+02 | 2 | 2 | 1 | 1 |
| 91 | Trifluralin | 1582-09-8 | 3.20E+02 | 1 | 1 | 1 | 1 |
| 92 | Total PCB | 1336-36-3 | 1.10E+00 | 0 | 0 | 1 | 1 |
| 94 | Total PCDD/PCDF | 1746-01-6 | 1.60E-05 | 0 | -1 | -1 | -1 |
| 95 | Methyl ethyl ketone | 78-93-3 | 4.60E+04 | 1 | 2 | 1 | 1 |
| 96 | Methyl isobutyl ketone | 108-10-1 | 6.10E+03 | 0 | 1 | 1 | 1 |
| 97 | Dibutyl phthalate | 84-74-2 | 6.10E+03 | 0 | 1 | -1 | 1 |
| 98 | DEHP | 117-81-7 | 1.70E+02 | 1 | 0 | 1 | 1 |
| 99 | Hexachlorobutadine | 87-68-3 | 4.00E+01 | 1 | 1 | 1 | 1 |
| 100 | Methyl tert-butyl ether | 1634-04-4 | 2.30E+03 | 2 | 2 | 1 | 1 |
|  |  | Overall | C1 | C2 | C3 | C4 | C5 |
|  |  | Scores | 94 | 1.11 | 1.21 | 60 | 76 |
|  |  |  |  |  | No. >0 | 77 | 85 |
|  |  |  |  |  | No. <0 | 17 | 9 |

**Table S16** Kansas Dept. of Health and Environment

| Number | Pollutant | CAS. No | RGV (mg/kg) | C_2_ | C_3_ | C_4_ | C_5_ |
| --- | --- | --- | --- | --- | --- | --- | --- |
| 1 | Antimony | 7440-36-0 | 3.13E+01 | 0 | 1 | 1 | 1 |
| 2 | Arsenic | 7440-38-2 | 1.89E+01 | 0 | 0 | -1 | 1 |
| 3 | Barium | 7440-39-3 | 1.53E+04 | 1 | 1 | 1 | 1 |
| 4 | Beryllium | 7440-41-7 | 1.55E+02 | 1 | 1 | 1 | 1 |
| 5 | Cadmium | 7440-43-9 | 3.90E+01 | 1 | 1 | 1 | 1 |
| 6 | Chromium (III) | 16065-83-1 | 3.36E+01 | -1 | -1 | -1 | -1 |
| 7 | Chromium (VI) | 18540-29-9 | 3.36E+01 | 0 | 0 | -1 | -1 |
| 8 | Cobalt | 7440-48-4 | 2.34E+01 | 0 | 0 | -1 | -1 |
| 9 | Copper | 7440-50-8 | 3.13E+03 | 1 | 1 | 1 | 1 |
| 10 | Lead | 7439-92-1 | 4.00E+02 | 0 | 1 | 1 | 1 |
| 11 | Manganese | 7439-96-5 | 9.30E+03 | 1 | 1 | 1 | 1 |
| 12 | Mercury | 7439-97-6 | 2.00E+00 | 0 | 0 | -1 | -1 |
| 14 | Nickel | 7440-02-0 | 1.54E+03 | 1 | 1 | 1 | 1 |
| 15 | Selenium | 7782-49-2 | 3.91E+02 | 1 | 1 | 1 | 1 |
| 16 | Silver | 7440-22-4 | 3.91E+02 | 1 | 1 | 1 | 1 |
| 20 | Zinc | 7440-66-6 | 2.35E+04 | 2 | 2 | 1 | 1 |
| 21 | Cyanide | 57-12-5 | 4.69E+01 | 0 | 0 | -1 | 1 |
| 22 | Acrylonitril | 107-13-1 | 3.18E+00 | 0 | 0 | 1 | 1 |
| 23 | Dichloromethane | 75-09-2 | 3.12E+02 | 2 | 2 | 1 | 1 |
| 24 | Trichloromethane | 67-66-3 | 4.22E+00 | 1 | 1 | 1 | 1 |
| 25 | Tetrachloromethane | 56-23-5 | 8.44E+00 | 1 | 1 | 1 | 1 |
| 26 | Bromoform | 75-25-2 | 1.01E+03 | 1 | 2 | 1 | 1 |
| 27 | Bromomethane | 74-83-9 | 9.39E+00 | 0 | 0 | 1 | 1 |
| 28 | 1,2-Dichloroethane | 107-06-2 | 6.27E+00 | 1 | 1 | 1 | 1 |
| 29 | 1,1,1-Trichloroethane | 71-55-6 | 1.18E+04 | 2 | 2 | 1 | 1 |
| 30 | 1,1,2-Trichloroethane | 79-00-5 | 2.38E+00 | 0 | 0 | -1 | 1 |
| 31 | Chloroethene | 75-01-4 | 4.47E+00 | 2 | 1 | 1 | 1 |
| 32 | 1,1-Dichloroethene | 75-35-4 | 4.68E+01 | 1 | 1 | 1 | 1 |
| 33 | Trichloroethene | 79-01-6 | 5.85E+00 | 0 | 1 | 1 | 1 |
| 34 | Tetrachloroethene | 127-18-4 | 1.09E+02 | 2 | 2 | 1 | 1 |
| 35 | Benzene | 71-43-2 | 1.59E+01 | 2 | 1 | 1 | 1 |
| 36 | Toluene | 108-88-3 | 4.32E+03 | 2 | 2 | 1 | 1 |
| 37 | Chlorobenzene | 108-90-7 | 3.80E+02 | 1 | 1 | 1 | 1 |
| 38 | 1,2-Dichlorobenzene | 95-50-1 | 2.34E+03 | 1 | 2 | 1 | 1 |
| 40 | 1,4-Dichlorobenzene | 106-46-7 | 3.75E+01 | 1 | 1 | 1 | 1 |
| 41 | 1,2,4Trichlorobenzene | 120-82-1 | 8.92E+01 | 1 | 1 | 1 | 1 |
| 42 | Hexachlorobenzene | 118-74-1 | 4.97E+00 | 1 | 1 | 1 | 1 |
| 43 | Nitrobenzene | 98-95-3 | 3.22E+01 | 0 | 1 | 1 | 1 |
| 44 | Ethylbenzene | 100-41-4 | 8.20E+01 | 1 | 1 | 1 | 1 |
| 45 | Styrene | 100-42-5 | 7.02E+03 | 2 | 2 | 1 | 1 |
| 46 | Xylenes | 1330-20-7 | 9.36E+02 | 1 | 1 | 1 | 1 |
| 47 | Cresol | 1319-77-3 | 3.06E+03 | 1 | 2 | 1 | 1 |
| 48 | Phenol | 108-95-2 | 1.83E+04 | 2 | 2 | 1 | 1 |
| 50 | 2,4-Dichlorophenol | 120-83-2 | 1.83E+02 | 1 | 1 | 1 | 1 |
| 51 | 2,4,5-Trichlorophenol | 95-95-4 | 6.11E+03 | 1 | 2 | 1 | 1 |
| 52 | 2,4,6-Trichlorophenol | 88-06-2 | 6.11E+01 | 1 | 1 | 1 | 1 |
| 53 | 2,3,4,6-Tetrahlorophenol | 58-90-2 | 1.83E+03 | 1 | 2 | 1 | 1 |
| 54 | Pentachlorophenol | 87-86-5 | 1.99E+01 | 1 | 1 | 1 | 1 |
| 55 | Benz(a)anthracene | 56-55-3 | 1.09E+01 | 1 | 1 | 1 | 1 |
| 56 | Benzo(a)pyrene | 50-32-8 | 1.09E+00 | 0 | 0 | 1 | 1 |
| 57 | Benzo(b)fluoranthene | 205-99-2 | 1.09E+01 | 1 | 1 | 1 | 1 |
| 58 | Benzo(k)fluoranthene | 207-08-9 | 1.09E+02 | 1 | 1 | 1 | 1 |
| 59 | Chrysene | 218-01-9 | 1.09E+03 | 2 | 2 | 1 | 1 |
| 60 | Dibena(a,h)anthracene | 53-70-3 | 1.09E+00 | 0 | 0 | 1 | 1 |
| 61 | Indeno(1,2,3-c,d)pyrebe | 193-39-5 | 1.09E+01 | 1 | 1 | 1 | 1 |
| 62 | Naphthalene | 91-20-3 | 3.05E+01 | 0 | 1 | 1 | 1 |
| 63 | Acenaphehene | 83-32-9 | 3.42E+03 | 2 | 2 | 1 | 1 |
| 65 | Anthracene | 120-12-7 | 1.80E+04 | 2 | 2 | 1 | 1 |
| 67 | Flouranthene | 206-44-0 | 2.44E+03 | 3 | 2 | 1 | 1 |
| 68 | Flouorene | 86-73-7 | 2.36E+03 | 1 | 1 | 1 | 1 |
| 70 | Pyrene | 129-00-0 | 1.83E+03 | 1 | 1 | 1 | 1 |
| 71 | Aldrin | 309-00-2 | 4.68E-01 | 1 | 1 | 1 | 1 |
| 72 | Chlordane | 57-74-9 | 2.27E+01 | 1 | 1 | 1 | 1 |
| 73 | DDT | 50-29-3 | 2.34E+01 | 1 | 1 | 1 | 1 |
| 75 | Endosulfan | 115-29-7 | 3.67E+02 | 1 | 2 | 1 | 1 |
| 76 | Endrin | 72-20-8 | 1.83E+01 | 1 | 1 | 1 | 1 |
| 77 | Heptachlor | 76-44-8 | 1.77E+00 | 1 | 1 | 1 | 1 |
| 78 | Lindane | 58-89-9 | 7.24E+00 | 1 | 1 | 1 | 1 |
| 79 | Toxaphene | 8001-35-2 | 7.24E+00 | 1 | 1 | 1 | 1 |
| 80 | 2,4-D | 94-75-7 | 6.11E+02 | 0 | 1 | -1 | 1 |
| 81 | Atrazine | 1912-24-9 | 3.46E+01 | 1 | 1 | 1 | 1 |
| 82 | Carbaryl | 63-25-2 | 6.11E+03 | 0 | 2 | 1 | 1 |
| 83 | Carbofuran | 1563-66-2 | 3.06E+02 | 0 | 1 | 1 | 1 |
| 84 | Chloryrifos | 2921-88-2 | 6.11E+01 | 0 | 0 | -1 | -1 |
| 85 | Diuron | 330-54-1 | 1.22E+02 | 0 | 1 | 1 | 1 |
| 86 | Gylphosate | 1071-83-6 | 6.11E+03 | 0 | 1 | 1 | 1 |
| 87 | Malathion | 121-75-5 | 1.22E+03 | 0 | 1 | 1 | 1 |
| 89 | Picloram | 1918-02-1 | 4.28E+03 | 0 | 1 | -1 | 1 |
| 90 | Simazine | 122-34-9 | 6.63E+01 | 1 | 1 | 1 | 1 |
| 94 | Total PCDD/PCDF | 1746-01-6 | 4.28E-05 | 0 | 0 | -1 | -1 |
| 95 | Methyl ethyl ketone | 78-93-3 | 2.60E+04 | 1 | 2 | 1 | 1 |
| 96 | Methyl isobutyl ketone | 108-10-1 | 4.45E+03 | 0 | 1 | 1 | 1 |
| 97 | Dibutyl phthalate | 84-74-2 | 6.11E+03 | 0 | 1 | 1 | 1 |
| 98 | DEHP | 117-81-7 | 5.69E+02 | 1 | 1 | 1 | 1 |
| 99 | Hexachlorobutadine | 87-68-3 | 6.11E+01 | 1 | 1 | 1 | 1 |
| 100 | Methyl tert-butyl ether | 1634-04-4 | 5.85E+02 | 1 | 1 | 1 | 1 |
|  |  | Overall | C1 | C2 | C3 | C4 | C5 |
|  |  | Scores | 86 | 0.84 | 1.06 | 64 | 74 |
|  |  |  |  |  | No. >0 | 75 | 80 |
|  |  |  |  |  | No. <0 | 11 | 6 |

**Table S17a** Louisiana Dept. of Environmental Quality - Table S1

| Number | Pollutant | CAS. No | RGV (mg/kg) | C_2_ | C_3_ | C_4_ | C_5_ |
| --- | --- | --- | --- | --- | --- | --- | --- |
| 1 | Antimony | 7440-36-0 | 3.10E+00 | -1 | 0 | -1 | -1 |
| 2 | Arsenic | 7440-38-2 | 1.20E+01 | 0 | 0 | -1 | 1 |
| 3 | Barium | 7440-39-3 | 5.50E+02 | 0 | 0 | -1 | -1 |
| 4 | Beryllium | 7440-41-7 | 1.60E+01 | 0 | 0 | -1 | -1 |
| 5 | Cadmium | 7440-43-9 | 3.90E+00 | 0 | 0 | -1 | -1 |
| 6 | Chromium (III) | 16065-83-1 | 1.20E+04 | 2 | 1 | 1 | 1 |
| 7 | Chromium (VI) | 18540-29-9 | 2.30E+01 | 0 | 0 | -1 | -1 |
| 8 | Cobalt | 7440-48-4 | 4.70E+02 | 1 | 1 | 1 | 1 |
| 9 | Copper | 7440-50-8 | 3.10E+02 | 0 | 0 | 1 | 1 |
| 10 | Lead | 7439-92-1 | 4.00E+02 | 0 | 1 | 1 | 1 |
| 12 | Mercury | 7439-97-6 | 2.30E+00 | 0 | 0 | -1 | -1 |
| 14 | Nickel | 7440-02-0 | 1.60E+02 | 0 | 0 | 1 | -1 |
| 15 | Selenium | 7782-49-2 | 3.90E+01 | 0 | 0 | 1 | 1 |
| 16 | Silver | 7440-22-4 | 3.90E+01 | 0 | 0 | -1 | -1 |
| 17 | Thallium | 7440-28-0 | 5.50E-01 | 0 | 0 | -1 | -1 |
| 19 | Vanadium | 7440-62-2 | 5.50E+01 | 0 | 0 | -1 | -1 |
| 20 | Zinc | 7440-66-6 | 2.30E+03 | 1 | 1 | 1 | 1 |
| 21 | Cyanide | 57-12-5 | 1.50E+02 | 1 | 1 | 1 | 1 |
| 23 | Dichloromethane | 75-09-2 | 1.90E+01 | 0 | 0 | 1 | 1 |
| 24 | Trichloromethane | 67-66-3 | 4.40E-02 | -1 | -1 | -1 | -1 |
| 25 | Tetrachloromethane | 56-23-5 | 1.80E-01 | -1 | -1 | -1 | -1 |
| 26 | Bromoform | 75-25-2 | 4.80E+01 | 0 | 0 | -1 | -1 |
| 27 | Bromomethane | 74-83-9 | 4.30E-01 | -1 | -1 | -1 | -1 |
| 28 | 1,2-Dichloroethane | 107-06-2 | 8.20E-01 | 0 | 0 | -1 | -1 |
| 29 | 1,1,1-Trichloroethane | 71-55-6 | 8.20E+01 | -1 | 0 | -1 | -1 |
| 30 | 1,1,2-Trichloroethane | 79-00-5 | 1.90E+00 | 0 | 0 | -1 | -1 |
| 31 | Chloroethene | 75-01-4 | 2.40E-01 | 0 | 0 | 1 | -1 |
| 32 | 1,1-Dichloroethene | 75-35-4 | 1.30E+01 | 0 | 0 | 1 | 1 |
| 33 | Trichloroethene | 79-01-6 | 1.00E-01 | -1 | -1 | -1 | -1 |
| 34 | Tetrachloroethene | 127-18-4 | 8.30E+00 | 1 | 1 | 1 | 1 |
| 35 | Benzene | 71-43-2 | 1.50E+00 | 1 | 0 | 1 | 1 |
| 36 | Toluene | 108-88-3 | 6.80E+01 | 0 | 0 | -1 | -1 |
| 37 | Chlorobenzene | 108-90-7 | 1.70E+01 | 0 | 0 | -1 | -1 |
| 38 | 1,2-Dichlorobenzene | 95-50-1 | 9.90E+01 | 0 | 0 | -1 | 1 |
| 39 | 1,3-Dichlorobenzene | 541-73-1 | 2.10E+00 | -1 | -1 | -1 | -1 |
| 40 | 1,4-Dichlorobenzene | 106-46-7 | 6.70E+00 | 0 | 0 | -1 | -1 |
| 41 | 1,2,4Trichlorobenzene | 120-82-1 | 6.60E+01 | 1 | 1 | 1 | 1 |
| 42 | Hexachlorobenzene | 118-74-1 | 3.40E-01 | 0 | 0 | -1 | -1 |
| 43 | Nitrobenzene | 98-95-3 | 2.20E+00 | -1 | -1 | -1 | -1 |
| 44 | Ethylbenzene | 100-41-4 | 1.60E+02 | 1 | 1 | 1 | 1 |
| 45 | Styrene | 100-42-5 | 5.00E+02 | 1 | 1 | 1 | 1 |
| 46 | Xylenes | 1330-20-7 | 1.80E+01 | -1 | -1 | -1 | -1 |
| 48 | Phenol | 108-95-2 | 1.30E+03 | 1 | 1 | 1 | 1 |
| 49 | 2-Chlorophenol | 95-57-8 | 1.50E+01 | 0 | 0 | -1 | -1 |
| 50 | 2,4-Dichlorophenol | 120-83-2 | 1.60E+01 | 0 | 0 | -1 | 1 |
| 51 | 2,4,5-Trichlorophenol | 95-95-4 | 5.30E+02 | 0 | 1 | -1 | 1 |
| 52 | 2,4,6-Trichlorophenol | 88-06-2 | 4.00E+01 | 1 | 1 | 1 | 1 |
| 53 | 2,3,4,6-Tetrahlorophenol | 58-90-2 | 1.40E+02 | 0 | 0 | -1 | 1 |
| 54 | Pentachlorophenol | 87-86-5 | 2.80E+00 | 0 | 0 | -1 | -1 |
| 55 | Benz(a)anthracene | 56-55-3 | 6.20E-01 | 0 | 0 | -1 | -1 |
| 56 | Benzo(a)pyrene | 50-32-8 | 3.30E-01 | 0 | 0 | -1 | -1 |
| 57 | Benzo(b)fluoranthene | 205-99-2 | 6.20E-01 | 0 | 0 | -1 | -1 |
| 58 | Benzo(k)fluoranthene | 207-08-9 | 6.20E+00 | 0 | 0 | -1 | -1 |
| 59 | Chrysene | 218-01-9 | 6.20E+01 | 1 | 1 | 1 | 1 |
| 60 | Dibena(a,h)anthracene | 53-70-3 | 3.30E-01 | 0 | 0 | -1 | -1 |
| 61 | Indeno(1,2,3-c,d)pyrebe | 193-39-5 | 6.20E-01 | 0 | -1 | -1 | -1 |
| 62 | Naphthalene | 91-20-3 | 6.20E+00 | 0 | 1 | -1 | 1 |
| 63 | Acenaphehene | 83-32-9 | 3.70E+02 | 1 | 1 | 1 | 1 |
| 64 | Acenaphthylene | 208-96-8 | 3.50E+02 | 1 | 1 | 1 | 1 |
| 65 | Anthracene | 120-12-7 | 2.20E+03 | 2 | 1 | 1 | 1 |
| 67 | Flouranthene | 206-44-0 | 2.20E+02 | 2 | 1 | 1 | 1 |
| 68 | Flouorene | 86-73-7 | 2.80E+02 | 1 | 1 | 1 | 1 |
| 69 | Phenanthrene | 85-01-8 | 2.10E+03 | 2 | 2 | 1 | 1 |
| 70 | Pyrene | 129-00-0 | 2.30E+02 | 0 | 0 | 1 | 1 |
| 71 | Aldrin | 309-00-2 | 2.80E-02 | 0 | -1 | -1 | -1 |
| 72 | Chlordane | 57-74-9 | 1.60E+00 | 0 | 0 | -1 | -1 |
| 73 | DDT | 50-29-3 | 1.70E+00 | 0 | 0 | -1 | -1 |
| 74 | Dieldrin | 60-57-1 | 3.00E-02 | 0 | -1 | -1 | -1 |
| 75 | Endosulfan | 115-29-7 | 3.40E+01 | 0 | 1 | -1 | 1 |
| 76 | Endrin | 72-20-8 | 1.80E+00 | 0 | 0 | -1 | -1 |
| 77 | Heptachlor | 76-44-8 | 1.60E-02 | -1 | -1 | -1 | -1 |
| 78 | Lindane | 58-89-9 | 3.90E-01 | 0 | 0 | -1 | -1 |
| 79 | Toxaphene | 8001-35-2 | 4.40E-01 | 0 | 0 | -1 | -1 |
| 92 | Total PCB | 1336-36-3 | 1.10E-01 | -1 | -1 | -1 | -1 |
| 95 | Methyl ethyl ketone | 78-93-3 | 5.90E+02 | -1 | 0 | -1 | -1 |
| 96 | Methyl isobutyl ketone | 108-10-1 | 4.50E+02 | -1 | 0 | -1 | -1 |
| 98 | DEHP | 117-81-7 | 3.50E+01 | 0 | 0 | -1 | -1 |
| 99 | Hexachlorobutadine | 87-68-3 | 8.20E-01 | -1 | 0 | -1 | -1 |
| 100 | Methyl tert-butyl ether | 1634-04-4 | 6.50E+02 | 1 | 1 | 1 | 1 |
|  |  | Overall | C1 | C2 | C3 | C4 | C5 |
|  |  | Scores | 79 | 0.10 | 0.14 | -25 | -15 |
|  |  |  |  |  | No. >0 | 27 | 32 |
|  |  |  |  |  | No. <0 | 52 | 47 |

**Table S17b** Louisiana Dept. of Environmental Quality - Table S2

| Number | Pollutant | CAS. No | RGV (mg/kg) | C_2_ | C_3_ | C_4_ | C_5_ |
| --- | --- | --- | --- | --- | --- | --- | --- |
| 1 | Antimony | 7440-36-0 | 3.10E+01 | 0 | 1 | 1 | 1 |
| 2 | Arsenic | 7440-38-2 | 1.20E+01 | 0 | 0 | -1 | 1 |
| 3 | Barium | 7440-39-3 | 5.50E+03 | 1 | 1 | 1 | 1 |
| 4 | Beryllium | 7440-41-7 | 1.60E+02 | 1 | 1 | 1 | 1 |
| 5 | Cadmium | 7440-43-9 | 3.90E+01 | 1 | 1 | 1 | 1 |
| 6 | Chromium (III) | 16065-83-1 | 1.20E+05 | 3 | 2 | 1 | 1 |
| 7 | Chromium (VI) | 18540-29-9 | 2.30E+02 | 1 | 1 | 1 | 1 |
| 8 | Cobalt | 7440-48-4 | 4.70E+03 | 2 | 2 | 1 | 1 |
| 9 | Copper | 7440-50-8 | 3.10E+03 | 1 | 1 | 1 | 1 |
| 10 | Lead | 7439-92-1 | 4.00E+02 | 0 | 1 | 1 | 1 |
| 12 | Mercury | 7439-97-6 | 2.30E+01 | 1 | 1 | 1 | 1 |
| 14 | Nickel | 7440-02-0 | 1.60E+03 | 1 | 1 | 1 | 1 |
| 15 | Selenium | 7782-49-2 | 3.90E+02 | 1 | 1 | 1 | 1 |
| 16 | Silver | 7440-22-4 | 3.90E+02 | 1 | 1 | 1 | 1 |
| 17 | Thallium | 7440-28-0 | 5.50E+00 | 1 | 1 | 1 | 1 |
| 19 | Vanadium | 7440-62-2 | 5.50E+02 | 1 | 1 | 1 | 1 |
| 20 | Zinc | 7440-66-6 | 2.30E+04 | 2 | 2 | 1 | 1 |
| 21 | Cyanide | 57-12-5 | 1.50E+03 | 2 | 2 | 1 | 1 |
| 23 | Dichloromethane | 75-09-2 | 1.90E+01 | 0 | 0 | 1 | 1 |
| 24 | Trichloromethane | 67-66-3 | 4.40E-01 | 0 | 0 | -1 | -1 |
| 25 | Tetrachloromethane | 56-23-5 | 5.30E-01 | 0 | 0 | -1 | -1 |
| 26 | Bromoform | 75-25-2 | 4.80E+01 | 0 | 0 | -1 | -1 |
| 27 | Bromomethane | 74-83-9 | 4.30E+00 | 0 | 0 | -1 | -1 |
| 28 | 1,2-Dichloroethane | 107-06-2 | 8.20E-01 | 0 | 0 | -1 | -1 |
| 29 | 1,1,1-Trichloroethane | 71-55-6 | 8.20E+02 | 0 | 1 | 1 | 1 |
| 30 | 1,1,2-Trichloroethane | 79-00-5 | 1.90E+00 | 0 | 0 | -1 | -1 |
| 31 | Chloroethene | 75-01-4 | 2.40E-01 | 0 | 0 | 1 | -1 |
| 32 | 1,1-Dichloroethene | 75-35-4 | 1.30E+02 | 1 | 1 | 1 | 1 |
| 33 | Trichloroethene | 79-01-6 | 1.00E-01 | -1 | -1 | -1 | -1 |
| 34 | Tetrachloroethene | 127-18-4 | 8.30E+00 | 1 | 1 | 1 | 1 |
| 35 | Benzene | 71-43-2 | 1.50E+00 | 1 | 0 | 1 | 1 |
| 36 | Toluene | 108-88-3 | 6.80E+02 | 1 | 1 | 1 | 1 |
| 37 | Chlorobenzene | 108-90-7 | 1.70E+02 | 1 | 1 | 1 | 1 |
| 38 | 1,2-Dichlorobenzene | 95-50-1 | 9.90E+02 | 1 | 1 | 1 | 1 |
| 39 | 1,3-Dichlorobenzene | 541-73-1 | 2.10E+01 | 0 | 0 | -1 | 1 |
| 40 | 1,4-Dichlorobenzene | 106-46-7 | 6.70E+00 | 0 | 0 | -1 | -1 |
| 41 | 1,2,4Trichlorobenzene | 120-82-1 | 6.60E+02 | 2 | 2 | 1 | 1 |
| 42 | Hexachlorobenzene | 118-74-1 | 3.40E-01 | 0 | 0 | -1 | -1 |
| 43 | Nitrobenzene | 98-95-3 | 2.20E+01 | 0 | 0 | 1 | 1 |
| 44 | Ethylbenzene | 100-41-4 | 1.60E+03 | 2 | 2 | 1 | 1 |
| 45 | Styrene | 100-42-5 | 5.00E+03 | 2 | 2 | 1 | 1 |
| 46 | Xylenes | 1330-20-7 | 1.80E+02 | 0 | 0 | 1 | 1 |
| 48 | Phenol | 108-95-2 | 1.30E+04 | 2 | 2 | 1 | 1 |
| 49 | 2-Chlorophenol | 95-57-8 | 1.50E+02 | 1 | 1 | 1 | 1 |
| 50 | 2,4-Dichlorophenol | 120-83-2 | 1.60E+02 | 1 | 1 | 1 | 1 |
| 51 | 2,4,5-Trichlorophenol | 95-95-4 | 5.30E+03 | 1 | 2 | 1 | 1 |
| 52 | 2,4,6-Trichlorophenol | 88-06-2 | 4.00E+01 | 1 | 1 | 1 | 1 |
| 53 | 2,3,4,6-Tetrahlorophenol | 58-90-2 | 1.40E+03 | 1 | 1 | 1 | 1 |
| 54 | Pentachlorophenol | 87-86-5 | 2.80E+00 | 0 | 0 | -1 | -1 |
| 55 | Benz(a)anthracene | 56-55-3 | 6.20E-01 | 0 | 0 | -1 | -1 |
| 56 | Benzo(a)pyrene | 50-32-8 | 3.30E-01 | 0 | 0 | -1 | -1 |
| 57 | Benzo(b)fluoranthene | 205-99-2 | 6.20E-01 | 0 | 0 | -1 | -1 |
| 58 | Benzo(k)fluoranthene | 207-08-9 | 6.20E+00 | 0 | 0 | -1 | -1 |
| 59 | Chrysene | 218-01-9 | 6.20E+02 | 2 | 2 | 1 | 1 |
| 60 | Dibena(a,h)anthracene | 53-70-3 | 3.30E-01 | 0 | 0 | -1 | -1 |
| 61 | Indeno(1,2,3-c,d)pyrebe | 193-39-5 | 6.20E-01 | 0 | -1 | -1 | -1 |
| 62 | Naphthalene | 91-20-3 | 6.20E+01 | 1 | 2 | 1 | 1 |
| 63 | Acenaphehene | 83-32-9 | 3.70E+03 | 2 | 2 | 1 | 1 |
| 64 | Acenaphthylene | 208-96-8 | 3.50E+03 | 2 | 2 | 1 | 1 |
| 65 | Anthracene | 120-12-7 | 2.20E+04 | 3 | 2 | 1 | 1 |
| 67 | Flouranthene | 206-44-0 | 2.20E+03 | 3 | 2 | 1 | 1 |
| 68 | Flouorene | 86-73-7 | 2.80E+03 | 2 | 2 | 1 | 1 |
| 69 | Phenanthrene | 85-01-8 | 2.10E+04 | 3 | 3 | 1 | 1 |
| 70 | Pyrene | 129-00-0 | 2.30E+03 | 1 | 1 | 1 | 1 |
| 71 | Aldrin | 309-00-2 | 2.80E-02 | 0 | -1 | -1 | -1 |
| 72 | Chlordane | 57-74-9 | 1.60E+00 | 0 | 0 | -1 | -1 |
| 73 | DDT | 50-29-3 | 1.70E+00 | 0 | 0 | -1 | -1 |
| 74 | Dieldrin | 60-57-1 | 3.00E-02 | 0 | -1 | -1 | -1 |
| 75 | Endosulfan | 115-29-7 | 3.40E+02 | 1 | 2 | 1 | 1 |
| 76 | Endrin | 72-20-8 | 1.80E+01 | 1 | 1 | 1 | 1 |
| 77 | Heptachlor | 76-44-8 | 1.60E-02 | -1 | -1 | -1 | -1 |
| 78 | Lindane | 58-89-9 | 3.90E-01 | 0 | 0 | -1 | -1 |
| 79 | Toxaphene | 8001-35-2 | 4.40E-01 | 0 | 0 | -1 | -1 |
| 92 | Total PCB | 1336-36-3 | 2.10E-01 | 0 | 0 | -1 | -1 |
| 95 | Methyl ethyl ketone | 78-93-3 | 5.90E+03 | 0 | 1 | -1 | 1 |
| 96 | Methyl isobutyl ketone | 108-10-1 | 4.50E+03 | 0 | 1 | 1 | 1 |
| 98 | DEHP | 117-81-7 | 3.50E+01 | 0 | 0 | -1 | -1 |
| 99 | Hexachlorobutadine | 87-68-3 | 4.50E+00 | 0 | 0 | -1 | 1 |
| 100 | Methyl tert-butyl ether | 1634-04-4 | 6.50E+03 | 2 | 2 | 1 | 1 |
|  |  | Overall | C1 | C2 | C3 | C4 | C5 |
|  |  | Scores | 79 | 0.76 | 0.78 | 21 | 27 |
|  |  |  |  |  | No. >0 | 50 | 53 |
|  |  |  |  |  | No. <0 | 29 | 26 |

Overall, Louisiana’s RGVs are higher than the test statistics by more than half order of magnitude.

**Table S18** Maine Dept. of Environmental Protection

| Number | Pollutant | CAS. No | RGV (mg/kg) | C_2_ | C_3_ | C_4_ | C_5_ |
| --- | --- | --- | --- | --- | --- | --- | --- |
| 1 | Antimony | 7440-36-0 | 6.80E+01 | 1 | 1 | 1 | 1 |
| 2 | Arsenic | 7440-38-2 | 1.40E+00 | -1 | -1 | -1 | -1 |
| 3 | Barium | 7440-39-3 | 1.00E+04 | 1 | 1 | 1 | 1 |
| 5 | Cadmium | 7440-43-9 | 1.10E+01 | 0 | 0 | 1 | 1 |
| 6 | Chromium (III) | 16065-83-1 | 1.00E+04 | 2 | 1 | 1 | 1 |
| 7 | Chromium (VI) | 18540-29-9 | 5.10E+02 | 1 | 1 | 1 | 1 |
| 8 | Cobalt | 7440-48-4 | 5.10E+01 | 0 | 0 | 1 | -1 |
| 9 | Copper | 7440-50-8 | 2.40E+03 | 1 | 1 | 1 | 1 |
| 10 | Lead | 7439-92-1 | 3.40E+02 | 0 | 0 | 1 | 1 |
| 11 | Manganese | 7439-96-5 | 4.10E+03 | 1 | 1 | 1 | 1 |
| 13 | Molybdenum | 7439-98-7 | 8.50E+02 | 1 | 1 | 1 | 1 |
| 14 | Nickel | 7440-02-0 | 5.10E+02 | 1 | 1 | 1 | 1 |
| 15 | Selenium | 7782-49-2 | 8.50E+02 | 2 | 2 | 1 | 1 |
| 16 | Silver | 7440-22-4 | 8.50E+02 | 1 | 1 | 1 | 1 |
| 19 | Vanadium | 7440-62-2 | 1.20E+03 | 1 | 1 | 1 | 1 |
| 20 | Zinc | 7440-66-6 | 1.00E+04 | 1 | 1 | 1 | 1 |
| 21 | Cyanide | 57-12-5 | 1.00E+02 | 1 | 1 | 1 | 1 |
| 22 | Acrylonitril | 107-13-1 | 2.60E+01 | 1 | 1 | 1 | 1 |
| 23 | Dichloromethane | 75-09-2 | 1.00E+03 | 2 | 2 | 1 | 1 |
| 24 | Trichloromethane | 67-66-3 | 4.60E+02 | 3 | 3 | 1 | 1 |
| 25 | Tetrachloromethane | 56-23-5 | 2.00E+02 | 3 | 3 | 1 | 1 |
| 26 | Bromoform | 75-25-2 | 1.40E+03 | 2 | 2 | 1 | 1 |
| 27 | Bromomethane | 74-83-9 | 2.40E+02 | 2 | 2 | 1 | 1 |
| 28 | 1,2-Dichloroethane | 107-06-2 | 1.60E+02 | 2 | 2 | 1 | 1 |
| 29 | 1,1,1-Trichloroethane | 71-55-6 | 1.00E+04 | 2 | 2 | 1 | 1 |
| 30 | 1,1,2-Trichloroethane | 79-00-5 | 2.50E+02 | 2 | 2 | 1 | 1 |
| 31 | Chloroethene | 75-01-4 | 4.80E-01 | 1 | 0 | 1 | 1 |
| 32 | 1,1-Dichloroethene | 75-35-4 | 8.50E+03 | 3 | 3 | 1 | 1 |
| 33 | Trichloroethene | 79-01-6 | 8.50E+01 | 1 | 2 | 1 | 1 |
| 34 | Tetrachloroethene | 127-18-4 | 1.00E+03 | 3 | 3 | 1 | 1 |
| 35 | Benzene | 71-43-2 | 8.50E+01 | 2 | 2 | 1 | 1 |
| 36 | Toluene | 108-88-3 | 1.00E+04 | 2 | 2 | 1 | 1 |
| 37 | Chlorobenzene | 108-90-7 | 3.40E+03 | 2 | 2 | 1 | 1 |
| 38 | 1,2-Dichlorobenzene | 95-50-1 | 5.10E+03 | 2 | 2 | 1 | 1 |
| 39 | 1,3-Dichlorobenzene | 541-73-1 | 3.40E+01 | 0 | 0 | 1 | 1 |
| 40 | 1,4-Dichlorobenzene | 106-46-7 | 2.60E+03 | 3 | 3 | 1 | 1 |
| 41 | 1,2,4Trichlorobenzene | 120-82-1 | 4.90E+02 | 2 | 2 | 1 | 1 |
| 42 | Hexachlorobenzene | 118-74-1 | 6.80E+00 | 1 | 1 | 1 | 1 |
| 44 | Ethylbenzene | 100-41-4 | 1.30E+03 | 2 | 2 | 1 | 1 |
| 45 | Styrene | 100-42-5 | 1.00E+04 | 2 | 2 | 1 | 1 |
| 46 | Xylenes | 1330-20-7 | 1.00E+04 | 2 | 2 | 1 | 1 |
| 47 | Cresol | 1319-77-3 | 6.70E+02 | 1 | 1 | 1 | 1 |
| 48 | Phenol | 108-95-2 | 1.00E+04 | 2 | 2 | 1 | 1 |
| 49 | 2-Chlorophenol | 95-57-8 | 8.50E+02 | 2 | 2 | 1 | 1 |
| 50 | 2,4-Dichlorophenol | 120-83-2 | 4.00E+02 | 2 | 2 | 1 | 1 |
| 51 | 2,4,5-Trichlorophenol | 95-95-4 | 1.00E+04 | 1 | 2 | 1 | 1 |
| 52 | 2,4,6-Trichlorophenol | 88-06-2 | 1.30E+02 | 1 | 1 | 1 | 1 |
| 54 | Pentachlorophenol | 87-86-5 | 2.00E+01 | 1 | 1 | 1 | 1 |
| 55 | Benz(a)anthracene | 56-55-3 | 2.60E+00 | 0 | 0 | 1 | -1 |
| 56 | Benzo(a)pyrene | 50-32-8 | 2.60E-01 | 0 | 0 | -1 | -1 |
| 57 | Benzo(b)fluoranthene | 205-99-2 | 2.60E+00 | 0 | 0 | 1 | -1 |
| 58 | Benzo(k)fluoranthene | 207-08-9 | 2.60E+01 | 1 | 1 | 1 | 1 |
| 59 | Chrysene | 218-01-9 | 2.60E+02 | 1 | 1 | 1 | 1 |
| 60 | Dibena(a,h)anthracene | 53-70-3 | 2.60E-01 | 0 | 0 | -1 | -1 |
| 61 | Indeno(1,2,3-c,d)pyrebe | 193-39-5 | 2.60E+00 | 0 | 0 | -1 | -1 |
| 62 | Naphthalene | 91-20-3 | 2.50E+03 | 2 | 3 | 1 | 1 |
| 63 | Acenaphehene | 83-32-9 | 7.50E+03 | 2 | 2 | 1 | 1 |
| 64 | Acenaphthylene | 208-96-8 | 7.50E+03 | 2 | 2 | 1 | 1 |
| 65 | Anthracene | 120-12-7 | 1.00E+04 | 2 | 2 | 1 | 1 |
| 66 | Benzo(g,h,i) perylene | 191-24-2 | 3.70E+03 | 2 | 2 | 1 | 1 |
| 67 | Flouranthene | 206-44-0 | 5.00E+03 | 3 | 2 | 1 | 1 |
| 68 | Flouorene | 86-73-7 | 5.00E+03 | 2 | 2 | 1 | 1 |
| 69 | Phenanthrene | 85-01-8 | 3.70E+03 | 2 | 2 | 1 | 1 |
| 70 | Pyrene | 129-00-0 | 3.70E+03 | 2 | 2 | 1 | 1 |
| 71 | Aldrin | 309-00-2 | 6.40E-01 | 1 | 1 | 1 | 1 |
| 72 | Chlordane | 57-74-9 | 3.60E+01 | 1 | 1 | 1 | 1 |
| 73 | DDT | 50-29-3 | 3.80E+01 | 2 | 1 | 1 | 1 |
| 74 | Dieldrin | 60-57-1 | 6.80E-01 | 1 | 1 | 1 | 1 |
| 75 | Endosulfan | 115-29-7 | 8.00E+02 | 2 | 2 | 1 | 1 |
| 76 | Endrin | 72-20-8 | 4.00E+01 | 1 | 1 | 1 | 1 |
| 77 | Heptachlor | 76-44-8 | 1.30E+00 | 1 | 1 | 1 | 1 |
| 78 | Lindane | 58-89-9 | 6.10E-01 | 0 | 0 | 1 | 1 |
| 81 | Atrazine | 1912-24-9 | 4.70E+01 | 1 | 1 | 1 | 1 |
| 87 | Malathion | 121-75-5 | 2.70E+03 | 1 | 1 | 1 | 1 |
| 92 | Total PCB | 1336-36-3 | 2.40E+00 | 1 | 1 | 1 | 1 |
| 94 | Total PCDD/PCDF | 1746-01-6 | 1.00E-04 | 0 | 0 | 1 | -1 |
| 95 | Methyl ethyl ketone | 78-93-3 | 1.00E+04 | 0 | 1 | 1 | 1 |
| 96 | Methyl isobutyl ketone | 108-10-1 | 1.00E+04 | 1 | 1 | 1 | 1 |
| 97 | Dibutyl phthalate | 84-74-2 | 1.00E+04 | 0 | 1 | 1 | 1 |
| 98 | DEHP | 117-81-7 | 7.70E+02 | 1 | 1 | 1 | 1 |
| 99 | Hexachlorobutadine | 87-68-3 | 1.30E+02 | 2 | 2 | 1 | 1 |
| 100 | Methyl tert-butyl ether | 1634-04-4 | 5.10E+03 | 2 | 2 | 1 | 1 |
|  |  | Overall | C1 | C2 | C3 | C4 | C5 |
|  |  | Scores | 82 | 1.34 | 1.35 | 74 | 66 |
|  |  |  |  |  | No. >0 | 78 | 74 |
|  |  |  |  |  | No. <0 | 4 | 8 |

**Table S19** Maryland Dept. of the Environment

| Number | Pollutant | CAS. No | RGV (mg/kg) | C_2_ | C_3_ | C_4_ | C_5_ |
| --- | --- | --- | --- | --- | --- | --- | --- |
| 1 | Antimony | 7440-36-0 | 3.10E+00 | -1 | 0 | -1 | -1 |
| 2 | Arsenic | 7440-38-2 | 4.30E-01 | -1 | -1 | -1 | -1 |
| 3 | Barium | 7440-39-3 | 1.60E+03 | 1 | 0 | 1 | 1 |
| 4 | Beryllium | 7440-41-7 | 1.60E+01 | 0 | 0 | -1 | -1 |
| 5 | Cadmium | 7440-43-9 | 3.90E+00 | 0 | 0 | -1 | -1 |
| 6 | Chromium (III) | 16065-83-1 | 1.20E+04 | 2 | 1 | 1 | 1 |
| 7 | Chromium (VI) | 18540-29-9 | 2.30E+01 | 0 | 0 | -1 | -1 |
| 9 | Copper | 7440-50-8 | 3.10E+02 | 0 | 0 | 1 | 1 |
| 10 | Lead | 7439-92-1 | 4.00E+02 | 0 | 1 | 1 | 1 |
| 11 | Manganese | 7439-96-5 | 1.60E+02 | -1 | -1 | -1 | -1 |
| 12 | Mercury | 7439-97-6 | 2.30E+00 | 0 | 0 | -1 | -1 |
| 14 | Nickel | 7440-02-0 | 1.60E+02 | 0 | 0 | 1 | -1 |
| 15 | Selenium | 7782-49-2 | 3.90E+01 | 0 | 0 | 1 | 1 |
| 16 | Silver | 7440-22-4 | 3.90E+01 | 0 | 0 | -1 | -1 |
| 17 | Thallium | 7440-28-0 | 5.50E-01 | 0 | 0 | -1 | -1 |
| 18 | Tin | 7440-31-5 | 4.70E+03 | 2 | 1 | 1 | 1 |
| 19 | Vanadium | 7440-62-2 | 7.80E+00 | -1 | -1 | -1 | -1 |
| 20 | Zinc | 7440-66-6 | 2.30E+03 | 1 | 1 | 1 | 1 |
| 21 | Cyanide | 57-12-5 | 1.60E+02 | 1 | 1 | 1 | 1 |
| 23 | Dichloromethane | 75-09-2 | 8.50E+01 | 1 | 1 | 1 | 1 |
| 24 | Trichloromethane | 67-66-3 | 7.80E+01 | 2 | 2 | 1 | 1 |
| 25 | Tetrachloromethane | 56-23-5 | 4.90E+00 | 1 | 1 | 1 | 1 |
| 26 | Bromoform | 75-25-2 | 8.10E+01 | 0 | 0 | 1 | 1 |
| 27 | Bromomethane | 74-83-9 | 1.10E+01 | 0 | 0 | 1 | 1 |
| 28 | 1,2-Dichloroethane | 107-06-2 | 7.00E+00 | 1 | 1 | 1 | 1 |
| 29 | 1,1,1-Trichloroethane | 71-55-6 | 1.60E+04 | 2 | 2 | 1 | 1 |
| 30 | 1,1,2-Trichloroethane | 79-00-5 | 1.10E+01 | 1 | 1 | 1 | 1 |
| 31 | Chloroethene | 75-01-4 | 9.00E-02 | 0 | 0 | -1 | -1 |
| 34 | Tetrachloroethene | 127-18-4 | 1.20E+00 | 0 | 0 | -1 | -1 |
| 35 | Benzene | 71-43-2 | 1.20E+01 | 1 | 1 | 1 | 1 |
| 36 | Toluene | 108-88-3 | 6.30E+02 | 1 | 1 | 1 | 1 |
| 37 | Chlorobenzene | 108-90-7 | 1.60E+02 | 1 | 1 | 1 | 1 |
| 38 | 1,2-Dichlorobenzene | 95-50-1 | 7.00E+02 | 1 | 1 | 1 | 1 |
| 39 | 1,3-Dichlorobenzene | 541-73-1 | 2.30E+01 | 0 | 0 | -1 | 1 |
| 40 | 1,4-Dichlorobenzene | 106-46-7 | 2.70E+01 | 1 | 1 | 1 | 1 |
| 42 | Hexachlorobenzene | 118-74-1 | 4.00E-01 | 0 | 0 | -1 | -1 |
| 43 | Nitrobenzene | 98-95-3 | 3.90E+00 | 0 | 0 | -1 | -1 |
| 44 | Ethylbenzene | 100-41-4 | 7.80E+02 | 2 | 2 | 1 | 1 |
| 45 | Styrene | 100-42-5 | 1.60E+03 | 1 | 2 | 1 | 1 |
| 46 | Xylenes | 1330-20-7 | 1.60E+03 | 1 | 1 | 1 | 1 |
| 47 | Cresol | 1319-77-3 | 3.90E+01 | 0 | 0 | -1 | -1 |
| 48 | Phenol | 108-95-2 | 2.30E+03 | 1 | 1 | 1 | 1 |
| 49 | 2-Chlorophenol | 95-57-8 | 3.90E+01 | 0 | 0 | -1 | 1 |
| 50 | 2,4-Dichlorophenol | 120-83-2 | 2.30E+01 | 0 | 1 | 1 | 1 |
| 51 | 2,4,5-Trichlorophenol | 95-95-4 | 7.80E+02 | 0 | 1 | 1 | 1 |
| 52 | 2,4,6-Trichlorophenol | 88-06-2 | 5.80E+01 | 1 | 1 | 1 | 1 |
| 54 | Pentachlorophenol | 87-86-5 | 5.30E+00 | 0 | 0 | 1 | 1 |
| 55 | Benz(a)anthracene | 56-55-3 | 2.20E-01 | -1 | -1 | -1 | -1 |
| 56 | Benzo(a)pyrene | 50-32-8 | 2.20E-02 | -1 | -1 | -1 | -1 |
| 57 | Benzo(b)fluoranthene | 205-99-2 | 2.20E-01 | -1 | -1 | -1 | -1 |
| 58 | Benzo(k)fluoranthene | 207-08-9 | 2.20E+00 | 0 | 0 | -1 | -1 |
| 59 | Chrysene | 218-01-9 | 2.20E+01 | 0 | 0 | -1 | -1 |
| 60 | Dibena(a,h)anthracene | 53-70-3 | 2.20E-02 | -1 | -1 | -1 | -1 |
| 61 | Indeno(1,2,3-c,d)pyrebe | 193-39-5 | 2.20E-01 | -1 | -1 | -1 | -1 |
| 62 | Naphthalene | 91-20-3 | 1.60E+02 | 1 | 2 | 1 | 1 |
| 63 | Acenaphehene | 83-32-9 | 4.70E+02 | 1 | 1 | 1 | 1 |
| 64 | Acenaphthylene | 208-96-8 | 4.70E+02 | 1 | 1 | 1 | 1 |
| 65 | Anthracene | 120-12-7 | 2.30E+03 | 2 | 1 | 1 | 1 |
| 66 | Benzo(g,h,i) perylene | 191-24-2 | 2.30E+02 | 1 | 1 | 1 | 1 |
| 67 | Flouranthene | 206-44-0 | 3.10E+02 | 2 | 1 | 1 | 1 |
| 68 | Flouorene | 86-73-7 | 3.10E+02 | 1 | 1 | 1 | 1 |
| 69 | Phenanthrene | 85-01-8 | 2.30E+03 | 2 | 2 | 1 | 1 |
| 70 | Pyrene | 129-00-0 | 2.30E+02 | 0 | 0 | 1 | 1 |
| 71 | Aldrin | 309-00-2 | 3.80E-02 | 0 | -1 | -1 | -1 |
| 72 | Chlordane | 57-74-9 | 1.80E+00 | 0 | 0 | -1 | -1 |
| 73 | DDT | 50-29-3 | 1.90E+00 | 0 | 0 | -1 | -1 |
| 74 | Dieldrin | 60-57-1 | 4.00E-02 | 0 | -1 | -1 | -1 |
| 75 | Endosulfan | 115-29-7 | 4.70E+01 | 0 | 1 | 1 | 1 |
| 76 | Endrin | 72-20-8 | 2.30E+00 | 0 | 0 | -1 | -1 |
| 77 | Heptachlor | 76-44-8 | 1.40E-01 | 0 | 0 | -1 | -1 |
| 79 | Toxaphene | 8001-35-2 | 5.80E-01 | 0 | 0 | -1 | -1 |
| 80 | 2,4-D | 94-75-7 | 7.80E+01 | -1 | 0 | -1 | -1 |
| 81 | Atrazine | 1912-24-9 | 2.90E+00 | 0 | 0 | 1 | -1 |
| 86 | Gylphosate | 1071-83-6 | 7.80E+02 | -1 | 0 | -1 | -1 |
| 90 | Simazine | 122-34-9 | 5.30E+00 | 0 | 0 | 1 | -1 |
| 93 | PCB 118 | 31508-00-6 | 3.20E-01 | 0 | 0 | 1 | 1 |
| 95 | Methyl ethyl ketone | 78-93-3 | 4.70E+03 | 0 | 1 | -1 | 1 |
| 97 | Dibutyl phthalate | 84-74-2 | 7.80E+02 | 0 | 0 | -1 | -1 |
| 98 | DEHP | 117-81-7 | 4.60E+01 | 0 | 0 | -1 | -1 |
| 99 | Hexachlorobutadine | 87-68-3 | 8.20E+00 | 0 | 1 | 1 | 1 |
| 100 | Methyl tert-butyl ether | 1634-04-4 | 1.60E+02 | 1 | 1 | 1 | 1 |
|  |  | Overall | C1 | C2 | C3 | C4 | C5 |
|  |  | Scores | 81 | 0.33 | 0.38 | 9 | 9 |
|  |  |  |  |  | No. >0 | 45 | 45 |
|  |  |  |  |  | No. <0 | 36 | 36 |

**Table S20a** Massachusetts Dept. of Environmental Protection M1-S1-GW3

| Number | Pollutant | CAS. No | RGV (mg/kg) | C_2_ | C_3_ | C_4_ | C_5_ |
| --- | --- | --- | --- | --- | --- | --- | --- |
| 1 | Antimony | 7440-36-0 | 2.00E+01 | 0 | 0 | -1 | 1 |
| 2 | Arsenic | 7440-38-2 | 2.00E+01 | 0 | 0 | -1 | 1 |
| 3 | Barium | 7440-39-3 | 1.00E+03 | 0 | 0 | 1 | -1 |
| 4 | Beryllium | 7440-41-7 | 9.00E+01 | 1 | 1 | 1 | 1 |
| 5 | Cadmium | 7440-43-9 | 7.00E+01 | 1 | 1 | 1 | 1 |
| 6 | Chromium (III) | 16065-83-1 | 1.00E+03 | 1 | 0 | 1 | 1 |
| 7 | Chromium (VI) | 18540-29-9 | 1.00E+02 | 0 | 1 | -1 | 1 |
| 10 | Lead | 7439-92-1 | 2.00E+02 | 0 | 0 | -1 | 1 |
| 12 | Mercury | 7439-97-6 | 2.00E+01 | 1 | 1 | 1 | 1 |
| 14 | Nickel | 7440-02-0 | 6.00E+02 | 1 | 1 | 1 | 1 |
| 15 | Selenium | 7782-49-2 | 4.00E+02 | 1 | 1 | 1 | 1 |
| 16 | Silver | 7440-22-4 | 1.00E+02 | 1 | 1 | 1 | 1 |
| 17 | Thallium | 7440-28-0 | 8.00E+00 | 1 | 1 | 1 | 1 |
| 19 | Vanadium | 7440-62-2 | 4.00E+02 | 1 | 1 | 1 | 1 |
| 20 | Zinc | 7440-66-6 | 1.00E+03 | 0 | 0 | 1 | -1 |
| 21 | Cyanide | 57-12-5 | 3.00E+01 | 0 | 0 | -1 | -1 |
| 23 | Dichloromethane | 75-09-2 | 4.00E+02 | 2 | 2 | 1 | 1 |
| 24 | Trichloromethane | 67-66-3 | 5.00E+02 | 3 | 3 | 1 | 1 |
| 25 | Tetrachloromethane | 56-23-5 | 3.00E+01 | 2 | 2 | 1 | 1 |
| 26 | Bromoform | 75-25-2 | 3.00E+02 | 1 | 1 | 1 | 1 |
| 27 | Bromomethane | 74-83-9 | 3.00E+01 | 1 | 1 | 1 | 1 |
| 28 | 1,2-Dichloroethane | 107-06-2 | 2.00E+01 | 1 | 1 | 1 | 1 |
| 29 | 1,1,1-Trichloroethane | 71-55-6 | 5.00E+02 | 0 | 1 | -1 | 1 |
| 30 | 1,1,2-Trichloroethane | 79-00-5 | 4.00E+01 | 1 | 1 | 1 | 1 |
| 31 | Chloroethene | 75-01-4 | 1.00E+00 | 1 | 1 | 1 | 1 |
| 32 | 1,1-Dichloroethene | 75-35-4 | 5.00E+02 | 2 | 2 | 1 | 1 |
| 33 | Trichloroethene | 79-01-6 | 3.00E+01 | 1 | 1 | 1 | 1 |
| 34 | Tetrachloroethene | 127-18-4 | 3.00E+01 | 1 | 1 | 1 | 1 |
| 35 | Benzene | 71-43-2 | 4.00E+01 | 2 | 2 | 1 | 1 |
| 36 | Toluene | 108-88-3 | 5.00E+02 | 1 | 1 | 1 | 1 |
| 37 | Chlorobenzene | 108-90-7 | 1.00E+02 | 0 | 1 | 1 | 1 |
| 38 | 1,2-Dichlorobenzene | 95-50-1 | 3.00E+02 | 0 | 1 | 1 | 1 |
| 39 | 1,3-Dichlorobenzene | 541-73-1 | 1.00E+02 | 1 | 1 | 1 | 1 |
| 40 | 1,4-Dichlorobenzene | 106-46-7 | 8.00E+01 | 1 | 1 | 1 | 1 |
| 41 | 1,2,4Trichlorobenzene | 120-82-1 | 7.00E+02 | 2 | 2 | 1 | 1 |
| 42 | Hexachlorobenzene | 118-74-1 | 7.00E-01 | 0 | 0 | 1 | -1 |
| 44 | Ethylbenzene | 100-41-4 | 5.00E+02 | 2 | 1 | 1 | 1 |
| 45 | Styrene | 100-42-5 | 7.00E+01 | 0 | 0 | -1 | -1 |
| 46 | Xylenes | 1330-20-7 | 5.00E+02 | 1 | 1 | 1 | 1 |
| 48 | Phenol | 108-95-2 | 2.00E+01 | -1 | -1 | -1 | -1 |
| 49 | 2-Chlorophenol | 95-57-8 | 1.00E+02 | 1 | 1 | 1 | 1 |
| 50 | 2,4-Dichlorophenol | 120-83-2 | 4.00E+01 | 1 | 1 | 1 | 1 |
| 51 | 2,4,5-Trichlorophenol | 95-95-4 | 6.00E+02 | 0 | 1 | -1 | 1 |
| 52 | 2,4,6-Trichlorophenol | 88-06-2 | 2.00E+01 | 0 | 0 | 1 | 1 |
| 54 | Pentachlorophenol | 87-86-5 | 3.00E+00 | 0 | 0 | -1 | -1 |
| 55 | Benz(a)anthracene | 56-55-3 | 7.00E+00 | 1 | 1 | 1 | 1 |
| 56 | Benzo(a)pyrene | 50-32-8 | 2.00E+00 | 1 | 1 | 1 | 1 |
| 57 | Benzo(b)fluoranthene | 205-99-2 | 7.00E+00 | 1 | 1 | 1 | 1 |
| 58 | Benzo(k)fluoranthene | 207-08-9 | 7.00E+01 | 1 | 1 | 1 | 1 |
| 59 | Chrysene | 218-01-9 | 7.00E+01 | 1 | 1 | 1 | 1 |
| 60 | Dibena(a,h)anthracene | 53-70-3 | 7.00E-01 | 0 | 0 | 1 | -1 |
| 61 | Indeno(1,2,3-c,d)pyrebe | 193-39-5 | 7.00E+00 | 1 | 1 | 1 | 1 |
| 62 | Naphthalene | 91-20-3 | 5.00E+02 | 2 | 2 | 1 | 1 |
| 63 | Acenaphehene | 83-32-9 | 1.00E+03 | 1 | 1 | 1 | 1 |
| 64 | Acenaphthylene | 208-96-8 | 1.00E+01 | -1 | -1 | -1 | -1 |
| 65 | Anthracene | 120-12-7 | 1.00E+03 | 1 | 1 | 1 | 1 |
| 66 | Benzo(g,h,i) perylene | 191-24-2 | 1.00E+03 | 2 | 1 | 1 | 1 |
| 67 | Flouranthene | 206-44-0 | 1.00E+03 | 2 | 1 | 1 | 1 |
| 68 | Flouorene | 86-73-7 | 1.00E+03 | 1 | 1 | 1 | 1 |
| 69 | Phenanthrene | 85-01-8 | 5.00E+02 | 1 | 1 | 1 | 1 |
| 70 | Pyrene | 129-00-0 | 1.00E+03 | 1 | 1 | 1 | 1 |
| 71 | Aldrin | 309-00-2 | 8.00E-02 | 0 | 0 | -1 | -1 |
| 72 | Chlordane | 57-74-9 | 5.00E+00 | 0 | 0 | 1 | 1 |
| 73 | DDT | 50-29-3 | 6.00E+00 | 1 | 0 | 1 | 1 |
| 74 | Dieldrin | 60-57-1 | 8.00E-02 | 0 | 0 | -1 | -1 |
| 75 | Endosulfan | 115-29-7 | 1.00E+00 | -1 | -1 | -1 | -1 |
| 76 | Endrin | 72-20-8 | 1.00E+01 | 1 | 1 | 1 | 1 |
| 77 | Heptachlor | 76-44-8 | 3.00E-01 | 0 | 0 | 1 | -1 |
| 78 | Lindane | 58-89-9 | 5.00E-01 | 0 | 0 | -1 | -1 |
| 92 | Total PCB | 1336-36-3 | 1.00E+00 | 0 | 0 | 1 | 1 |
| 94 | Total PCDD/PCDF | 1746-01-6 | 2.00E-05 | 0 | 0 | -1 | -1 |
| 95 | Methyl ethyl ketone | 78-93-3 | 4.00E+02 | -1 | 0 | -1 | -1 |
| 96 | Methyl isobutyl ketone | 108-10-1 | 4.00E+02 | -1 | 0 | -1 | -1 |
| 98 | DEHP | 117-81-7 | 9.00E+01 | 0 | 0 | 1 | -1 |
| 99 | Hexachlorobutadine | 87-68-3 | 3.00E+01 | 1 | 1 | 1 | 1 |
| 100 | Methyl tert-butyl ether | 1634-04-4 | 1.00E+02 | 0 | 1 | 1 | 1 |
|  |  | Overall | C1 | C2 | C3 | C4 | C5 |
|  |  | Scores | 76 | 0.67 | 0.72 | 40 | 40 |
|  |  |  |  |  | No. >0 | 58 | 58 |
|  |  |  |  |  | No. <0 | 18 | 18 |

**Table S20b** Massachusetts M1-S2-GW3

| Number | Pollutant | CAS. No | RGV (mg/kg) | C_2_ | C_3_ | C_4_ | C_5_ |
| --- | --- | --- | --- | --- | --- | --- | --- |
| 1 | Antimony | 7440-36-0 | 3.00E+01 | 0 | 1 | 1 | 1 |
| 2 | Arsenic | 7440-38-2 | 2.00E+01 | 0 | 0 | -1 | 1 |
| 3 | Barium | 7440-39-3 | 3.00E+03 | 1 | 1 | 1 | 1 |
| 4 | Beryllium | 7440-41-7 | 2.00E+02 | 1 | 1 | 1 | 1 |
| 5 | Cadmium | 7440-43-9 | 1.00E+02 | 1 | 1 | 1 | 1 |
| 6 | Chromium (III) | 16065-83-1 | 3.00E+03 | 1 | 1 | 1 | 1 |
| 7 | Chromium (VI) | 18540-29-9 | 2.00E+02 | 1 | 1 | 1 | 1 |
| 10 | Lead | 7439-92-1 | 6.00E+02 | 1 | 1 | 1 | 1 |
| 12 | Mercury | 7439-97-6 | 3.00E+01 | 1 | 1 | 1 | 1 |
| 14 | Nickel | 7440-02-0 | 1.00E+03 | 1 | 1 | 1 | 1 |
| 15 | Selenium | 7782-49-2 | 7.00E+02 | 2 | 2 | 1 | 1 |
| 16 | Silver | 7440-22-4 | 2.00E+02 | 1 | 1 | 1 | 1 |
| 17 | Thallium | 7440-28-0 | 6.00E+01 | 2 | 2 | 1 | 1 |
| 19 | Vanadium | 7440-62-2 | 7.00E+02 | 1 | 1 | 1 | 1 |
| 20 | Zinc | 7440-66-6 | 3.00E+03 | 1 | 1 | 1 | 1 |
| 21 | Cyanide | 57-12-5 | 1.00E+02 | 1 | 1 | 1 | 1 |
| 23 | Dichloromethane | 75-09-2 | 7.00E+02 | 2 | 2 | 1 | 1 |
| 24 | Trichloromethane | 67-66-3 | 1.00E+03 | 3 | 3 | 1 | 1 |
| 25 | Tetrachloromethane | 56-23-5 | 1.00E+02 | 2 | 2 | 1 | 1 |
| 26 | Bromoform | 75-25-2 | 8.00E+02 | 1 | 1 | 1 | 1 |
| 27 | Bromomethane | 74-83-9 | 3.00E+01 | 1 | 1 | 1 | 1 |
| 28 | 1,2-Dichloroethane | 107-06-2 | 1.00E+02 | 2 | 2 | 1 | 1 |
| 29 | 1,1,1-Trichloroethane | 71-55-6 | 1.00E+03 | 1 | 1 | 1 | 1 |
| 30 | 1,1,2-Trichloroethane | 79-00-5 | 2.00E+02 | 2 | 2 | 1 | 1 |
| 31 | Chloroethene | 75-01-4 | 7.00E+00 | 2 | 2 | 1 | 1 |
| 32 | 1,1-Dichloroethene | 75-35-4 | 1.00E+03 | 2 | 2 | 1 | 1 |
| 33 | Trichloroethene | 79-01-6 | 6.00E+01 | 1 | 2 | 1 | 1 |
| 34 | Tetrachloroethene | 127-18-4 | 2.00E+02 | 2 | 2 | 1 | 1 |
| 35 | Benzene | 71-43-2 | 2.00E+02 | 3 | 3 | 1 | 1 |
| 36 | Toluene | 108-88-3 | 1.00E+03 | 1 | 1 | 1 | 1 |
| 37 | Chlorobenzene | 108-90-7 | 1.00E+02 | 0 | 1 | 1 | 1 |
| 38 | 1,2-Dichlorobenzene | 95-50-1 | 3.00E+02 | 0 | 1 | 1 | 1 |
| 39 | 1,3-Dichlorobenzene | 541-73-1 | 5.00E+02 | 1 | 2 | 1 | 1 |
| 40 | 1,4-Dichlorobenzene | 106-46-7 | 4.00E+02 | 2 | 2 | 1 | 1 |
| 41 | 1,2,4Trichlorobenzene | 120-82-1 | 3.00E+03 | 2 | 2 | 1 | 1 |
| 42 | Hexachlorobenzene | 118-74-1 | 8.00E-01 | 0 | 0 | 1 | -1 |
| 44 | Ethylbenzene | 100-41-4 | 1.00E+03 | 2 | 2 | 1 | 1 |
| 45 | Styrene | 100-42-5 | 3.00E+02 | 1 | 1 | 1 | 1 |
| 46 | Xylenes | 1330-20-7 | 1.00E+03 | 1 | 1 | 1 | 1 |
| 48 | Phenol | 108-95-2 | 2.00E+01 | -1 | -1 | -1 | -1 |
| 49 | 2-Chlorophenol | 95-57-8 | 3.00E+02 | 1 | 1 | 1 | 1 |
| 50 | 2,4-Dichlorophenol | 120-83-2 | 4.00E+01 | 1 | 1 | 1 | 1 |
| 51 | 2,4,5-Trichlorophenol | 95-95-4 | 6.00E+02 | 0 | 1 | -1 | 1 |
| 52 | 2,4,6-Trichlorophenol | 88-06-2 | 2.00E+01 | 0 | 0 | 1 | 1 |
| 54 | Pentachlorophenol | 87-86-5 | 1.00E+01 | 1 | 1 | 1 | 1 |
| 55 | Benz(a)anthracene | 56-55-3 | 4.00E+01 | 2 | 1 | 1 | 1 |
| 56 | Benzo(a)pyrene | 50-32-8 | 7.00E+00 | 1 | 1 | 1 | 1 |
| 57 | Benzo(b)fluoranthene | 205-99-2 | 4.00E+01 | 2 | 1 | 1 | 1 |
| 58 | Benzo(k)fluoranthene | 207-08-9 | 4.00E+02 | 2 | 2 | 1 | 1 |
| 59 | Chrysene | 218-01-9 | 4.00E+02 | 1 | 1 | 1 | 1 |
| 60 | Dibena(a,h)anthracene | 53-70-3 | 4.00E+00 | 1 | 1 | 1 | 1 |
| 61 | Indeno(1,2,3-c,d)pyrebe | 193-39-5 | 4.00E+01 | 1 | 1 | 1 | 1 |
| 62 | Naphthalene | 91-20-3 | 1.00E+03 | 2 | 3 | 1 | 1 |
| 63 | Acenaphehene | 83-32-9 | 3.00E+03 | 2 | 2 | 1 | 1 |
| 64 | Acenaphthylene | 208-96-8 | 1.00E+01 | -1 | -1 | -1 | -1 |
| 65 | Anthracene | 120-12-7 | 3.00E+03 | 2 | 1 | 1 | 1 |
| 66 | Benzo(g,h,i) perylene | 191-24-2 | 3.00E+03 | 2 | 2 | 1 | 1 |
| 67 | Flouranthene | 206-44-0 | 3.00E+03 | 3 | 2 | 1 | 1 |
| 68 | Flouorene | 86-73-7 | 3.00E+03 | 2 | 2 | 1 | 1 |
| 69 | Phenanthrene | 85-01-8 | 1.00E+03 | 2 | 1 | 1 | 1 |
| 70 | Pyrene | 129-00-0 | 3.00E+03 | 1 | 2 | 1 | 1 |
| 71 | Aldrin | 309-00-2 | 5.00E-01 | 1 | 1 | 1 | 1 |
| 72 | Chlordane | 57-74-9 | 3.00E+01 | 1 | 1 | 1 | 1 |
| 73 | DDT | 50-29-3 | 3.00E+01 | 1 | 1 | 1 | 1 |
| 74 | Dieldrin | 60-57-1 | 5.00E-01 | 1 | 0 | 1 | 1 |
| 75 | Endosulfan | 115-29-7 | 1.00E+00 | -1 | -1 | -1 | -1 |
| 76 | Endrin | 72-20-8 | 2.00E+01 | 1 | 1 | 1 | 1 |
| 77 | Heptachlor | 76-44-8 | 2.00E+00 | 1 | 1 | 1 | 1 |
| 78 | Lindane | 58-89-9 | 5.00E-01 | 0 | 0 | -1 | -1 |
| 92 | Total PCB | 1336-36-3 | 4.00E+00 | 1 | 1 | 1 | 1 |
| 94 | Total PCDD/PCDF | 1746-01-6 | 5.00E-05 | 0 | 0 | -1 | -1 |
| 95 | Methyl ethyl ketone | 78-93-3 | 4.00E+02 | -1 | 0 | -1 | -1 |
| 96 | Methyl isobutyl ketone | 108-10-1 | 4.00E+02 | -1 | 0 | -1 | -1 |
| 98 | DEHP | 117-81-7 | 6.00E+02 | 1 | 1 | 1 | 1 |
| 99 | Hexachlorobutadine | 87-68-3 | 1.00E+02 | 1 | 2 | 1 | 1 |
| 100 | Methyl tert-butyl ether | 1634-04-4 | 5.00E+02 | 1 | 1 | 1 | 1 |
|  |  | Overall | C1 | C2 | C3 | C4 | C5 |
|  |  | Scores | 76 | 1.09 | 1.17 | 58 | 60 |
|  |  |  |  |  | No. >0 | 67 | 68 |
|  |  |  |  |  | No. <0 | 9 | 8 |

**Table S20c** Massachusetts M1-S3-GW3

| Number | Pollutant | CAS. No | RGV (mg/kg) | C_2_ | C_3_ | C_4_ | C_5_ |
| --- | --- | --- | --- | --- | --- | --- | --- |
| 1 | Antimony | 7440-36-0 | 3.00E+01 | 0 | 1 | 1 | 1 |
| 2 | Arsenic | 7440-38-2 | 5.00E+01 | 1 | 1 | 1 | 1 |
| 3 | Barium | 7440-39-3 | 5.00E+03 | 1 | 1 | 1 | 1 |
| 4 | Beryllium | 7440-41-7 | 2.00E+02 | 1 | 1 | 1 | 1 |
| 5 | Cadmium | 7440-43-9 | 1.00E+02 | 1 | 1 | 1 | 1 |
| 6 | Chromium (III) | 16065-83-1 | 5.00E+03 | 2 | 1 | 1 | 1 |
| 7 | Chromium (VI) | 18540-29-9 | 2.00E+02 | 1 | 1 | 1 | 1 |
| 10 | Lead | 7439-92-1 | 6.00E+02 | 1 | 1 | 1 | 1 |
| 12 | Mercury | 7439-97-6 | 3.00E+01 | 1 | 1 | 1 | 1 |
| 14 | Nickel | 7440-02-0 | 1.00E+03 | 1 | 1 | 1 | 1 |
| 15 | Selenium | 7782-49-2 | 7.00E+02 | 2 | 2 | 1 | 1 |
| 16 | Silver | 7440-22-4 | 2.00E+02 | 1 | 1 | 1 | 1 |
| 17 | Thallium | 7440-28-0 | 8.00E+01 | 2 | 2 | 1 | 1 |
| 19 | Vanadium | 7440-62-2 | 7.00E+02 | 1 | 1 | 1 | 1 |
| 20 | Zinc | 7440-66-6 | 5.00E+03 | 1 | 1 | 1 | 1 |
| 21 | Cyanide | 57-12-5 | 5.00E+02 | 1 | 1 | 1 | 1 |
| 23 | Dichloromethane | 75-09-2 | 7.00E+02 | 2 | 2 | 1 | 1 |
| 24 | Trichloromethane | 67-66-3 | 1.00E+03 | 3 | 3 | 1 | 1 |
| 25 | Tetrachloromethane | 56-23-5 | 1.00E+03 | 3 | 3 | 1 | 1 |
| 26 | Bromoform | 75-25-2 | 8.00E+02 | 1 | 1 | 1 | 1 |
| 27 | Bromomethane | 74-83-9 | 3.00E+01 | 1 | 1 | 1 | 1 |
| 28 | 1,2-Dichloroethane | 107-06-2 | 3.00E+02 | 2 | 3 | 1 | 1 |
| 29 | 1,1,1-Trichloroethane | 71-55-6 | 3.00E+03 | 1 | 1 | 1 | 1 |
| 30 | 1,1,2-Trichloroethane | 79-00-5 | 5.00E+02 | 2 | 2 | 1 | 1 |
| 31 | Chloroethene | 75-01-4 | 6.00E+01 | 3 | 3 | 1 | 1 |
| 32 | 1,1-Dichloroethene | 75-35-4 | 3.00E+03 | 3 | 3 | 1 | 1 |
| 33 | Trichloroethene | 79-01-6 | 6.00E+01 | 1 | 2 | 1 | 1 |
| 34 | Tetrachloroethene | 127-18-4 | 1.00E+03 | 3 | 3 | 1 | 1 |
| 35 | Benzene | 71-43-2 | 1.00E+03 | 3 | 3 | 1 | 1 |
| 36 | Toluene | 108-88-3 | 3.00E+03 | 2 | 2 | 1 | 1 |
| 37 | Chlorobenzene | 108-90-7 | 1.00E+02 | 0 | 1 | 1 | 1 |
| 38 | 1,2-Dichlorobenzene | 95-50-1 | 3.00E+02 | 0 | 1 | 1 | 1 |
| 39 | 1,3-Dichlorobenzene | 541-73-1 | 5.00E+02 | 1 | 2 | 1 | 1 |
| 40 | 1,4-Dichlorobenzene | 106-46-7 | 2.00E+03 | 3 | 3 | 1 | 1 |
| 41 | 1,2,4Trichlorobenzene | 120-82-1 | 5.00E+03 | 3 | 3 | 1 | 1 |
| 42 | Hexachlorobenzene | 118-74-1 | 8.00E-01 | 0 | 0 | 1 | -1 |
| 44 | Ethylbenzene | 100-41-4 | 3.00E+03 | 2 | 2 | 1 | 1 |
| 45 | Styrene | 100-42-5 | 2.00E+03 | 2 | 2 | 1 | 1 |
| 46 | Xylenes | 1330-20-7 | 3.00E+03 | 2 | 2 | 1 | 1 |
| 48 | Phenol | 108-95-2 | 2.00E+01 | -1 | -1 | -1 | -1 |
| 49 | 2-Chlorophenol | 95-57-8 | 3.00E+02 | 1 | 1 | 1 | 1 |
| 50 | 2,4-Dichlorophenol | 120-83-2 | 4.00E+01 | 1 | 1 | 1 | 1 |
| 51 | 2,4,5-Trichlorophenol | 95-95-4 | 6.00E+02 | 0 | 1 | -1 | 1 |
| 52 | 2,4,6-Trichlorophenol | 88-06-2 | 2.00E+01 | 0 | 0 | 1 | 1 |
| 54 | Pentachlorophenol | 87-86-5 | 1.00E+01 | 1 | 1 | 1 | 1 |
| 55 | Benz(a)anthracene | 56-55-3 | 3.00E+02 | 2 | 2 | 1 | 1 |
| 56 | Benzo(a)pyrene | 50-32-8 | 3.00E+01 | 2 | 2 | 1 | 1 |
| 57 | Benzo(b)fluoranthene | 205-99-2 | 3.00E+02 | 2 | 2 | 1 | 1 |
| 58 | Benzo(k)fluoranthene | 207-08-9 | 3.00E+03 | 3 | 3 | 1 | 1 |
| 59 | Chrysene | 218-01-9 | 3.00E+03 | 2 | 2 | 1 | 1 |
| 60 | Dibena(a,h)anthracene | 53-70-3 | 3.00E+01 | 2 | 2 | 1 | 1 |
| 61 | Indeno(1,2,3-c,d)pyrebe | 193-39-5 | 3.00E+02 | 2 | 2 | 1 | 1 |
| 62 | Naphthalene | 91-20-3 | 3.00E+03 | 2 | 3 | 1 | 1 |
| 63 | Acenaphehene | 83-32-9 | 5.00E+03 | 2 | 2 | 1 | 1 |
| 64 | Acenaphthylene | 208-96-8 | 1.00E+01 | -1 | -1 | -1 | -1 |
| 65 | Anthracene | 120-12-7 | 5.00E+03 | 2 | 2 | 1 | 1 |
| 66 | Benzo(g,h,i) perylene | 191-24-2 | 5.00E+03 | 2 | 2 | 1 | 1 |
| 67 | Flouranthene | 206-44-0 | 5.00E+03 | 3 | 2 | 1 | 1 |
| 68 | Flouorene | 86-73-7 | 5.00E+03 | 2 | 2 | 1 | 1 |
| 69 | Phenanthrene | 85-01-8 | 3.00E+03 | 2 | 2 | 1 | 1 |
| 70 | Pyrene | 129-00-0 | 5.00E+03 | 2 | 2 | 1 | 1 |
| 71 | Aldrin | 309-00-2 | 3.00E+00 | 2 | 1 | 1 | 1 |
| 72 | Chlordane | 57-74-9 | 6.00E+01 | 2 | 2 | 1 | 1 |
| 73 | DDT | 50-29-3 | 6.00E+01 | 2 | 1 | 1 | 1 |
| 74 | Dieldrin | 60-57-1 | 3.00E+00 | 2 | 1 | 1 | 1 |
| 75 | Endosulfan | 115-29-7 | 1.00E+00 | -1 | -1 | -1 | -1 |
| 76 | Endrin | 72-20-8 | 2.00E+01 | 1 | 1 | 1 | 1 |
| 77 | Heptachlor | 76-44-8 | 1.00E+01 | 2 | 2 | 1 | 1 |
| 78 | Lindane | 58-89-9 | 5.00E-01 | 0 | 0 | -1 | -1 |
| 92 | Total PCB | 1336-36-3 | 4.00E+00 | 1 | 1 | 1 | 1 |
| 94 | Total PCDD/PCDF | 1746-01-6 | 5.00E-05 | 0 | 0 | -1 | -1 |
| 95 | Methyl ethyl ketone | 78-93-3 | 4.00E+02 | -1 | 0 | -1 | -1 |
| 96 | Methyl isobutyl ketone | 108-10-1 | 4.00E+02 | -1 | 0 | -1 | -1 |
| 98 | DEHP | 117-81-7 | 2.00E+03 | 2 | 1 | 1 | 1 |
| 99 | Hexachlorobutadine | 87-68-3 | 1.00E+02 | 1 | 2 | 1 | 1 |
| 100 | Methyl tert-butyl ether | 1634-04-4 | 5.00E+02 | 1 | 1 | 1 | 1 |
|  |  | Overall | C1 | C2 | C3 | C4 | C5 |
|  |  | Scores | 76 | 1.39 | 1.47 | 60 | 60 |
|  |  |  |  |  | No. >0 | 68 | 68 |
|  |  |  |  |  | No. <0 | 8 | 8 |

**Table S20d** Massachusetts M2-S1-direct

| Number | Pollutant | CAS. No | RGV (mg/kg) | C_2_ | C_3_ | C_4_ | C_5_ |
| --- | --- | --- | --- | --- | --- | --- | --- |
| 1 | Antimony | 7440-36-0 | 2.00E+01 | 0 | 0 | -1 | 1 |
| 2 | Arsenic | 7440-38-2 | 2.00E+01 | 0 | 0 | -1 | 1 |
| 3 | Barium | 7440-39-3 | 1.00E+03 | 0 | 0 | 1 | -1 |
| 4 | Beryllium | 7440-41-7 | 9.00E+01 | 1 | 1 | 1 | 1 |
| 5 | Cadmium | 7440-43-9 | 7.00E+01 | 1 | 1 | 1 | 1 |
| 6 | Chromium (III) | 16065-83-1 | 1.00E+03 | 1 | 0 | 1 | 1 |
| 7 | Chromium (VI) | 18540-29-9 | 1.00E+02 | 0 | 1 | -1 | 1 |
| 10 | Lead | 7439-92-1 | 2.00E+02 | 0 | 0 | -1 | 1 |
| 12 | Mercury | 7439-97-6 | 2.00E+01 | 1 | 1 | 1 | 1 |
| 14 | Nickel | 7440-02-0 | 6.00E+02 | 1 | 1 | 1 | 1 |
| 15 | Selenium | 7782-49-2 | 4.00E+02 | 1 | 1 | 1 | 1 |
| 16 | Silver | 7440-22-4 | 1.00E+02 | 1 | 1 | 1 | 1 |
| 17 | Thallium | 7440-28-0 | 8.00E+00 | 1 | 1 | 1 | 1 |
| 19 | Vanadium | 7440-62-2 | 4.00E+02 | 1 | 1 | 1 | 1 |
| 20 | Zinc | 7440-66-6 | 1.00E+03 | 0 | 0 | 1 | -1 |
| 21 | Cyanide | 57-12-5 | 3.00E+01 | 0 | 0 | -1 | -1 |
| 23 | Dichloromethane | 75-09-2 | 4.00E+02 | 2 | 2 | 1 | 1 |
| 24 | Trichloromethane | 67-66-3 | 5.00E+02 | 3 | 3 | 1 | 1 |
| 25 | Tetrachloromethane | 56-23-5 | 3.00E+01 | 2 | 2 | 1 | 1 |
| 26 | Bromoform | 75-25-2 | 3.00E+02 | 1 | 1 | 1 | 1 |
| 27 | Bromomethane | 74-83-9 | 9.00E+01 | 1 | 1 | 1 | 1 |
| 28 | 1,2-Dichloroethane | 107-06-2 | 2.00E+01 | 1 | 1 | 1 | 1 |
| 29 | 1,1,1-Trichloroethane | 71-55-6 | 5.00E+02 | 0 | 1 | -1 | 1 |
| 30 | 1,1,2-Trichloroethane | 79-00-5 | 4.00E+01 | 1 | 1 | 1 | 1 |
| 31 | Chloroethene | 75-01-4 | 1.00E+00 | 1 | 1 | 1 | 1 |
| 32 | 1,1-Dichloroethene | 75-35-4 | 5.00E+02 | 2 | 2 | 1 | 1 |
| 33 | Trichloroethene | 79-01-6 | 3.00E+01 | 1 | 1 | 1 | 1 |
| 34 | Tetrachloroethene | 127-18-4 | 3.00E+01 | 1 | 1 | 1 | 1 |
| 35 | Benzene | 71-43-2 | 4.00E+01 | 2 | 2 | 1 | 1 |
| 36 | Toluene | 108-88-3 | 5.00E+02 | 1 | 1 | 1 | 1 |
| 37 | Chlorobenzene | 108-90-7 | 5.00E+02 | 1 | 2 | 1 | 1 |
| 38 | 1,2-Dichlorobenzene | 95-50-1 | 1.00E+03 | 1 | 1 | 1 | 1 |
| 39 | 1,3-Dichlorobenzene | 541-73-1 | 1.00E+02 | 1 | 1 | 1 | 1 |
| 40 | 1,4-Dichlorobenzene | 106-46-7 | 8.00E+01 | 1 | 1 | 1 | 1 |
| 41 | 1,2,4Trichlorobenzene | 120-82-1 | 7.00E+02 | 2 | 2 | 1 | 1 |
| 42 | Hexachlorobenzene | 118-74-1 | 7.00E-01 | 0 | 0 | 1 | -1 |
| 44 | Ethylbenzene | 100-41-4 | 5.00E+02 | 2 | 1 | 1 | 1 |
| 45 | Styrene | 100-42-5 | 7.00E+01 | 0 | 0 | -1 | -1 |
| 46 | Xylenes | 1330-20-7 | 5.00E+02 | 1 | 1 | 1 | 1 |
| 48 | Phenol | 108-95-2 | 5.00E+02 | 1 | 1 | 1 | 1 |
| 49 | 2-Chlorophenol | 95-57-8 | 1.00E+02 | 1 | 1 | 1 | 1 |
| 50 | 2,4-Dichlorophenol | 120-83-2 | 7.00E+01 | 1 | 1 | 1 | 1 |
| 51 | 2,4,5-Trichlorophenol | 95-95-4 | 1.00E+03 | 0 | 1 | 1 | 1 |
| 52 | 2,4,6-Trichlorophenol | 88-06-2 | 2.00E+01 | 0 | 0 | 1 | 1 |
| 54 | Pentachlorophenol | 87-86-5 | 3.00E+00 | 0 | 0 | -1 | -1 |
| 55 | Benz(a)anthracene | 56-55-3 | 7.00E+00 | 1 | 1 | 1 | 1 |
| 56 | Benzo(a)pyrene | 50-32-8 | 2.00E+00 | 1 | 1 | 1 | 1 |
| 57 | Benzo(b)fluoranthene | 205-99-2 | 7.00E+00 | 1 | 1 | 1 | 1 |
| 58 | Benzo(k)fluoranthene | 207-08-9 | 7.00E+01 | 1 | 1 | 1 | 1 |
| 59 | Chrysene | 218-01-9 | 7.00E+01 | 1 | 1 | 1 | 1 |
| 60 | Dibena(a,h)anthracene | 53-70-3 | 7.00E-01 | 0 | 0 | 1 | -1 |
| 61 | Indeno(1,2,3-c,d)pyrebe | 193-39-5 | 7.00E+00 | 1 | 1 | 1 | 1 |
| 62 | Naphthalene | 91-20-3 | 5.00E+02 | 2 | 2 | 1 | 1 |
| 63 | Acenaphehene | 83-32-9 | 1.00E+03 | 1 | 1 | 1 | 1 |
| 64 | Acenaphthylene | 208-96-8 | 1.00E+03 | 1 | 1 | 1 | 1 |
| 65 | Anthracene | 120-12-7 | 1.00E+03 | 1 | 1 | 1 | 1 |
| 66 | Benzo(g,h,i) perylene | 191-24-2 | 1.00E+03 | 2 | 1 | 1 | 1 |
| 67 | Flouranthene | 206-44-0 | 1.00E+03 | 2 | 1 | 1 | 1 |
| 68 | Flouorene | 86-73-7 | 1.00E+03 | 1 | 1 | 1 | 1 |
| 69 | Phenanthrene | 85-01-8 | 5.00E+02 | 1 | 1 | 1 | 1 |
| 70 | Pyrene | 129-00-0 | 1.00E+03 | 1 | 1 | 1 | 1 |
| 71 | Aldrin | 309-00-2 | 8.00E-02 | 0 | 0 | -1 | -1 |
| 72 | Chlordane | 57-74-9 | 5.00E+00 | 0 | 0 | 1 | 1 |
| 73 | DDT | 50-29-3 | 6.00E+00 | 1 | 0 | 1 | 1 |
| 74 | Dieldrin | 60-57-1 | 8.00E-02 | 0 | 0 | -1 | -1 |
| 75 | Endosulfan | 115-29-7 | 3.00E+02 | 1 | 1 | 1 | 1 |
| 76 | Endrin | 72-20-8 | 1.00E+01 | 1 | 1 | 1 | 1 |
| 77 | Heptachlor | 76-44-8 | 3.00E-01 | 0 | 0 | 1 | -1 |
| 78 | Lindane | 58-89-9 | 1.00E+00 | 1 | 1 | 1 | 1 |
| 92 | Total PCB | 1336-36-3 | 1.00E+00 | 0 | 0 | 1 | 1 |
| 94 | Total PCDD/PCDF | 1746-01-6 | 2.00E-05 | 0 | 0 | -1 | -1 |
| 95 | Methyl ethyl ketone | 78-93-3 | 5.00E+02 | -1 | 0 | -1 | -1 |
| 96 | Methyl isobutyl ketone | 108-10-1 | 5.00E+02 | -1 | 0 | -1 | -1 |
| 98 | DEHP | 117-81-7 | 9.00E+01 | 0 | 0 | 1 | -1 |
| 99 | Hexachlorobutadine | 87-68-3 | 3.00E+01 | 1 | 1 | 1 | 1 |
| 100 | Methyl tert-butyl ether | 1634-04-4 | 1.00E+02 | 0 | 1 | 1 | 1 |
|  |  | Overall | C1 | C2 | C3 | C4 | C5 |
|  |  | Scores | 76 | 0.79 | 0.83 | 50 | 48 |
|  |  |  |  |  | No. >0 | 63 | 62 |
|  |  |  |  |  | No. <0 | 13 | 14 |

**Table S20e** Massachusetts M2-S2-direct

| Number | Pollutant | CAS. No | RGV (mg/kg) | C_2_ | C_3_ | C_4_ | C_5_ |
| --- | --- | --- | --- | --- | --- | --- | --- |
| 1 | Antimony | 7440-36-0 | 3.00E+01 | 0 | 1 | 1 | 1 |
| 2 | Arsenic | 7440-38-2 | 2.00E+01 | 0 | 0 | -1 | 1 |
| 3 | Barium | 7440-39-3 | 3.00E+03 | 1 | 1 | 1 | 1 |
| 4 | Beryllium | 7440-41-7 | 2.00E+02 | 1 | 1 | 1 | 1 |
| 5 | Cadmium | 7440-43-9 | 1.00E+02 | 1 | 1 | 1 | 1 |
| 6 | Chromium (III) | 16065-83-1 | 3.00E+03 | 1 | 1 | 1 | 1 |
| 7 | Chromium (VI) | 18540-29-9 | 2.00E+02 | 1 | 1 | 1 | 1 |
| 10 | Lead | 7439-92-1 | 6.00E+02 | 1 | 1 | 1 | 1 |
| 12 | Mercury | 7439-97-6 | 3.00E+01 | 1 | 1 | 1 | 1 |
| 14 | Nickel | 7440-02-0 | 1.00E+03 | 1 | 1 | 1 | 1 |
| 15 | Selenium | 7782-49-2 | 7.00E+02 | 2 | 2 | 1 | 1 |
| 16 | Silver | 7440-22-4 | 2.00E+02 | 1 | 1 | 1 | 1 |
| 17 | Thallium | 7440-28-0 | 6.00E+01 | 2 | 2 | 1 | 1 |
| 19 | Vanadium | 7440-62-2 | 7.00E+02 | 1 | 1 | 1 | 1 |
| 20 | Zinc | 7440-66-6 | 3.00E+03 | 1 | 1 | 1 | 1 |
| 21 | Cyanide | 57-12-5 | 4.00E+02 | 1 | 1 | 1 | 1 |
| 23 | Dichloromethane | 75-09-2 | 7.00E+02 | 2 | 2 | 1 | 1 |
| 24 | Trichloromethane | 67-66-3 | 1.00E+03 | 3 | 3 | 1 | 1 |
| 25 | Tetrachloromethane | 56-23-5 | 1.00E+02 | 2 | 2 | 1 | 1 |
| 26 | Bromoform | 75-25-2 | 1.00E+03 | 1 | 2 | 1 | 1 |
| 27 | Bromomethane | 74-83-9 | 6.00E+02 | 2 | 2 | 1 | 1 |
| 28 | 1,2-Dichloroethane | 107-06-2 | 1.00E+02 | 2 | 2 | 1 | 1 |
| 29 | 1,1,1-Trichloroethane | 71-55-6 | 1.00E+03 | 1 | 1 | 1 | 1 |
| 30 | 1,1,2-Trichloroethane | 79-00-5 | 2.00E+02 | 2 | 2 | 1 | 1 |
| 31 | Chloroethene | 75-01-4 | 7.00E+00 | 2 | 2 | 1 | 1 |
| 32 | 1,1-Dichloroethene | 75-35-4 | 1.00E+03 | 2 | 2 | 1 | 1 |
| 33 | Trichloroethene | 79-01-6 | 6.00E+01 | 1 | 2 | 1 | 1 |
| 34 | Tetrachloroethene | 127-18-4 | 2.00E+02 | 2 | 2 | 1 | 1 |
| 35 | Benzene | 71-43-2 | 2.00E+02 | 3 | 3 | 1 | 1 |
| 36 | Toluene | 108-88-3 | 1.00E+03 | 1 | 1 | 1 | 1 |
| 37 | Chlorobenzene | 108-90-7 | 1.00E+03 | 1 | 2 | 1 | 1 |
| 38 | 1,2-Dichlorobenzene | 95-50-1 | 3.00E+03 | 1 | 2 | 1 | 1 |
| 39 | 1,3-Dichlorobenzene | 541-73-1 | 5.00E+02 | 1 | 2 | 1 | 1 |
| 40 | 1,4-Dichlorobenzene | 106-46-7 | 4.00E+02 | 2 | 2 | 1 | 1 |
| 41 | 1,2,4Trichlorobenzene | 120-82-1 | 3.00E+03 | 2 | 2 | 1 | 1 |
| 42 | Hexachlorobenzene | 118-74-1 | 8.00E-01 | 0 | 0 | 1 | -1 |
| 44 | Ethylbenzene | 100-41-4 | 1.00E+03 | 2 | 2 | 1 | 1 |
| 45 | Styrene | 100-42-5 | 3.00E+02 | 1 | 1 | 1 | 1 |
| 46 | Xylenes | 1330-20-7 | 1.00E+03 | 1 | 1 | 1 | 1 |
| 48 | Phenol | 108-95-2 | 1.00E+03 | 1 | 1 | 1 | 1 |
| 49 | 2-Chlorophenol | 95-57-8 | 3.00E+02 | 1 | 1 | 1 | 1 |
| 50 | 2,4-Dichlorophenol | 120-83-2 | 8.00E+02 | 2 | 2 | 1 | 1 |
| 51 | 2,4,5-Trichlorophenol | 95-95-4 | 3.00E+03 | 1 | 1 | 1 | 1 |
| 52 | 2,4,6-Trichlorophenol | 88-06-2 | 4.00E+02 | 2 | 2 | 1 | 1 |
| 54 | Pentachlorophenol | 87-86-5 | 2.00E+01 | 1 | 1 | 1 | 1 |
| 55 | Benz(a)anthracene | 56-55-3 | 4.00E+01 | 2 | 1 | 1 | 1 |
| 56 | Benzo(a)pyrene | 50-32-8 | 7.00E+00 | 1 | 1 | 1 | 1 |
| 57 | Benzo(b)fluoranthene | 205-99-2 | 4.00E+00 | 1 | 0 | 1 | 1 |
| 58 | Benzo(k)fluoranthene | 207-08-9 | 4.00E+02 | 2 | 2 | 1 | 1 |
| 59 | Chrysene | 218-01-9 | 4.00E+02 | 1 | 1 | 1 | 1 |
| 60 | Dibena(a,h)anthracene | 53-70-3 | 4.00E+00 | 1 | 1 | 1 | 1 |
| 61 | Indeno(1,2,3-c,d)pyrebe | 193-39-5 | 4.00E+01 | 1 | 1 | 1 | 1 |
| 62 | Naphthalene | 91-20-3 | 1.00E+03 | 2 | 3 | 1 | 1 |
| 63 | Acenaphehene | 83-32-9 | 3.00E+03 | 2 | 2 | 1 | 1 |
| 64 | Acenaphthylene | 208-96-8 | 3.00E+03 | 2 | 2 | 1 | 1 |
| 65 | Anthracene | 120-12-7 | 3.00E+03 | 2 | 1 | 1 | 1 |
| 66 | Benzo(g,h,i) perylene | 191-24-2 | 3.00E+03 | 2 | 2 | 1 | 1 |
| 67 | Flouranthene | 206-44-0 | 3.00E+03 | 3 | 2 | 1 | 1 |
| 68 | Flouorene | 86-73-7 | 3.00E+03 | 2 | 2 | 1 | 1 |
| 69 | Phenanthrene | 85-01-8 | 1.00E+03 | 2 | 1 | 1 | 1 |
| 70 | Pyrene | 129-00-0 | 3.00E+03 | 1 | 2 | 1 | 1 |
| 71 | Aldrin | 309-00-2 | 5.00E-01 | 1 | 1 | 1 | 1 |
| 72 | Chlordane | 57-74-9 | 3.00E+01 | 1 | 1 | 1 | 1 |
| 73 | DDT | 50-29-3 | 3.00E+01 | 1 | 1 | 1 | 1 |
| 74 | Dieldrin | 60-57-1 | 5.00E-01 | 1 | 0 | 1 | 1 |
| 75 | Endosulfan | 115-29-7 | 5.00E+02 | 1 | 2 | 1 | 1 |
| 76 | Endrin | 72-20-8 | 2.00E+01 | 1 | 1 | 1 | 1 |
| 77 | Heptachlor | 76-44-8 | 2.00E+00 | 1 | 1 | 1 | 1 |
| 78 | Lindane | 58-89-9 | 7.00E+00 | 1 | 1 | 1 | 1 |
| 92 | Total PCB | 1336-36-3 | 4.00E+00 | 1 | 1 | 1 | 1 |
| 94 | Total PCDD/PCDF | 1746-01-6 | 5.00E-05 | 0 | 0 | -1 | -1 |
| 95 | Methyl ethyl ketone | 78-93-3 | 1.00E+03 | -1 | 0 | -1 | -1 |
| 96 | Methyl isobutyl ketone | 108-10-1 | 1.00E+03 | 0 | 0 | -1 | 1 |
| 98 | DEHP | 117-81-7 | 6.00E+02 | 1 | 1 | 1 | 1 |
| 99 | Hexachlorobutadine | 87-68-3 | 1.00E+02 | 1 | 2 | 1 | 1 |
| 100 | Methyl tert-butyl ether | 1634-04-4 | 5.00E+02 | 1 | 1 | 1 | 1 |
|  |  | Overall | C1 | C2 | C3 | C4 | C5 |
|  |  | Scores | 76 | 1.29 | 1.37 | 68 | 70 |
|  |  |  |  |  | No. >0 | 72 | 73 |
|  |  |  |  |  | No. <0 | 4 | 3 |

**Table S20f** Massachusetts M2-S3-direct

| Number | Pollutant | CAS. No | RGV (mg/kg) | C_2_ | C_3_ | C_4_ | C_5_ |
| --- | --- | --- | --- | --- | --- | --- | --- |
| 1 | Antimony | 7440-36-0 | 3.00E+01 | 0 | 1 | 1 | 1 |
| 3 | Barium | 7440-39-3 | 5.00E+03 | 1 | 1 | 1 | 1 |
| 4 | Beryllium | 7440-41-7 | 2.00E+02 | 1 | 1 | 1 | 1 |
| 5 | Cadmium | 7440-43-9 | 1.00E+02 | 1 | 1 | 1 | 1 |
| 6 | Chromium (III) | 16065-83-1 | 5.00E+03 | 2 | 1 | 1 | 1 |
| 7 | Chromium (VI) | 18540-29-9 | 2.00E+02 | 1 | 1 | 1 | 1 |
| 10 | Lead | 7439-92-1 | 6.00E+02 | 1 | 1 | 1 | 1 |
| 12 | Mercury | 7439-97-6 | 3.00E+01 | 1 | 1 | 1 | 1 |
| 14 | Nickel | 7440-02-0 | 1.00E+03 | 1 | 1 | 1 | 1 |
| 15 | Selenium | 7782-49-2 | 7.00E+02 | 2 | 2 | 1 | 1 |
| 16 | Silver | 7440-22-4 | 2.00E+02 | 1 | 1 | 1 | 1 |
| 17 | Thallium | 7440-28-0 | 8.00E+01 | 2 | 2 | 1 | 1 |
| 19 | Vanadium | 7440-62-2 | 7.00E+02 | 1 | 1 | 1 | 1 |
| 20 | Zinc | 7440-66-6 | 5.00E+03 | 1 | 1 | 1 | 1 |
| 21 | Cyanide | 57-12-5 | 5.00E+02 | 1 | 1 | 1 | 1 |
| 23 | Dichloromethane | 75-09-2 | 7.00E+02 | 2 | 2 | 1 | 1 |
| 24 | Trichloromethane | 67-66-3 | 1.00E+03 | 3 | 3 | 1 | 1 |
| 25 | Tetrachloromethane | 56-23-5 | 1.00E+03 | 3 | 3 | 1 | 1 |
| 26 | Bromoform | 75-25-2 | 3.00E+03 | 2 | 2 | 1 | 1 |
| 27 | Bromomethane | 74-83-9 | 6.00E+02 | 2 | 2 | 1 | 1 |
| 28 | 1,2-Dichloroethane | 107-06-2 | 9.00E+02 | 3 | 3 | 1 | 1 |
| 29 | 1,1,1-Trichloroethane | 71-55-6 | 3.00E+03 | 1 | 1 | 1 | 1 |
| 30 | 1,1,2-Trichloroethane | 79-00-5 | 5.00E+02 | 2 | 2 | 1 | 1 |
| 31 | Chloroethene | 75-01-4 | 6.00E+01 | 3 | 3 | 1 | 1 |
| 32 | 1,1-Dichloroethene | 75-35-4 | 3.00E+03 | 3 | 3 | 1 | 1 |
| 33 | Trichloroethene | 79-01-6 | 6.00E+01 | 1 | 2 | 1 | 1 |
| 34 | Tetrachloroethene | 127-18-4 | 1.00E+03 | 3 | 3 | 1 | 1 |
| 35 | Benzene | 71-43-2 | 1.00E+03 | 3 | 3 | 1 | 1 |
| 36 | Toluene | 108-88-3 | 3.00E+03 | 2 | 2 | 1 | 1 |
| 37 | Chlorobenzene | 108-90-7 | 3.00E+03 | 2 | 2 | 1 | 1 |
| 38 | 1,2-Dichlorobenzene | 95-50-1 | 5.00E+03 | 2 | 2 | 1 | 1 |
| 39 | 1,3-Dichlorobenzene | 541-73-1 | 5.00E+02 | 1 | 2 | 1 | 1 |
| 40 | 1,4-Dichlorobenzene | 106-46-7 | 3.00E+03 | 3 | 3 | 1 | 1 |
| 41 | 1,2,4Trichlorobenzene | 120-82-1 | 5.00E+03 | 3 | 3 | 1 | 1 |
| 42 | Hexachlorobenzene | 118-74-1 | 8.00E-01 | 0 | 0 | 1 | -1 |
| 44 | Ethylbenzene | 100-41-4 | 3.00E+03 | 2 | 2 | 1 | 1 |
| 45 | Styrene | 100-42-5 | 3.00E+03 | 2 | 2 | 1 | 1 |
| 46 | Xylenes | 1330-20-7 | 3.00E+03 | 2 | 2 | 1 | 1 |
| 48 | Phenol | 108-95-2 | 3.00E+03 | 2 | 1 | 1 | 1 |
| 49 | 2-Chlorophenol | 95-57-8 | 3.00E+02 | 1 | 1 | 1 | 1 |
| 50 | 2,4-Dichlorophenol | 120-83-2 | 8.00E+02 | 2 | 2 | 1 | 1 |
| 51 | 2,4,5-Trichlorophenol | 95-95-4 | 5.00E+03 | 1 | 2 | 1 | 1 |
| 52 | 2,4,6-Trichlorophenol | 88-06-2 | 4.00E+02 | 2 | 2 | 1 | 1 |
| 54 | Pentachlorophenol | 87-86-5 | 7.00E+01 | 1 | 1 | 1 | 1 |
| 55 | Benz(a)anthracene | 56-55-3 | 3.00E+02 | 2 | 2 | 1 | 1 |
| 56 | Benzo(a)pyrene | 50-32-8 | 3.00E+01 | 2 | 2 | 1 | 1 |
| 57 | Benzo(b)fluoranthene | 205-99-2 | 3.00E+02 | 2 | 2 | 1 | 1 |
| 58 | Benzo(k)fluoranthene | 207-08-9 | 3.00E+03 | 3 | 3 | 1 | 1 |
| 59 | Chrysene | 218-01-9 | 3.00E+03 | 2 | 2 | 1 | 1 |
| 60 | Dibena(a,h)anthracene | 53-70-3 | 3.00E+01 | 2 | 2 | 1 | 1 |
| 61 | Indeno(1,2,3-c,d)pyrebe | 193-39-5 | 3.00E+02 | 2 | 2 | 1 | 1 |
| 62 | Naphthalene | 91-20-3 | 3.00E+03 | 2 | 3 | 1 | 1 |
| 63 | Acenaphehene | 83-32-9 | 5.00E+03 | 2 | 2 | 1 | 1 |
| 64 | Acenaphthylene | 208-96-8 | 5.00E+03 | 2 | 2 | 1 | 1 |
| 65 | Anthracene | 120-12-7 | 5.00E+03 | 2 | 2 | 1 | 1 |
| 66 | Benzo(g,h,i) perylene | 191-24-2 | 5.00E+03 | 2 | 2 | 1 | 1 |
| 67 | Flouranthene | 206-44-0 | 5.00E+03 | 3 | 2 | 1 | 1 |
| 68 | Flouorene | 86-73-7 | 5.00E+03 | 2 | 2 | 1 | 1 |
| 69 | Phenanthrene | 85-01-8 | 3.00E+03 | 2 | 2 | 1 | 1 |
| 70 | Pyrene | 129-00-0 | 5.00E+03 | 2 | 2 | 1 | 1 |
| 71 | Aldrin | 309-00-2 | 3.00E+00 | 2 | 1 | 1 | 1 |
| 72 | Chlordane | 57-74-9 | 6.00E+01 | 2 | 2 | 1 | 1 |
| 73 | DDT | 50-29-3 | 6.00E+01 | 2 | 1 | 1 | 1 |
| 74 | Dieldrin | 60-57-1 | 3.00E+00 | 2 | 1 | 1 | 1 |
| 75 | Endosulfan | 115-29-7 | 5.00E+02 | 1 | 2 | 1 | 1 |
| 76 | Endrin | 72-20-8 | 2.00E+01 | 1 | 1 | 1 | 1 |
| 77 | Heptachlor | 76-44-8 | 1.00E+01 | 2 | 2 | 1 | 1 |
| 78 | Lindane | 58-89-9 | 6.00E+01 | 2 | 2 | 1 | 1 |
| 92 | Total PCB | 1336-36-3 | 4.00E+00 | 1 | 1 | 1 | 1 |
| 94 | Total PCDD/PCDF | 1746-01-6 | 5.00E-05 | 0 | 0 | -1 | -1 |
| 95 | Methyl ethyl ketone | 78-93-3 | 3.00E+03 | 0 | 1 | -1 | 1 |
| 96 | Methyl isobutyl ketone | 108-10-1 | 3.00E+03 | 0 | 1 | -1 | 1 |
| 98 | DEHP | 117-81-7 | 2.00E+03 | 2 | 1 | 1 | 1 |
| 99 | Hexachlorobutadine | 87-68-3 | 1.00E+02 | 1 | 2 | 1 | 1 |
| 100 | Methyl tert-butyl ether | 1634-04-4 | 5.00E+02 | 1 | 1 | 1 | 1 |
|  |  | Overall | C1 | C2 | C3 | C4 | C5 |
|  |  | Scores | 76 | 1.70 | 1.74 | 69 | 71 |
|  |  |  |  |  | No. >0 | 72 | 73 |
|  |  |  |  |  | No. <0 | 3 | 2 |

**Table S20g** Massachusetts M3

| Number | Pollutant | CAS. No | RGV (mg/kg) | C_2_ | C_3_ | C_4_ | C_5_ |
| --- | --- | --- | --- | --- | --- | --- | --- |
| 1 | Antimony | 7440-36-0 | 3.00E+02 | 1 | 2 | 1 | 1 |
| 2 | Arsenic | 7440-38-2 | 5.00E+02 | 2 | 2 | 1 | 1 |
| 3 | Barium | 7440-39-3 | 1.00E+04 | 1 | 1 | 1 | 1 |
| 4 | Beryllium | 7440-41-7 | 2.00E+03 | 2 | 2 | 1 | 1 |
| 5 | Cadmium | 7440-43-9 | 1.00E+03 | 2 | 2 | 1 | 1 |
| 6 | Chromium (III) | 16065-83-1 | 1.00E+04 | 2 | 1 | 1 | 1 |
| 7 | Chromium (VI) | 18540-29-9 | 2.00E+03 | 2 | 2 | 1 | 1 |
| 10 | Lead | 7439-92-1 | 6.00E+03 | 2 | 2 | 1 | 1 |
| 12 | Mercury | 7439-97-6 | 3.00E+02 | 2 | 2 | 1 | 1 |
| 14 | Nickel | 7440-02-0 | 1.00E+04 | 2 | 2 | 1 | 1 |
| 15 | Selenium | 7782-49-2 | 7.00E+03 | 3 | 3 | 1 | 1 |
| 16 | Silver | 7440-22-4 | 2.00E+03 | 2 | 2 | 1 | 1 |
| 17 | Thallium | 7440-28-0 | 8.00E+02 | 3 | 3 | 1 | 1 |
| 19 | Vanadium | 7440-62-2 | 7.00E+03 | 2 | 2 | 1 | 1 |
| 20 | Zinc | 7440-66-6 | 1.00E+04 | 1 | 1 | 1 | 1 |
| 21 | Cyanide | 57-12-5 | 5.00E+03 | 2 | 2 | 1 | 1 |
| 23 | Dichloromethane | 75-09-2 | 7.00E+03 | 3 | 3 | 1 | 1 |
| 24 | Trichloromethane | 67-66-3 | 1.00E+04 | 4 | 4 | 1 | 1 |
| 25 | Tetrachloromethane | 56-23-5 | 1.00E+04 | 4 | 4 | 1 | 1 |
| 26 | Bromoform | 75-25-2 | 1.00E+04 | 2 | 3 | 1 | 1 |
| 27 | Bromomethane | 74-83-9 | 6.00E+03 | 3 | 3 | 1 | 1 |
| 28 | 1,2-Dichloroethane | 107-06-2 | 9.00E+03 | 4 | 4 | 1 | 1 |
| 29 | 1,1,1-Trichloroethane | 71-55-6 | 1.00E+04 | 2 | 2 | 1 | 1 |
| 30 | 1,1,2-Trichloroethane | 79-00-5 | 5.00E+03 | 3 | 3 | 1 | 1 |
| 31 | Chloroethene | 75-01-4 | 6.00E+02 | 4 | 4 | 1 | 1 |
| 32 | 1,1-Dichloroethene | 75-35-4 | 1.00E+04 | 3 | 3 | 1 | 1 |
| 33 | Trichloroethene | 79-01-6 | 6.00E+02 | 2 | 3 | 1 | 1 |
| 34 | Tetrachloroethene | 127-18-4 | 1.00E+04 | 4 | 4 | 1 | 1 |
| 35 | Benzene | 71-43-2 | 1.00E+04 | 4 | 4 | 1 | 1 |
| 36 | Toluene | 108-88-3 | 1.00E+04 | 2 | 2 | 1 | 1 |
| 37 | Chlorobenzene | 108-90-7 | 1.00E+04 | 2 | 3 | 1 | 1 |
| 38 | 1,2-Dichlorobenzene | 95-50-1 | 1.00E+04 | 2 | 2 | 1 | 1 |
| 39 | 1,3-Dichlorobenzene | 541-73-1 | 5.00E+03 | 2 | 3 | 1 | 1 |
| 40 | 1,4-Dichlorobenzene | 106-46-7 | 1.00E+04 | 3 | 3 | 1 | 1 |
| 41 | 1,2,4Trichlorobenzene | 120-82-1 | 1.00E+04 | 3 | 3 | 1 | 1 |
| 42 | Hexachlorobenzene | 118-74-1 | 8.00E+00 | 1 | 1 | 1 | 1 |
| 44 | Ethylbenzene | 100-41-4 | 1.00E+04 | 3 | 3 | 1 | 1 |
| 45 | Styrene | 100-42-5 | 1.00E+04 | 2 | 2 | 1 | 1 |
| 46 | Xylenes | 1330-20-7 | 1.00E+04 | 2 | 2 | 1 | 1 |
| 48 | Phenol | 108-95-2 | 1.00E+04 | 2 | 2 | 1 | 1 |
| 49 | 2-Chlorophenol | 95-57-8 | 3.00E+03 | 2 | 2 | 1 | 1 |
| 50 | 2,4-Dichlorophenol | 120-83-2 | 8.00E+03 | 3 | 3 | 1 | 1 |
| 51 | 2,4,5-Trichlorophenol | 95-95-4 | 1.00E+04 | 1 | 2 | 1 | 1 |
| 52 | 2,4,6-Trichlorophenol | 88-06-2 | 4.00E+03 | 3 | 3 | 1 | 1 |
| 54 | Pentachlorophenol | 87-86-5 | 7.00E+02 | 2 | 2 | 1 | 1 |
| 55 | Benz(a)anthracene | 56-55-3 | 3.00E+03 | 3 | 3 | 1 | 1 |
| 56 | Benzo(a)pyrene | 50-32-8 | 3.00E+02 | 3 | 3 | 1 | 1 |
| 57 | Benzo(b)fluoranthene | 205-99-2 | 3.00E+03 | 3 | 3 | 1 | 1 |
| 58 | Benzo(k)fluoranthene | 207-08-9 | 1.00E+04 | 3 | 3 | 1 | 1 |
| 59 | Chrysene | 218-01-9 | 1.00E+04 | 3 | 3 | 1 | 1 |
| 60 | Dibena(a,h)anthracene | 53-70-3 | 3.00E+02 | 3 | 3 | 1 | 1 |
| 61 | Indeno(1,2,3-c,d)pyrebe | 193-39-5 | 3.00E+03 | 3 | 3 | 1 | 1 |
| 62 | Naphthalene | 91-20-3 | 1.00E+04 | 3 | 4 | 1 | 1 |
| 63 | Acenaphehene | 83-32-9 | 1.00E+04 | 2 | 2 | 1 | 1 |
| 64 | Acenaphthylene | 208-96-8 | 1.00E+04 | 2 | 2 | 1 | 1 |
| 65 | Anthracene | 120-12-7 | 1.00E+04 | 2 | 2 | 1 | 1 |
| 66 | Benzo(g,h,i) perylene | 191-24-2 | 1.00E+04 | 3 | 2 | 1 | 1 |
| 67 | Flouranthene | 206-44-0 | 1.00E+04 | 3 | 2 | 1 | 1 |
| 68 | Flouorene | 86-73-7 | 1.00E+04 | 2 | 2 | 1 | 1 |
| 69 | Phenanthrene | 85-01-8 | 1.00E+04 | 3 | 2 | 1 | 1 |
| 70 | Pyrene | 129-00-0 | 1.00E+04 | 2 | 2 | 1 | 1 |
| 71 | Aldrin | 309-00-2 | 3.00E+01 | 3 | 2 | 1 | 1 |
| 72 | Chlordane | 57-74-9 | 6.00E+02 | 3 | 3 | 1 | 1 |
| 73 | DDT | 50-29-3 | 6.00E+02 | 3 | 2 | 1 | 1 |
| 74 | Dieldrin | 60-57-1 | 3.00E+01 | 3 | 2 | 1 | 1 |
| 75 | Endosulfan | 115-29-7 | 5.00E+03 | 2 | 3 | 1 | 1 |
| 76 | Endrin | 72-20-8 | 2.00E+02 | 2 | 2 | 1 | 1 |
| 77 | Heptachlor | 76-44-8 | 1.00E+02 | 3 | 3 | 1 | 1 |
| 78 | Lindane | 58-89-9 | 6.00E+02 | 3 | 3 | 1 | 1 |
| 92 | Total PCB | 1336-36-3 | 1.00E+02 | 2 | 2 | 1 | 1 |
| 94 | Total PCDD/PCDF | 1746-01-6 | 5.00E-05 | 0 | 0 | -1 | -1 |
| 95 | Methyl ethyl ketone | 78-93-3 | 1.00E+04 | 0 | 1 | 1 | 1 |
| 96 | Methyl isobutyl ketone | 108-10-1 | 1.00E+04 | 1 | 1 | 1 | 1 |
| 98 | DEHP | 117-81-7 | 1.00E+04 | 2 | 2 | 1 | 1 |
| 99 | Hexachlorobutadine | 87-68-3 | 1.00E+03 | 2 | 3 | 1 | 1 |
| 100 | Methyl tert-butyl ether | 1634-04-4 | 5.00E+03 | 2 | 2 | 1 | 1 |
|  |  | Overall | C1 | C2 | C3 | C4 | C5 |
|  |  | Scores | 76 | 2.39 | 2.45 | 74 | 74 |
|  |  |  |  |  | No. >0 | 75 | 75 |
|  |  |  |  |  | No. <0 | 1 | 1 |

**Table S21** Michigan Dept. of Environmental Quality

| Number | Pollutant | CAS. No | RGV (mg/kg) | C_2_ | C_3_ | C_4_ | C_5_ |
| --- | --- | --- | --- | --- | --- | --- | --- |
| 1 | Antimony | 7440-36-0 | 1.80E+02 | 1 | 1 | 1 | 1 |
| 2 | Arsenic | 7440-38-2 | 7.60E+00 | 0 | 0 | -1 | -1 |
| 3 | Barium | 7440-39-3 | 3.70E+04 | 2 | 2 | 1 | 1 |
| 4 | Beryllium | 7440-41-7 | 4.10E+02 | 2 | 1 | 1 | 1 |
| 5 | Cadmium | 7440-43-9 | 5.50E+02 | 2 | 2 | 1 | 1 |
| 6 | Chromium (III) | 16065-83-1 | 7.90E+05 | 4 | 3 | 1 | 1 |
| 7 | Chromium (VI) | 18540-29-9 | 2.50E+03 | 2 | 2 | 1 | 1 |
| 8 | Cobalt | 7440-48-4 | 2.60E+03 | 2 | 2 | 1 | 1 |
| 9 | Copper | 7440-50-8 | 2.00E+04 | 2 | 2 | 1 | 1 |
| 10 | Lead | 7439-92-1 | 4.00E+02 | 0 | 1 | 1 | 1 |
| 11 | Manganese | 7439-96-5 | 2.50E+04 | 1 | 1 | 1 | 1 |
| 12 | Mercury | 7439-97-6 | 1.60E+02 | 2 | 2 | 1 | 1 |
| 13 | Molybdenum | 7439-98-7 | 2.60E+03 | 2 | 2 | 1 | 1 |
| 14 | Nickel | 7440-02-0 | 4.00E+04 | 3 | 3 | 1 | 1 |
| 15 | Selenium | 7782-49-2 | 2.60E+03 | 2 | 2 | 1 | 1 |
| 16 | Silver | 7440-22-4 | 2.50E+03 | 2 | 2 | 1 | 1 |
| 17 | Thallium | 7440-28-0 | 3.50E+01 | 1 | 1 | 1 | 1 |
| 19 | Vanadium | 7440-62-2 | 7.50E+02 | 1 | 1 | 1 | 1 |
| 20 | Zinc | 7440-66-6 | 1.70E+05 | 3 | 2 | 1 | 1 |
| 21 | Cyanide | 57-12-5 | 1.20E+01 | 0 | 0 | -1 | -1 |
| 22 | Acrylonitril | 107-13-1 | 1.60E+01 | 1 | 1 | 1 | 1 |
| 23 | Dichloromethane | 75-09-2 | 1.30E+03 | 2 | 2 | 1 | 1 |
| 24 | Trichloromethane | 67-66-3 | 1.20E+03 | 3 | 3 | 1 | 1 |
| 25 | Tetrachloromethane | 56-23-5 | 9.60E+01 | 2 | 2 | 1 | 1 |
| 26 | Bromoform | 75-25-2 | 8.20E+02 | 1 | 1 | 1 | 1 |
| 27 | Bromomethane | 74-83-9 | 3.20E+02 | 2 | 2 | 1 | 1 |
| 29 | 1,1,1-Trichloroethane | 71-55-6 | 5.00E+05 | 3 | 4 | 1 | 1 |
| 30 | 1,1,2-Trichloroethane | 79-00-5 | 1.80E+02 | 2 | 2 | 1 | 1 |
| 31 | Chloroethene | 75-01-4 | 3.80E+00 | 2 | 1 | 1 | 1 |
| 32 | 1,1-Dichloroethene | 75-35-4 | 2.00E+02 | 1 | 2 | 1 | 1 |
| 33 | Trichloroethene | 79-01-6 | 1.10E+02 | 2 | 2 | 1 | 1 |
| 34 | Tetrachloroethene | 127-18-4 | 2.00E+02 | 2 | 2 | 1 | 1 |
| 35 | Benzene | 71-43-2 | 1.80E+02 | 3 | 2 | 1 | 1 |
| 36 | Toluene | 108-88-3 | 5.00E+04 | 3 | 3 | 1 | 1 |
| 37 | Chlorobenzene | 108-90-7 | 4.30E+03 | 2 | 2 | 1 | 1 |
| 38 | 1,2-Dichlorobenzene | 95-50-1 | 1.90E+04 | 2 | 3 | 1 | 1 |
| 39 | 1,3-Dichlorobenzene | 541-73-1 | 2.00E+02 | 1 | 1 | 1 | 1 |
| 40 | 1,4-Dichlorobenzene | 106-46-7 | 4.00E+02 | 2 | 2 | 1 | 1 |
| 41 | 1,2,4Trichlorobenzene | 120-82-1 | 9.90E+02 | 2 | 2 | 1 | 1 |
| 42 | Hexachlorobenzene | 118-74-1 | 8.90E+00 | 1 | 1 | 1 | 1 |
| 43 | Nitrobenzene | 98-95-3 | 1.00E+02 | 1 | 1 | 1 | 1 |
| 44 | Ethylbenzene | 100-41-4 | 2.20E+04 | 3 | 3 | 1 | 1 |
| 45 | Styrene | 100-42-5 | 4.00E+02 | 1 | 1 | 1 | 1 |
| 46 | Xylenes | 1330-20-7 | 4.10E+04 | 3 | 3 | 1 | 1 |
| 47 | Cresol | 1319-77-3 | 1.10E+04 | 2 | 2 | 1 | 1 |
| 48 | Phenol | 108-95-2 | 4.00E+04 | 3 | 2 | 1 | 1 |
| 49 | 2-Chlorophenol | 95-57-8 | 1.40E+03 | 2 | 2 | 1 | 1 |
| 50 | 2,4-Dichlorophenol | 120-83-2 | 6.60E+02 | 2 | 2 | 1 | 1 |
| 51 | 2,4,5-Trichlorophenol | 95-95-4 | 2.30E+04 | 2 | 2 | 1 | 1 |
| 52 | 2,4,6-Trichlorophenol | 88-06-2 | 7.10E+02 | 2 | 2 | 1 | 1 |
| 54 | Pentachlorophenol | 87-86-5 | 9.00E+01 | 1 | 2 | 1 | 1 |
| 55 | Benz(a)anthracene | 56-55-3 | 2.00E+01 | 1 | 1 | 1 | 1 |
| 56 | Benzo(a)pyrene | 50-32-8 | 2.00E+00 | 1 | 1 | 1 | 1 |
| 57 | Benzo(b)fluoranthene | 205-99-2 | 2.00E+01 | 1 | 1 | 1 | 1 |
| 58 | Benzo(k)fluoranthene | 207-08-9 | 2.00E+02 | 2 | 2 | 1 | 1 |
| 59 | Chrysene | 218-01-9 | 2.00E+03 | 2 | 2 | 1 | 1 |
| 60 | Dibena(a,h)anthracene | 53-70-3 | 2.00E+00 | 1 | 1 | 1 | 1 |
| 61 | Indeno(1,2,3-c,d)pyrebe | 193-39-5 | 2.00E+01 | 1 | 1 | 1 | 1 |
| 62 | Naphthalene | 91-20-3 | 1.60E+04 | 3 | 4 | 1 | 1 |
| 63 | Acenaphehene | 83-32-9 | 4.10E+04 | 3 | 3 | 1 | 1 |
| 64 | Acenaphthylene | 208-96-8 | 1.60E+03 | 2 | 2 | 1 | 1 |
| 65 | Anthracene | 120-12-7 | 2.30E+05 | 4 | 3 | 1 | 1 |
| 66 | Benzo(g,h,i) perylene | 191-24-2 | 2.50E+03 | 2 | 2 | 1 | 1 |
| 67 | Flouranthene | 206-44-0 | 4.60E+04 | 4 | 3 | 1 | 1 |
| 68 | Flouorene | 86-73-7 | 2.70E+04 | 3 | 3 | 1 | 1 |
| 69 | Phenanthrene | 85-01-8 | 1.60E+03 | 2 | 2 | 1 | 1 |
| 70 | Pyrene | 129-00-0 | 2.90E+04 | 2 | 3 | 1 | 1 |
| 71 | Aldrin | 309-00-2 | 1.00E+00 | 1 | 1 | 1 | 1 |
| 72 | Chlordane | 57-74-9 | 3.10E+01 | 1 | 1 | 1 | 1 |
| 73 | DDT | 50-29-3 | 5.70E+01 | 2 | 1 | 1 | 1 |
| 74 | Dieldrin | 60-57-1 | 1.10E+03 | 4 | 4 | 1 | 1 |
| 75 | Endosulfan | 115-29-7 | 1.40E+03 | 2 | 2 | 1 | 1 |
| 76 | Endrin | 72-20-8 | 6.50E+01 | 1 | 2 | 1 | 1 |
| 77 | Heptachlor | 76-44-8 | 5.60E+00 | 2 | 1 | 1 | 1 |
| 78 | Lindane | 58-89-9 | 8.30E+00 | 1 | 1 | 1 | 1 |
| 79 | Toxaphene | 8001-35-2 | 2.00E+01 | 2 | 1 | 1 | 1 |
| 80 | 2,4-D | 94-75-7 | 2.50E+03 | 1 | 1 | 1 | 1 |
| 81 | Atrazine | 1912-24-9 | 7.10E+01 | 2 | 1 | 1 | 1 |
| 82 | Carbaryl | 63-25-2 | 2.20E+04 | 1 | 2 | 1 | 1 |
| 83 | Carbofuran | 1563-66-2 | 1.10E+03 | 1 | 2 | 1 | 1 |
| 84 | Chloryrifos | 2921-88-2 | 1.10E+04 | 2 | 2 | 1 | 1 |
| 85 | Diuron | 330-54-1 | 9.70E+02 | 1 | 2 | 1 | 1 |
| 86 | Gylphosate | 1071-83-6 | 1.10E+04 | 0 | 1 | 1 | 1 |
| 88 | MCPA | 94-74-6 | 2.30E+02 | 1 | 1 | 1 | 1 |
| 89 | Picloram | 1918-02-1 | 1.60E+04 | 1 | 1 | 1 | 1 |
| 90 | Simazine | 122-34-9 | 1.20E+03 | 3 | 3 | 1 | 1 |
| 91 | Trifluralin | 1582-09-8 | 2.00E+03 | 2 | 2 | 1 | 1 |
| 92 | Total PCB | 1336-36-3 | 1.20E+00 | 0 | 1 | 1 | 1 |
| 94 | Total PCDD/PCDF | 1746-01-6 | 9.00E-05 | 0 | 0 | 1 | -1 |
| 95 | Methyl ethyl ketone | 78-93-3 | 1.20E+05 | 1 | 2 | 1 | 1 |
| 96 | Methyl isobutyl ketone | 108-10-1 | 5.60E+04 | 1 | 2 | 1 | 1 |
| 97 | Dibutyl phthalate | 84-74-2 | 2.70E+04 | 1 | 2 | 1 | 1 |
| 98 | DEHP | 117-81-7 | 2.80E+03 | 2 | 2 | 1 | 1 |
| 99 | Hexachlorobutadine | 87-68-3 | 1.00E+02 | 1 | 2 | 1 | 1 |
| 100 | Methyl tert-butyl ether | 1634-04-4 | 1.50E+03 | 2 | 2 | 1 | 1 |
|  |  | Overall | C1 | C2 | C3 | C4 | C5 |
|  |  | Scores | 95 | 1.77 | 1.82 | 91 | 89 |
|  |  |  |  |  | No. >0 | 93 | 92 |
|  |  |  |  |  | No. <0 | 2 | 3 |

**Table S22** Minnesota Pollution Control Agency

| Number | Pollutant | CAS. No | RGV (mg/kg) | C_2_ | C_3_ | C_4_ | C_5_ |
| --- | --- | --- | --- | --- | --- | --- | --- |
| 1 | Antimony | 7440-36-0 | 1.20E+01 | 0 | 0 | -1 | -1 |
| 2 | Arsenic | 7440-38-2 | 9.00E+00 | 0 | 0 | -1 | -1 |
| 3 | Barium | 7440-39-3 | 1.10E+03 | 0 | 0 | 1 | 1 |
| 4 | Beryllium | 7440-41-7 | 5.50E+01 | 1 | 1 | 1 | 1 |
| 5 | Cadmium | 7440-43-9 | 2.50E+01 | 1 | 1 | 1 | 1 |
| 6 | Chromium (III) | 16065-83-1 | 4.40E+04 | 2 | 2 | 1 | 1 |
| 7 | Chromium (VI) | 18540-29-9 | 8.70E+01 | 0 | 0 | -1 | 1 |
| 8 | Cobalt | 7440-48-4 | 6.00E+02 | 1 | 1 | 1 | 1 |
| 9 | Copper | 7440-50-8 | 1.00E+02 | 0 | 0 | -1 | -1 |
| 10 | Lead | 7439-92-1 | 3.00E+02 | 0 | 0 | 1 | 1 |
| 11 | Manganese | 7439-96-5 | 3.60E+03 | 1 | 1 | 1 | 1 |
| 12 | Mercury | 7439-97-6 | 5.00E-01 | -1 | -1 | -1 | -1 |
| 14 | Nickel | 7440-02-0 | 5.60E+02 | 1 | 1 | 1 | 1 |
| 15 | Selenium | 7782-49-2 | 1.60E+02 | 1 | 1 | 1 | 1 |
| 16 | Silver | 7440-22-4 | 1.60E+02 | 1 | 1 | 1 | 1 |
| 17 | Thallium | 7440-28-0 | 3.00E+00 | 0 | 0 | -1 | 1 |
| 18 | Tin | 7440-31-5 | 9.00E+03 | 2 | 1 | 1 | 1 |
| 19 | Vanadium | 7440-62-2 | 3.00E+01 | 0 | -1 | -1 | -1 |
| 20 | Zinc | 7440-66-6 | 8.70E+03 | 1 | 1 | 1 | 1 |
| 21 | Cyanide | 57-12-5 | 6.00E+01 | 0 | 0 | 1 | 1 |
| 23 | Dichloromethane | 75-09-2 | 9.70E+01 | 1 | 1 | 1 | 1 |
| 24 | Trichloromethane | 67-66-3 | 2.50E+00 | 1 | 0 | 1 | 1 |
| 25 | Tetrachloromethane | 56-23-5 | 3.00E-01 | 0 | 0 | -1 | -1 |
| 26 | Bromoform | 75-25-2 | 3.70E+02 | 1 | 1 | 1 | 1 |
| 27 | Bromomethane | 74-83-9 | 7.00E-01 | -1 | -1 | -1 | -1 |
| 28 | 1,2-Dichloroethane | 107-06-2 | 4.00E+00 | 1 | 1 | 1 | 1 |
| 29 | 1,1,1-Trichloroethane | 71-55-6 | 1.40E+02 | 0 | 0 | -1 | -1 |
| 30 | 1,1,2-Trichloroethane | 79-00-5 | 9.00E+00 | 1 | 1 | 1 | 1 |
| 31 | Chloroethene | 75-01-4 | 8.00E-01 | 1 | 1 | 1 | 1 |
| 32 | 1,1-Dichloroethene | 75-35-4 | 2.00E+01 | 0 | 1 | 1 | 1 |
| 33 | Trichloroethene | 79-01-6 | 2.90E+01 | 1 | 1 | 1 | 1 |
| 34 | Tetrachloroethene | 127-18-4 | 7.20E+01 | 2 | 2 | 1 | 1 |
| 35 | Benzene | 71-43-2 | 6.00E+00 | 1 | 1 | 1 | 1 |
| 36 | Toluene | 108-88-3 | 1.07E+02 | 0 | 0 | -1 | 1 |
| 37 | Chlorobenzene | 108-90-7 | 1.10E+01 | -1 | 0 | -1 | -1 |
| 38 | 1,2-Dichlorobenzene | 95-50-1 | 2.60E+01 | -1 | 0 | -1 | -1 |
| 39 | 1,3-Dichlorobenzene | 541-73-1 | 2.60E+01 | 0 | 0 | -1 | 1 |
| 40 | 1,4-Dichlorobenzene | 106-46-7 | 3.00E+01 | 1 | 1 | 1 | 1 |
| 41 | 1,2,4Trichlorobenzene | 120-82-1 | 2.00E+02 | 1 | 1 | 1 | 1 |
| 42 | Hexachlorobenzene | 118-74-1 | 5.00E+00 | 1 | 1 | 1 | 1 |
| 44 | Ethylbenzene | 100-41-4 | 2.00E+02 | 1 | 1 | 1 | 1 |
| 45 | Styrene | 100-42-5 | 2.10E+02 | 1 | 1 | 1 | 1 |
| 46 | Xylenes | 1330-20-7 | 4.50E+01 | 0 | 0 | -1 | -1 |
| 47 | Cresol | 1319-77-3 | 1.00E+01 | -1 | -1 | -1 | -1 |
| 48 | Phenol | 108-95-2 | 1.50E+03 | 1 | 1 | 1 | 1 |
| 51 | 2,4,5-Trichlorophenol | 95-95-4 | 1.92E+03 | 1 | 1 | 1 | 1 |
| 52 | 2,4,6-Trichlorophenol | 88-06-2 | 5.95E+02 | 2 | 2 | 1 | 1 |
| 53 | 2,3,4,6-Tetrahlorophenol | 58-90-2 | 6.36E+02 | 1 | 1 | 1 | 1 |
| 54 | Pentachlorophenol | 87-86-5 | 8.00E+01 | 1 | 2 | 1 | 1 |
| 62 | Naphthalene | 91-20-3 | 1.00E+01 | 0 | 1 | -1 | 1 |
| 63 | Acenaphehene | 83-32-9 | 1.20E+03 | 1 | 1 | 1 | 1 |
| 65 | Anthracene | 120-12-7 | 7.88E+03 | 2 | 2 | 1 | 1 |
| 67 | Flouranthene | 206-44-0 | 1.08E+03 | 2 | 1 | 1 | 1 |
| 68 | Flouorene | 86-73-7 | 8.50E+02 | 1 | 1 | 1 | 1 |
| 70 | Pyrene | 129-00-0 | 8.90E+02 | 1 | 1 | 1 | 1 |
| 71 | Aldrin | 309-00-2 | 1.00E+00 | 1 | 1 | 1 | 1 |
| 72 | Chlordane | 57-74-9 | 1.30E+01 | 1 | 1 | 1 | 1 |
| 73 | DDT | 50-29-3 | 1.50E+01 | 1 | 1 | 1 | 1 |
| 74 | Dieldrin | 60-57-1 | 8.00E-01 | 1 | 1 | 1 | 1 |
| 75 | Endosulfan | 115-29-7 | 1.20E+02 | 1 | 1 | 1 | 1 |
| 76 | Endrin | 72-20-8 | 8.00E+00 | 0 | 1 | 1 | 1 |
| 77 | Heptachlor | 76-44-8 | 2.00E+00 | 1 | 1 | 1 | 1 |
| 78 | Lindane | 58-89-9 | 9.00E+00 | 1 | 1 | 1 | 1 |
| 79 | Toxaphene | 8001-35-2 | 1.30E+01 | 2 | 1 | 1 | 1 |
| 88 | MCPA | 94-74-6 | 1.60E+01 | 0 | 0 | -1 | -1 |
| 89 | Picloram | 1918-02-1 | 2.00E+03 | 0 | 0 | -1 | 1 |
| 92 | Total PCB | 1336-36-3 | 1.20E+00 | 0 | 1 | 1 | 1 |
| 94 | Total PCDD/PCDF | 1746-01-6 | 2.00E-05 | 0 | 0 | -1 | -1 |
| 95 | Methyl ethyl ketone | 78-93-3 | 5.50E+03 | 0 | 1 | -1 | 1 |
| 96 | Methyl isobutyl ketone | 108-10-1 | 1.70E+03 | 0 | 1 | -1 | 1 |
| 97 | Dibutyl phthalate | 84-74-2 | 2.44E+03 | 0 | 0 | -1 | 1 |
| 99 | Hexachlorobutadine | 87-68-3 | 6.00E+00 | 0 | 0 | -1 | 1 |
|  |  | Overall | C1 | C2 | C3 | C4 | C5 |
|  |  | Scores | 72 | 0.61 | 0.67 | 24 | 44 |
|  |  |  |  |  | No. >0 | 48 | 58 |
|  |  |  |  |  | No. <0 | 24 | 14 |

**Table S23** Mississippi Dept. of Environmental Quality

| Number | Pollutant | CAS. No | RGV (mg/kg) | C_2_ | C_3_ | C_4_ | C_5_ |
| --- | --- | --- | --- | --- | --- | --- | --- |
| 1 | Antimony | 7440-36-0 | 3.13E+01 | 0 | 1 | 1 | 1 |
| 2 | Arsenic | 7440-38-2 | 4.26E-01 | -1 | -1 | -1 | -1 |
| 3 | Barium | 7440-39-3 | 5.48E+03 | 1 | 1 | 1 | 1 |
| 4 | Beryllium | 7440-41-7 | 1.56E+02 | 1 | 1 | 1 | 1 |
| 5 | Cadmium | 7440-43-9 | 3.91E+01 | 1 | 1 | 1 | 1 |
| 6 | Chromium (III) | 16065-83-1 | 1.17E+05 | 3 | 2 | 1 | 1 |
| 7 | Chromium (VI) | 18540-29-9 | 2.27E+02 | 1 | 1 | 1 | 1 |
| 8 | Cobalt | 7440-48-4 | 4.69E+03 | 2 | 2 | 1 | 1 |
| 9 | Copper | 7440-50-8 | 3.13E+03 | 1 | 1 | 1 | 1 |
| 10 | Lead | 7439-92-1 | 4.00E+02 | 0 | 1 | 1 | 1 |
| 11 | Manganese | 7439-96-5 | 1.56E+03 | 0 | 0 | -1 | -1 |
| 12 | Mercury | 7439-97-6 | 1.00E+01 | 1 | 1 | 1 | 1 |
| 13 | Molybdenum | 7439-98-7 | 3.91E+02 | 1 | 1 | 1 | 1 |
| 14 | Nickel | 7440-02-0 | 1.56E+03 | 1 | 1 | 1 | 1 |
| 15 | Selenium | 7782-49-2 | 3.91E+02 | 1 | 1 | 1 | 1 |
| 16 | Silver | 7440-22-4 | 3.91E+02 | 1 | 1 | 1 | 1 |
| 17 | Thallium | 7440-28-0 | 5.48E+00 | 1 | 1 | 1 | 1 |
| 18 | Tin | 7440-31-5 | 4.69E+04 | 3 | 2 | 1 | 1 |
| 19 | Vanadium | 7440-62-2 | 5.48E+02 | 1 | 1 | 1 | 1 |
| 20 | Zinc | 7440-66-6 | 2.35E+04 | 2 | 2 | 1 | 1 |
| 21 | Cyanide | 57-12-5 | 1.56E+03 | 2 | 2 | 1 | 1 |
| 22 | Acrylonitril | 107-13-1 | 1.18E+00 | 0 | 0 | -1 | -1 |
| 23 | Dichloromethane | 75-09-2 | 1.43E+01 | 0 | 0 | 1 | 1 |
| 24 | Trichloromethane | 67-66-3 | 3.12E-01 | 0 | -1 | -1 | -1 |
| 25 | Tetrachloromethane | 56-23-5 | 3.71E-01 | 0 | 0 | -1 | -1 |
| 26 | Bromoform | 75-25-2 | 5.88E+01 | 0 | 0 | -1 | 1 |
| 27 | Bromomethane | 74-83-9 | 2.97E+00 | 0 | 0 | -1 | -1 |
| 28 | 1,2-Dichloroethane | 107-06-2 | 4.06E-01 | 0 | 0 | -1 | -1 |
| 29 | 1,1,1-Trichloroethane | 71-55-6 | 1.19E+03 | 1 | 1 | 1 | 1 |
| 30 | 1,1,2-Trichloroethane | 79-00-5 | 1.09E+00 | 0 | 0 | -1 | -1 |
| 31 | Chloroethene | 75-01-4 | 4.26E-01 | 1 | 0 | 1 | 1 |
| 32 | 1,1-Dichloroethene | 75-35-4 | 7.72E-02 | -2 | -2 | -1 | -1 |
| 33 | Trichloroethene | 79-01-6 | 5.17E+00 | 0 | 0 | 1 | 1 |
| 34 | Tetrachloroethene | 127-18-4 | 1.19E+01 | 1 | 1 | 1 | 1 |
| 35 | Benzene | 71-43-2 | 8.87E-01 | 0 | 0 | 1 | -1 |
| 36 | Toluene | 108-88-3 | 3.80E+01 | 0 | 0 | -1 | -1 |
| 37 | Chlorobenzene | 108-90-7 | 1.19E+00 | -1 | -1 | -1 | -1 |
| 38 | 1,2-Dichlorobenzene | 95-50-1 | 2.79E+02 | 0 | 1 | 1 | 1 |
| 39 | 1,3-Dichlorobenzene | 541-73-1 | 7.04E+01 | 1 | 1 | 1 | 1 |
| 40 | 1,4-Dichlorobenzene | 106-46-7 | 2.66E+01 | 1 | 1 | 1 | 1 |
| 41 | 1,2,4Trichlorobenzene | 120-82-1 | 3.91E+02 | 1 | 1 | 1 | 1 |
| 42 | Hexachlorobenzene | 118-74-1 | 3.99E-01 | 0 | 0 | -1 | -1 |
| 43 | Nitrobenzene | 98-95-3 | 8.41E+00 | 0 | 0 | -1 | -1 |
| 44 | Ethylbenzene | 100-41-4 | 3.95E+02 | 1 | 1 | 1 | 1 |
| 45 | Styrene | 100-42-5 | 3.84E+02 | 1 | 1 | 1 | 1 |
| 46 | Xylenes | 1330-20-7 | 3.18E+02 | 1 | 1 | 1 | 1 |
| 47 | Cresol | 1319-77-3 | 3.91E+02 | 1 | 1 | 1 | 1 |
| 48 | Phenol | 108-95-2 | 4.69E+04 | 3 | 3 | 1 | 1 |
| 49 | 2-Chlorophenol | 95-57-8 | 3.91E+02 | 1 | 1 | 1 | 1 |
| 50 | 2,4-Dichlorophenol | 120-83-2 | 2.35E+02 | 1 | 2 | 1 | 1 |
| 51 | 2,4,5-Trichlorophenol | 95-95-4 | 7.82E+03 | 1 | 2 | 1 | 1 |
| 52 | 2,4,6-Trichlorophenol | 88-06-2 | 5.81E+01 | 1 | 1 | 1 | 1 |
| 53 | 2,3,4,6-Tetrahlorophenol | 58-90-2 | 2.35E+03 | 1 | 2 | 1 | 1 |
| 54 | Pentachlorophenol | 87-86-5 | 2.66E+00 | 0 | 0 | -1 | -1 |
| 55 | Benz(a)anthracene | 56-55-3 | 8.75E-01 | 0 | 0 | -1 | -1 |
| 56 | Benzo(a)pyrene | 50-32-8 | 8.75E-02 | -1 | -1 | -1 | -1 |
| 57 | Benzo(b)fluoranthene | 205-99-2 | 8.75E-01 | 0 | 0 | -1 | -1 |
| 58 | Benzo(k)fluoranthene | 207-08-9 | 8.75E+00 | 0 | 0 | 1 | 1 |
| 59 | Chrysene | 218-01-9 | 8.75E+01 | 1 | 1 | 1 | 1 |
| 60 | Dibena(a,h)anthracene | 53-70-3 | 8.75E-02 | -1 | -1 | -1 | -1 |
| 61 | Indeno(1,2,3-c,d)pyrebe | 193-39-5 | 8.75E-01 | 0 | 0 | -1 | -1 |
| 62 | Naphthalene | 91-20-3 | 1.94E+02 | 1 | 2 | 1 | 1 |
| 63 | Acenaphehene | 83-32-9 | 4.69E+03 | 2 | 2 | 1 | 1 |
| 64 | Acenaphthylene | 208-96-8 | 4.69E+03 | 2 | 2 | 1 | 1 |
| 65 | Anthracene | 120-12-7 | 2.35E+04 | 3 | 2 | 1 | 1 |
| 66 | Benzo(g,h,i) perylene | 191-24-2 | 2.35E+03 | 2 | 2 | 1 | 1 |
| 67 | Flouranthene | 206-44-0 | 3.13E+03 | 3 | 2 | 1 | 1 |
| 68 | Flouorene | 86-73-7 | 3.13E+03 | 2 | 2 | 1 | 1 |
| 69 | Phenanthrene | 85-01-8 | 2.35E+03 | 2 | 2 | 1 | 1 |
| 70 | Pyrene | 129-00-0 | 2.35E+03 | 1 | 1 | 1 | 1 |
| 71 | Aldrin | 309-00-2 | 3.76E-02 | 0 | -1 | -1 | -1 |
| 72 | Chlordane | 57-74-9 | 1.82E+00 | 0 | 0 | -1 | -1 |
| 73 | DDT | 50-29-3 | 1.88E+00 | 0 | 0 | -1 | -1 |
| 74 | Dieldrin | 60-57-1 | 3.99E-02 | 0 | -1 | -1 | -1 |
| 75 | Endosulfan | 115-29-7 | 4.69E+02 | 1 | 2 | 1 | 1 |
| 76 | Endrin | 72-20-8 | 2.35E+01 | 1 | 1 | 1 | 1 |
| 77 | Heptachlor | 76-44-8 | 1.27E-01 | 0 | 0 | -1 | -1 |
| 78 | Lindane | 58-89-9 | 4.91E-01 | 0 | 0 | -1 | -1 |
| 79 | Toxaphene | 8001-35-2 | 5.81E-01 | 0 | 0 | -1 | -1 |
| 80 | 2,4-D | 94-75-7 | 7.82E+02 | 0 | 1 | 1 | 1 |
| 81 | Atrazine | 1912-24-9 | 2.88E+00 | 0 | 0 | 1 | -1 |
| 82 | Carbaryl | 63-25-2 | 7.82E+03 | 0 | 2 | 1 | 1 |
| 83 | Carbofuran | 1563-66-2 | 3.91E+02 | 0 | 1 | 1 | 1 |
| 84 | Chloryrifos | 2921-88-2 | 2.35E+02 | 1 | 1 | 1 | 1 |
| 85 | Diuron | 330-54-1 | 1.56E+02 | 0 | 1 | 1 | 1 |
| 86 | Gylphosate | 1071-83-6 | 7.82E+03 | 0 | 1 | 1 | 1 |
| 87 | Malathion | 121-75-5 | 1.56E+03 | 0 | 1 | 1 | 1 |
| 88 | MCPA | 94-74-6 | 3.91E+01 | 0 | 1 | 1 | 1 |
| 90 | Simazine | 122-34-9 | 5.32E+00 | 0 | 0 | 1 | -1 |
| 92 | Total PCB | 1336-36-3 | 1.00E+00 | 0 | 0 | 1 | 1 |
| 94 | Total PCDD/PCDF | 1746-01-6 | 4.26E-06 | -1 | -1 | -1 | -1 |
| 95 | Methyl ethyl ketone | 78-93-3 | 8.45E+01 | -2 | -1 | -1 | -1 |
| 96 | Methyl isobutyl ketone | 108-10-1 | 6.26E+03 | 0 | 1 | 1 | 1 |
| 97 | Dibutyl phthalate | 84-74-2 | 2.28E+03 | 0 | 1 | -1 | 1 |
| 98 | DEHP | 117-81-7 | 4.56E+01 | 0 | 0 | -1 | -1 |
| 99 | Hexachlorobutadine | 87-68-3 | 8.82E-02 | -2 | -1 | -1 | -1 |
| 100 | Methyl tert-butyl ether | 1634-04-4 | 3.91E+03 | 2 | 2 | 1 | 1 |
|  |  | Overall | C1 | C2 | C3 | C4 | C5 |
|  |  | Scores | 97 | 0.57 | 0.69 | 33 | 31 |
|  |  |  |  |  | No. >0 | 65 | 64 |
|  |  |  |  |  | No. <0 | 32 | 33 |

**Table S24a** Missouri Dept. of Natural Resources – Table SB-2

| Number | Pollutant | CAS. No | RGV (mg/kg) | C_2_ | C_3_ | C_4_ | C_5_ |
| --- | --- | --- | --- | --- | --- | --- | --- |
| 1 | Antimony | 7440-36-0 | 3.04E+01 | 0 | 1 | 1 | 1 |
| 2 | Arsenic | 7440-38-2 | 3.89E+00 | 0 | 0 | -1 | -1 |
| 3 | Barium | 7440-39-3 | 1.50E+04 | 1 | 1 | 1 | 1 |
| 4 | Beryllium | 7440-41-7 | 7.37E-01 | -1 | -1 | -1 | -1 |
| 5 | Cadmium | 7440-43-9 | 1.68E+01 | 1 | 1 | 1 | 1 |
| 6 | Chromium (III) | 16065-83-1 | 7.46E+04 | 3 | 2 | 1 | 1 |
| 7 | Chromium (VI) | 18540-29-9 | 1.47E-01 | -3 | -2 | -1 | -1 |
| 9 | Copper | 7440-50-8 | 3.04E+03 | 1 | 1 | 1 | 1 |
| 10 | Lead | 7439-92-1 | 2.60E+02 | 0 | 0 | -1 | 1 |
| 11 | Manganese | 7439-96-5 | 9.68E+03 | 1 | 1 | 1 | 1 |
| 12 | Mercury | 7439-97-6 | 4.63E+01 | 1 | 1 | 1 | 1 |
| 13 | Molybdenum | 7439-98-7 | 3.80E+02 | 1 | 1 | 1 | 1 |
| 14 | Nickel | 7440-02-0 | 1.51E+03 | 1 | 1 | 1 | 1 |
| 15 | Selenium | 7782-49-2 | 3.80E+02 | 1 | 1 | 1 | 1 |
| 16 | Silver | 7440-22-4 | 3.74E+02 | 1 | 1 | 1 | 1 |
| 19 | Vanadium | 7440-62-2 | 5.30E+02 | 1 | 1 | 1 | 1 |
| 20 | Zinc | 7440-66-6 | 2.28E+04 | 2 | 2 | 1 | 1 |
| 21 | Cyanide | 57-12-5 | 1.22E+03 | 2 | 2 | 1 | 1 |
| 22 | Acrylonitril | 107-13-1 | 1.15E+01 | 1 | 1 | 1 | 1 |
| 23 | Dichloromethane | 75-09-2 | 8.42E+02 | 2 | 2 | 1 | 1 |
| 24 | Trichloromethane | 67-66-3 | 1.80E+02 | 2 | 2 | 1 | 1 |
| 25 | Tetrachloromethane | 56-23-5 | 4.81E+01 | 2 | 2 | 1 | 1 |
| 26 | Bromoform | 75-25-2 | 6.02E+02 | 1 | 1 | 1 | 1 |
| 27 | Bromomethane | 74-83-9 | 9.59E+01 | 1 | 1 | 1 | 1 |
| 29 | 1,1,1-Trichloroethane | 71-55-6 | 2.06E+04 | 2 | 2 | 1 | 1 |
| 30 | 1,1,2-Trichloroethane | 79-00-5 | 1.06E+02 | 2 | 2 | 1 | 1 |
| 31 | Chloroethene | 75-01-4 | 4.56E+00 | 2 | 1 | 1 | 1 |
| 32 | 1,1-Dichloroethene | 75-35-4 | 3.47E+03 | 3 | 3 | 1 | 1 |
| 33 | Trichloroethene | 79-01-6 | 4.77E+02 | 2 | 2 | 1 | 1 |
| 34 | Tetrachloroethene | 127-18-4 | 1.18E+01 | 1 | 1 | 1 | 1 |
| 35 | Benzene | 71-43-2 | 1.77E+02 | 3 | 2 | 1 | 1 |
| 36 | Toluene | 108-88-3 | 6.21E+03 | 2 | 2 | 1 | 1 |
| 37 | Chlorobenzene | 108-90-7 | 1.34E+03 | 2 | 2 | 1 | 1 |
| 38 | 1,2-Dichlorobenzene | 95-50-1 | 5.73E+03 | 2 | 2 | 1 | 1 |
| 39 | 1,3-Dichlorobenzene | 541-73-1 | 2.05E+03 | 2 | 2 | 1 | 1 |
| 40 | 1,4-Dichlorobenzene | 106-46-7 | 9.66E+02 | 2 | 2 | 1 | 1 |
| 41 | 1,2,4Trichlorobenzene | 120-82-1 | 3.20E+02 | 1 | 1 | 1 | 1 |
| 42 | Hexachlorobenzene | 118-74-1 | 2.91E+00 | 1 | 1 | 1 | 1 |
| 43 | Nitrobenzene | 98-95-3 | 3.47E+01 | 1 | 1 | 1 | 1 |
| 44 | Ethylbenzene | 100-41-4 | 7.45E+03 | 3 | 3 | 1 | 1 |
| 45 | Styrene | 100-42-5 | 1.42E+04 | 2 | 2 | 1 | 1 |
| 46 | Xylenes | 1330-20-7 | 7.83E+03 | 2 | 2 | 1 | 1 |
| 47 | Cresol | 1319-77-3 | 2.74E+02 | 0 | 1 | 1 | 1 |
| 48 | Phenol | 108-95-2 | 1.17E+04 | 2 | 2 | 1 | 1 |
| 49 | 2-Chlorophenol | 95-57-8 | 3.42E+02 | 1 | 1 | 1 | 1 |
| 50 | 2,4-Dichlorophenol | 120-83-2 | 1.68E+02 | 1 | 1 | 1 | 1 |
| 51 | 2,4,5-Trichlorophenol | 95-95-4 | 5.92E+03 | 1 | 2 | 1 | 1 |
| 52 | 2,4,6-Trichlorophenol | 88-06-2 | 5.61E+00 | 0 | 0 | -1 | -1 |
| 54 | Pentachlorophenol | 87-86-5 | 2.97E+01 | 1 | 1 | 1 | 1 |
| 55 | Benz(a)anthracene | 56-55-3 | 6.20E+00 | 1 | 1 | 1 | 1 |
| 56 | Benzo(a)pyrene | 50-32-8 | 6.20E-01 | 0 | 0 | -1 | -1 |
| 57 | Benzo(b)fluoranthene | 205-99-2 | 6.19E+00 | 1 | 1 | 1 | 1 |
| 58 | Benzo(k)fluoranthene | 207-08-9 | 6.20E+01 | 1 | 1 | 1 | 1 |
| 59 | Chrysene | 218-01-9 | 5.99E+02 | 1 | 2 | 1 | 1 |
| 60 | Dibena(a,h)anthracene | 53-70-3 | 6.20E-01 | 0 | 0 | 1 | -1 |
| 61 | Indeno(1,2,3-c,d)pyrebe | 193-39-5 | 3.77E+00 | 0 | 0 | 1 | 1 |
| 62 | Naphthalene | 91-20-3 | 3.63E+01 | 0 | 1 | 1 | 1 |
| 63 | Acenaphehene | 83-32-9 | 3.13E+03 | 2 | 2 | 1 | 1 |
| 64 | Acenaphthylene | 208-96-8 | 4.18E+03 | 2 | 2 | 1 | 1 |
| 65 | Anthracene | 120-12-7 | 1.57E+04 | 2 | 2 | 1 | 1 |
| 66 | Benzo(g,h,i) perylene | 191-24-2 | 1.72E+03 | 2 | 2 | 1 | 1 |
| 67 | Flouranthene | 206-44-0 | 2.28E+03 | 3 | 2 | 1 | 1 |
| 68 | Flouorene | 86-73-7 | 2.20E+03 | 1 | 1 | 1 | 1 |
| 69 | Phenanthrene | 85-01-8 | 2.17E+03 | 2 | 2 | 1 | 1 |
| 70 | Pyrene | 129-00-0 | 1.71E+03 | 1 | 1 | 1 | 1 |
| 71 | Aldrin | 309-00-2 | 2.85E-01 | 1 | 0 | 1 | 1 |
| 73 | DDT | 50-29-3 | 1.43E+01 | 1 | 1 | 1 | 1 |
| 74 | Dieldrin | 60-57-1 | 3.01E-01 | 1 | 0 | 1 | 1 |
| 75 | Endosulfan | 115-29-7 | 3.57E+02 | 1 | 2 | 1 | 1 |
| 76 | Endrin | 72-20-8 | 1.83E+01 | 1 | 1 | 1 | 1 |
| 77 | Heptachlor | 76-44-8 | 1.04E+00 | 1 | 1 | 1 | 1 |
| 78 | Lindane | 58-89-9 | 4.25E+00 | 1 | 1 | 1 | 1 |
| 79 | Toxaphene | 8001-35-2 | 4.41E+00 | 1 | 1 | 1 | 1 |
| 80 | 2,4-D | 94-75-7 | 6.11E+02 | 0 | 1 | -1 | 1 |
| 81 | Atrazine | 1912-24-9 | 2.11E+01 | 1 | 1 | 1 | 1 |
| 82 | Carbaryl | 63-25-2 | 5.54E+03 | 0 | 2 | -1 | 1 |
| 83 | Carbofuran | 1563-66-2 | 3.06E+02 | 0 | 1 | 1 | 1 |
| 84 | Chloryrifos | 2921-88-2 | 1.83E+02 | 0 | 1 | 1 | 1 |
| 85 | Diuron | 330-54-1 | 1.22E+02 | 0 | 1 | 1 | 1 |
| 86 | Gylphosate | 1071-83-6 | 6.09E+03 | 0 | 1 | -1 | 1 |
| 87 | Malathion | 121-75-5 | 1.22E+03 | 0 | 1 | 1 | 1 |
| 88 | MCPA | 94-74-6 | 3.06E+01 | 0 | 0 | -1 | 1 |
| 89 | Picloram | 1918-02-1 | 3.84E+03 | 0 | 1 | -1 | 1 |
| 90 | Simazine | 122-34-9 | 4.05E+01 | 1 | 1 | 1 | 1 |
| 91 | Trifluralin | 1582-09-8 | 4.50E+02 | 1 | 1 | 1 | 1 |
| 92 | Total PCB | 1336-36-3 | 2.20E+00 | 1 | 1 | 1 | 1 |
| 95 | Methyl ethyl ketone | 78-93-3 | 5.46E+02 | -1 | 0 | -1 | -1 |
| 96 | Methyl isobutyl ketone | 108-10-1 | 6.17E+03 | 0 | 1 | 1 | 1 |
| 97 | Dibutyl phthalate | 84-74-2 | 6.11E+03 | 0 | 1 | 1 | 1 |
| 98 | DEHP | 117-81-7 | 3.47E+02 | 1 | 1 | 1 | 1 |
| 99 | Hexachlorobutadine | 87-68-3 | 1.65E+01 | 1 | 1 | 1 | 1 |
| 100 | Methyl tert-butyl ether | 1634-04-4 | 3.45E+03 | 2 | 2 | 1 | 1 |
|  |  | Overall | C1 | C2 | C3 | C4 | C5 |
|  |  | Scores | 92 | 1.05 | 1.20 | 68 | 78 |
|  |  |  |  |  | No. >0 | 80 | 85 |
|  |  |  |  |  | No. <0 | 12 | 7 |

**Table S24b** Missouri Dept. of Natural Resources – Table SB-3

| Number | Pollutant | CAS. No | RGV (mg/kg) | C_2_ | C_3_ | C_4_ | C_5_ |
| --- | --- | --- | --- | --- | --- | --- | --- |
| 1 | Antimony | 7440-36-0 | 3.04E+01 | 0 | 1 | 1 | 1 |
| 2 | Arsenic | 7440-38-2 | 3.89E+00 | 0 | 0 | -1 | -1 |
| 3 | Barium | 7440-39-3 | 1.50E+04 | 1 | 1 | 1 | 1 |
| 4 | Beryllium | 7440-41-7 | 7.37E-01 | -1 | -1 | -1 | -1 |
| 5 | Cadmium | 7440-43-9 | 1.68E+01 | 1 | 1 | 1 | 1 |
| 6 | Chromium (III) | 16065-83-1 | 7.46E+04 | 3 | 2 | 1 | 1 |
| 7 | Chromium (VI) | 18540-29-9 | 1.47E-01 | -3 | -2 | -1 | -1 |
| 9 | Copper | 7440-50-8 | 3.04E+03 | 1 | 1 | 1 | 1 |
| 10 | Lead | 7439-92-1 | 2.60E+02 | 0 | 0 | -1 | 1 |
| 11 | Manganese | 7439-96-5 | 9.68E+03 | 1 | 1 | 1 | 1 |
| 12 | Mercury | 7439-97-6 | 4.63E+01 | 1 | 1 | 1 | 1 |
| 13 | Molybdenum | 7439-98-7 | 3.80E+02 | 1 | 1 | 1 | 1 |
| 14 | Nickel | 7440-02-0 | 1.51E+03 | 1 | 1 | 1 | 1 |
| 15 | Selenium | 7782-49-2 | 3.80E+02 | 1 | 1 | 1 | 1 |
| 16 | Silver | 7440-22-4 | 3.74E+02 | 1 | 1 | 1 | 1 |
| 19 | Vanadium | 7440-62-2 | 5.30E+02 | 1 | 1 | 1 | 1 |
| 20 | Zinc | 7440-66-6 | 2.28E+04 | 2 | 2 | 1 | 1 |
| 21 | Cyanide | 57-12-5 | 1.22E+03 | 2 | 2 | 1 | 1 |
| 22 | Acrylonitril | 107-13-1 | 1.15E+01 | 1 | 1 | 1 | 1 |
| 23 | Dichloromethane | 75-09-2 | 8.42E+02 | 2 | 2 | 1 | 1 |
| 24 | Trichloromethane | 67-66-3 | 1.80E+02 | 2 | 2 | 1 | 1 |
| 25 | Tetrachloromethane | 56-23-5 | 4.81E+01 | 2 | 2 | 1 | 1 |
| 26 | Bromoform | 75-25-2 | 6.02E+02 | 1 | 1 | 1 | 1 |
| 27 | Bromomethane | 74-83-9 | 9.59E+01 | 1 | 1 | 1 | 1 |
| 29 | 1,1,1-Trichloroethane | 71-55-6 | 2.06E+04 | 2 | 2 | 1 | 1 |
| 30 | 1,1,2-Trichloroethane | 79-00-5 | 1.06E+02 | 2 | 2 | 1 | 1 |
| 31 | Chloroethene | 75-01-4 | 4.56E+00 | 2 | 1 | 1 | 1 |
| 32 | 1,1-Dichloroethene | 75-35-4 | 3.47E+03 | 3 | 3 | 1 | 1 |
| 33 | Trichloroethene | 79-01-6 | 4.77E+02 | 2 | 2 | 1 | 1 |
| 34 | Tetrachloroethene | 127-18-4 | 1.18E+01 | 1 | 1 | 1 | 1 |
| 35 | Benzene | 71-43-2 | 1.77E+02 | 3 | 2 | 1 | 1 |
| 36 | Toluene | 108-88-3 | 6.21E+03 | 2 | 2 | 1 | 1 |
| 37 | Chlorobenzene | 108-90-7 | 1.34E+03 | 2 | 2 | 1 | 1 |
| 38 | 1,2-Dichlorobenzene | 95-50-1 | 5.73E+03 | 2 | 2 | 1 | 1 |
| 39 | 1,3-Dichlorobenzene | 541-73-1 | 2.05E+03 | 2 | 2 | 1 | 1 |
| 40 | 1,4-Dichlorobenzene | 106-46-7 | 9.66E+02 | 2 | 2 | 1 | 1 |
| 41 | 1,2,4Trichlorobenzene | 120-82-1 | 3.20E+02 | 1 | 1 | 1 | 1 |
| 42 | Hexachlorobenzene | 118-74-1 | 2.91E+00 | 1 | 1 | 1 | 1 |
| 43 | Nitrobenzene | 98-95-3 | 3.47E+01 | 1 | 1 | 1 | 1 |
| 44 | Ethylbenzene | 100-41-4 | 7.45E+03 | 3 | 3 | 1 | 1 |
| 45 | Styrene | 100-42-5 | 1.42E+04 | 2 | 2 | 1 | 1 |
| 46 | Xylenes | 1330-20-7 | 7.83E+03 | 2 | 2 | 1 | 1 |
| 47 | Cresol | 1319-77-3 | 2.75E+02 | 0 | 1 | 1 | 1 |
| 48 | Phenol | 108-95-2 | 1.18E+04 | 2 | 2 | 1 | 1 |
| 49 | 2-Chlorophenol | 95-57-8 | 3.42E+02 | 1 | 1 | 1 | 1 |
| 50 | 2,4-Dichlorophenol | 120-83-2 | 1.70E+02 | 1 | 1 | 1 | 1 |
| 51 | 2,4,5-Trichlorophenol | 95-95-4 | 5.96E+03 | 1 | 2 | 1 | 1 |
| 52 | 2,4,6-Trichlorophenol | 88-06-2 | 5.72E+00 | 0 | 0 | -1 | -1 |
| 54 | Pentachlorophenol | 87-86-5 | 2.97E+01 | 1 | 1 | 1 | 1 |
| 55 | Benz(a)anthracene | 56-55-3 | 6.20E+00 | 1 | 1 | 1 | 1 |
| 56 | Benzo(a)pyrene | 50-32-8 | 6.20E-01 | 0 | 0 | -1 | -1 |
| 57 | Benzo(b)fluoranthene | 205-99-2 | 6.19E+00 | 1 | 1 | 1 | 1 |
| 58 | Benzo(k)fluoranthene | 207-08-9 | 6.20E+01 | 1 | 1 | 1 | 1 |
| 59 | Chrysene | 218-01-9 | 6.05E+02 | 1 | 2 | 1 | 1 |
| 60 | Dibena(a,h)anthracene | 53-70-3 | 6.20E-01 | 0 | 0 | 1 | -1 |
| 61 | Indeno(1,2,3-c,d)pyrebe | 193-39-5 | 3.77E+00 | 0 | 0 | 1 | 1 |
| 62 | Naphthalene | 91-20-3 | 3.63E+01 | 0 | 1 | 1 | 1 |
| 63 | Acenaphehene | 83-32-9 | 3.21E+03 | 2 | 2 | 1 | 1 |
| 64 | Acenaphthylene | 208-96-8 | 4.31E+03 | 2 | 2 | 1 | 1 |
| 65 | Anthracene | 120-12-7 | 1.61E+04 | 2 | 2 | 1 | 1 |
| 66 | Benzo(g,h,i) perylene | 191-24-2 | 1.72E+03 | 2 | 2 | 1 | 1 |
| 67 | Flouranthene | 206-44-0 | 2.28E+03 | 3 | 2 | 1 | 1 |
| 68 | Flouorene | 86-73-7 | 2.22E+03 | 1 | 1 | 1 | 1 |
| 69 | Phenanthrene | 85-01-8 | 2.22E+03 | 2 | 2 | 1 | 1 |
| 70 | Pyrene | 129-00-0 | 1.71E+03 | 1 | 1 | 1 | 1 |
| 71 | Aldrin | 309-00-2 | 2.85E-01 | 1 | 0 | 1 | 1 |
| 73 | DDT | 50-29-3 | 1.43E+01 | 1 | 1 | 1 | 1 |
| 74 | Dieldrin | 60-57-1 | 3.01E-01 | 1 | 0 | 1 | 1 |
| 75 | Endosulfan | 115-29-7 | 3.59E+02 | 1 | 2 | 1 | 1 |
| 76 | Endrin | 72-20-8 | 1.83E+01 | 1 | 1 | 1 | 1 |
| 77 | Heptachlor | 76-44-8 | 1.04E+00 | 1 | 1 | 1 | 1 |
| 78 | Lindane | 58-89-9 | 4.28E+00 | 1 | 1 | 1 | 1 |
| 79 | Toxaphene | 8001-35-2 | 4.41E+00 | 1 | 1 | 1 | 1 |
| 80 | 2,4-D | 94-75-7 | 6.11E+02 | 0 | 1 | -1 | 1 |
| 81 | Atrazine | 1912-24-9 | 2.11E+01 | 1 | 1 | 1 | 1 |
| 82 | Carbaryl | 63-25-2 | 5.54E+03 | 0 | 2 | -1 | 1 |
| 83 | Carbofuran | 1563-66-2 | 3.06E+02 | 0 | 1 | 1 | 1 |
| 84 | Chloryrifos | 2921-88-2 | 1.83E+02 | 0 | 1 | 1 | 1 |
| 85 | Diuron | 330-54-1 | 1.22E+02 | 0 | 1 | 1 | 1 |
| 86 | Gylphosate | 1071-83-6 | 6.06E+03 | 0 | 1 | -1 | 1 |
| 87 | Malathion | 121-75-5 | 1.22E+03 | 0 | 1 | 1 | 1 |
| 88 | MCPA | 94-74-6 | 3.06E+01 | 0 | 0 | -1 | 1 |
| 89 | Picloram | 1918-02-1 | 3.84E+03 | 0 | 1 | -1 | 1 |
| 90 | Simazine | 122-34-9 | 4.05E+01 | 1 | 1 | 1 | 1 |
| 91 | Trifluralin | 1582-09-8 | 4.52E+02 | 1 | 1 | 1 | 1 |
| 92 | Total PCB | 1336-36-3 | 2.21E+00 | 1 | 1 | 1 | 1 |
| 95 | Methyl ethyl ketone | 78-93-3 | 5.46E+02 | -1 | 0 | -1 | -1 |
| 96 | Methyl isobutyl ketone | 108-10-1 | 6.17E+03 | 0 | 1 | 1 | 1 |
| 97 | Dibutyl phthalate | 84-74-2 | 6.11E+03 | 0 | 1 | 1 | 1 |
| 98 | DEHP | 117-81-7 | 3.47E+02 | 1 | 1 | 1 | 1 |
| 99 | Hexachlorobutadine | 87-68-3 | 1.65E+01 | 1 | 1 | 1 | 1 |
| 100 | Methyl tert-butyl ether | 1634-04-4 | 3.45E+03 | 2 | 2 | 1 | 1 |
|  |  | Overall | C1 | C2 | C3 | C4 | C5 |
|  |  | Scores | 92 | 1.05 | 1.20 | 68 | 78 |
|  |  |  |  |  | No. >0 | 80 | 85 |
|  |  |  |  |  | No. <0 | 12 | 7 |

**Table S24c** Missouri Dept. of Natural Resources – Table SB-4

| Number | Pollutant | CAS. No | RGV (mg/kg) | C_2_ | C_3_ | C_4_ | C_5_ |
| --- | --- | --- | --- | --- | --- | --- | --- |
| 1 | Antimony | 7440-36-0 | 3.04E+01 | 0 | 1 | 1 | 1 |
| 2 | Arsenic | 7440-38-2 | 3.89E+00 | 0 | 0 | -1 | -1 |
| 3 | Barium | 7440-39-3 | 1.50E+04 | 1 | 1 | 1 | 1 |
| 4 | Beryllium | 7440-41-7 | 7.37E-01 | -1 | -1 | -1 | -1 |
| 5 | Cadmium | 7440-43-9 | 1.68E+01 | 1 | 1 | 1 | 1 |
| 6 | Chromium (III) | 16065-83-1 | 7.46E+04 | 3 | 2 | 1 | 1 |
| 7 | Chromium (VI) | 18540-29-9 | 1.47E-01 | -3 | -2 | -1 | -1 |
| 9 | Copper | 7440-50-8 | 3.04E+03 | 1 | 1 | 1 | 1 |
| 10 | Lead | 7439-92-1 | 2.60E+02 | 0 | 0 | -1 | 1 |
| 11 | Manganese | 7439-96-5 | 9.68E+03 | 1 | 1 | 1 | 1 |
| 12 | Mercury | 7439-97-6 | 4.63E+01 | 1 | 1 | 1 | 1 |
| 13 | Molybdenum | 7439-98-7 | 3.80E+02 | 1 | 1 | 1 | 1 |
| 14 | Nickel | 7440-02-0 | 1.51E+03 | 1 | 1 | 1 | 1 |
| 15 | Selenium | 7782-49-2 | 3.80E+02 | 1 | 1 | 1 | 1 |
| 16 | Silver | 7440-22-4 | 3.74E+02 | 1 | 1 | 1 | 1 |
| 19 | Vanadium | 7440-62-2 | 5.30E+02 | 1 | 1 | 1 | 1 |
| 20 | Zinc | 7440-66-6 | 2.28E+04 | 2 | 2 | 1 | 1 |
| 21 | Cyanide | 57-12-5 | 1.22E+03 | 2 | 2 | 1 | 1 |
| 22 | Acrylonitril | 107-13-1 | 1.15E+01 | 1 | 1 | 1 | 1 |
| 23 | Dichloromethane | 75-09-2 | 8.42E+02 | 2 | 2 | 1 | 1 |
| 24 | Trichloromethane | 67-66-3 | 1.80E+02 | 2 | 2 | 1 | 1 |
| 25 | Tetrachloromethane | 56-23-5 | 4.81E+01 | 2 | 2 | 1 | 1 |
| 26 | Bromoform | 75-25-2 | 6.02E+02 | 1 | 1 | 1 | 1 |
| 27 | Bromomethane | 74-83-9 | 9.59E+01 | 1 | 1 | 1 | 1 |
| 29 | 1,1,1-Trichloroethane | 71-55-6 | 2.06E+04 | 2 | 2 | 1 | 1 |
| 30 | 1,1,2-Trichloroethane | 79-00-5 | 1.06E+02 | 2 | 2 | 1 | 1 |
| 31 | Chloroethene | 75-01-4 | 4.56E+00 | 2 | 1 | 1 | 1 |
| 32 | 1,1-Dichloroethene | 75-35-4 | 3.47E+03 | 3 | 3 | 1 | 1 |
| 33 | Trichloroethene | 79-01-6 | 4.77E+02 | 2 | 2 | 1 | 1 |
| 34 | Tetrachloroethene | 127-18-4 | 1.18E+01 | 1 | 1 | 1 | 1 |
| 35 | Benzene | 71-43-2 | 1.77E+02 | 3 | 2 | 1 | 1 |
| 36 | Toluene | 108-88-3 | 6.21E+03 | 2 | 2 | 1 | 1 |
| 37 | Chlorobenzene | 108-90-7 | 1.34E+03 | 2 | 2 | 1 | 1 |
| 38 | 1,2-Dichlorobenzene | 95-50-1 | 5.73E+03 | 2 | 2 | 1 | 1 |
| 39 | 1,3-Dichlorobenzene | 541-73-1 | 2.05E+03 | 2 | 2 | 1 | 1 |
| 40 | 1,4-Dichlorobenzene | 106-46-7 | 9.66E+02 | 2 | 2 | 1 | 1 |
| 41 | 1,2,4Trichlorobenzene | 120-82-1 | 3.20E+02 | 1 | 1 | 1 | 1 |
| 42 | Hexachlorobenzene | 118-74-1 | 2.91E+00 | 1 | 1 | 1 | 1 |
| 43 | Nitrobenzene | 98-95-3 | 3.47E+01 | 1 | 1 | 1 | 1 |
| 44 | Ethylbenzene | 100-41-4 | 7.45E+03 | 3 | 3 | 1 | 1 |
| 45 | Styrene | 100-42-5 | 1.42E+04 | 2 | 2 | 1 | 1 |
| 46 | Xylenes | 1330-20-7 | 7.83E+03 | 2 | 2 | 1 | 1 |
| 47 | Cresol | 1319-77-3 | 2.74E+02 | 0 | 1 | 1 | 1 |
| 48 | Phenol | 108-95-2 | 1.15E+04 | 2 | 2 | 1 | 1 |
| 49 | 2-Chlorophenol | 95-57-8 | 3.42E+02 | 1 | 1 | 1 | 1 |
| 50 | 2,4-Dichlorophenol | 120-83-2 | 1.70E+02 | 1 | 1 | 1 | 1 |
| 51 | 2,4,5-Trichlorophenol | 95-95-4 | 5.97E+03 | 1 | 2 | 1 | 1 |
| 52 | 2,4,6-Trichlorophenol | 88-06-2 | 5.77E+00 | 0 | 0 | -1 | -1 |
| 54 | Pentachlorophenol | 87-86-5 | 2.97E+01 | 1 | 1 | 1 | 1 |
| 55 | Benz(a)anthracene | 56-55-3 | 6.20E+00 | 1 | 1 | 1 | 1 |
| 56 | Benzo(a)pyrene | 50-32-8 | 6.20E-01 | 0 | 0 | -1 | -1 |
| 57 | Benzo(b)fluoranthene | 205-99-2 | 6.20E+00 | 1 | 1 | 1 | 1 |
| 58 | Benzo(k)fluoranthene | 207-08-9 | 6.20E+01 | 1 | 1 | 1 | 1 |
| 59 | Chrysene | 218-01-9 | 6.08E+02 | 1 | 2 | 1 | 1 |
| 60 | Dibena(a,h)anthracene | 53-70-3 | 6.20E-01 | 0 | 0 | 1 | -1 |
| 61 | Indeno(1,2,3-c,d)pyrebe | 193-39-5 | 3.77E+00 | 0 | 0 | 1 | 1 |
| 62 | Naphthalene | 91-20-3 | 3.63E+01 | 0 | 1 | 1 | 1 |
| 63 | Acenaphehene | 83-32-9 | 3.26E+03 | 2 | 2 | 1 | 1 |
| 64 | Acenaphthylene | 208-96-8 | 4.39E+03 | 2 | 2 | 1 | 1 |
| 65 | Anthracene | 120-12-7 | 1.64E+04 | 2 | 2 | 1 | 1 |
| 66 | Benzo(g,h,i) perylene | 191-24-2 | 1.72E+03 | 2 | 2 | 1 | 1 |
| 67 | Flouranthene | 206-44-0 | 2.28E+03 | 3 | 2 | 1 | 1 |
| 68 | Flouorene | 86-73-7 | 2.24E+03 | 1 | 1 | 1 | 1 |
| 69 | Phenanthrene | 85-01-8 | 2.25E+03 | 2 | 2 | 1 | 1 |
| 70 | Pyrene | 129-00-0 | 1.71E+03 | 1 | 1 | 1 | 1 |
| 71 | Aldrin | 309-00-2 | 2.85E-01 | 1 | 0 | 1 | 1 |
| 73 | DDT | 50-29-3 | 1.43E+01 | 1 | 1 | 1 | 1 |
| 74 | Dieldrin | 60-57-1 | 3.02E-01 | 1 | 0 | 1 | 1 |
| 75 | Endosulfan | 115-29-7 | 3.60E+02 | 1 | 2 | 1 | 1 |
| 76 | Endrin | 72-20-8 | 1.83E+01 | 1 | 1 | 1 | 1 |
| 77 | Heptachlor | 76-44-8 | 1.04E+00 | 1 | 1 | 1 | 1 |
| 78 | Lindane | 58-89-9 | 4.29E+00 | 1 | 1 | 1 | 1 |
| 79 | Toxaphene | 8001-35-2 | 4.41E+00 | 1 | 1 | 1 | 1 |
| 80 | 2,4-D | 94-75-7 | 6.11E+02 | 0 | 1 | -1 | 1 |
| 81 | Atrazine | 1912-24-9 | 2.11E+01 | 1 | 1 | 1 | 1 |
| 82 | Carbaryl | 63-25-2 | 5.54E+03 | 0 | 2 | -1 | 1 |
| 83 | Carbofuran | 1563-66-2 | 3.06E+02 | 0 | 1 | 1 | 1 |
| 84 | Chloryrifos | 2921-88-2 | 1.83E+02 | 0 | 1 | 1 | 1 |
| 85 | Diuron | 330-54-1 | 1.22E+02 | 0 | 1 | 1 | 1 |
| 86 | Gylphosate | 1071-83-6 | 6.04E+03 | 0 | 1 | -1 | 1 |
| 87 | Malathion | 121-75-5 | 1.22E+03 | 0 | 1 | 1 | 1 |
| 88 | MCPA | 94-74-6 | 3.06E+01 | 0 | 0 | -1 | 1 |
| 89 | Picloram | 1918-02-1 | 3.84E+03 | 0 | 1 | -1 | 1 |
| 90 | Simazine | 122-34-9 | 4.05E+01 | 1 | 1 | 1 | 1 |
| 91 | Trifluralin | 1582-09-8 | 4.53E+02 | 1 | 1 | 1 | 1 |
| 92 | Total PCB | 1336-36-3 | 2.21E+00 | 1 | 1 | 1 | 1 |
| 95 | Methyl ethyl ketone | 78-93-3 | 5.46E+02 | -1 | 0 | -1 | -1 |
| 96 | Methyl isobutyl ketone | 108-10-1 | 6.17E+03 | 0 | 1 | 1 | 1 |
| 97 | Dibutyl phthalate | 84-74-2 | 6.11E+03 | 0 | 1 | 1 | 1 |
| 98 | DEHP | 117-81-7 | 3.47E+02 | 1 | 1 | 1 | 1 |
| 99 | Hexachlorobutadine | 87-68-3 | 1.65E+01 | 1 | 1 | 1 | 1 |
| 100 | Methyl tert-butyl ether | 1634-04-4 | 3.45E+03 | 2 | 2 | 1 | 1 |
|  |  | Overall | C1 | C2 | C3 | C4 | C5 |
|  |  | Scores | 92 | 1.05 | 1.20 | 68 | 78 |
|  |  |  |  |  | No. >0 | 80 | 85 |
|  |  |  |  |  | No. <0 | 12 | 7 |

**Table S25** Nebraska Dept. of Environmental Quality

| Number | Pollutant | CAS. No | RGV (mg/kg) | C_2_ | C_3_ | C_4_ | C_5_ |
| --- | --- | --- | --- | --- | --- | --- | --- |
| 1 | Antimony | 7440-36-0 | 7.80E+00 | 0 | 0 | -1 | -1 |
| 2 | Arsenic | 7440-38-2 | 3.90E-01 | -1 | -1 | -1 | -1 |
| 3 | Barium | 7440-39-3 | 3.80E+03 | 1 | 1 | 1 | 1 |
| 4 | Beryllium | 7440-41-7 | 3.90E+01 | 1 | 0 | 1 | 1 |
| 5 | Cadmium | 7440-43-9 | 1.80E+01 | 1 | 1 | 1 | 1 |
| 6 | Chromium (III) | 16065-83-1 | 2.90E+04 | 2 | 2 | 1 | 1 |
| 7 | Chromium (VI) | 18540-29-9 | 2.90E-01 | -2 | -2 | -1 | -1 |
| 8 | Cobalt | 7440-48-4 | 5.80E+00 | -1 | -1 | -1 | -1 |
| 9 | Copper | 7440-50-8 | 7.80E+02 | 1 | 1 | 1 | 1 |
| 10 | Lead | 7439-92-1 | 4.00E+02 | 0 | 1 | 1 | 1 |
| 11 | Manganese | 7439-96-5 | 4.60E+02 | 0 | 0 | -1 | -1 |
| 12 | Mercury | 7439-97-6 | 3.10E+00 | 0 | 0 | -1 | -1 |
| 13 | Molybdenum | 7439-98-7 | 7.60E+01 | 0 | 0 | 1 | 1 |
| 14 | Nickel | 7440-02-0 | 3.90E+02 | 1 | 1 | 1 | 1 |
| 15 | Selenium | 7782-49-2 | 9.80E+01 | 1 | 1 | 1 | 1 |
| 16 | Silver | 7440-22-4 | 9.80E+01 | 1 | 1 | 1 | 1 |
| 18 | Tin | 7440-31-5 | 1.20E+04 | 2 | 2 | 1 | 1 |
| 19 | Vanadium | 7440-62-2 | 9.70E+01 | 0 | 0 | -1 | -1 |
| 20 | Zinc | 7440-66-6 | 5.90E+03 | 1 | 1 | 1 | 1 |
| 21 | Cyanide | 57-12-5 | 3.90E+02 | 1 | 1 | 1 | 1 |
| 22 | Acrylonitril | 107-13-1 | 2.70E-01 | -1 | -1 | -1 | -1 |
| 23 | Dichloromethane | 75-09-2 | 1.20E+01 | 0 | 0 | 1 | -1 |
| 24 | Trichloromethane | 67-66-3 | 3.50E-01 | 0 | -1 | -1 | -1 |
| 25 | Tetrachloromethane | 56-23-5 | 7.20E+01 | 2 | 2 | 1 | 1 |
| 26 | Bromoform | 75-25-2 | 6.10E+01 | 0 | 0 | -1 | 1 |
| 27 | Bromomethane | 74-83-9 | 2.20E+00 | 0 | 0 | -1 | -1 |
| 28 | 1,2-Dichloroethane | 107-06-2 | 5.10E-01 | 0 | 0 | -1 | -1 |
| 29 | 1,1,1-Trichloroethane | 71-55-6 | 2.60E+03 | 1 | 1 | 1 | 1 |
| 30 | 1,1,2-Trichloroethane | 79-00-5 | 1.30E+00 | 0 | 0 | -1 | -1 |
| 31 | Chloroethene | 75-01-4 | 6.30E-02 | 0 | 0 | -1 | -1 |
| 32 | 1,1-Dichloroethene | 75-35-4 | 7.20E+01 | 1 | 1 | 1 | 1 |
| 33 | Trichloroethene | 79-01-6 | 1.30E+00 | 0 | 0 | -1 | -1 |
| 34 | Tetrachloroethene | 127-18-4 | 2.50E+01 | 1 | 1 | 1 | 1 |
| 35 | Benzene | 71-43-2 | 1.30E+00 | 0 | 0 | 1 | 1 |
| 36 | Toluene | 108-88-3 | 1.30E+03 | 1 | 1 | 1 | 1 |
| 37 | Chlorobenzene | 108-90-7 | 8.50E+01 | 0 | 1 | 1 | 1 |
| 38 | 1,2-Dichlorobenzene | 95-50-1 | 5.40E+02 | 1 | 1 | 1 | 1 |
| 40 | 1,4-Dichlorobenzene | 106-46-7 | 2.90E+00 | 0 | 0 | -1 | -1 |
| 41 | 1,2,4Trichlorobenzene | 120-82-1 | 1.80E+01 | 0 | 0 | -1 | -1 |
| 42 | Hexachlorobenzene | 118-74-1 | 3.00E-01 | 0 | 0 | -1 | -1 |
| 43 | Nitrobenzene | 98-95-3 | 5.70E+00 | 0 | 0 | -1 | -1 |
| 44 | Ethylbenzene | 100-41-4 | 6.30E+00 | 0 | 0 | -1 | -1 |
| 45 | Styrene | 100-42-5 | 1.70E+03 | 2 | 2 | 1 | 1 |
| 46 | Xylenes | 1330-20-7 | 1.90E+02 | 0 | 0 | 1 | 1 |
| 47 | Cresol | 1319-77-3 | 7.60E+01 | 0 | 0 | -1 | 1 |
| 48 | Phenol | 108-95-2 | 4.60E+03 | 2 | 2 | 1 | 1 |
| 49 | 2-Chlorophenol | 95-57-8 | 9.80E+01 | 1 | 1 | 1 | 1 |
| 50 | 2,4-Dichlorophenol | 120-83-2 | 4.60E+01 | 1 | 1 | 1 | 1 |
| 51 | 2,4,5-Trichlorophenol | 95-95-4 | 1.50E+03 | 1 | 1 | 1 | 1 |
| 52 | 2,4,6-Trichlorophenol | 88-06-2 | 1.50E+01 | 0 | 0 | 1 | 1 |
| 53 | 2,3,4,6-Tetrahlorophenol | 58-90-2 | 4.60E+02 | 1 | 1 | 1 | 1 |
| 54 | Pentachlorophenol | 87-86-5 | 3.00E+00 | 0 | 0 | -1 | -1 |
| 55 | Benz(a)anthracene | 56-55-3 | 1.50E-01 | -1 | -1 | -1 | -1 |
| 56 | Benzo(a)pyrene | 50-32-8 | 1.50E-02 | -1 | -1 | -1 | -1 |
| 57 | Benzo(b)fluoranthene | 205-99-2 | 1.50E-01 | -1 | -1 | -1 | -1 |
| 58 | Benzo(k)fluoranthene | 207-08-9 | 1.50E+00 | -1 | 0 | -1 | -1 |
| 59 | Chrysene | 218-01-9 | 1.50E+01 | 0 | 0 | -1 | -1 |
| 60 | Dibena(a,h)anthracene | 53-70-3 | 1.50E-02 | -1 | -1 | -1 | -1 |
| 61 | Indeno(1,2,3-c,d)pyrebe | 193-39-5 | 1.50E-01 | -1 | -1 | -1 | -1 |
| 62 | Naphthalene | 91-20-3 | 4.30E+00 | 0 | 0 | -1 | 1 |
| 63 | Acenaphehene | 83-32-9 | 1.20E+03 | 1 | 1 | 1 | 1 |
| 65 | Anthracene | 120-12-7 | 5.90E+03 | 2 | 2 | 1 | 1 |
| 67 | Flouranthene | 206-44-0 | 5.70E+02 | 2 | 1 | 1 | 1 |
| 68 | Flouorene | 86-73-7 | 7.80E+02 | 1 | 1 | 1 | 1 |
| 70 | Pyrene | 129-00-0 | 4.30E+02 | 1 | 1 | 1 | 1 |
| 71 | Aldrin | 309-00-2 | 2.90E-02 | 0 | -1 | -1 | -1 |
| 72 | Chlordane | 57-74-9 | 1.80E+00 | 0 | 0 | -1 | -1 |
| 73 | DDT | 50-29-3 | 1.70E+00 | 0 | 0 | -1 | -1 |
| 74 | Dieldrin | 60-57-1 | 3.00E-02 | 0 | -1 | -1 | -1 |
| 75 | Endosulfan | 115-29-7 | 9.20E+01 | 1 | 1 | 1 | 1 |
| 76 | Endrin | 72-20-8 | 4.60E+00 | 0 | 0 | -1 | 1 |
| 77 | Heptachlor | 76-44-8 | 1.10E-01 | 0 | 0 | -1 | -1 |
| 78 | Lindane | 58-89-9 | 5.20E-01 | 0 | 0 | -1 | 1 |
| 79 | Toxaphene | 8001-35-2 | 4.40E-01 | 0 | 0 | -1 | -1 |
| 80 | 2,4-D | 94-75-7 | 1.70E+02 | 0 | 0 | -1 | -1 |
| 81 | Atrazine | 1912-24-9 | 2.10E+00 | 0 | 0 | -1 | -1 |
| 82 | Carbaryl | 63-25-2 | 1.50E+03 | 0 | 1 | -1 | 1 |
| 83 | Carbofuran | 1563-66-2 | 7.60E+01 | 0 | 1 | -1 | 1 |
| 84 | Chloryrifos | 2921-88-2 | 4.60E+01 | 0 | 0 | -1 | -1 |
| 85 | Diuron | 330-54-1 | 3.10E+01 | 0 | 0 | -1 | -1 |
| 86 | Gylphosate | 1071-83-6 | 1.50E+03 | 0 | 0 | -1 | 1 |
| 87 | Malathion | 121-75-5 | 3.10E+02 | 0 | 0 | -1 | -1 |
| 88 | MCPA | 94-74-6 | 7.60E+00 | 0 | 0 | -1 | -1 |
| 89 | Picloram | 1918-02-1 | 1.10E+03 | 0 | 0 | -1 | -1 |
| 90 | Simazine | 122-34-9 | 4.00E+00 | 0 | 0 | -1 | -1 |
| 92 | Total PCB | 1336-36-3 | 2.20E-01 | 0 | 0 | -1 | -1 |
| 94 | Total PCDD/PCDF | 1746-01-6 | 3.70E-06 | -1 | -1 | -1 | -1 |
| 95 | Methyl ethyl ketone | 78-93-3 | 7.50E+03 | 0 | 1 | 1 | 1 |
| 96 | Methyl isobutyl ketone | 108-10-1 | 1.40E+03 | 0 | 1 | -1 | 1 |
| 97 | Dibutyl phthalate | 84-74-2 | 1.50E+03 | 0 | 0 | -1 | 1 |
| 98 | DEHP | 117-81-7 | 3.50E+01 | 0 | 0 | -1 | -1 |
| 99 | Hexachlorobutadine | 87-68-3 | 6.20E+00 | 0 | 0 | -1 | 1 |
| 100 | Methyl tert-butyl ether | 1634-04-4 | 5.10E+01 | 0 | 0 | -1 | 1 |
|  |  | Overall | C1 | C2 | C3 | C4 | C5 |
|  |  | Scores | 93 | 0.26 | 0.28 | -19 | 3 |
|  |  |  |  |  | No. >0 | 37 | 48 |
|  |  |  |  |  | No. <0 | 56 | 45 |

**Table S26a** Nevada Dept. of Environmental Protection - Discovery Events

| Number | Pollutant | CAS. No | RGV (mg/kg) | C_2_ | C_3_ | C_4_ | C_5_ |
| --- | --- | --- | --- | --- | --- | --- | --- |
| 1 | Antimony | 7440-36-0 | 5.00E+00 | 0 | 0 | -1 | -1 |
| 2 | Arsenic | 7440-38-2 | 3.90E-01 | -1 | -1 | -1 | -1 |
| 3 | Barium | 7440-39-3 | 1.60E+03 | 1 | 0 | 1 | 1 |
| 4 | Beryllium | 7440-41-7 | 6.30E+01 | 1 | 1 | 1 | 1 |
| 5 | Cadmium | 7440-43-9 | 8.00E+00 | 0 | 0 | 1 | 1 |
| 6 | Chromium (III) | 16065-83-1 | 1.20E+05 | 3 | 2 | 1 | 1 |
| 7 | Chromium (VI) | 18540-29-9 | 3.80E+01 | 0 | 0 | -1 | -1 |
| 8 | Cobalt | 7440-48-4 | 2.30E+01 | 0 | 0 | -1 | -1 |
| 9 | Copper | 7440-50-8 | 3.10E+03 | 1 | 1 | 1 | 1 |
| 10 | Lead | 7439-92-1 | 4.00E+02 | 0 | 1 | 1 | 1 |
| 11 | Manganese | 7439-96-5 | 1.80E+03 | 0 | 0 | 1 | 1 |
| 12 | Mercury | 7439-97-6 | 6.70E+00 | 1 | 1 | 1 | 1 |
| 13 | Molybdenum | 7439-98-7 | 3.90E+02 | 1 | 1 | 1 | 1 |
| 14 | Nickel | 7440-02-0 | 1.30E+02 | 0 | 0 | 1 | -1 |
| 15 | Selenium | 7782-49-2 | 5.00E+00 | -1 | -1 | -1 | -1 |
| 16 | Silver | 7440-22-4 | 3.40E+01 | 0 | 0 | -1 | -1 |
| 17 | Thallium | 7440-28-0 | 5.10E+00 | 0 | 0 | 1 | 1 |
| 18 | Tin | 7440-31-5 | 4.70E+04 | 3 | 2 | 1 | 1 |
| 19 | Vanadium | 7440-62-2 | 3.90E+02 | 1 | 1 | 1 | 1 |
| 20 | Zinc | 7440-66-6 | 1.20E+04 | 2 | 1 | 1 | 1 |
| 21 | Cyanide | 57-12-5 | 1.60E+03 | 2 | 2 | 1 | 1 |
| 22 | Acrylonitril | 107-13-1 | 2.40E-01 | -1 | -1 | -1 | -1 |
| 23 | Dichloromethane | 75-09-2 | 2.00E-02 | -2 | -3 | -1 | -1 |
| 24 | Trichloromethane | 67-66-3 | 3.00E-01 | 0 | -1 | -1 | -1 |
| 25 | Tetrachloromethane | 56-23-5 | 7.00E-02 | -1 | -1 | -1 | -1 |
| 26 | Bromoform | 75-25-2 | 8.00E-01 | -2 | -2 | -1 | -1 |
| 27 | Bromomethane | 74-83-9 | 2.00E-01 | -1 | -1 | -1 | -1 |
| 28 | 1,2-Dichloroethane | 107-06-2 | 2.00E-02 | -2 | -2 | -1 | -1 |
| 29 | 1,1,1-Trichloroethane | 71-55-6 | 2.00E+00 | -2 | -2 | -1 | -1 |
| 30 | 1,1,2-Trichloroethane | 79-00-5 | 2.00E-02 | -2 | -2 | -1 | -1 |
| 31 | Chloroethene | 75-01-4 | 1.00E-02 | -1 | -1 | -1 | -1 |
| 32 | 1,1-Dichloroethene | 75-35-4 | 6.00E-02 | -2 | -2 | -1 | -1 |
| 33 | Trichloroethene | 79-01-6 | 6.00E-02 | -2 | -1 | -1 | -1 |
| 35 | Benzene | 71-43-2 | 3.00E-02 | -1 | -1 | -1 | -1 |
| 36 | Toluene | 108-88-3 | 1.20E+01 | -1 | -1 | -1 | -1 |
| 37 | Chlorobenzene | 108-90-7 | 1.00E+00 | -2 | -1 | -1 | -1 |
| 38 | 1,2-Dichlorobenzene | 95-50-1 | 1.70E+01 | -1 | 0 | -1 | -1 |
| 40 | 1,4-Dichlorobenzene | 106-46-7 | 2.00E+00 | 0 | 0 | -1 | -1 |
| 41 | 1,2,4Trichlorobenzene | 120-82-1 | 5.00E+00 | 0 | 0 | -1 | -1 |
| 42 | Hexachlorobenzene | 118-74-1 | 3.00E-01 | 0 | 0 | -1 | -1 |
| 43 | Nitrobenzene | 98-95-3 | 1.00E-01 | -2 | -2 | -1 | -1 |
| 44 | Ethylbenzene | 100-41-4 | 5.70E+00 | 0 | -1 | -1 | -1 |
| 45 | Styrene | 100-42-5 | 4.00E+00 | -1 | -1 | -1 | -1 |
| 47 | Cresol | 1319-77-3 | 3.10E+02 | 0 | 1 | 1 | 1 |
| 48 | Phenol | 108-95-2 | 1.00E+02 | 0 | 0 | -1 | -1 |
| 49 | 2-Chlorophenol | 95-57-8 | 4.00E+00 | -1 | 0 | -1 | -1 |
| 50 | 2,4-Dichlorophenol | 120-83-2 | 1.00E+00 | -1 | -1 | -1 | -1 |
| 51 | 2,4,5-Trichlorophenol | 95-95-4 | 2.70E+02 | 0 | 0 | -1 | 1 |
| 52 | 2,4,6-Trichlorophenol | 88-06-2 | 2.00E-01 | -2 | -2 | -1 | -1 |
| 54 | Pentachlorophenol | 87-86-5 | 3.00E-02 | -2 | -2 | -1 | -1 |
| 55 | Benz(a)anthracene | 56-55-3 | 1.50E-01 | -1 | -1 | -1 | -1 |
| 56 | Benzo(a)pyrene | 50-32-8 | 1.50E-02 | -1 | -1 | -1 | -1 |
| 57 | Benzo(b)fluoranthene | 205-99-2 | 1.50E-01 | -1 | -1 | -1 | -1 |
| 58 | Benzo(k)fluoranthene | 207-08-9 | 1.50E+00 | -1 | 0 | -1 | -1 |
| 59 | Chrysene | 218-01-9 | 1.50E+01 | 0 | 0 | -1 | -1 |
| 60 | Dibena(a,h)anthracene | 53-70-3 | 1.50E-02 | -1 | -1 | -1 | -1 |
| 61 | Indeno(1,2,3-c,d)pyrebe | 193-39-5 | 1.50E-01 | -1 | -1 | -1 | -1 |
| 62 | Naphthalene | 91-20-3 | 3.90E+00 | 0 | 0 | -1 | 1 |
| 63 | Acenaphehene | 83-32-9 | 5.70E+02 | 1 | 1 | 1 | 1 |
| 65 | Anthracene | 120-12-7 | 1.20E+04 | 2 | 2 | 1 | 1 |
| 67 | Flouranthene | 206-44-0 | 2.30E+03 | 3 | 2 | 1 | 1 |
| 68 | Flouorene | 86-73-7 | 5.60E+02 | 1 | 1 | 1 | 1 |
| 70 | Pyrene | 129-00-0 | 1.70E+03 | 1 | 1 | 1 | 1 |
| 71 | Aldrin | 309-00-2 | 2.90E-02 | 0 | -1 | -1 | -1 |
| 72 | Chlordane | 57-74-9 | 1.60E+00 | 0 | 0 | -1 | -1 |
| 73 | DDT | 50-29-3 | 1.70E+00 | 0 | 0 | -1 | -1 |
| 74 | Dieldrin | 60-57-1 | 4.00E-03 | -1 | -2 | -1 | -1 |
| 75 | Endosulfan | 115-29-7 | 1.80E+01 | 0 | 0 | -1 | 1 |
| 76 | Endrin | 72-20-8 | 1.00E+00 | 0 | 0 | -1 | -1 |
| 77 | Heptachlor | 76-44-8 | 1.10E-01 | 0 | 0 | -1 | -1 |
| 78 | Lindane | 58-89-9 | 9.00E-03 | -2 | -2 | -1 | -1 |
| 79 | Toxaphene | 8001-35-2 | 4.40E-01 | 0 | 0 | -1 | -1 |
| 80 | 2,4-D | 94-75-7 | 6.90E+02 | 0 | 1 | -1 | 1 |
| 81 | Atrazine | 1912-24-9 | 2.10E+00 | 0 | 0 | -1 | -1 |
| 82 | Carbaryl | 63-25-2 | 6.10E+03 | 0 | 2 | 1 | 1 |
| 83 | Carbofuran | 1563-66-2 | 3.10E+02 | 0 | 1 | 1 | 1 |
| 84 | Chloryrifos | 2921-88-2 | 1.80E+02 | 0 | 1 | 1 | 1 |
| 85 | Diuron | 330-54-1 | 1.20E+02 | 0 | 1 | -1 | 1 |
| 86 | Gylphosate | 1071-83-6 | 6.10E+03 | 0 | 1 | -1 | 1 |
| 87 | Malathion | 121-75-5 | 1.20E+03 | 0 | 1 | -1 | 1 |
| 88 | MCPA | 94-74-6 | 3.10E+01 | 0 | 0 | -1 | 1 |
| 89 | Picloram | 1918-02-1 | 4.30E+03 | 0 | 1 | -1 | 1 |
| 90 | Simazine | 122-34-9 | 4.00E+00 | 0 | 0 | -1 | -1 |
| 91 | Trifluralin | 1582-09-8 | 6.30E+01 | 0 | 0 | -1 | -1 |
| 92 | Total PCB | 1336-36-3 | 2.40E-01 | 0 | 0 | -1 | -1 |
| 93 | PCB 118 | 31508-00-6 | 1.10E-01 | 0 | 0 | -1 | -1 |
| 94 | Total PCDD/PCDF | 1746-01-6 | 4.50E-06 | -1 | -1 | -1 | -1 |
| 95 | Methyl ethyl ketone | 78-93-3 | 2.80E+04 | 1 | 2 | 1 | 1 |
| 96 | Methyl isobutyl ketone | 108-10-1 | 5.30E+03 | 0 | 1 | 1 | 1 |
| 97 | Dibutyl phthalate | 84-74-2 | 2.30E+03 | 0 | 1 | -1 | 1 |
| 98 | DEHP | 117-81-7 | 3.50E+01 | 0 | 0 | -1 | -1 |
| 99 | Hexachlorobutadine | 87-68-3 | 2.00E+00 | 0 | 0 | -1 | -1 |
| 100 | Methyl tert-butyl ether | 1634-04-4 | 3.90E+01 | 0 | 0 | -1 | -1 |
|  |  | Overall | C1 | C2 | C3 | C4 | C5 |
|  |  | Scores | 93 | -0.20 | -0.11 | -41 | -23 |
|  |  |  |  |  | No. >0 | 26 | 35 |
|  |  |  |  |  | No. <0 | 67 | 58 |

**Table S26b** Nevada - Basic Comparison Levels

| Number | Pollutant | CAS. No | RGV (mg/kg) | C_2_ | C_3_ | C_4_ | C_5_ |
| --- | --- | --- | --- | --- | --- | --- | --- |
| 1 | Antimony | 7440-36-0 | 3.13E+01 | 0 | 1 | 1 | 1 |
| 2 | Arsenic | 7440-38-2 | 4.24E-01 | -1 | -1 | -1 | -1 |
| 3 | Barium | 7440-39-3 | 1.53E+04 | 1 | 1 | 1 | 1 |
| 4 | Beryllium | 7440-41-7 | 1.55E+02 | 1 | 1 | 1 | 1 |
| 5 | Cadmium | 7440-43-9 | 7.77E+01 | 1 | 1 | 1 | 1 |
| 6 | Chromium (III) | 16065-83-1 | 1.00E+05 | 3 | 2 | 1 | 1 |
| 7 | Chromium (VI) | 18540-29-9 | 2.34E+02 | 1 | 1 | 1 | 1 |
| 8 | Cobalt | 7440-48-4 | 2.34E+01 | 0 | 0 | -1 | -1 |
| 9 | Copper | 7440-50-8 | 2.91E+03 | 1 | 1 | 1 | 1 |
| 11 | Manganese | 7439-96-5 | 1.82E+03 | 0 | 0 | 1 | 1 |
| 12 | Mercury | 7439-97-6 | 1.25E+01 | 1 | 1 | 1 | 1 |
| 13 | Molybdenum | 7439-98-7 | 3.91E+02 | 1 | 1 | 1 | 1 |
| 14 | Nickel | 7440-02-0 | 1.54E+03 | 1 | 1 | 1 | 1 |
| 15 | Selenium | 7782-49-2 | 3.91E+02 | 1 | 1 | 1 | 1 |
| 16 | Silver | 7440-22-4 | 3.91E+02 | 1 | 1 | 1 | 1 |
| 17 | Thallium | 7440-28-0 | 5.16E+00 | 0 | 0 | 1 | 1 |
| 18 | Tin | 7440-31-5 | 4.69E+04 | 3 | 2 | 1 | 1 |
| 19 | Vanadium | 7440-62-2 | 3.91E+02 | 1 | 1 | 1 | 1 |
| 20 | Zinc | 7440-66-6 | 2.35E+04 | 2 | 2 | 1 | 1 |
| 21 | Cyanide | 57-12-5 | 5.72E+00 | -1 | -1 | -1 | -1 |
| 22 | Acrylonitril | 107-13-1 | 2.89E-01 | -1 | -1 | -1 | -1 |
| 23 | Dichloromethane | 75-09-2 | 1.26E+01 | 0 | 0 | 1 | 1 |
| 24 | Trichloromethane | 67-66-3 | 3.53E-01 | 0 | -1 | -1 | -1 |
| 25 | Tetrachloromethane | 56-23-5 | 8.43E-01 | 0 | 0 | -1 | -1 |
| 26 | Bromoform | 75-25-2 | 6.74E+01 | 0 | 0 | -1 | 1 |
| 27 | Bromomethane | 74-83-9 | 8.70E+00 | 0 | 0 | 1 | 1 |
| 28 | 1,2-Dichloroethane | 107-06-2 | 4.98E-01 | 0 | 0 | -1 | -1 |
| 29 | 1,1,1-Trichloroethane | 71-55-6 | 1.39E+03 | 1 | 1 | 1 | 1 |
| 30 | 1,1,2-Trichloroethane | 79-00-5 | 1.20E+00 | 0 | 0 | -1 | -1 |
| 31 | Chloroethene | 75-01-4 | 3.28E-01 | 1 | 0 | 1 | 1 |
| 32 | 1,1-Dichloroethene | 75-35-4 | 2.85E+02 | 2 | 2 | 1 | 1 |
| 33 | Trichloroethene | 79-01-6 | 1.18E+00 | 0 | 0 | -1 | -1 |
| 34 | Tetrachloroethene | 127-18-4 | 6.97E-01 | -1 | 0 | -1 | -1 |
| 35 | Benzene | 71-43-2 | 9.30E-01 | 0 | 0 | 1 | -1 |
| 36 | Toluene | 108-88-3 | 5.21E+02 | 1 | 1 | 1 | 1 |
| 37 | Chlorobenzene | 108-90-7 | 2.73E+02 | 1 | 1 | 1 | 1 |
| 38 | 1,2-Dichlorobenzene | 95-50-1 | 3.73E+02 | 1 | 1 | 1 | 1 |
| 39 | 1,3-Dichlorobenzene | 541-73-1 | 2.14E+02 | 1 | 1 | 1 | 1 |
| 40 | 1,4-Dichlorobenzene | 106-46-7 | 2.97E+00 | 0 | 0 | -1 | -1 |
| 41 | 1,2,4Trichlorobenzene | 120-82-1 | 2.40E+01 | 0 | 0 | -1 | 1 |
| 42 | Hexachlorobenzene | 118-74-1 | 3.33E-01 | 0 | 0 | -1 | -1 |
| 43 | Nitrobenzene | 98-95-3 | 3.11E+00 | -1 | 0 | -1 | -1 |
| 44 | Ethylbenzene | 100-41-4 | 4.35E+00 | -1 | -1 | -1 | -1 |
| 45 | Styrene | 100-42-5 | 1.73E+03 | 2 | 2 | 1 | 1 |
| 46 | Xylenes | 1330-20-7 | 2.14E+02 | 0 | 0 | 1 | 1 |
| 47 | Cresol | 1319-77-3 | 3.08E+02 | 0 | 1 | 1 | 1 |
| 48 | Phenol | 108-95-2 | 1.85E+04 | 2 | 2 | 1 | 1 |
| 49 | 2-Chlorophenol | 95-57-8 | 2.20E+02 | 1 | 1 | 1 | 1 |
| 50 | 2,4-Dichlorophenol | 120-83-2 | 1.85E+02 | 1 | 1 | 1 | 1 |
| 51 | 2,4,5-Trichlorophenol | 95-95-4 | 6.16E+03 | 1 | 2 | 1 | 1 |
| 52 | 2,4,6-Trichlorophenol | 88-06-2 | 4.84E+01 | 1 | 1 | 1 | 1 |
| 53 | 2,3,4,6-Tetrahlorophenol | 58-90-2 | 1.85E+03 | 1 | 2 | 1 | 1 |
| 54 | Pentachlorophenol | 87-86-5 | 4.93E+01 | 1 | 1 | 1 | 1 |
| 55 | Benz(a)anthracene | 56-55-3 | 6.82E-01 | 0 | 0 | -1 | -1 |
| 56 | Benzo(a)pyrene | 50-32-8 | 6.82E-02 | -1 | -1 | -1 | -1 |
| 57 | Benzo(b)fluoranthene | 205-99-2 | 6.82E-01 | 0 | 0 | -1 | -1 |
| 58 | Benzo(k)fluoranthene | 207-08-9 | 6.82E+00 | 0 | 0 | -1 | -1 |
| 59 | Chrysene | 218-01-9 | 6.82E+01 | 1 | 1 | 1 | 1 |
| 60 | Dibena(a,h)anthracene | 53-70-3 | 6.82E-02 | -1 | -1 | -1 | -1 |
| 61 | Indeno(1,2,3-c,d)pyrebe | 193-39-5 | 6.82E-01 | 0 | 0 | -1 | -1 |
| 62 | Naphthalene | 91-20-3 | 3.58E+00 | -1 | 0 | -1 | 1 |
| 63 | Acenaphehene | 83-32-9 | 5.09E+02 | 1 | 1 | 1 | 1 |
| 64 | Acenaphthylene | 208-96-8 | 1.47E+02 | 1 | 1 | 1 | 1 |
| 65 | Anthracene | 120-12-7 | 2.00E+03 | 2 | 1 | 1 | 1 |
| 66 | Benzo(g,h,i) perylene | 191-24-2 | 2.35E+03 | 2 | 2 | 1 | 1 |
| 67 | Flouranthene | 206-44-0 | 2.32E+03 | 3 | 2 | 1 | 1 |
| 68 | Flouorene | 86-73-7 | 6.71E+02 | 1 | 1 | 1 | 1 |
| 69 | Phenanthrene | 85-01-8 | 2.45E+01 | 0 | 0 | -1 | -1 |
| 70 | Pyrene | 129-00-0 | 1.89E+03 | 1 | 1 | 1 | 1 |
| 71 | Aldrin | 309-00-2 | 3.13E-02 | 0 | -1 | -1 | -1 |
| 72 | Chlordane | 57-74-9 | 1.77E+00 | 0 | 0 | -1 | -1 |
| 73 | DDT | 50-29-3 | 1.87E+00 | 0 | 0 | -1 | -1 |
| 74 | Dieldrin | 60-57-1 | 3.33E-02 | 0 | -1 | -1 | -1 |
| 75 | Endosulfan | 115-29-7 | 3.70E+02 | 1 | 2 | 1 | 1 |
| 76 | Endrin | 72-20-8 | 1.85E+01 | 1 | 1 | 1 | 1 |
| 77 | Heptachlor | 76-44-8 | 1.18E-01 | 0 | 0 | -1 | -1 |
| 78 | Lindane | 58-89-9 | 7.06E-01 | 0 | 0 | 1 | 1 |
| 79 | Toxaphene | 8001-35-2 | 4.84E-01 | 0 | 0 | -1 | -1 |
| 80 | 2,4-D | 94-75-7 | 6.89E+02 | 0 | 1 | -1 | 1 |
| 81 | Atrazine | 1912-24-9 | 2.40E+00 | 0 | 0 | -1 | -1 |
| 82 | Carbaryl | 63-25-2 | 6.16E+03 | 0 | 2 | 1 | 1 |
| 83 | Carbofuran | 1563-66-2 | 3.08E+02 | 0 | 1 | 1 | 1 |
| 84 | Chloryrifos | 2921-88-2 | 1.85E+02 | 0 | 1 | 1 | 1 |
| 85 | Diuron | 330-54-1 | 1.23E+02 | 0 | 1 | 1 | 1 |
| 86 | Gylphosate | 1071-83-6 | 6.16E+03 | 0 | 1 | 1 | 1 |
| 87 | Malathion | 121-75-5 | 1.23E+03 | 0 | 1 | 1 | 1 |
| 88 | MCPA | 94-74-6 | 3.08E+01 | 0 | 0 | -1 | 1 |
| 90 | Simazine | 122-34-9 | 4.44E+02 | 2 | 2 | 1 | 1 |
| 92 | Total PCB | 1336-36-3 | 2.43E-01 | 0 | 0 | -1 | -1 |
| 94 | Total PCDD/PCDF | 1746-01-6 | 5.00E-05 | 0 | 0 | -1 | -1 |
| 95 | Methyl ethyl ketone | 78-93-3 | 3.21E+04 | 1 | 2 | 1 | 1 |
| 96 | Methyl isobutyl ketone | 108-10-1 | 5.80E+03 | 0 | 1 | 1 | 1 |
| 97 | Dibutyl phthalate | 84-74-2 | 6.16E+03 | 0 | 1 | 1 | 1 |
| 98 | DEHP | 117-81-7 | 3.80E+01 | 0 | 0 | -1 | -1 |
| 99 | Hexachlorobutadine | 87-68-3 | 6.83E+00 | 0 | 0 | 1 | 1 |
| 100 | Methyl tert-butyl ether | 1634-04-4 | 4.49E+01 | 0 | 0 | -1 | 1 |
|  |  | Overall | C1 | C2 | C3 | C4 | C5 |
|  |  | Scores | 96 | 0.47 | 0.58 | 22 | 32 |
|  |  |  |  |  | No. >0 | 59 | 64 |
|  |  |  |  |  | No. <0 | 37 | 32 |

**Table S27a** New Hampshire Dept. of Environmental Services - Appendix E NH S-1

| Number | Pollutant | CAS. No | RGV (mg/kg) | C_2_ | C_3_ | C_4_ | C_5_ |
| --- | --- | --- | --- | --- | --- | --- | --- |
| 1 | Antimony | 7440-36-0 | 9.00E+00 | 0 | 0 | -1 | -1 |
| 2 | Arsenic | 7440-38-2 | 1.10E+01 | 0 | 0 | -1 | -1 |
| 3 | Barium | 7440-39-3 | 1.00E+03 | 0 | 0 | 1 | -1 |
| 4 | Beryllium | 7440-41-7 | 1.20E+01 | 0 | 0 | -1 | -1 |
| 5 | Cadmium | 7440-43-9 | 3.30E+01 | 1 | 1 | 1 | 1 |
| 6 | Chromium (III) | 16065-83-1 | 1.00E+03 | 1 | 0 | 1 | 1 |
| 7 | Chromium (VI) | 18540-29-9 | 1.30E+02 | 0 | 1 | 1 | 1 |
| 10 | Lead | 7439-92-1 | 4.00E+02 | 0 | 1 | 1 | 1 |
| 11 | Manganese | 7439-96-5 | 1.00E+03 | 0 | 0 | -1 | -1 |
| 12 | Mercury | 7439-97-6 | 7.00E+00 | 1 | 1 | 1 | 1 |
| 14 | Nickel | 7440-02-0 | 4.00E+02 | 1 | 1 | 1 | 1 |
| 15 | Selenium | 7782-49-2 | 1.80E+02 | 1 | 1 | 1 | 1 |
| 16 | Silver | 7440-22-4 | 8.90E+01 | 0 | 0 | 1 | 1 |
| 17 | Thallium | 7440-28-0 | 1.00E+01 | 1 | 1 | 1 | 1 |
| 20 | Zinc | 7440-66-6 | 1.00E+03 | 0 | 0 | 1 | -1 |
| 21 | Cyanide | 57-12-5 | 2.20E+01 | 0 | 0 | -1 | -1 |
| 22 | Acrylonitril | 107-13-1 | 5.00E-01 | 0 | 0 | -1 | -1 |
| 23 | Dichloromethane | 75-09-2 | 1.00E-01 | -2 | -2 | -1 | -1 |
| 25 | Tetrachloromethane | 56-23-5 | 1.20E+01 | 1 | 1 | 1 | 1 |
| 26 | Bromoform | 75-25-2 | 1.00E-01 | -3 | -2 | -1 | -1 |
| 27 | Bromomethane | 74-83-9 | 3.00E-01 | -1 | -1 | -1 | -1 |
| 28 | 1,2-Dichloroethane | 107-06-2 | 1.00E-01 | -1 | -1 | -1 | -1 |
| 29 | 1,1,1-Trichloroethane | 71-55-6 | 7.80E+01 | -1 | 0 | -1 | -1 |
| 30 | 1,1,2-Trichloroethane | 79-00-5 | 1.00E-01 | -1 | -1 | -1 | -1 |
| 31 | Chloroethene | 75-01-4 | 1.00E+00 | 1 | 1 | 1 | 1 |
| 32 | 1,1-Dichloroethene | 75-35-4 | 1.40E+01 | 0 | 0 | 1 | 1 |
| 33 | Trichloroethene | 79-01-6 | 8.00E-01 | -1 | 0 | -1 | -1 |
| 34 | Tetrachloroethene | 127-18-4 | 2.00E+00 | 0 | 0 | -1 | -1 |
| 35 | Benzene | 71-43-2 | 3.00E-01 | 0 | 0 | -1 | -1 |
| 36 | Toluene | 108-88-3 | 1.00E+02 | 0 | 0 | -1 | -1 |
| 38 | 1,2-Dichlorobenzene | 95-50-1 | 8.80E+01 | 0 | 0 | -1 | 1 |
| 39 | 1,3-Dichlorobenzene | 541-73-1 | 1.50E+02 | 1 | 1 | 1 | 1 |
| 40 | 1,4-Dichlorobenzene | 106-46-7 | 7.00E+00 | 0 | 0 | 1 | -1 |
| 41 | 1,2,4Trichlorobenzene | 120-82-1 | 1.90E+01 | 0 | 0 | -1 | -1 |
| 42 | Hexachlorobenzene | 118-74-1 | 8.00E-01 | 0 | 0 | 1 | -1 |
| 44 | Ethylbenzene | 100-41-4 | 1.20E+02 | 1 | 1 | 1 | 1 |
| 45 | Styrene | 100-42-5 | 1.70E+01 | 0 | 0 | -1 | -1 |
| 46 | Xylenes | 1330-20-7 | 5.00E+02 | 1 | 1 | 1 | 1 |
| 47 | Cresol | 1319-77-3 | 7.00E-01 | -2 | -2 | -1 | -1 |
| 48 | Phenol | 108-95-2 | 5.60E+01 | 0 | 0 | -1 | -1 |
| 49 | 2-Chlorophenol | 95-57-8 | 2.00E+00 | -1 | -1 | -1 | -1 |
| 53 | 2,3,4,6-Tetrahlorophenol | 58-90-2 | 1.30E+02 | 0 | 0 | -1 | 1 |
| 54 | Pentachlorophenol | 87-86-5 | 3.00E+00 | 0 | 0 | -1 | -1 |
| 55 | Benz(a)anthracene | 56-55-3 | 1.00E+00 | 0 | 0 | -1 | -1 |
| 56 | Benzo(a)pyrene | 50-32-8 | 7.00E-01 | 0 | 0 | -1 | 1 |
| 57 | Benzo(b)fluoranthene | 205-99-2 | 1.00E+00 | 0 | 0 | -1 | -1 |
| 58 | Benzo(k)fluoranthene | 207-08-9 | 1.20E+01 | 0 | 0 | 1 | 1 |
| 59 | Chrysene | 218-01-9 | 1.20E+02 | 1 | 1 | 1 | 1 |
| 60 | Dibena(a,h)anthracene | 53-70-3 | 7.00E-01 | 0 | 0 | 1 | -1 |
| 61 | Indeno(1,2,3-c,d)pyrebe | 193-39-5 | 1.00E+00 | 0 | 0 | -1 | -1 |
| 62 | Naphthalene | 91-20-3 | 5.00E+00 | 0 | 0 | -1 | 1 |
| 63 | Acenaphehene | 83-32-9 | 3.40E+02 | 1 | 1 | 1 | 1 |
| 64 | Acenaphthylene | 208-96-8 | 4.90E+02 | 1 | 1 | 1 | 1 |
| 65 | Anthracene | 120-12-7 | 1.00E+03 | 1 | 1 | 1 | 1 |
| 67 | Flouranthene | 206-44-0 | 9.60E+02 | 2 | 1 | 1 | 1 |
| 68 | Flouorene | 86-73-7 | 7.70E+01 | 0 | 0 | -1 | -1 |
| 70 | Pyrene | 129-00-0 | 7.20E+02 | 1 | 1 | 1 | 1 |
| 71 | Aldrin | 309-00-2 | 9.00E-02 | 0 | 0 | -1 | -1 |
| 72 | Chlordane | 57-74-9 | 4.00E+00 | 0 | 0 | 1 | 1 |
| 73 | DDT | 50-29-3 | 4.00E+00 | 1 | 0 | 1 | 1 |
| 74 | Dieldrin | 60-57-1 | 6.00E-02 | 0 | 0 | -1 | -1 |
| 75 | Endosulfan | 115-29-7 | 4.50E+01 | 0 | 1 | 1 | 1 |
| 76 | Endrin | 72-20-8 | 8.00E+00 | 0 | 1 | 1 | 1 |
| 77 | Heptachlor | 76-44-8 | 2.00E-01 | 0 | 0 | -1 | -1 |
| 78 | Lindane | 58-89-9 | 9.00E-02 | -1 | -1 | -1 | -1 |
| 79 | Toxaphene | 8001-35-2 | 1.00E+00 | 0 | 0 | 1 | -1 |
| 80 | 2,4-D | 94-75-7 | 3.00E+02 | 0 | 0 | -1 | 1 |
| 81 | Atrazine | 1912-24-9 | 9.00E-02 | -1 | -2 | -1 | -1 |
| 83 | Carbofuran | 1563-66-2 | 6.00E-01 | -2 | -1 | -1 | -1 |
| 88 | MCPA | 94-74-6 | 1.30E+01 | 0 | 0 | -1 | -1 |
| 89 | Picloram | 1918-02-1 | 6.00E+00 | -3 | -2 | -1 | -1 |
| 90 | Simazine | 122-34-9 | 4.00E-01 | -1 | -1 | -1 | -1 |
| 92 | Total PCB | 1336-36-3 | 1.00E+00 | 0 | 0 | 1 | 1 |
| 94 | Total PCDD/PCDF | 1746-01-6 | 1.00E-03 | 1 | 1 | 1 | 1 |
| 95 | Methyl ethyl ketone | 78-93-3 | 5.10E+01 | -2 | -1 | -1 | -1 |
| 96 | Methyl isobutyl ketone | 108-10-1 | 2.90E+01 | -2 | -1 | -1 | -1 |
| 97 | Dibutyl phthalate | 84-74-2 | 2.60E+03 | -2 | -1 | -1 | 1 |
| 98 | DEHP | 117-81-7 | 7.20E+01 | 0 | 0 | -1 | -1 |
| 99 | Hexachlorobutadine | 87-68-3 | 1.70E+01 | 1 | 1 | 1 | 1 |
| 100 | Methyl tert-butyl ether | 1634-04-4 | 2.00E-01 | -2 | -2 | -1 | -1 |
|  |  | Overall | C1 | C2 | C3 | C4 | C5 |
|  |  | Scores | 80 | -0.1 | 0 | -10 | -10 |
|  |  |  |  |  | No. >0 | 35 | 35 |
|  |  |  |  |  | No. <0 | 45 | 45 |

**Table S27b** New Hampshire Dept. of Environmental Services - Appendix E Risk S-1

| Number | Pollutant | CAS. No | RGV (mg/kg) | C_2_ | C_3_ | C_4_ | C_5_ |
| --- | --- | --- | --- | --- | --- | --- | --- |
| 1 | Antimony | 7440-36-0 | 9.00E+00 | 0 | 0 | -1 | -1 |
| 2 | Arsenic | 7440-38-2 | 1.00E+00 | -1 | -1 | -1 | -1 |
| 3 | Barium | 7440-39-3 | 4.00E+03 | 1 | 1 | 1 | 1 |
| 4 | Beryllium | 7440-41-7 | 1.20E+01 | 0 | 0 | -1 | -1 |
| 5 | Cadmium | 7440-43-9 | 3.30E+01 | 1 | 1 | 1 | 1 |
| 6 | Chromium (III) | 16065-83-1 | 5.60E+04 | 3 | 2 | 1 | 1 |
| 7 | Chromium (VI) | 18540-29-9 | 3.00E+00 | -1 | -1 | -1 | -1 |
| 11 | Manganese | 7439-96-5 | 5.20E+03 | 1 | 1 | 1 | 1 |
| 12 | Mercury | 7439-97-6 | 7.00E+00 | 1 | 1 | 1 | 1 |
| 14 | Nickel | 7440-02-0 | 4.00E+02 | 1 | 1 | 1 | 1 |
| 15 | Selenium | 7782-49-2 | 1.80E+02 | 1 | 1 | 1 | 1 |
| 16 | Silver | 7440-22-4 | 8.90E+01 | 0 | 0 | 1 | 1 |
| 17 | Thallium | 7440-28-0 | 4.00E-01 | -1 | -1 | -1 | -1 |
| 20 | Zinc | 7440-66-6 | 1.06E+04 | 1 | 1 | 1 | 1 |
| 21 | Cyanide | 57-12-5 | 2.20E+01 | 0 | 0 | -1 | -1 |
| 22 | Acrylonitril | 107-13-1 | 3.00E+00 | 0 | 0 | 1 | 1 |
| 23 | Dichloromethane | 75-09-2 | 2.00E+02 | 2 | 1 | 1 | 1 |
| 25 | Tetrachloromethane | 56-23-5 | 2.20E+01 | 2 | 2 | 1 | 1 |
| 26 | Bromoform | 75-25-2 | 1.70E+02 | 1 | 1 | 1 | 1 |
| 27 | Bromomethane | 74-83-9 | 5.40E+01 | 1 | 1 | 1 | 1 |
| 28 | 1,2-Dichloroethane | 107-06-2 | 1.40E+01 | 1 | 1 | 1 | 1 |
| 29 | 1,1,1-Trichloroethane | 71-55-6 | 7.70E+04 | 2 | 3 | 1 | 1 |
| 30 | 1,1,2-Trichloroethane | 79-00-5 | 2.30E+01 | 1 | 1 | 1 | 1 |
| 31 | Chloroethene | 75-01-4 | 1.00E+00 | 1 | 1 | 1 | 1 |
| 32 | 1,1-Dichloroethene | 75-35-4 | 1.90E+03 | 2 | 3 | 1 | 1 |
| 33 | Trichloroethene | 79-01-6 | 1.70E+01 | 1 | 1 | 1 | 1 |
| 34 | Tetrachloroethene | 127-18-4 | 2.04E+02 | 2 | 2 | 1 | 1 |
| 35 | Benzene | 71-43-2 | 2.80E+01 | 2 | 2 | 1 | 1 |
| 36 | Toluene | 108-88-3 | 2.70E+03 | 2 | 2 | 1 | 1 |
| 38 | 1,2-Dichlorobenzene | 95-50-1 | 3.06E+03 | 1 | 2 | 1 | 1 |
| 40 | 1,4-Dichlorobenzene | 106-46-7 | 2.40E+02 | 2 | 2 | 1 | 1 |
| 41 | 1,2,4Trichlorobenzene | 120-82-1 | 4.50E+01 | 0 | 1 | 1 | 1 |
| 42 | Hexachlorobenzene | 118-74-1 | 8.00E-01 | 0 | 0 | 1 | -1 |
| 44 | Ethylbenzene | 100-41-4 | 1.20E+02 | 1 | 1 | 1 | 1 |
| 45 | Styrene | 100-42-5 | 6.80E+03 | 2 | 2 | 1 | 1 |
| 46 | Xylenes | 1330-20-7 | 6.80E+03 | 2 | 2 | 1 | 1 |
| 47 | Cresol | 1319-77-3 | 2.60E+03 | 1 | 2 | 1 | 1 |
| 48 | Phenol | 108-95-2 | 7.90E+03 | 2 | 2 | 1 | 1 |
| 49 | 2-Chlorophenol | 95-57-8 | 1.30E+02 | 1 | 1 | 1 | 1 |
| 53 | 2,3,4,6-Tetrahlorophenol | 58-90-2 | 7.90E+02 | 1 | 1 | 1 | 1 |
| 54 | Pentachlorophenol | 87-86-5 | 1.70E+00 | 0 | 0 | -1 | -1 |
| 55 | Benz(a)anthracene | 56-55-3 | 1.00E+00 | 0 | 0 | -1 | -1 |
| 56 | Benzo(a)pyrene | 50-32-8 | 1.00E-01 | -1 | -1 | -1 | -1 |
| 57 | Benzo(b)fluoranthene | 205-99-2 | 1.00E+00 | 0 | 0 | -1 | -1 |
| 58 | Benzo(k)fluoranthene | 207-08-9 | 1.20E+01 | 0 | 0 | 1 | 1 |
| 59 | Chrysene | 218-01-9 | 1.20E+02 | 1 | 1 | 1 | 1 |
| 60 | Dibena(a,h)anthracene | 53-70-3 | 1.00E-01 | -1 | -1 | -1 | -1 |
| 61 | Indeno(1,2,3-c,d)pyrebe | 193-39-5 | 1.00E+00 | 0 | 0 | -1 | -1 |
| 62 | Naphthalene | 91-20-3 | 4.80E+02 | 2 | 2 | 1 | 1 |
| 63 | Acenaphehene | 83-32-9 | 1.40E+03 | 1 | 1 | 1 | 1 |
| 65 | Anthracene | 120-12-7 | 7.20E+03 | 2 | 2 | 1 | 1 |
| 67 | Flouranthene | 206-44-0 | 9.60E+02 | 2 | 1 | 1 | 1 |
| 68 | Flouorene | 86-73-7 | 9.60E+02 | 1 | 1 | 1 | 1 |
| 70 | Pyrene | 129-00-0 | 7.20E+02 | 1 | 1 | 1 | 1 |
| 71 | Aldrin | 309-00-2 | 6.00E-02 | 0 | 0 | -1 | -1 |
| 72 | Chlordane | 57-74-9 | 4.00E+00 | 0 | 0 | 1 | 1 |
| 73 | DDT | 50-29-3 | 4.00E+00 | 1 | 0 | 1 | 1 |
| 74 | Dieldrin | 60-57-1 | 6.00E-02 | 0 | 0 | -1 | -1 |
| 75 | Endosulfan | 115-29-7 | 1.60E+02 | 1 | 1 | 1 | 1 |
| 76 | Endrin | 72-20-8 | 8.00E+00 | 0 | 1 | 1 | 1 |
| 77 | Heptachlor | 76-44-8 | 2.00E-01 | 0 | 0 | -1 | -1 |
| 78 | Lindane | 58-89-9 | 1.00E+00 | 1 | 1 | 1 | 1 |
| 79 | Toxaphene | 8001-35-2 | 1.00E+00 | 0 | 0 | 1 | -1 |
| 80 | 2,4-D | 94-75-7 | 3.10E+02 | 0 | 0 | -1 | 1 |
| 81 | Atrazine | 1912-24-9 | 4.00E+00 | 0 | 0 | 1 | -1 |
| 83 | Carbofuran | 1563-66-2 | 1.30E+02 | 0 | 1 | -1 | 1 |
| 88 | MCPA | 94-74-6 | 1.30E+01 | 0 | 0 | -1 | -1 |
| 89 | Picloram | 1918-02-1 | 1.80E+03 | 0 | 0 | -1 | 1 |
| 90 | Simazine | 122-34-9 | 8.00E+00 | 1 | 0 | 1 | 1 |
| 92 | Total PCB | 1336-36-3 | 4.00E-01 | 0 | 0 | -1 | -1 |
| 94 | Total PCDD/PCDF | 1746-01-6 | 1.00E-05 | -1 | -1 | -1 | -1 |
| 95 | Methyl ethyl ketone | 78-93-3 | 2.00E+04 | 1 | 1 | 1 | 1 |
| 96 | Methyl isobutyl ketone | 108-10-1 | 2.70E+03 | 0 | 1 | -1 | 1 |
| 97 | Dibutyl phthalate | 84-74-2 | 2.60E+03 | 0 | 1 | -1 | 1 |
| 98 | DEHP | 117-81-7 | 7.20E+01 | 0 | 0 | -1 | -1 |
| 99 | Hexachlorobutadine | 87-68-3 | 1.70E+01 | 1 | 1 | 1 | 1 |
| 100 | Methyl tert-butyl ether | 1634-04-4 | 5.40E+02 | 1 | 1 | 1 | 1 |
|  |  | Overall | C1 | C2 | C3 | C4 | C5 |
|  |  | Scores | 77 | 0.70 | 0.75 | 29 | 33 |
|  |  |  |  |  | No. >0 | 53 | 55 |
|  |  |  |  |  | No. <0 | 24 | 22 |

**Table S27c** New Hampshire Code of Administrative Rules

| Number | Pollutant | CAS. No | RGV (mg/kg) | C_2_ | C_3_ | C_4_ | C_5_ |
| --- | --- | --- | --- | --- | --- | --- | --- |
| 1 | Antimony | 7440-36-0 | 9.00E+00 | 0 | 0 | -1 | -1 |
| 2 | Arsenic | 7440-38-2 | 1.10E+01 | 0 | 0 | -1 | -1 |
| 3 | Barium | 7440-39-3 | 1.00E+03 | 0 | 0 | 1 | -1 |
| 4 | Beryllium | 7440-41-7 | 1.20E+01 | 0 | 0 | -1 | -1 |
| 5 | Cadmium | 7440-43-9 | 3.30E+01 | 1 | 1 | 1 | 1 |
| 6 | Chromium (III) | 16065-83-1 | 1.00E+03 | 1 | 0 | 1 | 1 |
| 7 | Chromium (VI) | 18540-29-9 | 1.30E+02 | 0 | 1 | 1 | 1 |
| 10 | Lead | 7439-92-1 | 4.00E+02 | 0 | 1 | 1 | 1 |
| 11 | Manganese | 7439-96-5 | 1.00E+03 | 0 | 0 | -1 | -1 |
| 12 | Mercury | 7439-97-6 | 7.00E+00 | 1 | 1 | 1 | 1 |
| 14 | Nickel | 7440-02-0 | 4.00E+02 | 1 | 1 | 1 | 1 |
| 15 | Selenium | 7782-49-2 | 1.80E+02 | 1 | 1 | 1 | 1 |
| 16 | Silver | 7440-22-4 | 8.90E+01 | 0 | 0 | 1 | 1 |
| 17 | Thallium | 7440-28-0 | 1.00E+01 | 1 | 1 | 1 | 1 |
| 20 | Zinc | 7440-66-6 | 1.00E+03 | 0 | 0 | 1 | -1 |
| 21 | Cyanide | 57-12-5 | 2.20E+01 | 0 | 0 | -1 | -1 |
| 22 | Acrylonitril | 107-13-1 | 5.00E-01 | 0 | 0 | -1 | -1 |
| 25 | Tetrachloromethane | 56-23-5 | 1.20E+01 | 1 | 1 | 1 | 1 |
| 26 | Bromoform | 75-25-2 | 1.00E-01 | -3 | -2 | -1 | -1 |
| 27 | Bromomethane | 74-83-9 | 3.00E-01 | -1 | -1 | -1 | -1 |
| 28 | 1,2-Dichloroethane | 107-06-2 | 1.00E-01 | -1 | -1 | -1 | -1 |
| 29 | 1,1,1-Trichloroethane | 71-55-6 | 7.80E+01 | -1 | 0 | -1 | -1 |
| 30 | 1,1,2-Trichloroethane | 79-00-5 | 1.00E-01 | -1 | -1 | -1 | -1 |
| 31 | Chloroethene | 75-01-4 | 1.00E+00 | 1 | 1 | 1 | 1 |
| 32 | 1,1-Dichloroethene | 75-35-4 | 1.40E+01 | 0 | 0 | 1 | 1 |
| 33 | Trichloroethene | 79-01-6 | 8.00E-01 | -1 | 0 | -1 | -1 |
| 34 | Tetrachloroethene | 127-18-4 | 2.00E+00 | 0 | 0 | -1 | -1 |
| 35 | Benzene | 71-43-2 | 3.00E-01 | 0 | 0 | -1 | -1 |
| 36 | Toluene | 108-88-3 | 1.00E+02 | 0 | 0 | -1 | -1 |
| 38 | 1,2-Dichlorobenzene | 95-50-1 | 8.80E+01 | 0 | 0 | -1 | 1 |
| 39 | 1,3-Dichlorobenzene | 541-73-1 | 1.50E+02 | 1 | 1 | 1 | 1 |
| 40 | 1,4-Dichlorobenzene | 106-46-7 | 7.00E+00 | 0 | 0 | 1 | -1 |
| 41 | 1,2,4Trichlorobenzene | 120-82-1 | 1.90E+01 | 0 | 0 | -1 | -1 |
| 42 | Hexachlorobenzene | 118-74-1 | 8.00E-01 | 0 | 0 | 1 | -1 |
| 44 | Ethylbenzene | 100-41-4 | 1.20E+02 | 1 | 1 | 1 | 1 |
| 45 | Styrene | 100-42-5 | 1.70E+01 | 0 | 0 | -1 | -1 |
| 46 | Xylenes | 1330-20-7 | 5.00E+02 | 1 | 1 | 1 | 1 |
| 47 | Cresol | 1319-77-3 | 7.00E-01 | -2 | -2 | -1 | -1 |
| 48 | Phenol | 108-95-2 | 5.60E+01 | 0 | 0 | -1 | -1 |
| 49 | 2-Chlorophenol | 95-57-8 | 2.00E+00 | -1 | -1 | -1 | -1 |
| 50 | 2,4-Dichlorophenol | 120-83-2 | 7.00E-01 | -1 | -1 | -1 | -1 |
| 51 | 2,4,5-Trichlorophenol | 95-95-4 | 2.40E+01 | -1 | -1 | -1 | -1 |
| 52 | 2,4,6-Trichlorophenol | 88-06-2 | 7.00E-01 | -1 | -1 | -1 | -1 |
| 53 | 2,3,4,6-Tetrahlorophenol | 58-90-2 | 1.30E+02 | 0 | 0 | -1 | 1 |
| 54 | Pentachlorophenol | 87-86-5 | 3.00E+00 | 0 | 0 | -1 | -1 |
| 55 | Benz(a)anthracene | 56-55-3 | 1.00E+00 | 0 | 0 | -1 | -1 |
| 56 | Benzo(a)pyrene | 50-32-8 | 7.00E-01 | 0 | 0 | -1 | 1 |
| 57 | Benzo(b)fluoranthene | 205-99-2 | 1.00E+00 | 0 | 0 | -1 | -1 |
| 58 | Benzo(k)fluoranthene | 207-08-9 | 1.20E+01 | 0 | 0 | 1 | 1 |
| 59 | Chrysene | 218-01-9 | 1.20E+02 | 1 | 1 | 1 | 1 |
| 60 | Dibena(a,h)anthracene | 53-70-3 | 7.00E-01 | 0 | 0 | 1 | -1 |
| 61 | Indeno(1,2,3-c,d)pyrebe | 193-39-5 | 1.00E+00 | 0 | 0 | -1 | -1 |
| 62 | Naphthalene | 91-20-3 | 5.00E+00 | 0 | 0 | -1 | 1 |
| 63 | Acenaphehene | 83-32-9 | 3.40E+02 | 1 | 1 | 1 | 1 |
| 64 | Acenaphthylene | 208-96-8 | 4.90E+02 | 1 | 1 | 1 | 1 |
| 65 | Anthracene | 120-12-7 | 1.00E+03 | 1 | 1 | 1 | 1 |
| 67 | Flouranthene | 206-44-0 | 9.60E+02 | 2 | 1 | 1 | 1 |
| 68 | Flouorene | 86-73-7 | 7.70E+01 | 0 | 0 | -1 | -1 |
| 70 | Pyrene | 129-00-0 | 7.20E+02 | 1 | 1 | 1 | 1 |
| 71 | Aldrin | 309-00-2 | 9.00E-02 | 0 | 0 | -1 | -1 |
| 72 | Chlordane | 57-74-9 | 4.00E+00 | 0 | 0 | 1 | 1 |
| 73 | DDT | 50-29-3 | 4.00E+00 | 1 | 0 | 1 | 1 |
| 74 | Dieldrin | 60-57-1 | 6.00E-02 | 0 | 0 | -1 | -1 |
| 75 | Endosulfan | 115-29-7 | 4.50E+01 | 0 | 1 | 1 | 1 |
| 76 | Endrin | 72-20-8 | 8.00E+00 | 0 | 1 | 1 | 1 |
| 77 | Heptachlor | 76-44-8 | 2.00E-01 | 0 | 0 | -1 | -1 |
| 78 | Lindane | 58-89-9 | 9.00E-02 | -1 | -1 | -1 | -1 |
| 79 | Toxaphene | 8001-35-2 | 1.00E+00 | 0 | 0 | 1 | -1 |
| 80 | 2,4-D | 94-75-7 | 3.00E+02 | 0 | 0 | -1 | 1 |
| 81 | Atrazine | 1912-24-9 | 9.00E-02 | -1 | -2 | -1 | -1 |
| 83 | Carbofuran | 1563-66-2 | 6.00E-01 | -2 | -1 | -1 | -1 |
| 88 | MCPA | 94-74-6 | 1.30E+01 | 0 | 0 | -1 | -1 |
| 89 | Picloram | 1918-02-1 | 6.00E+00 | -3 | -2 | -1 | -1 |
| 90 | Simazine | 122-34-9 | 4.00E-01 | -1 | -1 | -1 | -1 |
| 92 | Total PCB | 1336-36-3 | 1.00E+00 | 0 | 0 | 1 | 1 |
| 94 | Total PCDD/PCDF | 1746-01-6 | 1.00E-03 | 1 | 1 | 1 | 1 |
| 95 | Methyl ethyl ketone | 78-93-3 | 5.10E+01 | -2 | -1 | -1 | -1 |
| 96 | Methyl isobutyl ketone | 108-10-1 | 2.90E+01 | -2 | -1 | -1 | -1 |
| 97 | Dibutyl phthalate | 84-74-2 | 2.60E+03 | -2 | -1 | -1 | 1 |
| 98 | DEHP | 117-81-7 | 7.20E+01 | 0 | 0 | -1 | -1 |
| 99 | Hexachlorobutadine | 87-68-3 | 1.70E+01 | 1 | 1 | 1 | 1 |
| 100 | Methyl tert-butyl ether | 1634-04-4 | 2.00E-01 | -2 | -2 | -1 | -1 |
|  |  | Overall | C1 | C2 | C3 | C4 | C5 |
|  |  | Scores | 82 | -0.11 | -0.012 | -12 | -12 |
|  |  |  |  |  | No. >0 | 35 | 35 |
|  |  |  |  |  | No. <0 | 47 | 47 |

**Table S28a** New Jersey Administrative Code

| Number | Pollutant | CAS. No | RGV (mg/kg) | C_2_ | C_3_ | C_4_ | C_5_ |
| --- | --- | --- | --- | --- | --- | --- | --- |
| 1 | Antimony | 7440-36-0 | 3.10E+01 | 0 | 1 | 1 | 1 |
| 2 | Arsenic | 7440-38-2 | 1.90E+01 | 0 | 0 | -1 | 1 |
| 3 | Barium | 7440-39-3 | 1.60E+04 | 2 | 1 | 1 | 1 |
| 4 | Beryllium | 7440-41-7 | 1.60E+01 | 0 | 0 | -1 | -1 |
| 5 | Cadmium | 7440-43-9 | 7.80E+01 | 1 | 1 | 1 | 1 |
| 8 | Cobalt | 7440-48-4 | 1.60E+03 | 2 | 2 | 1 | 1 |
| 9 | Copper | 7440-50-8 | 3.10E+03 | 1 | 1 | 1 | 1 |
| 10 | Lead | 7439-92-1 | 4.00E+02 | 0 | 1 | 1 | 1 |
| 11 | Manganese | 7439-96-5 | 1.10E+04 | 1 | 1 | 1 | 1 |
| 12 | Mercury | 7439-97-6 | 2.30E+01 | 1 | 1 | 1 | 1 |
| 15 | Selenium | 7782-49-2 | 3.90E+02 | 1 | 1 | 1 | 1 |
| 16 | Silver | 7440-22-4 | 3.90E+02 | 1 | 1 | 1 | 1 |
| 17 | Thallium | 7440-28-0 | 5.00E+00 | 0 | 0 | 1 | 1 |
| 19 | Vanadium | 7440-62-2 | 7.80E+01 | 0 | 0 | -1 | -1 |
| 20 | Zinc | 7440-66-6 | 2.30E+04 | 2 | 2 | 1 | 1 |
| 21 | Cyanide | 57-12-5 | 1.60E+03 | 2 | 2 | 1 | 1 |
| 22 | Acrylonitril | 107-13-1 | 9.00E-01 | 0 | 0 | -1 | -1 |
| 23 | Dichloromethane | 75-09-2 | 3.40E+01 | 1 | 1 | 1 | 1 |
| 24 | Trichloromethane | 67-66-3 | 6.00E-01 | 0 | 0 | -1 | -1 |
| 25 | Tetrachloromethane | 56-23-5 | 6.00E-01 | 0 | 0 | -1 | -1 |
| 26 | Bromoform | 75-25-2 | 8.10E+01 | 0 | 0 | 1 | 1 |
| 27 | Bromomethane | 74-83-9 | 2.50E+01 | 1 | 1 | 1 | 1 |
| 28 | 1,2-Dichloroethane | 107-06-2 | 9.00E-01 | 0 | 0 | -1 | -1 |
| 29 | 1,1,1-Trichloroethane | 71-55-6 | 2.90E+02 | 0 | 0 | -1 | 1 |
| 30 | 1,1,2-Trichloroethane | 79-00-5 | 2.00E+00 | 0 | 0 | -1 | -1 |
| 31 | Chloroethene | 75-01-4 | 7.00E-01 | 1 | 1 | 1 | 1 |
| 32 | 1,1-Dichloroethene | 75-35-4 | 1.10E+01 | 0 | 0 | -1 | 1 |
| 33 | Trichloroethene | 79-01-6 | 7.00E+00 | 0 | 1 | 1 | 1 |
| 34 | Tetrachloroethene | 127-18-4 | 2.00E+00 | 0 | 0 | -1 | -1 |
| 35 | Benzene | 71-43-2 | 2.00E+00 | 1 | 1 | 1 | 1 |
| 36 | Toluene | 108-88-3 | 6.30E+03 | 2 | 2 | 1 | 1 |
| 37 | Chlorobenzene | 108-90-7 | 5.10E+02 | 1 | 2 | 1 | 1 |
| 38 | 1,2-Dichlorobenzene | 95-50-1 | 5.30E+03 | 2 | 2 | 1 | 1 |
| 39 | 1,3-Dichlorobenzene | 541-73-1 | 5.30E+03 | 2 | 3 | 1 | 1 |
| 40 | 1,4-Dichlorobenzene | 106-46-7 | 5.00E+00 | 0 | 0 | -1 | -1 |
| 41 | 1,2,4Trichlorobenzene | 120-82-1 | 7.30E+01 | 1 | 1 | 1 | 1 |
| 42 | Hexachlorobenzene | 118-74-1 | 3.00E-01 | 0 | 0 | -1 | -1 |
| 43 | Nitrobenzene | 98-95-3 | 3.10E+01 | 0 | 1 | 1 | 1 |
| 44 | Ethylbenzene | 100-41-4 | 7.80E+03 | 3 | 3 | 1 | 1 |
| 45 | Styrene | 100-42-5 | 9.00E+01 | 0 | 0 | -1 | 1 |
| 46 | Xylenes | 1330-20-7 | 1.20E+04 | 2 | 2 | 1 | 1 |
| 47 | Cresol | 1319-77-3 | 3.10E+01 | -1 | 0 | -1 | -1 |
| 48 | Phenol | 108-95-2 | 1.80E+04 | 2 | 2 | 1 | 1 |
| 49 | 2-Chlorophenol | 95-57-8 | 3.10E+02 | 1 | 1 | 1 | 1 |
| 50 | 2,4-Dichlorophenol | 120-83-2 | 1.80E+02 | 1 | 1 | 1 | 1 |
| 51 | 2,4,5-Trichlorophenol | 95-95-4 | 6.10E+03 | 1 | 2 | 1 | 1 |
| 52 | 2,4,6-Trichlorophenol | 88-06-2 | 1.90E+01 | 0 | 0 | 1 | 1 |
| 54 | Pentachlorophenol | 87-86-5 | 3.00E+00 | 0 | 0 | -1 | -1 |
| 55 | Benz(a)anthracene | 56-55-3 | 6.00E-01 | 0 | 0 | -1 | -1 |
| 56 | Benzo(a)pyrene | 50-32-8 | 2.00E-01 | 0 | 0 | -1 | -1 |
| 57 | Benzo(b)fluoranthene | 205-99-2 | 6.00E-01 | 0 | 0 | -1 | -1 |
| 58 | Benzo(k)fluoranthene | 207-08-9 | 6.00E+00 | 0 | 0 | -1 | -1 |
| 59 | Chrysene | 218-01-9 | 6.20E+01 | 1 | 1 | 1 | 1 |
| 60 | Dibena(a,h)anthracene | 53-70-3 | 2.00E-01 | 0 | 0 | -1 | -1 |
| 61 | Indeno(1,2,3-c,d)pyrebe | 193-39-5 | 6.00E-01 | 0 | -1 | -1 | -1 |
| 62 | Naphthalene | 91-20-3 | 6.00E+00 | 0 | 1 | -1 | 1 |
| 63 | Acenaphehene | 83-32-9 | 3.40E+03 | 2 | 2 | 1 | 1 |
| 65 | Anthracene | 120-12-7 | 1.70E+04 | 2 | 2 | 1 | 1 |
| 66 | Benzo(g,h,i) perylene | 191-24-2 | 3.80E+05 | 4 | 4 | 1 | 1 |
| 67 | Flouranthene | 206-44-0 | 2.30E+03 | 3 | 2 | 1 | 1 |
| 68 | Flouorene | 86-73-7 | 2.30E+03 | 1 | 1 | 1 | 1 |
| 70 | Pyrene | 129-00-0 | 1.70E+03 | 1 | 1 | 1 | 1 |
| 71 | Aldrin | 309-00-2 | 4.00E-02 | 0 | -1 | -1 | -1 |
| 72 | Chlordane | 57-74-9 | 2.00E-01 | -1 | -1 | -1 | -1 |
| 73 | DDT | 50-29-3 | 2.00E+00 | 0 | 0 | -1 | -1 |
| 74 | Dieldrin | 60-57-1 | 4.00E-02 | 0 | -1 | -1 | -1 |
| 75 | Endosulfan | 115-29-7 | 4.70E+02 | 1 | 2 | 1 | 1 |
| 76 | Endrin | 72-20-8 | 2.30E+01 | 1 | 1 | 1 | 1 |
| 77 | Heptachlor | 76-44-8 | 1.00E-01 | 0 | 0 | -1 | -1 |
| 78 | Lindane | 58-89-9 | 4.00E-01 | 0 | 0 | -1 | -1 |
| 79 | Toxaphene | 8001-35-2 | 6.00E-01 | 0 | 0 | -1 | -1 |
| 81 | Atrazine | 1912-24-9 | 2.10E+02 | 2 | 2 | 1 | 1 |
| 95 | Methyl ethyl ketone | 78-93-3 | 3.10E+03 | 0 | 1 | -1 | 1 |
| 97 | Dibutyl phthalate | 84-74-2 | 6.10E+03 | 0 | 0 | -1 | 1 |
| 98 | DEHP | 117-81-7 | 3.50E+01 | 0 | 0 | -1 | -1 |
| 99 | Hexachlorobutadine | 87-68-3 | 6.00E+00 | 0 | 0 | -1 | 1 |
| 100 | Methyl tert-butyl ether | 1634-04-4 | 1.10E+02 | 0 | 1 | 1 | 1 |
|  |  | Overall | C1 | C2 | C3 | C4 | C5 |
|  |  | Scores | 77 | 0.68 | 0.77 | 9 | 25 |
|  |  |  |  |  | No. >0 | 43 | 51 |
|  |  |  |  |  | No. <0 | 34 | 26 |

**Table S28b** New Jersey Dept. of Environmental Protection

| Number | Pollutant | CAS. No | RGV (mg/kg) | C_2_ | C_3_ | C_4_ | C_5_ |
| --- | --- | --- | --- | --- | --- | --- | --- |
| 1 | Antimony | 7440-36-0 | 1.40E+01 | 0 | 0 | -1 | -1 |
| 2 | Arsenic | 7440-38-2 | 2.00E+01 | 0 | 0 | -1 | 1 |
| 3 | Barium | 7440-39-3 | 7.00E+02 | 0 | 0 | -1 | -1 |
| 4 | Beryllium | 7440-41-7 | 1.00E+00 | -1 | -1 | -1 | -1 |
| 5 | Cadmium | 7440-43-9 | 3.90E+01 | 1 | 1 | 1 | 1 |
| 6 | Chromium (III) | 16065-83-1 | 1.20E+05 | 3 | 2 | 1 | 1 |
| 7 | Chromium (VI) | 18540-29-9 | 2.40E+02 | 1 | 1 | 1 | 1 |
| 9 | Copper | 7440-50-8 | 6.00E+02 | 1 | 1 | 1 | 1 |
| 10 | Lead | 7439-92-1 | 4.00E+02 | 0 | 1 | 1 | 1 |
| 12 | Mercury | 7439-97-6 | 1.40E+01 | 1 | 1 | 1 | 1 |
| 14 | Nickel | 7440-02-0 | 2.50E+02 | 1 | 0 | 1 | 1 |
| 15 | Selenium | 7782-49-2 | 6.30E+01 | 0 | 1 | 1 | 1 |
| 16 | Silver | 7440-22-4 | 1.10E+02 | 1 | 1 | 1 | 1 |
| 17 | Thallium | 7440-28-0 | 2.00E+00 | 0 | 0 | -1 | -1 |
| 19 | Vanadium | 7440-62-2 | 3.70E+02 | 1 | 1 | 1 | 1 |
| 20 | Zinc | 7440-66-6 | 1.50E+03 | 1 | 0 | 1 | 1 |
| 21 | Cyanide | 57-12-5 | 1.10E+03 | 2 | 2 | 1 | 1 |
| 22 | Acrylonitril | 107-13-1 | 1.00E+00 | 0 | 0 | -1 | -1 |
| 23 | Dichloromethane | 75-09-2 | 4.90E+01 | 1 | 1 | 1 | 1 |
| 24 | Trichloromethane | 67-66-3 | 1.90E+01 | 1 | 1 | 1 | 1 |
| 25 | Tetrachloromethane | 56-23-5 | 2.00E+00 | 1 | 1 | 1 | 1 |
| 26 | Bromoform | 75-25-2 | 8.60E+01 | 0 | 0 | 1 | 1 |
| 27 | Bromomethane | 74-83-9 | 7.90E+01 | 1 | 1 | 1 | 1 |
| 28 | 1,2-Dichloroethane | 107-06-2 | 6.00E+00 | 1 | 1 | 1 | 1 |
| 29 | 1,1,1-Trichloroethane | 71-55-6 | 2.10E+02 | 0 | 0 | -1 | -1 |
| 30 | 1,1,2-Trichloroethane | 79-00-5 | 2.20E+01 | 1 | 1 | 1 | 1 |
| 31 | Chloroethene | 75-01-4 | 2.00E+00 | 1 | 1 | 1 | 1 |
| 32 | 1,1-Dichloroethene | 75-35-4 | 8.00E+00 | 0 | 0 | -1 | -1 |
| 33 | Trichloroethene | 79-01-6 | 2.30E+01 | 1 | 1 | 1 | 1 |
| 34 | Tetrachloroethene | 127-18-4 | 4.00E+00 | 0 | 0 | 1 | 1 |
| 35 | Benzene | 71-43-2 | 3.00E+00 | 1 | 1 | 1 | 1 |
| 36 | Toluene | 108-88-3 | 1.00E+03 | 1 | 1 | 1 | 1 |
| 37 | Chlorobenzene | 108-90-7 | 3.70E+01 | 0 | 0 | -1 | 1 |
| 38 | 1,2-Dichlorobenzene | 95-50-1 | 5.10E+03 | 2 | 2 | 1 | 1 |
| 39 | 1,3-Dichlorobenzene | 541-73-1 | 5.10E+03 | 2 | 3 | 1 | 1 |
| 40 | 1,4-Dichlorobenzene | 106-46-7 | 5.70E+02 | 2 | 2 | 1 | 1 |
| 41 | 1,2,4Trichlorobenzene | 120-82-1 | 6.80E+01 | 1 | 1 | 1 | 1 |
| 42 | Hexachlorobenzene | 118-74-1 | 6.60E-01 | 0 | 0 | -1 | -1 |
| 43 | Nitrobenzene | 98-95-3 | 2.80E+01 | 0 | 1 | 1 | 1 |
| 44 | Ethylbenzene | 100-41-4 | 1.00E+03 | 2 | 2 | 1 | 1 |
| 45 | Styrene | 100-42-5 | 2.30E+01 | 0 | 0 | -1 | -1 |
| 46 | Xylenes | 1330-20-7 | 4.10E+02 | 1 | 1 | 1 | 1 |
| 47 | Cresol | 1319-77-3 | 2.80E+03 | 1 | 2 | 1 | 1 |
| 48 | Phenol | 108-95-2 | 1.00E+04 | 2 | 2 | 1 | 1 |
| 49 | 2-Chlorophenol | 95-57-8 | 2.80E+02 | 1 | 1 | 1 | 1 |
| 50 | 2,4-Dichlorophenol | 120-83-2 | 1.70E+02 | 1 | 1 | 1 | 1 |
| 51 | 2,4,5-Trichlorophenol | 95-95-4 | 5.60E+03 | 1 | 2 | 1 | 1 |
| 52 | 2,4,6-Trichlorophenol | 88-06-2 | 6.20E+01 | 1 | 1 | 1 | 1 |
| 54 | Pentachlorophenol | 87-86-5 | 6.00E+00 | 0 | 0 | 1 | 1 |
| 55 | Benz(a)anthracene | 56-55-3 | 9.00E-01 | 0 | 0 | -1 | -1 |
| 56 | Benzo(a)pyrene | 50-32-8 | 6.60E-01 | 0 | 0 | -1 | -1 |
| 57 | Benzo(b)fluoranthene | 205-99-2 | 9.00E-01 | 0 | 0 | -1 | -1 |
| 58 | Benzo(k)fluoranthene | 207-08-9 | 9.00E-01 | -1 | -1 | -1 | -1 |
| 59 | Chrysene | 218-01-9 | 9.00E+00 | 0 | 0 | -1 | -1 |
| 60 | Dibena(a,h)anthracene | 53-70-3 | 6.60E-01 | 0 | 0 | 1 | -1 |
| 61 | Indeno(1,2,3-c,d)pyrebe | 193-39-5 | 9.00E-01 | 0 | 0 | -1 | -1 |
| 62 | Naphthalene | 91-20-3 | 2.30E+02 | 1 | 2 | 1 | 1 |
| 63 | Acenaphehene | 83-32-9 | 3.40E+03 | 2 | 2 | 1 | 1 |
| 65 | Anthracene | 120-12-7 | 1.00E+04 | 2 | 2 | 1 | 1 |
| 67 | Flouranthene | 206-44-0 | 2.30E+03 | 3 | 2 | 1 | 1 |
| 68 | Flouorene | 86-73-7 | 2.30E+03 | 1 | 1 | 1 | 1 |
| 70 | Pyrene | 129-00-0 | 1.70E+03 | 1 | 1 | 1 | 1 |
| 71 | Aldrin | 309-00-2 | 4.00E-02 | 0 | -1 | -1 | -1 |
| 73 | DDT | 50-29-3 | 2.00E+00 | 0 | 0 | -1 | -1 |
| 74 | Dieldrin | 60-57-1 | 4.20E-02 | 0 | -1 | -1 | -1 |
| 75 | Endosulfan | 115-29-7 | 3.40E+02 | 1 | 2 | 1 | 1 |
| 76 | Endrin | 72-20-8 | 1.70E+01 | 1 | 1 | 1 | 1 |
| 77 | Heptachlor | 76-44-8 | 1.50E-01 | 0 | 0 | -1 | -1 |
| 78 | Lindane | 58-89-9 | 5.20E-01 | 0 | 0 | -1 | 1 |
| 79 | Toxaphene | 8001-35-2 | 1.00E-01 | -1 | -1 | -1 | -1 |
| 92 | Total PCB | 1336-36-3 | 4.90E-01 | 0 | 0 | -1 | -1 |
| 95 | Methyl ethyl ketone | 78-93-3 | 1.00E+03 | -1 | 0 | -1 | -1 |
| 96 | Methyl isobutyl ketone | 108-10-1 | 1.00E+03 | 0 | 0 | -1 | 1 |
| 97 | Dibutyl phthalate | 84-74-2 | 5.70E+03 | -1 | 0 | -1 | 1 |
| 98 | DEHP | 117-81-7 | 4.90E+01 | 0 | 0 | -1 | -1 |
| 99 | Hexachlorobutadine | 87-68-3 | 1.00E+00 | -1 | 0 | -1 | -1 |
|  |  | Overall | C1 | C2 | C3 | C4 | C5 |
|  |  | Scores | 76 | 0.61 | 0.67 | 18 | 26 |
|  |  |  |  |  | No. >0 | 47 | 51 |
|  |  |  |  |  | No. <0 | 29 | 25 |

**Table S29** New Mexico Environment Department

| Number | Pollutant | CAS. No | RGV (mg/kg) | C_2_ | C_3_ | C_4_ | C_5_ |
| --- | --- | --- | --- | --- | --- | --- | --- |
| 1 | Antimony | 7440-36-0 | 3.13E+01 | 0 | 1 | 1 | 1 |
| 2 | Arsenic | 7440-38-2 | 4.25E+00 | 0 | 0 | -1 | -1 |
| 3 | Barium | 7440-39-3 | 1.56E+04 | 1 | 1 | 1 | 1 |
| 4 | Beryllium | 7440-41-7 | 1.56E+02 | 1 | 1 | 1 | 1 |
| 5 | Cadmium | 7440-43-9 | 7.05E+01 | 1 | 1 | 1 | 1 |
| 6 | Chromium (III) | 16065-83-1 | 1.17E+05 | 3 | 2 | 1 | 1 |
| 7 | Chromium (VI) | 18540-29-9 | 3.05E+00 | -1 | -1 | -1 | -1 |
| 9 | Copper | 7440-50-8 | 3.13E+03 | 1 | 1 | 1 | 1 |
| 10 | Lead | 7439-92-1 | 4.00E+02 | 0 | 1 | 1 | 1 |
| 11 | Manganese | 7439-96-5 | 1.05E+04 | 1 | 1 | 1 | 1 |
| 12 | Mercury | 7439-97-6 | 2.38E+01 | 1 | 1 | 1 | 1 |
| 13 | Molybdenum | 7439-98-7 | 3.91E+02 | 1 | 1 | 1 | 1 |
| 14 | Nickel | 7440-02-0 | 1.56E+03 | 1 | 1 | 1 | 1 |
| 15 | Selenium | 7782-49-2 | 3.91E+02 | 1 | 1 | 1 | 1 |
| 16 | Silver | 7440-22-4 | 3.91E+02 | 1 | 1 | 1 | 1 |
| 17 | Thallium | 7440-28-0 | 7.82E-01 | 0 | 0 | -1 | -1 |
| 19 | Vanadium | 7440-62-2 | 3.94E+02 | 1 | 1 | 1 | 1 |
| 20 | Zinc | 7440-66-6 | 2.35E+04 | 2 | 2 | 1 | 1 |
| 21 | Cyanide | 57-12-5 | 1.12E+01 | 0 | 0 | -1 | -1 |
| 22 | Acrylonitril | 107-13-1 | 4.93E+00 | 1 | 1 | 1 | 1 |
| 23 | Dichloromethane | 75-09-2 | 4.09E+02 | 2 | 2 | 1 | 1 |
| 24 | Trichloromethane | 67-66-3 | 5.90E+00 | 1 | 1 | 1 | 1 |
| 25 | Tetrachloromethane | 56-23-5 | 1.07E+01 | 1 | 1 | 1 | 1 |
| 26 | Bromoform | 75-25-2 | 6.74E+02 | 1 | 1 | 1 | 1 |
| 27 | Bromomethane | 74-83-9 | 1.77E+01 | 1 | 1 | 1 | 1 |
| 28 | 1,2-Dichloroethane | 107-06-2 | 8.32E+00 | 1 | 1 | 1 | 1 |
| 29 | 1,1,1-Trichloroethane | 71-55-6 | 1.44E+04 | 2 | 2 | 1 | 1 |
| 30 | 1,1,2-Trichloroethane | 79-00-5 | 2.61E+00 | 0 | 0 | -1 | 1 |
| 31 | Chloroethene | 75-01-4 | 7.42E-01 | 1 | 1 | 1 | 1 |
| 32 | 1,1-Dichloroethene | 75-35-4 | 4.40E+02 | 2 | 2 | 1 | 1 |
| 33 | Trichloroethene | 79-01-6 | 6.77E+00 | 0 | 1 | 1 | 1 |
| 34 | Tetrachloroethene | 127-18-4 | 1.11E+02 | 2 | 2 | 1 | 1 |
| 35 | Benzene | 71-43-2 | 1.78E+01 | 2 | 1 | 1 | 1 |
| 36 | Toluene | 108-88-3 | 5.23E+03 | 2 | 2 | 1 | 1 |
| 37 | Chlorobenzene | 108-90-7 | 3.78E+02 | 1 | 1 | 1 | 1 |
| 38 | 1,2-Dichlorobenzene | 95-50-1 | 2.15E+03 | 1 | 2 | 1 | 1 |
| 40 | 1,4-Dichlorobenzene | 106-46-7 | 3.28E+01 | 1 | 1 | 1 | 1 |
| 41 | 1,2,4Trichlorobenzene | 120-82-1 | 8.29E+01 | 1 | 1 | 1 | 1 |
| 42 | Hexachlorobenzene | 118-74-1 | 3.33E+00 | 1 | 1 | 1 | 1 |
| 43 | Nitrobenzene | 98-95-3 | 6.04E+01 | 1 | 1 | 1 | 1 |
| 44 | Ethylbenzene | 100-41-4 | 7.51E+01 | 1 | 1 | 1 | 1 |
| 45 | Styrene | 100-42-5 | 7.26E+03 | 2 | 2 | 1 | 1 |
| 46 | Xylenes | 1330-20-7 | 8.71E+02 | 1 | 1 | 1 | 1 |
| 48 | Phenol | 108-95-2 | 1.85E+04 | 2 | 2 | 1 | 1 |
| 49 | 2-Chlorophenol | 95-57-8 | 3.91E+02 | 1 | 1 | 1 | 1 |
| 50 | 2,4-Dichlorophenol | 120-83-2 | 1.85E+02 | 1 | 1 | 1 | 1 |
| 51 | 2,4,5-Trichlorophenol | 95-95-4 | 6.16E+03 | 1 | 2 | 1 | 1 |
| 52 | 2,4,6-Trichlorophenol | 88-06-2 | 6.16E+01 | 1 | 1 | 1 | 1 |
| 54 | Pentachlorophenol | 87-86-5 | 9.85E+00 | 1 | 1 | 1 | 1 |
| 55 | Benz(a)anthracene | 56-55-3 | 1.53E+00 | 0 | 0 | -1 | -1 |
| 56 | Benzo(a)pyrene | 50-32-8 | 1.53E-01 | 0 | 0 | -1 | -1 |
| 57 | Benzo(b)fluoranthene | 205-99-2 | 1.53E+00 | 0 | 0 | -1 | -1 |
| 58 | Benzo(k)fluoranthene | 207-08-9 | 1.53E+01 | 0 | 1 | 1 | 1 |
| 59 | Chrysene | 218-01-9 | 1.53E+02 | 1 | 1 | 1 | 1 |
| 60 | Dibena(a,h)anthracene | 53-70-3 | 1.53E-01 | 0 | 0 | -1 | -1 |
| 61 | Indeno(1,2,3-c,d)pyrebe | 193-39-5 | 1.53E+00 | 0 | 0 | -1 | -1 |
| 62 | Naphthalene | 91-20-3 | 4.97E+01 | 1 | 1 | 1 | 1 |
| 63 | Acenaphehene | 83-32-9 | 3.48E+03 | 2 | 2 | 1 | 1 |
| 65 | Anthracene | 120-12-7 | 1.74E+04 | 2 | 2 | 1 | 1 |
| 67 | Flouranthene | 206-44-0 | 2.32E+03 | 3 | 2 | 1 | 1 |
| 68 | Flouorene | 86-73-7 | 2.32E+03 | 1 | 1 | 1 | 1 |
| 70 | Pyrene | 129-00-0 | 1.74E+03 | 1 | 1 | 1 | 1 |
| 71 | Aldrin | 309-00-2 | 3.11E-01 | 1 | 0 | 1 | 1 |
| 72 | Chlordane | 57-74-9 | 1.77E+01 | 1 | 1 | 1 | 1 |
| 73 | DDT | 50-29-3 | 1.87E+01 | 1 | 1 | 1 | 1 |
| 74 | Dieldrin | 60-57-1 | 3.33E-01 | 1 | 0 | 1 | 1 |
| 75 | Endosulfan | 115-29-7 | 3.70E+02 | 1 | 2 | 1 | 1 |
| 76 | Endrin | 72-20-8 | 1.85E+01 | 1 | 1 | 1 | 1 |
| 77 | Heptachlor | 76-44-8 | 1.18E+00 | 1 | 1 | 1 | 1 |
| 78 | Lindane | 58-89-9 | 5.63E+00 | 1 | 1 | 1 | 1 |
| 79 | Toxaphene | 8001-35-2 | 4.84E+00 | 1 | 1 | 1 | 1 |
| 93 | PCB 118 | 31508-00-6 | 1.25E+00 | 1 | 1 | 1 | 1 |
| 94 | Total PCDD/PCDF | 1746-01-6 | 4.90E-05 | 0 | 0 | -1 | -1 |
| 95 | Methyl ethyl ketone | 78-93-3 | 3.74E+04 | 1 | 2 | 1 | 1 |
| 96 | Methyl isobutyl ketone | 108-10-1 | 5.81E+03 | 0 | 1 | 1 | 1 |
| 97 | Dibutyl phthalate | 84-74-2 | 6.16E+03 | 0 | 1 | 1 | 1 |
| 98 | DEHP | 117-81-7 | 3.80E+02 | 1 | 1 | 1 | 1 |
| 100 | Methyl tert-butyl ether | 1634-04-4 | 9.75E+02 | 1 | 2 | 1 | 1 |
|  |  | Overall | C1 | C2 | C3 | C4 | C5 |
|  |  | Scores | 78 | 0.96 | 1.04 | 56 | 58 |
|  |  |  |  |  | No. >0 | 67 | 68 |
|  |  |  |  |  | No. <0 | 11 | 10 |

**Table S30a** New York Dept. of Environmental Conservation - Supplemental

| Number | Pollutant | CAS. No | RGV (mg/kg) | C_2_ | C_3_ | C_4_ | C_5_ |
| --- | --- | --- | --- | --- | --- | --- | --- |
| 8 | Cobalt | 7440-48-4 | 3.00E+01 | 0 | 0 | -1 | -1 |
| 19 | Vanadium | 7440-62-2 | 1.00E+02 | 0 | 0 | -1 | -1 |
| 35 | Benzene | 71-43-2 | 6.00E-02 | -1 | -1 | -1 | -1 |
| 36 | Toluene | 108-88-3 | 7.00E-01 | -2 | -2 | -1 | -1 |
| 42 | Hexachlorobenzene | 118-74-1 | 4.10E-01 | 0 | 0 | -1 | -1 |
| 43 | Nitrobenzene | 98-95-3 | 3.70E+00 | 0 | 0 | -1 | -1 |
| 44 | Ethylbenzene | 100-41-4 | 1.00E+00 | -1 | -1 | -1 | -1 |
| 46 | Xylenes | 1330-20-7 | 2.60E-01 | -3 | -2 | -1 | -1 |
| 49 | 2-Chlorophenol | 95-57-8 | 1.00E+02 | 1 | 1 | 1 | 1 |
| 50 | 2,4-Dichlorophenol | 120-83-2 | 1.00E+02 | 1 | 1 | 1 | 1 |
| 51 | 2,4,5-Trichlorophenol | 95-95-4 | 1.00E+02 | -1 | 0 | -1 | -1 |
| 55 | Benz(a)anthracene | 56-55-3 | 1.00E+00 | 0 | 0 | -1 | -1 |
| 56 | Benzo(a)pyrene | 50-32-8 | 1.00E+00 | 0 | 0 | 1 | 1 |
| 57 | Benzo(b)fluoranthene | 205-99-2 | 1.00E+00 | 0 | 0 | -1 | -1 |
| 58 | Benzo(k)fluoranthene | 207-08-9 | 8.00E-01 | -1 | -1 | -1 | -1 |
| 59 | Chrysene | 218-01-9 | 1.00E+00 | -1 | -1 | -1 | -1 |
| 61 | Indeno(1,2,3-c,d)pyrebe | 193-39-5 | 5.00E-01 | -1 | -1 | -1 | -1 |
| 62 | Naphthalene | 91-20-3 | 1.20E+01 | 0 | 1 | -1 | 1 |
| 63 | Acenaphehene | 83-32-9 | 2.00E+01 | 0 | -1 | -1 | -1 |
| 64 | Acenaphthylene | 208-96-8 | 1.00E+02 | 0 | 0 | 1 | 1 |
| 65 | Anthracene | 120-12-7 | 1.00E+02 | 0 | 0 | -1 | -1 |
| 66 | Benzo(g,h,i) perylene | 191-24-2 | 1.00E+02 | 1 | 0 | 1 | 1 |
| 67 | Flouranthene | 206-44-0 | 1.00E+02 | 1 | 0 | 1 | -1 |
| 68 | Flouorene | 86-73-7 | 3.00E+01 | 0 | 0 | -1 | -1 |
| 69 | Phenanthrene | 85-01-8 | 1.00E+02 | 1 | 0 | 1 | 1 |
| 70 | Pyrene | 129-00-0 | 1.00E+02 | 0 | 0 | -1 | -1 |
| 80 | 2,4-D | 94-75-7 | 1.00E+02 | -1 | 0 | -1 | -1 |
| 95 | Methyl ethyl ketone | 78-93-3 | 1.00E+02 | -2 | -1 | -1 | -1 |
| 97 | Dibutyl phthalate | 84-74-2 | 1.00E+02 | 0 | 0 | -1 | -1 |
| 98 | DEHP | 117-81-7 | 5.00E+01 | 0 | 0 | -1 | -1 |
| 100 | Methyl tert-butyl ether | 1634-04-4 | 9.30E-01 | -2 | -1 | -1 | -1 |
|  |  | Overall | C1 | C2 | C3 | C4 | C5 |
|  |  | Scores | 31 | -0.35 | -0.29 | -17 | -17 |
|  |  |  |  |  | No. >0 | 7 | 7 |
|  |  |  |  |  | No. <0 | 24 | 24 |

**Table S30b** New York (NyDEC, 2006) - Table 375-6.8(a) Unrestricted Use

| Number | Pollutant | CAS. No | RGV (mg/kg) | C_2_ | C_3_ | C_4_ | C_5_ |
| --- | --- | --- | --- | --- | --- | --- | --- |
| 2 | Arsenic | 7440-38-2 | 1.30E+01 | 0 | 0 | -1 | 1 |
| 3 | Barium | 7440-39-3 | 3.50E+02 | 0 | 0 | -1 | -1 |
| 4 | Beryllium | 7440-41-7 | 7.20E+00 | 0 | 0 | -1 | -1 |
| 5 | Cadmium | 7440-43-9 | 2.50E+00 | 0 | 0 | -1 | -1 |
| 6 | Chromium (III) | 16065-83-1 | 3.00E+01 | -1 | -1 | -1 | -1 |
| 7 | Chromium (VI) | 18540-29-9 | 1.00E+00 | -2 | -1 | -1 | -1 |
| 9 | Copper | 7440-50-8 | 5.00E+01 | 0 | -1 | -1 | -1 |
| 10 | Lead | 7439-92-1 | 6.30E+01 | 0 | 0 | -1 | -1 |
| 11 | Manganese | 7439-96-5 | 1.60E+03 | 0 | 0 | 1 | -1 |
| 12 | Mercury | 7439-97-6 | 1.80E-01 | -1 | -1 | -1 | -1 |
| 14 | Nickel | 7440-02-0 | 3.00E+01 | 0 | 0 | -1 | -1 |
| 15 | Selenium | 7782-49-2 | 3.90E+00 | -1 | -1 | -1 | -1 |
| 16 | Silver | 7440-22-4 | 2.00E+00 | -1 | -1 | -1 | -1 |
| 20 | Zinc | 7440-66-6 | 1.09E+02 | -1 | -1 | -1 | -1 |
| 21 | Cyanide | 57-12-5 | 2.70E+01 | 0 | 0 | -1 | -1 |
| 23 | Dichloromethane | 75-09-2 | 5.00E-02 | -2 | -2 | -1 | -1 |
| 24 | Trichloromethane | 67-66-3 | 3.70E-01 | 0 | -1 | -1 | -1 |
| 25 | Tetrachloromethane | 56-23-5 | 7.60E-01 | 0 | 0 | -1 | -1 |
| 28 | 1,2-Dichloroethane | 107-06-2 | 2.00E-02 | -2 | -2 | -1 | -1 |
| 29 | 1,1,1-Trichloroethane | 71-55-6 | 6.80E-01 | -3 | -2 | -1 | -1 |
| 31 | Chloroethene | 75-01-4 | 2.00E-02 | -1 | -1 | -1 | -1 |
| 32 | 1,1-Dichloroethene | 75-35-4 | 3.30E-01 | -1 | -1 | -1 | -1 |
| 33 | Trichloroethene | 79-01-6 | 4.70E-01 | -1 | -1 | -1 | -1 |
| 34 | Tetrachloroethene | 127-18-4 | 1.30E+00 | 0 | 0 | -1 | -1 |
| 35 | Benzene | 71-43-2 | 6.00E-02 | -1 | -1 | -1 | -1 |
| 36 | Toluene | 108-88-3 | 7.00E-01 | -2 | -2 | -1 | -1 |
| 37 | Chlorobenzene | 108-90-7 | 1.10E+00 | -2 | -1 | -1 | -1 |
| 38 | 1,2-Dichlorobenzene | 95-50-1 | 1.10E+00 | -2 | -2 | -1 | -1 |
| 39 | 1,3-Dichlorobenzene | 541-73-1 | 2.40E+00 | -1 | -1 | -1 | -1 |
| 40 | 1,4-Dichlorobenzene | 106-46-7 | 1.80E+00 | 0 | 0 | -1 | -1 |
| 41 | 1,2,4Trichlorobenzene | 120-82-1 | 3.60E+00 | -1 | -1 | -1 | -1 |
| 42 | Hexachlorobenzene | 118-74-1 | 3.30E-01 | 0 | 0 | -1 | -1 |
| 44 | Ethylbenzene | 100-41-4 | 1.00E+00 | -1 | -1 | -1 | -1 |
| 46 | Xylenes | 1330-20-7 | 2.60E-01 | -3 | -2 | -1 | -1 |
| 47 | Cresol | 1319-77-3 | 3.30E-01 | -2 | -2 | -1 | -1 |
| 48 | Phenol | 108-95-2 | 3.30E-01 | -2 | -3 | -1 | -1 |
| 54 | Pentachlorophenol | 87-86-5 | 8.00E-01 | -1 | 0 | -1 | -1 |
| 55 | Benz(a)anthracene | 56-55-3 | 1.00E+00 | 0 | 0 | -1 | -1 |
| 56 | Benzo(a)pyrene | 50-32-8 | 1.00E+00 | 0 | 0 | 1 | 1 |
| 57 | Benzo(b)fluoranthene | 205-99-2 | 1.00E+00 | 0 | 0 | -1 | -1 |
| 58 | Benzo(k)fluoranthene | 207-08-9 | 8.00E-01 | -1 | -1 | -1 | -1 |
| 59 | Chrysene | 218-01-9 | 1.00E+00 | -1 | -1 | -1 | -1 |
| 60 | Dibena(a,h)anthracene | 53-70-3 | 3.30E-01 | 0 | 0 | -1 | -1 |
| 61 | Indeno(1,2,3-c,d)pyrebe | 193-39-5 | 5.00E-01 | -1 | -1 | -1 | -1 |
| 62 | Naphthalene | 91-20-3 | 1.20E+01 | 0 | 1 | -1 | 1 |
| 63 | Acenaphehene | 83-32-9 | 2.00E+01 | 0 | -1 | -1 | -1 |
| 64 | Acenaphthylene | 208-96-8 | 1.00E+02 | 0 | 0 | 1 | 1 |
| 65 | Anthracene | 120-12-7 | 1.00E+02 | 0 | 0 | -1 | -1 |
| 66 | Benzo(g,h,i) perylene | 191-24-2 | 1.00E+02 | 1 | 0 | 1 | 1 |
| 67 | Flouranthene | 206-44-0 | 1.00E+02 | 1 | 0 | 1 | -1 |
| 68 | Flouorene | 86-73-7 | 3.00E+01 | 0 | 0 | -1 | -1 |
| 69 | Phenanthrene | 85-01-8 | 1.00E+02 | 1 | 0 | 1 | 1 |
| 70 | Pyrene | 129-00-0 | 1.00E+02 | 0 | 0 | -1 | -1 |
| 71 | Aldrin | 309-00-2 | 5.00E-03 | -1 | -1 | -1 | -1 |
| 72 | Chlordane | 57-74-9 | 9.40E-02 | -1 | -1 | -1 | -1 |
| 73 | DDT | 50-29-3 | 3.30E-03 | -3 | -3 | -1 | -1 |
| 74 | Dieldrin | 60-57-1 | 5.00E-03 | -1 | -2 | -1 | -1 |
| 75 | Endosulfan | 115-29-7 | 2.40E+00 | -1 | -1 | -1 | -1 |
| 76 | Endrin | 72-20-8 | 1.40E-02 | -2 | -2 | -1 | -1 |
| 77 | Heptachlor | 76-44-8 | 4.20E-02 | 0 | -1 | -1 | -1 |
| 78 | Lindane | 58-89-9 | 1.00E-01 | 0 | 0 | -1 | -1 |
| 92 | Total PCB | 1336-36-3 | 1.00E-01 | -1 | -1 | -1 | -1 |
| 95 | Methyl ethyl ketone | 78-93-3 | 1.20E-01 | -5 | -4 | -1 | -1 |
| 100 | Methyl tert-butyl ether | 1634-04-4 | 9.30E-01 | -2 | -1 | -1 | -1 |
|  |  | Overall | C1 | C2 | C3 | C4 | C5 |
|  |  | Scores | 64 | -0.81 | -0.83 | -52 | -52 |
|  |  |  |  |  | No. >0 | 6 | 6 |
|  |  |  |  |  | No. <0 | 58 | 58 |

**Table S30c** New York (NyDEC, 2006) - Table 375-6.8(b) Restrict Residential

| Number | Pollutant | CAS. No | RGV (mg/kg) | C_2_ | C_3_ | C_4_ | C_5_ |
| --- | --- | --- | --- | --- | --- | --- | --- |
| 2 | Arsenic | 7440-38-2 | 1.60E+01 | 0 | 0 | -1 | 1 |
| 3 | Barium | 7440-39-3 | 3.50E+02 | 0 | 0 | -1 | -1 |
| 4 | Beryllium | 7440-41-7 | 1.40E+01 | 0 | 0 | -1 | -1 |
| 5 | Cadmium | 7440-43-9 | 2.50E+00 | 0 | 0 | -1 | -1 |
| 6 | Chromium (III) | 16065-83-1 | 3.60E+01 | -1 | -1 | -1 | -1 |
| 7 | Chromium (VI) | 18540-29-9 | 2.20E+01 | 0 | 0 | -1 | -1 |
| 9 | Copper | 7440-50-8 | 2.70E+02 | 0 | 0 | 1 | -1 |
| 10 | Lead | 7439-92-1 | 4.00E+02 | 0 | 1 | 1 | 1 |
| 11 | Manganese | 7439-96-5 | 2.00E+03 | 0 | 0 | 1 | 1 |
| 12 | Mercury | 7439-97-6 | 8.10E-01 | 0 | 0 | -1 | -1 |
| 14 | Nickel | 7440-02-0 | 1.40E+02 | 0 | 0 | 1 | -1 |
| 15 | Selenium | 7782-49-2 | 3.60E+01 | 0 | 0 | 1 | 1 |
| 16 | Silver | 7440-22-4 | 3.60E+01 | 0 | 0 | -1 | -1 |
| 20 | Zinc | 7440-66-6 | 2.20E+03 | 1 | 1 | 1 | 1 |
| 21 | Cyanide | 57-12-5 | 2.70E+01 | 0 | 0 | -1 | -1 |
| 23 | Dichloromethane | 75-09-2 | 5.10E+01 | 1 | 1 | 1 | 1 |
| 24 | Trichloromethane | 67-66-3 | 1.00E+01 | 1 | 1 | 1 | 1 |
| 25 | Tetrachloromethane | 56-23-5 | 1.40E+00 | 0 | 0 | 1 | 1 |
| 28 | 1,2-Dichloroethane | 107-06-2 | 2.30E+00 | 0 | 0 | 1 | 1 |
| 29 | 1,1,1-Trichloroethane | 71-55-6 | 1.00E+02 | 0 | 0 | -1 | -1 |
| 31 | Chloroethene | 75-01-4 | 2.10E-01 | 0 | 0 | 1 | -1 |
| 32 | 1,1-Dichloroethene | 75-35-4 | 1.00E+02 | 1 | 1 | 1 | 1 |
| 33 | Trichloroethene | 79-01-6 | 1.00E+01 | 1 | 1 | 1 | 1 |
| 34 | Tetrachloroethene | 127-18-4 | 5.50E+00 | 0 | 0 | 1 | 1 |
| 35 | Benzene | 71-43-2 | 2.90E+00 | 1 | 1 | 1 | 1 |
| 36 | Toluene | 108-88-3 | 1.00E+02 | 0 | 0 | -1 | -1 |
| 37 | Chlorobenzene | 108-90-7 | 1.00E+02 | 0 | 1 | 1 | 1 |
| 38 | 1,2-Dichlorobenzene | 95-50-1 | 1.00E+02 | 0 | 0 | -1 | 1 |
| 39 | 1,3-Dichlorobenzene | 541-73-1 | 1.70E+01 | 0 | 0 | -1 | -1 |
| 40 | 1,4-Dichlorobenzene | 106-46-7 | 9.80E+00 | 0 | 0 | 1 | 1 |
| 42 | Hexachlorobenzene | 118-74-1 | 3.30E-01 | 0 | 0 | -1 | -1 |
| 44 | Ethylbenzene | 100-41-4 | 3.00E+01 | 0 | 0 | 1 | -1 |
| 46 | Xylenes | 1330-20-7 | 1.00E+02 | 0 | 0 | -1 | -1 |
| 47 | Cresol | 1319-77-3 | 3.40E+01 | 0 | 0 | -1 | -1 |
| 48 | Phenol | 108-95-2 | 1.00E+02 | 0 | 0 | -1 | -1 |
| 54 | Pentachlorophenol | 87-86-5 | 2.40E+00 | 0 | 0 | -1 | -1 |
| 55 | Benz(a)anthracene | 56-55-3 | 1.00E+00 | 0 | 0 | -1 | -1 |
| 56 | Benzo(a)pyrene | 50-32-8 | 1.00E+00 | 0 | 0 | 1 | 1 |
| 57 | Benzo(b)fluoranthene | 205-99-2 | 1.00E+00 | 0 | 0 | -1 | -1 |
| 58 | Benzo(k)fluoranthene | 207-08-9 | 1.00E+00 | -1 | -1 | -1 | -1 |
| 59 | Chrysene | 218-01-9 | 1.00E+00 | -1 | -1 | -1 | -1 |
| 60 | Dibena(a,h)anthracene | 53-70-3 | 3.30E-01 | 0 | 0 | -1 | -1 |
| 61 | Indeno(1,2,3-c,d)pyrebe | 193-39-5 | 5.00E-01 | -1 | -1 | -1 | -1 |
| 62 | Naphthalene | 91-20-3 | 1.00E+02 | 1 | 2 | 1 | 1 |
| 63 | Acenaphehene | 83-32-9 | 1.00E+02 | 0 | 0 | 1 | -1 |
| 64 | Acenaphthylene | 208-96-8 | 1.00E+02 | 0 | 0 | 1 | 1 |
| 65 | Anthracene | 120-12-7 | 1.00E+02 | 0 | 0 | -1 | -1 |
| 66 | Benzo(g,h,i) perylene | 191-24-2 | 1.00E+02 | 1 | 0 | 1 | 1 |
| 67 | Flouranthene | 206-44-0 | 1.00E+02 | 1 | 0 | 1 | -1 |
| 68 | Flouorene | 86-73-7 | 1.00E+02 | 0 | 0 | -1 | -1 |
| 69 | Phenanthrene | 85-01-8 | 1.00E+02 | 1 | 0 | 1 | 1 |
| 70 | Pyrene | 129-00-0 | 1.00E+02 | 0 | 0 | -1 | -1 |
| 71 | Aldrin | 309-00-2 | 1.90E-02 | 0 | -1 | -1 | -1 |
| 72 | Chlordane | 57-74-9 | 9.10E-02 | -1 | -1 | -1 | -1 |
| 73 | DDT | 50-29-3 | 1.70E+00 | 0 | 0 | -1 | -1 |
| 74 | Dieldrin | 60-57-1 | 3.90E-02 | 0 | -1 | -1 | -1 |
| 75 | Endosulfan | 115-29-7 | 4.80E+00 | -1 | 0 | -1 | -1 |
| 76 | Endrin | 72-20-8 | 2.20E+00 | 0 | 0 | -1 | -1 |
| 77 | Heptachlor | 76-44-8 | 4.20E-01 | 1 | 0 | 1 | -1 |
| 78 | Lindane | 58-89-9 | 2.80E-01 | 0 | 0 | -1 | -1 |
| 92 | Total PCB | 1336-36-3 | 1.00E+00 | 0 | 0 | 1 | 1 |
| 95 | Methyl ethyl ketone | 78-93-3 | 1.00E+02 | -2 | -1 | -1 | -1 |
| 100 | Methyl tert-butyl ether | 1634-04-4 | 6.20E+01 | 0 | 0 | -1 | 1 |
|  |  | Overall | C1 | C2 | C3 | C4 | C5 |
|  |  | Scores | 63 | 0.048 | 0.032 | -9 | -17 |
|  |  |  |  |  | No. >0 | 27 | 23 |
|  |  |  |  |  | No. <0 | 36 | 40 |

**Table S31a** North Carolina Environmental Quality - Excavated Soil

| Number | Pollutant | CAS. No | RGV (mg/kg) | C_2_ | C_3_ | C_4_ | C_5_ |
| --- | --- | --- | --- | --- | --- | --- | --- |
| 1 | Antimony | 7440-36-0 | 5.40E+00 | 0 | 0 | -1 | -1 |
| 2 | Arsenic | 7440-38-2 | 3.90E-01 | -1 | -1 | -1 | -1 |
| 3 | Barium | 7440-39-3 | 8.50E+02 | 0 | 0 | -1 | -1 |
| 4 | Beryllium | 7440-41-7 | 3.40E+00 | -1 | -1 | -1 | -1 |
| 5 | Cadmium | 7440-43-9 | 9.50E-01 | -1 | -1 | -1 | -1 |
| 6 | Chromium (III) | 16065-83-1 | 5.50E+02 | 1 | 0 | 1 | -1 |
| 7 | Chromium (VI) | 18540-29-9 | 3.00E+01 | 0 | 0 | -1 | -1 |
| 8 | Cobalt | 7440-48-4 | 7.30E+00 | -1 | -1 | -1 | -1 |
| 9 | Copper | 7440-50-8 | 7.00E+02 | 1 | 1 | 1 | 1 |
| 10 | Lead | 7439-92-1 | 2.70E+02 | 0 | 0 | 1 | 1 |
| 12 | Mercury | 7439-97-6 | 1.50E-02 | -2 | -2 | -1 | -1 |
| 14 | Nickel | 7440-02-0 | 5.60E+01 | 0 | 0 | -1 | -1 |
| 15 | Selenium | 7782-49-2 | 1.20E+01 | 0 | 0 | -1 | -1 |
| 16 | Silver | 7440-22-4 | 2.20E-01 | -2 | -2 | -1 | -1 |
| 17 | Thallium | 7440-28-0 | 5.10E-01 | -1 | -1 | -1 | -1 |
| 18 | Tin | 7440-31-5 | 2.20E+02 | 0 | 0 | 1 | -1 |
| 19 | Vanadium | 7440-62-2 | 5.50E+02 | 1 | 1 | 1 | 1 |
| 20 | Zinc | 7440-66-6 | 5.50E+02 | 0 | 0 | -1 | -1 |
| 21 | Cyanide | 57-12-5 | 1.10E+01 | 0 | 0 | -1 | -1 |
| 22 | Acrylonitril | 107-13-1 | 3.90E-04 | -3 | -3 | -1 | -1 |
| 23 | Dichloromethane | 75-09-2 | 2.00E-02 | -2 | -3 | -1 | -1 |
| 24 | Trichloromethane | 67-66-3 | 2.20E-01 | 0 | -1 | -1 | -1 |
| 25 | Tetrachloromethane | 56-23-5 | 2.40E-03 | -2 | -2 | -1 | -1 |
| 26 | Bromoform | 75-25-2 | 2.90E-02 | -3 | -3 | -1 | -1 |
| 27 | Bromomethane | 74-83-9 | 2.00E-01 | -1 | -1 | -1 | -1 |
| 28 | 1,2-Dichloroethane | 107-06-2 | 1.80E-03 | -3 | -3 | -1 | -1 |
| 29 | 1,1,1-Trichloroethane | 71-55-6 | 1.70E+00 | -2 | -2 | -1 | -1 |
| 30 | 1,1,2-Trichloroethane | 79-00-5 | 1.70E-02 | -2 | -2 | -1 | -1 |
| 31 | Chloroethene | 75-01-4 | 9.50E-05 | -3 | -3 | -1 | -1 |
| 32 | 1,1-Dichloroethene | 75-35-4 | 4.40E-02 | -2 | -2 | -1 | -1 |
| 33 | Trichloroethene | 79-01-6 | 1.80E-02 | -2 | -2 | -1 | -1 |
| 34 | Tetrachloroethene | 127-18-4 | 7.40E-03 | -2 | -2 | -1 | -1 |
| 35 | Benzene | 71-43-2 | 5.60E-03 | -2 | -2 | -1 | -1 |
| 36 | Toluene | 108-88-3 | 7.30E+00 | -1 | -1 | -1 | -1 |
| 37 | Chlorobenzene | 108-90-7 | 4.40E-01 | -2 | -2 | -1 | -1 |
| 38 | 1,2-Dichlorobenzene | 95-50-1 | 2.80E-01 | -3 | -2 | -1 | -1 |
| 39 | 1,3-Dichlorobenzene | 541-73-1 | 6.50E+00 | 0 | 0 | -1 | -1 |
| 41 | 1,2,4Trichlorobenzene | 120-82-1 | 2.60E+00 | -1 | -1 | -1 | -1 |
| 42 | Hexachlorobenzene | 118-74-1 | 3.00E-02 | -1 | -1 | -1 | -1 |
| 43 | Nitrobenzene | 98-95-3 | 1.00E-01 | -2 | -2 | -1 | -1 |
| 44 | Ethylbenzene | 100-41-4 | 4.60E+00 | 0 | -1 | -1 | -1 |
| 45 | Styrene | 100-42-5 | 2.20E+00 | -1 | -1 | -1 | -1 |
| 46 | Xylenes | 1330-20-7 | 5.00E+00 | -1 | -1 | -1 | -1 |
| 47 | Cresol | 1319-77-3 | 1.70E-02 | -4 | -3 | -1 | -1 |
| 48 | Phenol | 108-95-2 | 1.80E+00 | -2 | -2 | -1 | -1 |
| 49 | 2-Chlorophenol | 95-57-8 | 4.30E-03 | -4 | -3 | -1 | -1 |
| 50 | 2,4-Dichlorophenol | 120-83-2 | 1.00E+00 | -1 | -1 | -1 | -1 |
| 51 | 2,4,5-Trichlorophenol | 95-95-4 | 4.05E+01 | -1 | 0 | -1 | -1 |
| 52 | 2,4,6-Trichlorophenol | 88-06-2 | 2.00E-01 | -2 | -2 | -1 | -1 |
| 53 | 2,3,4,6-Tetrahlorophenol | 58-90-2 | 2.10E+00 | -2 | -1 | -1 | -1 |
| 54 | Pentachlorophenol | 87-86-5 | 2.20E-02 | -2 | -2 | -1 | -1 |
| 55 | Benz(a)anthracene | 56-55-3 | 3.43E-01 | -1 | -1 | -1 | -1 |
| 56 | Benzo(a)pyrene | 50-32-8 | 6.20E-02 | -1 | -1 | -1 | -1 |
| 57 | Benzo(b)fluoranthene | 205-99-2 | 6.20E-01 | 0 | 0 | -1 | -1 |
| 58 | Benzo(k)fluoranthene | 207-08-9 | 6.20E+00 | 0 | 0 | -1 | -1 |
| 59 | Chrysene | 218-01-9 | 3.80E+01 | 0 | 0 | 1 | 1 |
| 60 | Dibena(a,h)anthracene | 53-70-3 | 6.20E-02 | -1 | -1 | -1 | -1 |
| 61 | Indeno(1,2,3-c,d)pyrebe | 193-39-5 | 6.20E-01 | 0 | -1 | -1 | -1 |
| 62 | Naphthalene | 91-20-3 | 5.80E-01 | -1 | -1 | -1 | -1 |
| 63 | Acenaphehene | 83-32-9 | 8.20E+00 | -1 | -1 | -1 | -1 |
| 64 | Acenaphthylene | 208-96-8 | 1.10E+01 | -1 | -1 | -1 | -1 |
| 65 | Anthracene | 120-12-7 | 1.00E+03 | 1 | 1 | 1 | 1 |
| 66 | Benzo(g,h,i) perylene | 191-24-2 | 4.70E+02 | 1 | 1 | 1 | 1 |
| 67 | Flouranthene | 206-44-0 | 2.80E+02 | 2 | 1 | 1 | 1 |
| 68 | Flouorene | 86-73-7 | 4.40E+01 | 0 | 0 | -1 | -1 |
| 69 | Phenanthrene | 85-01-8 | 6.00E+01 | 0 | 0 | 1 | -1 |
| 70 | Pyrene | 129-00-0 | 2.90E+02 | 0 | 1 | 1 | 1 |
| 71 | Aldrin | 309-00-2 | 2.90E-02 | 0 | -1 | -1 | -1 |
| 72 | Chlordane | 57-74-9 | 1.00E-01 | -1 | -1 | -1 | -1 |
| 73 | DDT | 50-29-3 | 1.40E+00 | 0 | 0 | -1 | -1 |
| 74 | Dieldrin | 60-57-1 | 1.10E-03 | -2 | -2 | -1 | -1 |
| 75 | Endosulfan | 115-29-7 | 1.80E+01 | 0 | 0 | -1 | 1 |
| 76 | Endrin | 72-20-8 | 4.40E-01 | -1 | -1 | -1 | -1 |
| 78 | Lindane | 58-89-9 | 6.20E-03 | -2 | -2 | -1 | -1 |
| 79 | Toxaphene | 8001-35-2 | 6.00E-02 | -1 | -1 | -1 | -1 |
| 80 | 2,4-D | 94-75-7 | 3.10E-01 | -3 | -3 | -1 | -1 |
| 81 | Atrazine | 1912-24-9 | 2.40E-02 | -2 | -2 | -1 | -1 |
| 86 | Gylphosate | 1071-83-6 | 3.60E+01 | -2 | -1 | -1 | -1 |
| 89 | Picloram | 1918-02-1 | 2.60E+01 | -2 | -1 | -1 | -1 |
| 90 | Simazine | 122-34-9 | 2.70E-02 | -2 | -2 | -1 | -1 |
| 92 | Total PCB | 1336-36-3 | 3.40E-04 | -3 | -3 | -1 | -1 |
| 94 | Total PCDD/PCDF | 1746-01-6 | 3.90E-06 | -1 | -1 | -1 | -1 |
| 95 | Methyl ethyl ketone | 78-93-3 | 1.70E+01 | -2 | -2 | -1 | -1 |
| 96 | Methyl isobutyl ketone | 108-10-1 | 8.10E+00 | -2 | -2 | -1 | -1 |
| 97 | Dibutyl phthalate | 84-74-2 | 2.50E+01 | -3 | -2 | -1 | -1 |
| 98 | DEHP | 117-81-7 | 6.70E+00 | -1 | -1 | -1 | -1 |
| 99 | Hexachlorobutadine | 87-68-3 | 2.57E-01 | -1 | -1 | -1 | -1 |
| 100 | Methyl tert-butyl ether | 1634-04-4 | 9.20E-01 | -2 | -1 | -1 | -1 |
|  |  | Overall | C1 | C2 | C3 | C4 | C5 |
|  |  | Scores | 88 | -1.14 | -1.10 | -66 | -70 |
|  |  |  |  |  | No. >0 | 11 | 9 |
|  |  |  |  |  | No. <0 | 77 | 79 |

**Table S31b** North Carolina - MSCCs Residential

| Number | Pollutant | CAS. No | RGV (mg/kg) | C_2_ | C_3_ | C_4_ | C_5_ |
| --- | --- | --- | --- | --- | --- | --- | --- |
| 3 | Barium | 7440-39-3 | 3.10E+03 | 1 | 1 | 1 | 1 |
| 6 | Chromium (III) | 16065-83-1 | 2.35E+04 | 2 | 2 | 1 | 1 |
| 7 | Chromium (VI) | 18540-29-9 | 4.70E+01 | 0 | 0 | -1 | -1 |
| 10 | Lead | 7439-92-1 | 4.00E+02 | 0 | 1 | 1 | 1 |
| 16 | Silver | 7440-22-4 | 7.82E+01 | 0 | 0 | 1 | 1 |
| 23 | Dichloromethane | 75-09-2 | 8.50E+01 | 1 | 1 | 1 | 1 |
| 24 | Trichloromethane | 67-66-3 | 2.00E+01 | 1 | 1 | 1 | 1 |
| 26 | Bromoform | 75-25-2 | 8.10E+01 | 0 | 0 | 1 | 1 |
| 27 | Bromomethane | 74-83-9 | 2.20E+01 | 1 | 1 | 1 | 1 |
| 28 | 1,2-Dichloroethane | 107-06-2 | 7.00E+00 | 1 | 1 | 1 | 1 |
| 29 | 1,1,1-Trichloroethane | 71-55-6 | 3.10E+04 | 2 | 2 | 1 | 1 |
| 30 | 1,1,2-Trichloroethane | 79-00-5 | 1.00E+01 | 1 | 1 | 1 | 1 |
| 31 | Chloroethene | 75-01-4 | 4.60E-01 | 1 | 0 | 1 | 1 |
| 32 | 1,1-Dichloroethene | 75-35-4 | 7.80E+02 | 2 | 2 | 1 | 1 |
| 33 | Trichloroethene | 79-01-6 | 4.60E+00 | 0 | 0 | -1 | 1 |
| 34 | Tetrachloroethene | 127-18-4 | 1.10E+00 | 0 | 0 | -1 | -1 |
| 35 | Benzene | 71-43-2 | 1.80E+01 | 2 | 1 | 1 | 1 |
| 36 | Toluene | 108-88-3 | 1.20E+03 | 1 | 1 | 1 | 1 |
| 37 | Chlorobenzene | 108-90-7 | 3.12E+02 | 1 | 1 | 1 | 1 |
| 38 | 1,2-Dichlorobenzene | 95-50-1 | 1.40E+03 | 1 | 2 | 1 | 1 |
| 39 | 1,3-Dichlorobenzene | 541-73-1 | 4.60E+02 | 1 | 2 | 1 | 1 |
| 40 | 1,4-Dichlorobenzene | 106-46-7 | 1.10E+02 | 1 | 1 | 1 | 1 |
| 41 | 1,2,4Trichlorobenzene | 120-82-1 | 1.56E+02 | 1 | 1 | 1 | 1 |
| 44 | Ethylbenzene | 100-41-4 | 1.56E+03 | 2 | 2 | 1 | 1 |
| 45 | Styrene | 100-42-5 | 3.13E+03 | 2 | 2 | 1 | 1 |
| 46 | Xylenes | 1330-20-7 | 3.13E+03 | 2 | 2 | 1 | 1 |
| 47 | Cresol | 1319-77-3 | 7.00E+02 | 1 | 1 | 1 | 1 |
| 48 | Phenol | 108-95-2 | 4.69E+03 | 2 | 2 | 1 | 1 |
| 50 | 2,4-Dichlorophenol | 120-83-2 | 4.00E+01 | 1 | 1 | 1 | 1 |
| 52 | 2,4,6-Trichlorophenol | 88-06-2 | 1.00E+01 | 0 | 0 | -1 | -1 |
| 54 | Pentachlorophenol | 87-86-5 | 5.00E+00 | 0 | 0 | -1 | 1 |
| 55 | Benz(a)anthracene | 56-55-3 | 8.80E-01 | 0 | 0 | -1 | -1 |
| 56 | Benzo(a)pyrene | 50-32-8 | 8.80E-02 | -1 | -1 | -1 | -1 |
| 57 | Benzo(b)fluoranthene | 205-99-2 | 8.80E-01 | 0 | 0 | -1 | -1 |
| 58 | Benzo(k)fluoranthene | 207-08-9 | 9.00E+00 | 0 | 0 | 1 | 1 |
| 59 | Chrysene | 218-01-9 | 8.80E+01 | 1 | 1 | 1 | 1 |
| 60 | Dibena(a,h)anthracene | 53-70-3 | 8.80E-02 | -1 | -1 | -1 | -1 |
| 61 | Indeno(1,2,3-c,d)pyrebe | 193-39-5 | 8.80E-01 | 0 | 0 | -1 | -1 |
| 62 | Naphthalene | 91-20-3 | 3.13E+02 | 1 | 2 | 1 | 1 |
| 63 | Acenaphehene | 83-32-9 | 9.40E+02 | 1 | 1 | 1 | 1 |
| 64 | Acenaphthylene | 208-96-8 | 4.69E+02 | 1 | 1 | 1 | 1 |
| 65 | Anthracene | 120-12-7 | 4.60E+03 | 2 | 2 | 1 | 1 |
| 66 | Benzo(g,h,i) perylene | 191-24-2 | 4.69E+02 | 1 | 1 | 1 | 1 |
| 67 | Flouranthene | 206-44-0 | 6.20E+02 | 2 | 1 | 1 | 1 |
| 68 | Flouorene | 86-73-7 | 6.20E+02 | 1 | 1 | 1 | 1 |
| 69 | Phenanthrene | 85-01-8 | 4.69E+02 | 1 | 1 | 1 | 1 |
| 70 | Pyrene | 129-00-0 | 4.69E+02 | 1 | 1 | 1 | 1 |
| 95 | Methyl ethyl ketone | 78-93-3 | 9.39E+03 | 0 | 1 | 1 | 1 |
| 96 | Methyl isobutyl ketone | 108-10-1 | 1.20E+03 | 0 | 0 | -1 | 1 |
| 98 | DEHP | 117-81-7 | 4.60E+01 | 0 | 0 | -1 | -1 |
| 99 | Hexachlorobutadine | 87-68-3 | 4.60E+00 | 0 | 0 | -1 | 1 |
| 100 | Methyl tert-butyl ether | 1634-04-4 | 3.50E+02 | 1 | 1 | 1 | 1 |
|  |  | Overall | C1 | C2 | C3 | C4 | C5 |
|  |  | Scores | 52 | 0.81 | 0.85 | 26 | 34 |
|  |  |  |  |  | No. >0 | 39 | 43 |
|  |  |  |  |  | No. <0 | 13 | 9 |

**Table S31c** North Carolina - Preliminary Soil Remediation Goals (PSRG)

| Number | Pollutant | CAS. No | RGV (mg/kg) | C_2_ | C_3_ | C_4_ | C_5_ |
| --- | --- | --- | --- | --- | --- | --- | --- |
| 1 | Antimony | 7440-36-0 | 6.20E+00 | 0 | 0 | -1 | -1 |
| 2 | Arsenic | 7440-38-2 | 6.80E-01 | -1 | -1 | -1 | -1 |
| 3 | Barium | 7440-39-3 | 3.00E+03 | 1 | 1 | 1 | 1 |
| 4 | Beryllium | 7440-41-7 | 3.20E+01 | 0 | 0 | 1 | 1 |
| 5 | Cadmium | 7440-43-9 | 1.42E+01 | 1 | 1 | 1 | 1 |
| 6 | Chromium (III) | 16065-83-1 | 2.40E+04 | 2 | 2 | 1 | 1 |
| 7 | Chromium (VI) | 18540-29-9 | 3.00E-01 | -2 | -2 | -1 | -1 |
| 8 | Cobalt | 7440-48-4 | 4.60E+00 | -1 | -1 | -1 | -1 |
| 9 | Copper | 7440-50-8 | 6.20E+02 | 1 | 1 | 1 | 1 |
| 10 | Lead | 7439-92-1 | 4.00E+02 | 0 | 1 | 1 | 1 |
| 11 | Manganese | 7439-96-5 | 3.60E+02 | 0 | 0 | -1 | -1 |
| 12 | Mercury | 7439-97-6 | 2.20E+00 | 0 | 0 | -1 | -1 |
| 13 | Molybdenum | 7439-98-7 | 7.80E+01 | 0 | 0 | 1 | 1 |
| 14 | Nickel | 7440-02-0 | 3.00E+02 | 1 | 1 | 1 | 1 |
| 15 | Selenium | 7782-49-2 | 7.80E+01 | 1 | 1 | 1 | 1 |
| 16 | Silver | 7440-22-4 | 7.80E+01 | 0 | 0 | 1 | 1 |
| 17 | Thallium | 7440-28-0 | 1.56E-01 | -1 | -1 | -1 | -1 |
| 18 | Tin | 7440-31-5 | 9.40E+03 | 2 | 1 | 1 | 1 |
| 19 | Vanadium | 7440-62-2 | 7.80E+01 | 0 | 0 | -1 | -1 |
| 20 | Zinc | 7440-66-6 | 4.60E+03 | 1 | 1 | 1 | 1 |
| 21 | Cyanide | 57-12-5 | 3.20E+00 | -1 | -1 | -1 | -1 |
| 22 | Acrylonitril | 107-13-1 | 2.50E-01 | -1 | -1 | -1 | -1 |
| 23 | Dichloromethane | 75-09-2 | 5.70E+01 | 1 | 1 | 1 | 1 |
| 24 | Trichloromethane | 67-66-3 | 3.20E-01 | 0 | -1 | -1 | -1 |
| 25 | Tetrachloromethane | 56-23-5 | 6.50E-01 | 0 | 0 | -1 | -1 |
| 26 | Bromoform | 75-25-2 | 1.90E+01 | 0 | 0 | -1 | -1 |
| 27 | Bromomethane | 74-83-9 | 1.36E+00 | 0 | 0 | -1 | -1 |
| 29 | 1,1,1-Trichloroethane | 71-55-6 | 6.40E+02 | 0 | 1 | 1 | 1 |
| 30 | 1,1,2-Trichloroethane | 79-00-5 | 3.00E-01 | -1 | -1 | -1 | -1 |
| 31 | Chloroethene | 75-01-4 | 5.90E-02 | 0 | 0 | -1 | -1 |
| 32 | 1,1-Dichloroethene | 75-35-4 | 4.60E+01 | 1 | 1 | 1 | 1 |
| 33 | Trichloroethene | 79-01-6 | 8.20E-01 | -1 | 0 | -1 | -1 |
| 34 | Tetrachloroethene | 127-18-4 | 1.62E+01 | 1 | 1 | 1 | 1 |
| 35 | Benzene | 71-43-2 | 1.20E+00 | 0 | 0 | 1 | 1 |
| 36 | Toluene | 108-88-3 | 8.18E+02 | 1 | 1 | 1 | 1 |
| 37 | Chlorobenzene | 108-90-7 | 5.60E+01 | 0 | 1 | -1 | 1 |
| 38 | 1,2-Dichlorobenzene | 95-50-1 | 3.60E+02 | 0 | 1 | 1 | 1 |
| 40 | 1,4-Dichlorobenzene | 106-46-7 | 2.60E+00 | 0 | 0 | -1 | -1 |
| 41 | 1,2,4Trichlorobenzene | 120-82-1 | 1.16E+01 | 0 | 0 | -1 | -1 |
| 42 | Hexachlorobenzene | 118-74-1 | 2.10E-01 | 0 | 0 | -1 | -1 |
| 43 | Nitrobenzene | 98-95-3 | 5.10E+00 | 0 | 0 | -1 | -1 |
| 44 | Ethylbenzene | 100-41-4 | 5.80E+00 | 0 | 0 | -1 | -1 |
| 45 | Styrene | 100-42-5 | 8.67E+02 | 1 | 1 | 1 | 1 |
| 46 | Xylenes | 1330-20-7 | 1.16E+02 | 0 | 0 | -1 | -1 |
| 47 | Cresol | 1319-77-3 | 1.26E+03 | 1 | 2 | 1 | 1 |
| 48 | Phenol | 108-95-2 | 3.80E+03 | 2 | 1 | 1 | 1 |
| 49 | 2-Chlorophenol | 95-57-8 | 7.80E+01 | 1 | 1 | 1 | 1 |
| 50 | 2,4-Dichlorophenol | 120-83-2 | 3.80E+01 | 1 | 1 | 1 | 1 |
| 51 | 2,4,5-Trichlorophenol | 95-95-4 | 1.26E+03 | 1 | 1 | 1 | 1 |
| 52 | 2,4,6-Trichlorophenol | 88-06-2 | 1.26E+01 | 0 | 0 | 1 | -1 |
| 53 | 2,3,4,6-Tetrahlorophenol | 58-90-2 | 3.80E+02 | 1 | 1 | 1 | 1 |
| 54 | Pentachlorophenol | 87-86-5 | 1.00E+00 | 0 | 0 | -1 | -1 |
| 55 | Benz(a)anthracene | 56-55-3 | 1.60E-01 | -1 | -1 | -1 | -1 |
| 56 | Benzo(a)pyrene | 50-32-8 | 1.60E-02 | -1 | -1 | -1 | -1 |
| 57 | Benzo(b)fluoranthene | 205-99-2 | 1.60E-01 | -1 | -1 | -1 | -1 |
| 58 | Benzo(k)fluoranthene | 207-08-9 | 1.60E+00 | 0 | 0 | -1 | -1 |
| 59 | Chrysene | 218-01-9 | 1.60E+01 | 0 | 0 | -1 | -1 |
| 60 | Dibena(a,h)anthracene | 53-70-3 | 1.60E-02 | -1 | -1 | -1 | -1 |
| 61 | Indeno(1,2,3-c,d)pyrebe | 193-39-5 | 1.60E-01 | -1 | -1 | -1 | -1 |
| 62 | Naphthalene | 91-20-3 | 3.80E+00 | 0 | 0 | -1 | 1 |
| 63 | Acenaphehene | 83-32-9 | 7.20E+02 | 1 | 1 | 1 | 1 |
| 65 | Anthracene | 120-12-7 | 3.60E+03 | 2 | 1 | 1 | 1 |
| 67 | Flouranthene | 206-44-0 | 4.80E+02 | 2 | 1 | 1 | 1 |
| 68 | Flouorene | 86-73-7 | 4.80E+02 | 1 | 1 | 1 | 1 |
| 70 | Pyrene | 129-00-0 | 3.60E+02 | 1 | 1 | 1 | 1 |
| 71 | Aldrin | 309-00-2 | 3.90E-02 | 0 | -1 | -1 | -1 |
| 72 | Chlordane | 57-74-9 | 1.70E+00 | 0 | 0 | -1 | -1 |
| 73 | DDT | 50-29-3 | 1.90E+00 | 0 | 0 | -1 | -1 |
| 74 | Dieldrin | 60-57-1 | 3.40E-02 | 0 | -1 | -1 | -1 |
| 75 | Endosulfan | 115-29-7 | 9.40E+01 | 1 | 1 | 1 | 1 |
| 76 | Endrin | 72-20-8 | 3.80E+00 | 0 | 0 | -1 | 1 |
| 77 | Heptachlor | 76-44-8 | 1.30E-01 | 0 | 0 | -1 | -1 |
| 78 | Lindane | 58-89-9 | 5.70E-01 | 0 | 0 | 1 | 1 |
| 79 | Toxaphene | 8001-35-2 | 4.90E-01 | 0 | 0 | -1 | -1 |
| 80 | 2,4-D | 94-75-7 | 1.40E+02 | 0 | 0 | -1 | -1 |
| 81 | Atrazine | 1912-24-9 | 2.40E+00 | 0 | 0 | -1 | -1 |
| 82 | Carbaryl | 63-25-2 | 1.26E+03 | 0 | 1 | -1 | 1 |
| 83 | Carbofuran | 1563-66-2 | 6.40E+01 | 0 | 1 | -1 | 1 |
| 84 | Chloryrifos | 2921-88-2 | 1.26E+01 | -1 | -1 | -1 | -1 |
| 85 | Diuron | 330-54-1 | 2.60E+01 | 0 | 0 | -1 | -1 |
| 86 | Gylphosate | 1071-83-6 | 1.26E+03 | 0 | 0 | -1 | 1 |
| 87 | Malathion | 121-75-5 | 2.60E+02 | 0 | 0 | -1 | -1 |
| 88 | MCPA | 94-74-6 | 6.40E+00 | 0 | 0 | -1 | -1 |
| 89 | Picloram | 1918-02-1 | 8.80E+02 | 0 | 0 | -1 | -1 |
| 90 | Simazine | 122-34-9 | 4.50E+00 | 0 | 0 | 1 | -1 |
| 91 | Trifluralin | 1582-09-8 | 9.00E+01 | 0 | 0 | -1 | -1 |
| 92 | Total PCB | 1336-36-3 | 1.30E-01 | -1 | 0 | -1 | -1 |
| 93 | PCB 118 | 31508-00-6 | 1.20E-01 | 0 | 0 | -1 | -1 |
| 94 | Total PCDD/PCDF | 1746-01-6 | 4.80E-06 | -1 | -1 | -1 | -1 |
| 95 | Methyl ethyl ketone | 78-93-3 | 5.40E+03 | 0 | 1 | -1 | 1 |
| 96 | Methyl isobutyl ketone | 108-10-1 | 3.36E+03 | 0 | 1 | -1 | 1 |
| 97 | Dibutyl phthalate | 84-74-2 | 1.26E+03 | 0 | 1 | -1 | 1 |
| 98 | DEHP | 117-81-7 | 3.90E+01 | 0 | 0 | -1 | -1 |
| 99 | Hexachlorobutadine | 87-68-3 | 1.20E+00 | 0 | 0 | -1 | -1 |
| 100 | Methyl tert-butyl ether | 1634-04-4 | 4.70E+01 | 0 | 0 | -1 | 1 |
|  |  | Overall | C1 | C2 | C3 | C4 | C5 |
|  |  | Scores | 95 | 0.14 | 0.19 | -25 | -9 |
|  |  |  |  |  | No. >0 | 35 | 43 |
|  |  |  |  |  | No. <0 | 60 | 52 |

**Table S32a** Ohio Administrative Code - Generic

| Number | Pollutant | CAS. No | RGV (mg/kg) | C_2_ | C_3_ | C_4_ | C_5_ |
| --- | --- | --- | --- | --- | --- | --- | --- |
| 1 | Antimony | 7440-36-0 | 3.00E+01 | 0 | 1 | 1 | 1 |
| 2 | Arsenic | 7440-38-2 | 6.70E+00 | 0 | 0 | -1 | -1 |
| 3 | Barium | 7440-39-3 | 1.50E+04 | 1 | 1 | 1 | 1 |
| 4 | Beryllium | 7440-41-7 | 1.50E+02 | 1 | 1 | 1 | 1 |
| 5 | Cadmium | 7440-43-9 | 7.20E+01 | 1 | 1 | 1 | 1 |
| 6 | Chromium (III) | 16065-83-1 | 1.10E+05 | 3 | 2 | 1 | 1 |
| 7 | Chromium (VI) | 18540-29-9 | 2.30E+02 | 1 | 1 | 1 | 1 |
| 8 | Cobalt | 7440-48-4 | 1.40E+03 | 2 | 2 | 1 | 1 |
| 12 | Mercury | 7439-97-6 | 7.60E+00 | 1 | 1 | 1 | 1 |
| 14 | Nickel | 7440-02-0 | 1.50E+03 | 1 | 1 | 1 | 1 |
| 15 | Selenium | 7782-49-2 | 3.80E+02 | 1 | 1 | 1 | 1 |
| 16 | Silver | 7440-22-4 | 3.80E+02 | 1 | 1 | 1 | 1 |
| 17 | Thallium | 7440-28-0 | 6.10E+00 | 1 | 1 | 1 | 1 |
| 19 | Vanadium | 7440-62-2 | 6.80E+02 | 1 | 1 | 1 | 1 |
| 20 | Zinc | 7440-66-6 | 2.30E+04 | 2 | 2 | 1 | 1 |
| 21 | Cyanide | 57-12-5 | 1.50E+03 | 2 | 2 | 1 | 1 |
| 22 | Acrylonitril | 107-13-1 | 6.60E+00 | 1 | 1 | 1 | 1 |
| 23 | Dichloromethane | 75-09-2 | 2.50E+02 | 2 | 2 | 1 | 1 |
| 24 | Trichloromethane | 67-66-3 | 6.60E+00 | 1 | 1 | 1 | 1 |
| 25 | Tetrachloromethane | 56-23-5 | 5.50E+00 | 1 | 1 | 1 | 1 |
| 28 | 1,2-Dichloroethane | 107-06-2 | 8.70E+00 | 1 | 1 | 1 | 1 |
| 29 | 1,1,1-Trichloroethane | 71-55-6 | 1.30E+03 | 1 | 1 | 1 | 1 |
| 30 | 1,1,2-Trichloroethane | 79-00-5 | 2.50E+01 | 1 | 1 | 1 | 1 |
| 31 | Chloroethene | 75-01-4 | 4.60E+00 | 2 | 1 | 1 | 1 |
| 32 | 1,1-Dichloroethene | 75-35-4 | 4.10E+02 | 2 | 2 | 1 | 1 |
| 33 | Trichloroethene | 79-01-6 | 6.50E+01 | 1 | 2 | 1 | 1 |
| 34 | Tetrachloroethene | 127-18-4 | 1.70E+01 | 1 | 1 | 1 | 1 |
| 35 | Benzene | 71-43-2 | 6.40E+01 | 2 | 2 | 1 | 1 |
| 36 | Toluene | 108-88-3 | 5.20E+02 | 1 | 1 | 1 | 1 |
| 37 | Chlorobenzene | 108-90-7 | 4.10E+02 | 1 | 1 | 1 | 1 |
| 38 | 1,2-Dichlorobenzene | 95-50-1 | 3.70E+02 | 1 | 1 | 1 | 1 |
| 40 | 1,4-Dichlorobenzene | 106-46-7 | 6.00E+01 | 1 | 1 | 1 | 1 |
| 42 | Hexachlorobenzene | 118-74-1 | 5.20E+00 | 1 | 1 | 1 | 1 |
| 43 | Nitrobenzene | 98-95-3 | 2.70E+01 | 0 | 1 | 1 | 1 |
| 44 | Ethylbenzene | 100-41-4 | 2.30E+02 | 1 | 1 | 1 | 1 |
| 45 | Styrene | 100-42-5 | 1.70E+03 | 2 | 2 | 1 | 1 |
| 46 | Xylenes | 1330-20-7 | 3.70E+02 | 1 | 1 | 1 | 1 |
| 47 | Cresol | 1319-77-3 | 3.10E+02 | 0 | 1 | 1 | 1 |
| 48 | Phenol | 108-95-2 | 1.50E+04 | 2 | 2 | 1 | 1 |
| 51 | 2,4,5-Trichlorophenol | 95-95-4 | 6.30E+03 | 1 | 2 | 1 | 1 |
| 52 | 2,4,6-Trichlorophenol | 88-06-2 | 7.70E+02 | 2 | 2 | 1 | 1 |
| 54 | Pentachlorophenol | 87-86-5 | 5.50E+01 | 1 | 1 | 1 | 1 |
| 55 | Benz(a)anthracene | 56-55-3 | 1.10E+01 | 1 | 1 | 1 | 1 |
| 56 | Benzo(a)pyrene | 50-32-8 | 1.10E+00 | 0 | 0 | 1 | 1 |
| 57 | Benzo(b)fluoranthene | 205-99-2 | 1.10E+01 | 1 | 1 | 1 | 1 |
| 58 | Benzo(k)fluoranthene | 207-08-9 | 1.10E+02 | 1 | 1 | 1 | 1 |
| 59 | Chrysene | 218-01-9 | 1.10E+03 | 2 | 2 | 1 | 1 |
| 60 | Dibena(a,h)anthracene | 53-70-3 | 1.10E+00 | 1 | 0 | 1 | 1 |
| 61 | Indeno(1,2,3-c,d)pyrebe | 193-39-5 | 1.10E+01 | 1 | 1 | 1 | 1 |
| 62 | Naphthalene | 91-20-3 | 6.90E+01 | 1 | 2 | 1 | 1 |
| 63 | Acenaphehene | 83-32-9 | 3.50E+03 | 2 | 2 | 1 | 1 |
| 65 | Anthracene | 120-12-7 | 1.80E+04 | 2 | 2 | 1 | 1 |
| 67 | Flouranthene | 206-44-0 | 2.40E+03 | 3 | 2 | 1 | 1 |
| 68 | Flouorene | 86-73-7 | 2.40E+03 | 2 | 1 | 1 | 1 |
| 70 | Pyrene | 129-00-0 | 1.80E+03 | 1 | 1 | 1 | 1 |
| 72 | Chlordane | 57-74-9 | 2.80E+01 | 1 | 1 | 1 | 1 |
| 73 | DDT | 50-29-3 | 3.00E+01 | 1 | 1 | 1 | 1 |
| 76 | Endrin | 72-20-8 | 1.90E+01 | 1 | 1 | 1 | 1 |
| 77 | Heptachlor | 76-44-8 | 1.80E+00 | 1 | 1 | 1 | 1 |
| 78 | Lindane | 58-89-9 | 8.70E+00 | 1 | 1 | 1 | 1 |
| 79 | Toxaphene | 8001-35-2 | 7.80E+00 | 1 | 1 | 1 | 1 |
| 80 | 2,4-D | 94-75-7 | 6.30E+02 | 0 | 1 | -1 | 1 |
| 92 | Total PCB | 1336-36-3 | 1.20E+00 | 0 | 1 | 1 | 1 |
| 95 | Methyl ethyl ketone | 78-93-3 | 3.70E+04 | 1 | 2 | 1 | 1 |
| 96 | Methyl isobutyl ketone | 108-10-1 | 5.80E+03 | 0 | 1 | 1 | 1 |
| 97 | Dibutyl phthalate | 84-74-2 | 1.10E+02 | 0 | 1 | -1 | -1 |
| 98 | DEHP | 117-81-7 | 1.90E+02 | 1 | 0 | 1 | 1 |
| 100 | Methyl tert-butyl ether | 1634-04-4 | 8.50E+02 | 1 | 2 | 1 | 1 |
|  |  | Overall | C1 | C2 | C3 | C4 | C5 |
|  |  | Scores | 68 | 1.13 | 1.22 | 62 | 64 |
|  |  |  |  |  | No. >0 | 65 | 66 |
|  |  |  |  |  | No. <0 | 3 | 2 |

**Table S32b** Ohio Environmental Protection Agency - Generic Numerical Standards

| Number | Pollutant | CAS. No | RGV (mg/kg) | C_2_ | C_3_ | C_4_ | C_5_ |
| --- | --- | --- | --- | --- | --- | --- | --- |
| 1 | Antimony | 7440-36-0 | 6.30E+01 | 1 | 1 | 1 | 1 |
| 3 | Barium | 7440-39-3 | 3.00E+04 | 2 | 2 | 1 | 1 |
| 4 | Beryllium | 7440-41-7 | 3.10E+02 | 1 | 1 | 1 | 1 |
| 5 | Cadmium | 7440-43-9 | 1.40E+02 | 2 | 2 | 1 | 1 |
| 6 | Chromium (III) | 16065-83-1 | 2.30E+05 | 3 | 3 | 1 | 1 |
| 7 | Chromium (VI) | 18540-29-9 | 2.40E+01 | 0 | 0 | -1 | -1 |
| 8 | Cobalt | 7440-48-4 | 4.70E+01 | 0 | 0 | -1 | -1 |
| 9 | Copper | 7440-50-8 | 6.30E+03 | 2 | 2 | 1 | 1 |
| 12 | Mercury | 7439-97-6 | 9.70E+00 | 1 | 1 | 1 | 1 |
| 15 | Selenium | 7782-49-2 | 7.80E+02 | 2 | 2 | 1 | 1 |
| 16 | Silver | 7440-22-4 | 7.80E+02 | 1 | 1 | 1 | 1 |
| 17 | Thallium | 7440-28-0 | 1.60E+00 | 0 | 0 | -1 | -1 |
| 19 | Vanadium | 7440-62-2 | 7.90E+02 | 1 | 1 | 1 | 1 |
| 20 | Zinc | 7440-66-6 | 4.70E+04 | 2 | 2 | 1 | 1 |
| 21 | Cyanide | 57-12-5 | 5.00E+01 | 0 | 0 | 1 | 1 |
| 22 | Acrylonitril | 107-13-1 | 5.70E+00 | 1 | 1 | 1 | 1 |
| 23 | Dichloromethane | 75-09-2 | 7.50E+02 | 2 | 2 | 1 | 1 |
| 24 | Trichloromethane | 67-66-3 | 7.40E+00 | 1 | 1 | 1 | 1 |
| 25 | Tetrachloromethane | 56-23-5 | 1.50E+01 | 1 | 1 | 1 | 1 |
| 26 | Bromoform | 75-25-2 | 1.20E+03 | 1 | 2 | 1 | 1 |
| 27 | Bromomethane | 74-83-9 | 1.80E+01 | 1 | 1 | 1 | 1 |
| 28 | 1,2-Dichloroethane | 107-06-2 | 1.10E+01 | 1 | 1 | 1 | 1 |
| 29 | 1,1,1-Trichloroethane | 71-55-6 | 2.20E+04 | 2 | 2 | 1 | 1 |
| 30 | 1,1,2-Trichloroethane | 79-00-5 | 2.60E+01 | 1 | 1 | 1 | 1 |
| 31 | Chloroethene | 75-01-4 | 1.30E+00 | 1 | 1 | 1 | 1 |
| 32 | 1,1-Dichloroethene | 75-35-4 | 3.60E+02 | 2 | 2 | 1 | 1 |
| 33 | Trichloroethene | 79-01-6 | 1.10E+01 | 1 | 1 | 1 | 1 |
| 34 | Tetrachloroethene | 127-18-4 | 2.10E+02 | 2 | 2 | 1 | 1 |
| 35 | Benzene | 71-43-2 | 2.60E+01 | 2 | 2 | 1 | 1 |
| 36 | Toluene | 108-88-3 | 1.00E+04 | 2 | 2 | 1 | 1 |
| 37 | Chlorobenzene | 108-90-7 | 7.00E+02 | 1 | 2 | 1 | 1 |
| 38 | 1,2-Dichlorobenzene | 95-50-1 | 4.50E+03 | 2 | 2 | 1 | 1 |
| 40 | 1,4-Dichlorobenzene | 106-46-7 | 6.10E+01 | 1 | 1 | 1 | 1 |
| 41 | 1,2,4Trichlorobenzene | 120-82-1 | 1.50E+02 | 1 | 1 | 1 | 1 |
| 42 | Hexachlorobenzene | 118-74-1 | 6.10E+00 | 1 | 1 | 1 | 1 |
| 43 | Nitrobenzene | 98-95-3 | 1.20E+02 | 1 | 1 | 1 | 1 |
| 44 | Ethylbenzene | 100-41-4 | 1.30E+02 | 1 | 1 | 1 | 1 |
| 45 | Styrene | 100-42-5 | 1.40E+04 | 2 | 2 | 1 | 1 |
| 46 | Xylenes | 1330-20-7 | 1.60E+03 | 1 | 1 | 1 | 1 |
| 47 | Cresol | 1319-77-3 | 1.20E+04 | 2 | 2 | 1 | 1 |
| 48 | Phenol | 108-95-2 | 3.70E+04 | 3 | 2 | 1 | 1 |
| 49 | 2-Chlorophenol | 95-57-8 | 7.80E+02 | 2 | 2 | 1 | 1 |
| 50 | 2,4-Dichlorophenol | 120-83-2 | 3.70E+02 | 2 | 2 | 1 | 1 |
| 51 | 2,4,5-Trichlorophenol | 95-95-4 | 1.20E+04 | 2 | 2 | 1 | 1 |
| 52 | 2,4,6-Trichlorophenol | 88-06-2 | 1.20E+02 | 1 | 1 | 1 | 1 |
| 53 | 2,3,4,6-Tetrahlorophenol | 58-90-2 | 3.70E+03 | 2 | 2 | 1 | 1 |
| 54 | Pentachlorophenol | 87-86-5 | 1.80E+01 | 1 | 1 | 1 | 1 |
| 55 | Benz(a)anthracene | 56-55-3 | 1.20E+01 | 1 | 1 | 1 | 1 |
| 56 | Benzo(a)pyrene | 50-32-8 | 1.24E+00 | 0 | 1 | 1 | 1 |
| 57 | Benzo(b)fluoranthene | 205-99-2 | 1.20E+01 | 1 | 1 | 1 | 1 |
| 58 | Benzo(k)fluoranthene | 207-08-9 | 1.20E+02 | 1 | 1 | 1 | 1 |
| 59 | Chrysene | 218-01-9 | 1.20E+03 | 2 | 2 | 1 | 1 |
| 60 | Dibena(a,h)anthracene | 53-70-3 | 1.20E+00 | 1 | 0 | 1 | 1 |
| 61 | Indeno(1,2,3-c,d)pyrebe | 193-39-5 | 1.20E+01 | 1 | 1 | 1 | 1 |
| 62 | Naphthalene | 91-20-3 | 9.00E+01 | 1 | 2 | 1 | 1 |
| 63 | Acenaphehene | 83-32-9 | 6.90E+03 | 2 | 2 | 1 | 1 |
| 64 | Acenaphthylene | 208-96-8 | 6.90E+03 | 2 | 2 | 1 | 1 |
| 65 | Anthracene | 120-12-7 | 3.40E+04 | 3 | 2 | 1 | 1 |
| 66 | Benzo(g,h,i) perylene | 191-24-2 | 3.40E+03 | 2 | 2 | 1 | 1 |
| 67 | Flouranthene | 206-44-0 | 4.60E+03 | 3 | 2 | 1 | 1 |
| 68 | Flouorene | 86-73-7 | 4.60E+03 | 2 | 2 | 1 | 1 |
| 69 | Phenanthrene | 85-01-8 | 3.40E+04 | 3 | 3 | 1 | 1 |
| 70 | Pyrene | 129-00-0 | 3.40E+03 | 2 | 2 | 1 | 1 |
| 71 | Aldrin | 309-00-2 | 5.70E-01 | 1 | 1 | 1 | 1 |
| 72 | Chlordane | 57-74-9 | 3.20E+01 | 1 | 1 | 1 | 1 |
| 73 | DDT | 50-29-3 | 3.40E+01 | 1 | 1 | 1 | 1 |
| 74 | Dieldrin | 60-57-1 | 6.10E-01 | 1 | 1 | 1 | 1 |
| 75 | Endosulfan | 115-29-7 | 7.30E+02 | 2 | 2 | 1 | 1 |
| 76 | Endrin | 72-20-8 | 3.70E+01 | 1 | 1 | 1 | 1 |
| 77 | Heptachlor | 76-44-8 | 2.20E+00 | 1 | 1 | 1 | 1 |
| 78 | Lindane | 58-89-9 | 1.00E+01 | 2 | 2 | 1 | 1 |
| 79 | Toxaphene | 8001-35-2 | 8.80E+00 | 1 | 1 | 1 | 1 |
| 80 | 2,4-D | 94-75-7 | 1.40E+03 | 1 | 1 | 1 | 1 |
| 82 | Carbaryl | 63-25-2 | 1.20E+04 | 1 | 2 | 1 | 1 |
| 83 | Carbofuran | 1563-66-2 | 6.10E+02 | 1 | 2 | 1 | 1 |
| 84 | Chloryrifos | 2921-88-2 | 1.20E+02 | 0 | 0 | 1 | 1 |
| 85 | Diuron | 330-54-1 | 2.40E+02 | 1 | 1 | 1 | 1 |
| 87 | Malathion | 121-75-5 | 2.40E+03 | 1 | 1 | 1 | 1 |
| 91 | Trifluralin | 1582-09-8 | 9.20E+02 | 1 | 1 | 1 | 1 |
| 92 | Total PCB | 1336-36-3 | 4.40E+00 | 1 | 1 | 1 | 1 |
| 94 | Total PCDD/PCDF | 1746-01-6 | 9.00E-05 | 0 | 0 | 1 | -1 |
| 95 | Methyl ethyl ketone | 78-93-3 | 4.80E+04 | 1 | 2 | 1 | 1 |
| 96 | Methyl isobutyl ketone | 108-10-1 | 1.10E+04 | 1 | 1 | 1 | 1 |
| 97 | Dibutyl phthalate | 84-74-2 | 1.20E+04 | 0 | 1 | 1 | 1 |
| 98 | DEHP | 117-81-7 | 6.90E+02 | 1 | 1 | 1 | 1 |
| 99 | Hexachlorobutadine | 87-68-3 | 1.20E+02 | 2 | 2 | 1 | 1 |
| 100 | Methyl tert-butyl ether | 1634-04-4 | 1.10E+03 | 1 | 2 | 1 | 1 |
|  |  | Overall | C1 | C2 | C3 | C4 | C5 |
|  |  | Scores | 87 | 1.33 | 1.39 | 81 | 79 |
|  |  |  |  |  | No. >0 | 84 | 83 |
|  |  |  |  |  | No. <0 | 3 | 4 |

**Table S33a** Oregon Dept. of Environmental Quality - Residential

| Number | Pollutant | CAS. No | RGV (mg/kg) | C_2_ | C_3_ | C_4_ | C_5_ |
| --- | --- | --- | --- | --- | --- | --- | --- |
| 2 | Arsenic | 7440-38-2 | 4.30E-01 | -1 | -1 | -1 | -1 |
| 3 | Barium | 7440-39-3 | 1.50E+04 | 1 | 1 | 1 | 1 |
| 4 | Beryllium | 7440-41-7 | 1.60E+02 | 1 | 1 | 1 | 1 |
| 5 | Cadmium | 7440-43-9 | 7.80E+01 | 1 | 1 | 1 | 1 |
| 6 | Chromium (III) | 16065-83-1 | 1.20E+05 | 3 | 2 | 1 | 1 |
| 7 | Chromium (VI) | 18540-29-9 | 3.00E-01 | -2 | -2 | -1 | -1 |
| 9 | Copper | 7440-50-8 | 3.10E+03 | 1 | 1 | 1 | 1 |
| 10 | Lead | 7439-92-1 | 4.00E+02 | 0 | 1 | 1 | 1 |
| 11 | Manganese | 7439-96-5 | 1.80E+03 | 0 | 0 | 1 | 1 |
| 12 | Mercury | 7439-97-6 | 2.30E+01 | 1 | 1 | 1 | 1 |
| 14 | Nickel | 7440-02-0 | 1.50E+03 | 1 | 1 | 1 | 1 |
| 16 | Silver | 7440-22-4 | 3.90E+02 | 1 | 1 | 1 | 1 |
| 21 | Cyanide | 57-12-5 | 4.70E+01 | 0 | 0 | -1 | 1 |
| 22 | Acrylonitril | 107-13-1 | 8.60E-01 | 0 | 0 | -1 | -1 |
| 23 | Dichloromethane | 75-09-2 | 7.60E+01 | 1 | 1 | 1 | 1 |
| 24 | Trichloromethane | 67-66-3 | 5.80E+00 | 1 | 1 | 1 | 1 |
| 25 | Tetrachloromethane | 56-23-5 | 7.50E+00 | 1 | 1 | 1 | 1 |
| 26 | Bromoform | 75-25-2 | 5.70E+01 | 0 | 0 | -1 | 1 |
| 27 | Bromomethane | 74-83-9 | 4.60E+01 | 1 | 1 | 1 | 1 |
| 29 | 1,1,1-Trichloroethane | 71-55-6 | 5.30E+04 | 2 | 3 | 1 | 1 |
| 30 | 1,1,2-Trichloroethane | 79-00-5 | 3.20E+00 | 0 | 0 | -1 | 1 |
| 31 | Chloroethene | 75-01-4 | 3.60E-01 | 1 | 0 | 1 | 1 |
| 32 | 1,1-Dichloroethene | 75-35-4 | 1.80E+03 | 2 | 2 | 1 | 1 |
| 33 | Trichloroethene | 79-01-6 | 6.70E+00 | 0 | 1 | 1 | 1 |
| 34 | Tetrachloroethene | 127-18-4 | 2.20E+02 | 2 | 2 | 1 | 1 |
| 35 | Benzene | 71-43-2 | 8.20E+00 | 1 | 1 | 1 | 1 |
| 36 | Toluene | 108-88-3 | 5.80E+03 | 2 | 2 | 1 | 1 |
| 37 | Chlorobenzene | 108-90-7 | 5.30E+02 | 1 | 2 | 1 | 1 |
| 38 | 1,2-Dichlorobenzene | 95-50-1 | 2.20E+03 | 1 | 2 | 1 | 1 |
| 40 | 1,4-Dichlorobenzene | 106-46-7 | 1.40E+01 | 1 | 0 | 1 | 1 |
| 41 | 1,2,4Trichlorobenzene | 120-82-1 | 1.10E+02 | 1 | 1 | 1 | 1 |
| 42 | Hexachlorobenzene | 118-74-1 | 2.10E-01 | 0 | 0 | -1 | -1 |
| 44 | Ethylbenzene | 100-41-4 | 3.40E+01 | 0 | 0 | 1 | 1 |
| 45 | Styrene | 100-42-5 | 7.90E+03 | 2 | 2 | 1 | 1 |
| 46 | Xylenes | 1330-20-7 | 1.40E+03 | 1 | 1 | 1 | 1 |
| 52 | 2,4,6-Trichlorophenol | 88-06-2 | 4.90E+01 | 1 | 1 | 1 | 1 |
| 54 | Pentachlorophenol | 87-86-5 | 1.00E+00 | 0 | 0 | -1 | -1 |
| 55 | Benz(a)anthracene | 56-55-3 | 1.50E-01 | -1 | -1 | -1 | -1 |
| 56 | Benzo(a)pyrene | 50-32-8 | 1.50E-02 | -1 | -1 | -1 | -1 |
| 57 | Benzo(b)fluoranthene | 205-99-2 | 1.50E-01 | -1 | -1 | -1 | -1 |
| 58 | Benzo(k)fluoranthene | 207-08-9 | 1.50E+00 | -1 | 0 | -1 | -1 |
| 59 | Chrysene | 218-01-9 | 1.50E+01 | 0 | 0 | -1 | -1 |
| 60 | Dibena(a,h)anthracene | 53-70-3 | 1.50E-02 | -1 | -1 | -1 | -1 |
| 61 | Indeno(1,2,3-c,d)pyrebe | 193-39-5 | 1.50E-01 | -1 | -1 | -1 | -1 |
| 62 | Naphthalene | 91-20-3 | 5.30E+00 | 0 | 0 | -1 | 1 |
| 63 | Acenaphehene | 83-32-9 | 4.70E+03 | 2 | 2 | 1 | 1 |
| 65 | Anthracene | 120-12-7 | 2.30E+04 | 3 | 2 | 1 | 1 |
| 67 | Flouranthene | 206-44-0 | 2.40E+03 | 3 | 2 | 1 | 1 |
| 68 | Flouorene | 86-73-7 | 3.10E+03 | 2 | 2 | 1 | 1 |
| 70 | Pyrene | 129-00-0 | 1.80E+03 | 1 | 1 | 1 | 1 |
| 71 | Aldrin | 309-00-2 | 3.00E-02 | 0 | -1 | -1 | -1 |
| 72 | Chlordane | 57-74-9 | 1.70E+00 | 0 | 0 | -1 | -1 |
| 73 | DDT | 50-29-3 | 1.90E+00 | 0 | 0 | -1 | -1 |
| 74 | Dieldrin | 60-57-1 | 3.40E-02 | 0 | -1 | -1 | -1 |
| 75 | Endosulfan | 115-29-7 | 3.80E+02 | 1 | 2 | 1 | 1 |
| 76 | Endrin | 72-20-8 | 1.90E+01 | 1 | 1 | 1 | 1 |
| 77 | Heptachlor | 76-44-8 | 1.10E-01 | 0 | 0 | -1 | -1 |
| 78 | Lindane | 58-89-9 | 4.90E-01 | 0 | 0 | -1 | -1 |
| 79 | Toxaphene | 8001-35-2 | 4.90E-01 | 0 | 0 | -1 | -1 |
| 80 | 2,4-D | 94-75-7 | 6.30E+02 | 0 | 1 | -1 | 1 |
| 88 | MCPA | 94-74-6 | 3.20E+01 | 0 | 0 | 1 | 1 |
| 92 | Total PCB | 1336-36-3 | 2.30E-01 | 0 | 0 | -1 | -1 |
| 94 | Total PCDD/PCDF | 1746-01-6 | 4.70E-06 | -1 | -1 | -1 | -1 |
| 98 | DEHP | 117-81-7 | 3.90E+01 | 0 | 0 | -1 | -1 |
| 100 | Methyl tert-butyl ether | 1634-04-4 | 2.50E+02 | 1 | 1 | 1 | 1 |
|  |  | Overall | C1 | C2 | C3 | C4 | C5 |
|  |  | Scores | 65 | 0.55 | 0.57 | 11 | 21 |
|  |  |  |  |  | No. >0 | 38 | 43 |
|  |  |  |  |  | No. <0 | 27 | 22 |

**Table S33b** Oregon - Urban Residential

| Number | Pollutant | CAS. No | RGV (mg/kg) | C_2_ | C_3_ | C_4_ | C_5_ |
| --- | --- | --- | --- | --- | --- | --- | --- |
| 2 | Arsenic | 7440-38-2 | 1.00E+00 | -1 | -1 | -1 | -1 |
| 3 | Barium | 7440-39-3 | 3.10E+04 | 2 | 2 | 1 | 1 |
| 4 | Beryllium | 7440-41-7 | 3.10E+02 | 1 | 1 | 1 | 1 |
| 5 | Cadmium | 7440-43-9 | 1.60E+02 | 2 | 2 | 1 | 1 |
| 6 | Chromium (III) | 16065-83-1 | 2.30E+05 | 3 | 3 | 1 | 1 |
| 7 | Chromium (VI) | 18540-29-9 | 6.70E-01 | -2 | -2 | -1 | -1 |
| 9 | Copper | 7440-50-8 | 6.20E+03 | 2 | 2 | 1 | 1 |
| 10 | Lead | 7439-92-1 | 4.00E+02 | 0 | 1 | 1 | 1 |
| 11 | Manganese | 7439-96-5 | 3.60E+03 | 1 | 1 | 1 | 1 |
| 12 | Mercury | 7439-97-6 | 4.70E+01 | 1 | 1 | 1 | 1 |
| 14 | Nickel | 7440-02-0 | 3.10E+03 | 2 | 2 | 1 | 1 |
| 16 | Silver | 7440-22-4 | 7.80E+02 | 1 | 1 | 1 | 1 |
| 21 | Cyanide | 57-12-5 | 9.40E+01 | 1 | 1 | 1 | 1 |
| 22 | Acrylonitril | 107-13-1 | 2.50E+00 | 0 | 0 | 1 | 1 |
| 23 | Dichloromethane | 75-09-2 | 1.70E+02 | 1 | 1 | 1 | 1 |
| 24 | Trichloromethane | 67-66-3 | 2.20E+01 | 2 | 1 | 1 | 1 |
| 25 | Tetrachloromethane | 56-23-5 | 2.10E+01 | 2 | 2 | 1 | 1 |
| 26 | Bromoform | 75-25-2 | 1.70E+02 | 1 | 1 | 1 | 1 |
| 27 | Bromomethane | 74-83-9 | 9.20E+01 | 1 | 1 | 1 | 1 |
| 29 | 1,1,1-Trichloroethane | 71-55-6 | 1.10E+05 | 3 | 3 | 1 | 1 |
| 30 | 1,1,2-Trichloroethane | 79-00-5 | 6.30E+00 | 0 | 1 | 1 | 1 |
| 31 | Chloroethene | 75-01-4 | 8.00E-01 | 1 | 1 | 1 | 1 |
| 32 | 1,1-Dichloroethene | 75-35-4 | 3.50E+03 | 3 | 3 | 1 | 1 |
| 33 | Trichloroethene | 79-01-6 | 1.70E+01 | 1 | 1 | 1 | 1 |
| 34 | Tetrachloroethene | 127-18-4 | 5.40E+02 | 2 | 2 | 1 | 1 |
| 35 | Benzene | 71-43-2 | 2.40E+01 | 2 | 2 | 1 | 1 |
| 36 | Toluene | 108-88-3 | 1.20E+04 | 2 | 2 | 1 | 1 |
| 37 | Chlorobenzene | 108-90-7 | 1.10E+03 | 1 | 2 | 1 | 1 |
| 38 | 1,2-Dichlorobenzene | 95-50-1 | 4.40E+03 | 2 | 2 | 1 | 1 |
| 40 | 1,4-Dichlorobenzene | 106-46-7 | 6.20E+01 | 1 | 1 | 1 | 1 |
| 41 | 1,2,4Trichlorobenzene | 120-82-1 | 2.20E+02 | 1 | 1 | 1 | 1 |
| 42 | Hexachlorobenzene | 118-74-1 | 6.70E-01 | 0 | 0 | -1 | -1 |
| 44 | Ethylbenzene | 100-41-4 | 1.10E+02 | 1 | 1 | 1 | 1 |
| 45 | Styrene | 100-42-5 | 1.60E+04 | 2 | 3 | 1 | 1 |
| 46 | Xylenes | 1330-20-7 | 2.90E+03 | 1 | 2 | 1 | 1 |
| 52 | 2,4,6-Trichlorophenol | 88-06-2 | 1.20E+02 | 1 | 1 | 1 | 1 |
| 54 | Pentachlorophenol | 87-86-5 | 2.60E+00 | 0 | 0 | -1 | -1 |
| 55 | Benz(a)anthracene | 56-55-3 | 3.40E-01 | -1 | -1 | -1 | -1 |
| 56 | Benzo(a)pyrene | 50-32-8 | 3.40E-02 | -1 | -1 | -1 | -1 |
| 57 | Benzo(b)fluoranthene | 205-99-2 | 3.40E-01 | -1 | -1 | -1 | -1 |
| 58 | Benzo(k)fluoranthene | 207-08-9 | 3.40E+00 | 0 | 0 | -1 | -1 |
| 59 | Chrysene | 218-01-9 | 3.40E+01 | 0 | 0 | -1 | 1 |
| 60 | Dibena(a,h)anthracene | 53-70-3 | 3.40E-02 | -1 | -1 | -1 | -1 |
| 61 | Indeno(1,2,3-c,d)pyrebe | 193-39-5 | 3.40E-01 | -1 | -1 | -1 | -1 |
| 62 | Naphthalene | 91-20-3 | 2.50E+01 | 0 | 1 | 1 | 1 |
| 63 | Acenaphehene | 83-32-9 | 9.40E+03 | 2 | 2 | 1 | 1 |
| 65 | Anthracene | 120-12-7 | 4.70E+04 | 3 | 3 | 1 | 1 |
| 67 | Flouranthene | 206-44-0 | 4.80E+03 | 3 | 2 | 1 | 1 |
| 68 | Flouorene | 86-73-7 | 6.30E+03 | 2 | 2 | 1 | 1 |
| 70 | Pyrene | 129-00-0 | 3.60E+03 | 2 | 2 | 1 | 1 |
| 71 | Aldrin | 309-00-2 | 8.00E-02 | 0 | 0 | -1 | -1 |
| 72 | Chlordane | 57-74-9 | 4.20E+00 | 0 | 0 | 1 | 1 |
| 73 | DDT | 50-29-3 | 4.60E+00 | 1 | 0 | 1 | 1 |
| 74 | Dieldrin | 60-57-1 | 8.50E-02 | 0 | 0 | -1 | -1 |
| 75 | Endosulfan | 115-29-7 | 7.60E+02 | 2 | 2 | 1 | 1 |
| 76 | Endrin | 72-20-8 | 3.80E+01 | 1 | 1 | 1 | 1 |
| 77 | Heptachlor | 76-44-8 | 2.80E-01 | 0 | 0 | 1 | -1 |
| 78 | Lindane | 58-89-9 | 1.20E+00 | 1 | 1 | 1 | 1 |
| 79 | Toxaphene | 8001-35-2 | 1.20E+00 | 1 | 0 | 1 | -1 |
| 80 | 2,4-D | 94-75-7 | 1.30E+03 | 1 | 1 | 1 | 1 |
| 88 | MCPA | 94-74-6 | 6.30E+01 | 1 | 1 | 1 | 1 |
| 92 | Total PCB | 1336-36-3 | 3.30E-01 | 0 | 0 | -1 | -1 |
| 94 | Total PCDD/PCDF | 1746-01-6 | 1.20E-05 | 0 | -1 | -1 | -1 |
| 98 | DEHP | 117-81-7 | 9.70E+01 | 0 | 0 | 1 | -1 |
| 100 | Methyl tert-butyl ether | 1634-04-4 | 7.30E+02 | 1 | 2 | 1 | 1 |
|  |  | Overall | C1 | C2 | C3 | C4 | C5 |
|  |  | Scores | 65 | 0.92 | 0.95 | 35 | 31 |
|  |  |  |  |  | No. >0 | 50 | 48 |
|  |  |  |  |  | No. <0 | 15 | 17 |

**Table S33c** Oregon - Hot Spot Concentration(HSC) Residential

| Number | Pollutant | CAS. No | RGV (mg/kg) | C_2_ | C_3_ | C_4_ | C_5_ |
| --- | --- | --- | --- | --- | --- | --- | --- |
| 2 | Arsenic | 7440-38-2 | 4.30E+01 | 1 | 1 | 1 | 1 |
| 3 | Barium | 7440-39-3 | 1.50E+05 | 2 | 2 | 1 | 1 |
| 4 | Beryllium | 7440-41-7 | 1.60E+03 | 2 | 2 | 1 | 1 |
| 5 | Cadmium | 7440-43-9 | 7.80E+02 | 2 | 2 | 1 | 1 |
| 7 | Chromium (VI) | 18540-29-9 | 3.00E+01 | 0 | 0 | -1 | -1 |
| 9 | Copper | 7440-50-8 | 3.10E+04 | 2 | 2 | 1 | 1 |
| 10 | Lead | 7439-92-1 | 4.00E+03 | 1 | 2 | 1 | 1 |
| 11 | Manganese | 7439-96-5 | 1.80E+04 | 1 | 1 | 1 | 1 |
| 12 | Mercury | 7439-97-6 | 2.30E+02 | 2 | 2 | 1 | 1 |
| 14 | Nickel | 7440-02-0 | 1.50E+04 | 2 | 2 | 1 | 1 |
| 16 | Silver | 7440-22-4 | 3.90E+03 | 2 | 2 | 1 | 1 |
| 21 | Cyanide | 57-12-5 | 4.70E+02 | 1 | 1 | 1 | 1 |
| 22 | Acrylonitril | 107-13-1 | 8.60E+01 | 2 | 2 | 1 | 1 |
| 23 | Dichloromethane | 75-09-2 | 4.50E+03 | 3 | 3 | 1 | 1 |
| 24 | Trichloromethane | 67-66-3 | 5.80E+02 | 3 | 3 | 1 | 1 |
| 25 | Tetrachloromethane | 56-23-5 | 7.50E+02 | 3 | 3 | 1 | 1 |
| 26 | Bromoform | 75-25-2 | 5.70E+03 | 2 | 2 | 1 | 1 |
| 27 | Bromomethane | 74-83-9 | 4.60E+02 | 2 | 2 | 1 | 1 |
| 29 | 1,1,1-Trichloroethane | 71-55-6 | 5.30E+05 | 3 | 4 | 1 | 1 |
| 30 | 1,1,2-Trichloroethane | 79-00-5 | 3.20E+01 | 1 | 1 | 1 | 1 |
| 31 | Chloroethene | 75-01-4 | 3.60E+01 | 3 | 2 | 1 | 1 |
| 32 | 1,1-Dichloroethene | 75-35-4 | 1.80E+04 | 3 | 3 | 1 | 1 |
| 33 | Trichloroethene | 79-01-6 | 1.80E+02 | 2 | 2 | 1 | 1 |
| 34 | Tetrachloroethene | 127-18-4 | 2.70E+03 | 3 | 3 | 1 | 1 |
| 35 | Benzene | 71-43-2 | 8.20E+02 | 3 | 3 | 1 | 1 |
| 36 | Toluene | 108-88-3 | 5.80E+04 | 3 | 3 | 1 | 1 |
| 37 | Chlorobenzene | 108-90-7 | 5.30E+03 | 2 | 3 | 1 | 1 |
| 38 | 1,2-Dichlorobenzene | 95-50-1 | 2.20E+04 | 2 | 3 | 1 | 1 |
| 40 | 1,4-Dichlorobenzene | 106-46-7 | 1.40E+03 | 3 | 2 | 1 | 1 |
| 41 | 1,2,4Trichlorobenzene | 120-82-1 | 1.10E+03 | 2 | 2 | 1 | 1 |
| 42 | Hexachlorobenzene | 118-74-1 | 2.10E+01 | 2 | 2 | 1 | 1 |
| 44 | Ethylbenzene | 100-41-4 | 3.40E+03 | 2 | 2 | 1 | 1 |
| 45 | Styrene | 100-42-5 | 7.90E+04 | 3 | 3 | 1 | 1 |
| 46 | Xylenes | 1330-20-7 | 1.40E+04 | 2 | 2 | 1 | 1 |
| 52 | 2,4,6-Trichlorophenol | 88-06-2 | 6.30E+02 | 2 | 2 | 1 | 1 |
| 54 | Pentachlorophenol | 87-86-5 | 1.00E+02 | 2 | 2 | 1 | 1 |
| 55 | Benz(a)anthracene | 56-55-3 | 1.50E+01 | 1 | 1 | 1 | 1 |
| 56 | Benzo(a)pyrene | 50-32-8 | 1.50E+00 | 1 | 1 | 1 | 1 |
| 57 | Benzo(b)fluoranthene | 205-99-2 | 1.50E+01 | 1 | 1 | 1 | 1 |
| 58 | Benzo(k)fluoranthene | 207-08-9 | 1.50E+02 | 1 | 2 | 1 | 1 |
| 59 | Chrysene | 218-01-9 | 1.50E+03 | 2 | 2 | 1 | 1 |
| 60 | Dibena(a,h)anthracene | 53-70-3 | 1.50E+00 | 1 | 1 | 1 | 1 |
| 61 | Indeno(1,2,3-c,d)pyrebe | 193-39-5 | 1.50E+01 | 1 | 1 | 1 | 1 |
| 62 | Naphthalene | 91-20-3 | 5.30E+02 | 2 | 2 | 1 | 1 |
| 63 | Acenaphehene | 83-32-9 | 4.70E+04 | 3 | 3 | 1 | 1 |
| 65 | Anthracene | 120-12-7 | 2.30E+05 | 4 | 3 | 1 | 1 |
| 67 | Flouranthene | 206-44-0 | 2.40E+04 | 4 | 3 | 1 | 1 |
| 68 | Flouorene | 86-73-7 | 3.10E+04 | 3 | 3 | 1 | 1 |
| 70 | Pyrene | 129-00-0 | 1.80E+04 | 2 | 2 | 1 | 1 |
| 71 | Aldrin | 309-00-2 | 3.10E+00 | 2 | 1 | 1 | 1 |
| 72 | Chlordane | 57-74-9 | 1.70E+02 | 2 | 2 | 1 | 1 |
| 73 | DDT | 50-29-3 | 1.90E+02 | 2 | 2 | 1 | 1 |
| 74 | Dieldrin | 60-57-1 | 3.40E+00 | 2 | 1 | 1 | 1 |
| 75 | Endosulfan | 115-29-7 | 3.80E+03 | 2 | 3 | 1 | 1 |
| 76 | Endrin | 72-20-8 | 1.90E+02 | 2 | 2 | 1 | 1 |
| 77 | Heptachlor | 76-44-8 | 1.10E+01 | 2 | 2 | 1 | 1 |
| 78 | Lindane | 58-89-9 | 4.90E+01 | 2 | 2 | 1 | 1 |
| 79 | Toxaphene | 8001-35-2 | 4.90E+01 | 2 | 2 | 1 | 1 |
| 80 | 2,4-D | 94-75-7 | 6.30E+03 | 1 | 2 | 1 | 1 |
| 88 | MCPA | 94-74-6 | 3.20E+02 | 1 | 1 | 1 | 1 |
| 92 | Total PCB | 1336-36-3 | 3.30E+00 | 1 | 1 | 1 | 1 |
| 94 | Total PCDD/PCDF | 1746-01-6 | 4.70E-04 | 1 | 1 | 1 | 1 |
| 98 | DEHP | 117-81-7 | 3.90E+03 | 2 | 2 | 1 | 1 |
| 100 | Methyl tert-butyl ether | 1634-04-4 | 2.50E+04 | 3 | 3 | 1 | 1 |
|  |  | Overall | C1 | C2 | C3 | C4 | C5 |
|  |  | Scores | 64 | 2.02 | 2.03 | 62 | 62 |
|  |  |  |  |  | No. >0 | 63 | 63 |
|  |  |  |  |  | No. <0 | 1 | 1 |

**Table S33d** Oregon - HSC Urban Residential

| Number | Pollutant | CAS. No | RGV (mg/kg) | C_2_ | C_3_ | C_4_ | C_5_ |
| --- | --- | --- | --- | --- | --- | --- | --- |
| 2 | Arsenic | 7440-38-2 | 1.00E+02 | 1 | 1 | 1 | 1 |
| 3 | Barium | 7440-39-3 | 3.10E+05 | 3 | 3 | 1 | 1 |
| 4 | Beryllium | 7440-41-7 | 3.10E+03 | 2 | 2 | 1 | 1 |
| 5 | Cadmium | 7440-43-9 | 1.60E+03 | 3 | 3 | 1 | 1 |
| 7 | Chromium (VI) | 18540-29-9 | 6.70E+01 | 0 | 0 | -1 | 1 |
| 9 | Copper | 7440-50-8 | 6.20E+04 | 3 | 3 | 1 | 1 |
| 10 | Lead | 7439-92-1 | 4.00E+03 | 1 | 2 | 1 | 1 |
| 11 | Manganese | 7439-96-5 | 3.60E+04 | 2 | 2 | 1 | 1 |
| 12 | Mercury | 7439-97-6 | 4.70E+02 | 2 | 2 | 1 | 1 |
| 14 | Nickel | 7440-02-0 | 3.10E+04 | 3 | 3 | 1 | 1 |
| 16 | Silver | 7440-22-4 | 7.80E+03 | 2 | 2 | 1 | 1 |
| 21 | Cyanide | 57-12-5 | 9.40E+02 | 2 | 2 | 1 | 1 |
| 22 | Acrylonitril | 107-13-1 | 2.50E+02 | 2 | 2 | 1 | 1 |
| 23 | Dichloromethane | 75-09-2 | 8.90E+03 | 3 | 3 | 1 | 1 |
| 24 | Trichloromethane | 67-66-3 | 2.20E+03 | 4 | 3 | 1 | 1 |
| 25 | Tetrachloromethane | 56-23-5 | 2.10E+03 | 4 | 4 | 1 | 1 |
| 26 | Bromoform | 75-25-2 | 1.70E+04 | 3 | 3 | 1 | 1 |
| 27 | Bromomethane | 74-83-9 | 9.20E+02 | 2 | 2 | 1 | 1 |
| 30 | 1,1,2-Trichloroethane | 79-00-5 | 6.30E+01 | 1 | 2 | 1 | 1 |
| 31 | Chloroethene | 75-01-4 | 8.00E+01 | 3 | 3 | 1 | 1 |
| 32 | 1,1-Dichloroethene | 75-35-4 | 3.50E+04 | 4 | 4 | 1 | 1 |
| 33 | Trichloroethene | 79-01-6 | 3.50E+02 | 2 | 2 | 1 | 1 |
| 34 | Tetrachloroethene | 127-18-4 | 5.40E+03 | 3 | 3 | 1 | 1 |
| 35 | Benzene | 71-43-2 | 2.40E+03 | 4 | 4 | 1 | 1 |
| 36 | Toluene | 108-88-3 | 1.20E+05 | 3 | 3 | 1 | 1 |
| 37 | Chlorobenzene | 108-90-7 | 1.10E+04 | 2 | 3 | 1 | 1 |
| 38 | 1,2-Dichlorobenzene | 95-50-1 | 4.40E+04 | 3 | 3 | 1 | 1 |
| 40 | 1,4-Dichlorobenzene | 106-46-7 | 6.20E+03 | 3 | 3 | 1 | 1 |
| 41 | 1,2,4Trichlorobenzene | 120-82-1 | 2.20E+03 | 2 | 2 | 1 | 1 |
| 42 | Hexachlorobenzene | 118-74-1 | 6.70E+01 | 2 | 2 | 1 | 1 |
| 44 | Ethylbenzene | 100-41-4 | 1.10E+04 | 3 | 3 | 1 | 1 |
| 45 | Styrene | 100-42-5 | 1.60E+05 | 3 | 4 | 1 | 1 |
| 46 | Xylenes | 1330-20-7 | 2.90E+04 | 2 | 3 | 1 | 1 |
| 52 | 2,4,6-Trichlorophenol | 88-06-2 | 1.30E+03 | 2 | 2 | 1 | 1 |
| 54 | Pentachlorophenol | 87-86-5 | 2.60E+02 | 2 | 2 | 1 | 1 |
| 55 | Benz(a)anthracene | 56-55-3 | 3.40E+01 | 1 | 1 | 1 | 1 |
| 56 | Benzo(a)pyrene | 50-32-8 | 3.40E+00 | 1 | 1 | 1 | 1 |
| 57 | Benzo(b)fluoranthene | 205-99-2 | 3.40E+01 | 1 | 1 | 1 | 1 |
| 58 | Benzo(k)fluoranthene | 207-08-9 | 3.40E+02 | 2 | 2 | 1 | 1 |
| 59 | Chrysene | 218-01-9 | 3.40E+03 | 2 | 2 | 1 | 1 |
| 60 | Dibena(a,h)anthracene | 53-70-3 | 3.40E+00 | 1 | 1 | 1 | 1 |
| 61 | Indeno(1,2,3-c,d)pyrebe | 193-39-5 | 3.40E+01 | 1 | 1 | 1 | 1 |
| 62 | Naphthalene | 91-20-3 | 1.20E+03 | 2 | 3 | 1 | 1 |
| 63 | Acenaphehene | 83-32-9 | 9.40E+04 | 3 | 3 | 1 | 1 |
| 65 | Anthracene | 120-12-7 | 4.70E+05 | 4 | 4 | 1 | 1 |
| 67 | Flouranthene | 206-44-0 | 4.80E+04 | 4 | 3 | 1 | 1 |
| 68 | Flouorene | 86-73-7 | 6.30E+04 | 3 | 3 | 1 | 1 |
| 70 | Pyrene | 129-00-0 | 3.60E+04 | 3 | 3 | 1 | 1 |
| 71 | Aldrin | 309-00-2 | 7.80E+00 | 2 | 2 | 1 | 1 |
| 72 | Chlordane | 57-74-9 | 4.20E+02 | 2 | 2 | 1 | 1 |
| 73 | DDT | 50-29-3 | 4.60E+02 | 3 | 2 | 1 | 1 |
| 74 | Dieldrin | 60-57-1 | 8.50E+00 | 2 | 2 | 1 | 1 |
| 75 | Endosulfan | 115-29-7 | 7.60E+03 | 3 | 3 | 1 | 1 |
| 76 | Endrin | 72-20-8 | 3.80E+02 | 2 | 2 | 1 | 1 |
| 77 | Heptachlor | 76-44-8 | 2.80E+01 | 2 | 2 | 1 | 1 |
| 78 | Lindane | 58-89-9 | 1.20E+02 | 3 | 3 | 1 | 1 |
| 79 | Toxaphene | 8001-35-2 | 1.20E+02 | 3 | 2 | 1 | 1 |
| 80 | 2,4-D | 94-75-7 | 1.30E+04 | 2 | 2 | 1 | 1 |
| 88 | MCPA | 94-74-6 | 6.30E+02 | 2 | 2 | 1 | 1 |
| 92 | Total PCB | 1336-36-3 | 3.30E+00 | 1 | 1 | 1 | 1 |
| 94 | Total PCDD/PCDF | 1746-01-6 | 1.00E-03 | 1 | 1 | 1 | 1 |
| 98 | DEHP | 117-81-7 | 9.70E+03 | 2 | 2 | 1 | 1 |
| 100 | Methyl tert-butyl ether | 1634-04-4 | 7.30E+04 | 3 | 4 | 1 | 1 |
|  |  | Overall | C1 | C2 | C3 | C4 | C5 |
|  |  | Scores | 63 | 2.33 | 2.38 | 61 | 63 |
|  |  |  |  |  | No. >0 | 62 | 63 |
|  |  |  |  |  | No. <0 | 1 | 0 |

**Table S33e** Oregon - Catch Basin Screening

| Number | Pollutant | CAS. No | RGV (mg/kg) | C_2_ | C_3_ | C_4_ | C_5_ |
| --- | --- | --- | --- | --- | --- | --- | --- |
| 1 | Antimony | 7440-36-0 | 6.40E+01 | 1 | 1 | 1 | 1 |
| 2 | Arsenic | 7440-38-2 | 7.00E+00 | 0 | 0 | -1 | -1 |
| 5 | Cadmium | 7440-43-9 | 1.00E+00 | -1 | -1 | -1 | -1 |
| 6 | Chromium (III) | 16065-83-1 | 1.11E+02 | 0 | -1 | -1 | -1 |
| 7 | Chromium (VI) | 18540-29-9 | 1.11E+02 | 0 | 1 | 1 | 1 |
| 9 | Copper | 7440-50-8 | 1.49E+02 | 0 | 0 | -1 | -1 |
| 10 | Lead | 7439-92-1 | 1.70E+01 | -1 | -1 | -1 | -1 |
| 11 | Manganese | 7439-96-5 | 1.10E+03 | 0 | 0 | -1 | -1 |
| 12 | Mercury | 7439-97-6 | 7.00E-02 | -1 | -1 | -1 | -1 |
| 14 | Nickel | 7440-02-0 | 4.86E+01 | 0 | 0 | -1 | -1 |
| 15 | Selenium | 7782-49-2 | 2.00E+00 | -1 | -1 | -1 | -1 |
| 16 | Silver | 7440-22-4 | 5.00E+00 | -1 | -1 | -1 | -1 |
| 20 | Zinc | 7440-66-6 | 4.59E+02 | 0 | 0 | -1 | -1 |
| 33 | Trichloroethene | 79-01-6 | 2.10E+00 | 0 | 0 | -1 | -1 |
| 34 | Tetrachloroethene | 127-18-4 | 5.00E-01 | -1 | -1 | -1 | -1 |
| 38 | 1,2-Dichlorobenzene | 95-50-1 | 1.70E+00 | -2 | -1 | -1 | -1 |
| 39 | 1,3-Dichlorobenzene | 541-73-1 | 3.00E-01 | -2 | -2 | -1 | -1 |
| 40 | 1,4-Dichlorobenzene | 106-46-7 | 3.00E-01 | -1 | -1 | -1 | -1 |
| 41 | 1,2,4Trichlorobenzene | 120-82-1 | 9.20E+00 | 0 | 0 | -1 | -1 |
| 42 | Hexachlorobenzene | 118-74-1 | 1.90E-02 | -1 | -1 | -1 | -1 |
| 48 | Phenol | 108-95-2 | 5.00E-02 | -3 | -3 | -1 | -1 |
| 54 | Pentachlorophenol | 87-86-5 | 2.50E-01 | -1 | -1 | -1 | -1 |
| 55 | Benz(a)anthracene | 56-55-3 | 1.05E+00 | 0 | 0 | -1 | -1 |
| 56 | Benzo(a)pyrene | 50-32-8 | 1.45E+00 | 1 | 1 | 1 | 1 |
| 58 | Benzo(k)fluoranthene | 207-08-9 | 1.30E+01 | 0 | 0 | 1 | 1 |
| 60 | Dibena(a,h)anthracene | 53-70-3 | 1.30E+00 | 1 | 1 | 1 | 1 |
| 61 | Indeno(1,2,3-c,d)pyrebe | 193-39-5 | 1.00E-01 | -1 | -1 | -1 | -1 |
| 62 | Naphthalene | 91-20-3 | 5.61E-01 | -1 | -1 | -1 | -1 |
| 63 | Acenaphehene | 83-32-9 | 3.00E-01 | -2 | -2 | -1 | -1 |
| 64 | Acenaphthylene | 208-96-8 | 2.00E-01 | -2 | -2 | -1 | -1 |
| 65 | Anthracene | 120-12-7 | 8.45E-01 | -2 | -2 | -1 | -1 |
| 66 | Benzo(g,h,i) perylene | 191-24-2 | 3.00E-01 | -2 | -2 | -1 | -1 |
| 67 | Flouranthene | 206-44-0 | 2.23E+00 | 0 | -1 | -1 | -1 |
| 68 | Flouorene | 86-73-7 | 5.36E-01 | -2 | -2 | -1 | -1 |
| 69 | Phenanthrene | 85-01-8 | 1.17E+00 | -1 | -1 | -1 | -1 |
| 70 | Pyrene | 129-00-0 | 1.52E+00 | -2 | -2 | -1 | -1 |
| 71 | Aldrin | 309-00-2 | 4.00E-02 | 0 | -1 | -1 | -1 |
| 72 | Chlordane | 57-74-9 | 3.70E-04 | -4 | -4 | -1 | -1 |
| 73 | DDT | 50-29-3 | 3.30E-04 | -4 | -4 | -1 | -1 |
| 76 | Endrin | 72-20-8 | 2.07E-01 | -1 | -1 | -1 | -1 |
| 77 | Heptachlor | 76-44-8 | 1.00E-02 | -1 | -1 | -1 | -1 |
| 78 | Lindane | 58-89-9 | 4.99E-03 | -2 | -2 | -1 | -1 |
| 92 | Total PCB | 1336-36-3 | 3.90E-04 | -3 | -3 | -1 | -1 |
| 94 | Total PCDD/PCDF | 1746-01-6 | 9.10E-06 | -1 | -1 | -1 | -1 |
| 97 | Dibutyl phthalate | 84-74-2 | 6.00E-02 | 0 | 0 | -1 | -1 |
| 98 | DEHP | 117-81-7 | 3.30E-01 | -2 | -2 | -1 | -1 |
| 99 | Hexachlorobutadine | 87-68-3 | 6.00E-01 | -1 | -1 | -1 | -1 |
|  |  | Overall | C1 | C2 | C3 | C4 | C5 |
|  |  | Scores | 47 | -1 | -1.02 | -37 | -37 |
|  |  |  |  |  | No. >0 | 5 | 5 |
|  |  |  |  |  | No. <0 | 42 | 42 |

**Table S34** Pennsylvania Code

| Number | Pollutant | CAS. No | RGV (mg/kg) | C_2_ | C_3_ | C_4_ | C_5_ |
| --- | --- | --- | --- | --- | --- | --- | --- |
| 1 | Antimony | 7440-36-0 | 8.80E+01 | 1 | 1 | 1 | 1 |
| 2 | Arsenic | 7440-38-2 | 1.20E+01 | 0 | 0 | -1 | 1 |
| 3 | Barium | 7440-39-3 | 4.40E+04 | 2 | 2 | 1 | 1 |
| 4 | Beryllium | 7440-41-7 | 2.00E+00 | -1 | -1 | -1 | -1 |
| 5 | Cadmium | 7440-43-9 | 1.20E+00 | -1 | -1 | -1 | -1 |
| 6 | Chromium (III) | 16065-83-1 | 1.90E+05 | 3 | 3 | 1 | 1 |
| 7 | Chromium (VI) | 18540-29-9 | 4.00E+00 | -1 | -1 | -1 | -1 |
| 8 | Cobalt | 7440-48-4 | 6.60E+01 | 0 | 0 | 1 | -1 |
| 9 | Copper | 7440-50-8 | 8.10E+03 | 2 | 2 | 1 | 1 |
| 10 | Lead | 7439-92-1 | 5.00E+02 | 1 | 1 | 1 | 1 |
| 11 | Manganese | 7439-96-5 | 1.00E+04 | 1 | 1 | 1 | 1 |
| 12 | Mercury | 7439-97-6 | 3.50E+01 | 1 | 1 | 1 | 1 |
| 13 | Molybdenum | 7439-98-7 | 1.10E+03 | 1 | 2 | 1 | 1 |
| 14 | Nickel | 7440-02-0 | 4.40E+03 | 2 | 2 | 1 | 1 |
| 15 | Selenium | 7782-49-2 | 1.10E+03 | 2 | 2 | 1 | 1 |
| 16 | Silver | 7440-22-4 | 1.10E+03 | 2 | 2 | 1 | 1 |
| 17 | Thallium | 7440-28-0 | 2.00E+00 | 0 | 0 | -1 | -1 |
| 18 | Tin | 7440-31-5 | 1.30E+05 | 3 | 3 | 1 | 1 |
| 19 | Vanadium | 7440-62-2 | 1.50E+01 | -1 | -1 | -1 | -1 |
| 20 | Zinc | 7440-66-6 | 6.60E+04 | 2 | 2 | 1 | 1 |
| 21 | Cyanide | 57-12-5 | 1.30E+02 | 1 | 1 | 1 | 1 |
| 22 | Acrylonitril | 107-13-1 | 6.60E+00 | 1 | 1 | 1 | 1 |
| 23 | Dichloromethane | 75-09-2 | 1.30E+03 | 2 | 2 | 1 | 1 |
| 24 | Trichloromethane | 67-66-3 | 1.90E+01 | 1 | 1 | 1 | 1 |
| 25 | Tetrachloromethane | 56-23-5 | 7.40E+01 | 2 | 2 | 1 | 1 |
| 26 | Bromoform | 75-25-2 | 4.10E+02 | 1 | 1 | 1 | 1 |
| 27 | Bromomethane | 74-83-9 | 9.60E+01 | 1 | 1 | 1 | 1 |
| 28 | 1,2-Dichloroethane | 107-06-2 | 1.70E+01 | 1 | 1 | 1 | 1 |
| 29 | 1,1,1-Trichloroethane | 71-55-6 | 1.00E+04 | 2 | 2 | 1 | 1 |
| 30 | 1,1,2-Trichloroethane | 79-00-5 | 4.00E+00 | 0 | 0 | 1 | 1 |
| 31 | Chloroethene | 75-01-4 | 9.00E-01 | 1 | 1 | 1 | 1 |
| 32 | 1,1-Dichloroethene | 75-35-4 | 3.80E+03 | 3 | 3 | 1 | 1 |
| 33 | Trichloroethene | 79-01-6 | 3.80E+01 | 1 | 1 | 1 | 1 |
| 34 | Tetrachloroethene | 127-18-4 | 7.70E+02 | 3 | 3 | 1 | 1 |
| 35 | Benzene | 71-43-2 | 5.70E+01 | 2 | 2 | 1 | 1 |
| 36 | Toluene | 108-88-3 | 1.00E+04 | 2 | 2 | 1 | 1 |
| 37 | Chlorobenzene | 108-90-7 | 9.60E+02 | 1 | 2 | 1 | 1 |
| 38 | 1,2-Dichlorobenzene | 95-50-1 | 3.80E+03 | 2 | 2 | 1 | 1 |
| 39 | 1,3-Dichlorobenzene | 541-73-1 | 1.00E+04 | 3 | 3 | 1 | 1 |
| 41 | 1,2,4Trichlorobenzene | 120-82-1 | 6.40E+02 | 2 | 2 | 1 | 1 |
| 42 | Hexachlorobenzene | 118-74-1 | 1.20E+01 | 1 | 1 | 1 | 1 |
| 43 | Nitrobenzene | 98-95-3 | 4.40E+02 | 2 | 2 | 1 | 1 |
| 44 | Ethylbenzene | 100-41-4 | 1.80E+02 | 1 | 1 | 1 | 1 |
| 45 | Styrene | 100-42-5 | 1.00E+04 | 2 | 2 | 1 | 1 |
| 46 | Xylenes | 1330-20-7 | 1.90E+03 | 1 | 1 | 1 | 1 |
| 47 | Cresol | 1319-77-3 | 1.00E+04 | 2 | 2 | 1 | 1 |
| 48 | Phenol | 108-95-2 | 3.80E+03 | 2 | 1 | 1 | 1 |
| 49 | 2-Chlorophenol | 95-57-8 | 1.10E+03 | 2 | 2 | 1 | 1 |
| 50 | 2,4-Dichlorophenol | 120-83-2 | 6.60E+02 | 2 | 2 | 1 | 1 |
| 51 | 2,4,5-Trichlorophenol | 95-95-4 | 2.20E+04 | 2 | 2 | 1 | 1 |
| 52 | 2,4,6-Trichlorophenol | 88-06-2 | 2.20E+02 | 2 | 1 | 1 | 1 |
| 53 | 2,3,4,6-Tetrahlorophenol | 58-90-2 | 6.60E+03 | 2 | 2 | 1 | 1 |
| 54 | Pentachlorophenol | 87-86-5 | 4.70E+01 | 1 | 1 | 1 | 1 |
| 55 | Benz(a)anthracene | 56-55-3 | 6.00E+00 | 1 | 1 | 1 | 1 |
| 56 | Benzo(a)pyrene | 50-32-8 | 5.80E-01 | 0 | 0 | -1 | -1 |
| 57 | Benzo(b)fluoranthene | 205-99-2 | 3.50E+00 | 0 | 0 | 1 | 1 |
| 58 | Benzo(k)fluoranthene | 207-08-9 | 4.00E+00 | 0 | 0 | -1 | -1 |
| 59 | Chrysene | 218-01-9 | 3.50E+01 | 0 | 0 | 1 | 1 |
| 60 | Dibena(a,h)anthracene | 53-70-3 | 1.00E+00 | 0 | 0 | 1 | 1 |
| 61 | Indeno(1,2,3-c,d)pyrebe | 193-39-5 | 3.50E+00 | 0 | 0 | 1 | 1 |
| 62 | Naphthalene | 91-20-3 | 1.60E+02 | 1 | 2 | 1 | 1 |
| 63 | Acenaphehene | 83-32-9 | 1.30E+04 | 2 | 2 | 1 | 1 |
| 64 | Acenaphthylene | 208-96-8 | 1.30E+04 | 2 | 3 | 1 | 1 |
| 65 | Anthracene | 120-12-7 | 6.60E+04 | 3 | 3 | 1 | 1 |
| 66 | Benzo(g,h,i) perylene | 191-24-2 | 1.30E+04 | 3 | 3 | 1 | 1 |
| 67 | Flouranthene | 206-44-0 | 8.80E+03 | 3 | 2 | 1 | 1 |
| 68 | Flouorene | 86-73-7 | 8.80E+03 | 2 | 2 | 1 | 1 |
| 69 | Phenanthrene | 85-01-8 | 6.60E+04 | 3 | 3 | 1 | 1 |
| 70 | Pyrene | 129-00-0 | 6.60E+03 | 2 | 2 | 1 | 1 |
| 71 | Aldrin | 309-00-2 | 1.10E+00 | 1 | 1 | 1 | 1 |
| 72 | Chlordane | 57-74-9 | 5.30E+01 | 2 | 1 | 1 | 1 |
| 73 | DDT | 50-29-3 | 4.40E+01 | 2 | 1 | 1 | 1 |
| 74 | Dieldrin | 60-57-1 | 1.20E+00 | 1 | 1 | 1 | 1 |
| 75 | Endosulfan | 115-29-7 | 1.30E+03 | 2 | 2 | 1 | 1 |
| 76 | Endrin | 72-20-8 | 6.60E+01 | 1 | 2 | 1 | 1 |
| 77 | Heptachlor | 76-44-8 | 4.00E+00 | 2 | 1 | 1 | 1 |
| 78 | Lindane | 58-89-9 | 1.70E+01 | 2 | 2 | 1 | 1 |
| 79 | Toxaphene | 8001-35-2 | 1.70E+01 | 2 | 1 | 1 | 1 |
| 80 | 2,4-D | 94-75-7 | 2.20E+03 | 1 | 1 | 1 | 1 |
| 81 | Atrazine | 1912-24-9 | 8.10E+01 | 2 | 1 | 1 | 1 |
| 82 | Carbaryl | 63-25-2 | 2.20E+04 | 1 | 2 | 1 | 1 |
| 83 | Carbofuran | 1563-66-2 | 1.10E+03 | 1 | 2 | 1 | 1 |
| 84 | Chloryrifos | 2921-88-2 | 2.20E+02 | 1 | 1 | 1 | 1 |
| 85 | Diuron | 330-54-1 | 4.40E+02 | 1 | 1 | 1 | 1 |
| 86 | Gylphosate | 1071-83-6 | 2.20E+04 | 1 | 2 | 1 | 1 |
| 87 | Malathion | 121-75-5 | 4.40E+03 | 1 | 1 | 1 | 1 |
| 89 | Picloram | 1918-02-1 | 1.50E+04 | 1 | 1 | 1 | 1 |
| 90 | Simazine | 122-34-9 | 1.60E+02 | 2 | 2 | 1 | 1 |
| 93 | PCB 118 | 31508-00-6 | 4.40E+00 | 2 | 1 | 1 | 1 |
| 94 | Total PCDD/PCDF | 1746-01-6 | 1.40E-04 | 1 | 0 | 1 | 1 |
| 95 | Methyl ethyl ketone | 78-93-3 | 1.00E+04 | 0 | 1 | 1 | 1 |
| 96 | Methyl isobutyl ketone | 108-10-1 | 1.00E+04 | 1 | 1 | 1 | 1 |
| 98 | DEHP | 117-81-7 | 1.30E+03 | 1 | 1 | 1 | 1 |
| 99 | Hexachlorobutadine | 87-68-3 | 2.20E+02 | 2 | 2 | 1 | 1 |
| 100 | Methyl tert-butyl ether | 1634-04-4 | 1.70E+03 | 2 | 2 | 1 | 1 |
|  |  | Overall | C1 | C2 | C3 | C4 | C5 |
|  |  | Scores | 95 | 1.38 | 1.38 | 79 | 79 |
|  |  |  |  |  | No. >0 | 87 | 87 |
|  |  |  |  |  | No. <0 | 8 | 8 |

**Table S35** Rhode Island Dept. of Environmental Management

| Number | Pollutant | CAS. No | RGV (mg/kg) | C_2_ | C_3_ | C_4_ | C_5_ |
| --- | --- | --- | --- | --- | --- | --- | --- |
| 1 | Antimony | 7440-36-0 | 1.00E+01 | 0 | 0 | -1 | -1 |
| 2 | Arsenic | 7440-38-2 | 7.00E+00 | 0 | 0 | -1 | -1 |
| 3 | Barium | 7440-39-3 | 5.50E+03 | 1 | 1 | 1 | 1 |
| 4 | Beryllium | 7440-41-7 | 1.50E+00 | -1 | -1 | -1 | -1 |
| 5 | Cadmium | 7440-43-9 | 3.90E+01 | 1 | 1 | 1 | 1 |
| 6 | Chromium (III) | 16065-83-1 | 1.40E+03 | 1 | 0 | 1 | 1 |
| 7 | Chromium (VI) | 18540-29-9 | 3.90E+02 | 1 | 1 | 1 | 1 |
| 9 | Copper | 7440-50-8 | 3.10E+03 | 1 | 1 | 1 | 1 |
| 10 | Lead | 7439-92-1 | 1.50E+02 | 0 | 0 | -1 | -1 |
| 11 | Manganese | 7439-96-5 | 3.90E+02 | 0 | 0 | -1 | -1 |
| 12 | Mercury | 7439-97-6 | 2.30E+01 | 1 | 1 | 1 | 1 |
| 14 | Nickel | 7440-02-0 | 1.00E+03 | 1 | 1 | 1 | 1 |
| 15 | Selenium | 7782-49-2 | 3.90E+02 | 1 | 1 | 1 | 1 |
| 16 | Silver | 7440-22-4 | 2.00E+02 | 1 | 1 | 1 | 1 |
| 17 | Thallium | 7440-28-0 | 5.50E+00 | 1 | 1 | 1 | 1 |
| 19 | Vanadium | 7440-62-2 | 5.50E+02 | 1 | 1 | 1 | 1 |
| 20 | Zinc | 7440-66-6 | 6.00E+03 | 1 | 1 | 1 | 1 |
| 21 | Cyanide | 57-12-5 | 2.00E+02 | 1 | 1 | 1 | 1 |
| 23 | Dichloromethane | 75-09-2 | 4.50E+01 | 1 | 1 | 1 | 1 |
| 24 | Trichloromethane | 67-66-3 | 1.20E+00 | 0 | 0 | -1 | -1 |
| 25 | Tetrachloromethane | 56-23-5 | 1.50E+00 | 0 | 0 | 1 | 1 |
| 26 | Bromoform | 75-25-2 | 8.10E+01 | 0 | 0 | 1 | 1 |
| 27 | Bromomethane | 74-83-9 | 8.00E-01 | -1 | -1 | -1 | -1 |
| 28 | 1,2-Dichloroethane | 107-06-2 | 9.00E-01 | 0 | 0 | -1 | -1 |
| 29 | 1,1,1-Trichloroethane | 71-55-6 | 5.40E+02 | 0 | 1 | -1 | 1 |
| 30 | 1,1,2-Trichloroethane | 79-00-5 | 3.60E+00 | 0 | 0 | -1 | 1 |
| 31 | Chloroethene | 75-01-4 | 2.00E-02 | -1 | -1 | -1 | -1 |
| 32 | 1,1-Dichloroethene | 75-35-4 | 2.00E-01 | -2 | -1 | -1 | -1 |
| 33 | Trichloroethene | 79-01-6 | 1.30E+01 | 1 | 1 | 1 | 1 |
| 34 | Tetrachloroethene | 127-18-4 | 1.20E+01 | 1 | 1 | 1 | 1 |
| 35 | Benzene | 71-43-2 | 2.50E+00 | 1 | 1 | 1 | 1 |
| 36 | Toluene | 108-88-3 | 1.90E+02 | 0 | 1 | 1 | 1 |
| 37 | Chlorobenzene | 108-90-7 | 2.10E+02 | 1 | 1 | 1 | 1 |
| 38 | 1,2-Dichlorobenzene | 95-50-1 | 5.10E+02 | 1 | 1 | 1 | 1 |
| 39 | 1,3-Dichlorobenzene | 541-73-1 | 4.30E+02 | 1 | 2 | 1 | 1 |
| 40 | 1,4-Dichlorobenzene | 106-46-7 | 2.70E+01 | 1 | 1 | 1 | 1 |
| 41 | 1,2,4Trichlorobenzene | 120-82-1 | 9.60E+01 | 1 | 1 | 1 | 1 |
| 42 | Hexachlorobenzene | 118-74-1 | 4.00E-01 | 0 | 0 | -1 | -1 |
| 44 | Ethylbenzene | 100-41-4 | 7.10E+01 | 1 | 1 | 1 | 1 |
| 45 | Styrene | 100-42-5 | 1.30E+01 | -1 | -1 | -1 | -1 |
| 46 | Xylenes | 1330-20-7 | 1.10E+02 | 0 | 0 | -1 | -1 |
| 48 | Phenol | 108-95-2 | 6.00E+03 | 2 | 2 | 1 | 1 |
| 49 | 2-Chlorophenol | 95-57-8 | 5.00E+01 | 0 | 1 | 1 | 1 |
| 50 | 2,4-Dichlorophenol | 120-83-2 | 3.00E+01 | 0 | 1 | 1 | 1 |
| 51 | 2,4,5-Trichlorophenol | 95-95-4 | 3.30E+02 | 0 | 0 | -1 | 1 |
| 52 | 2,4,6-Trichlorophenol | 88-06-2 | 5.80E+01 | 1 | 1 | 1 | 1 |
| 54 | Pentachlorophenol | 87-86-5 | 5.30E+00 | 0 | 0 | 1 | 1 |
| 55 | Benz(a)anthracene | 56-55-3 | 9.00E-01 | 0 | 0 | -1 | -1 |
| 56 | Benzo(a)pyrene | 50-32-8 | 4.00E-01 | 0 | 0 | -1 | -1 |
| 57 | Benzo(b)fluoranthene | 205-99-2 | 9.00E-01 | 0 | 0 | -1 | -1 |
| 58 | Benzo(k)fluoranthene | 207-08-9 | 9.00E-01 | -1 | -1 | -1 | -1 |
| 59 | Chrysene | 218-01-9 | 4.00E-01 | -2 | -2 | -1 | -1 |
| 60 | Dibena(a,h)anthracene | 53-70-3 | 4.00E-01 | 0 | 0 | -1 | -1 |
| 61 | Indeno(1,2,3-c,d)pyrebe | 193-39-5 | 9.00E-01 | 0 | 0 | -1 | -1 |
| 62 | Naphthalene | 91-20-3 | 5.40E+01 | 1 | 1 | 1 | 1 |
| 63 | Acenaphehene | 83-32-9 | 4.30E+01 | 0 | 0 | -1 | -1 |
| 64 | Acenaphthylene | 208-96-8 | 2.30E+01 | 0 | 0 | -1 | -1 |
| 65 | Anthracene | 120-12-7 | 3.50E+01 | 0 | -1 | -1 | -1 |
| 66 | Benzo(g,h,i) perylene | 191-24-2 | 8.00E-01 | -2 | -2 | -1 | -1 |
| 67 | Flouranthene | 206-44-0 | 2.00E+01 | 1 | -1 | 1 | -1 |
| 68 | Flouorene | 86-73-7 | 2.80E+01 | 0 | 0 | -1 | -1 |
| 69 | Phenanthrene | 85-01-8 | 4.00E+01 | 0 | 0 | -1 | -1 |
| 70 | Pyrene | 129-00-0 | 1.30E+01 | -1 | -1 | -1 | -1 |
| 72 | Chlordane | 57-74-9 | 5.00E-01 | -1 | -1 | -1 | -1 |
| 74 | Dieldrin | 60-57-1 | 4.00E-02 | 0 | -1 | -1 | -1 |
| 92 | Total PCB | 1336-36-3 | 1.00E+01 | 1 | 1 | 1 | 1 |
| 95 | Methyl ethyl ketone | 78-93-3 | 1.00E+04 | 0 | 1 | 1 | 1 |
| 96 | Methyl isobutyl ketone | 108-10-1 | 1.20E+03 | 0 | 0 | -1 | 1 |
| 98 | DEHP | 117-81-7 | 4.60E+01 | 0 | 0 | -1 | -1 |
| 99 | Hexachlorobutadine | 87-68-3 | 8.20E+00 | 0 | 1 | 1 | 1 |
| 100 | Methyl tert-butyl ether | 1634-04-4 | 3.90E+02 | 1 | 1 | 1 | 1 |
|  |  | Overall | C1 | C2 | C3 | C4 | C5 |
|  |  | Scores | 71 | 0.24 | 0.28 | 3 | 9 |
|  |  |  |  |  | No. >0 | 37 | 40 |
|  |  |  |  |  | No. <0 | 34 | 31 |

**Table S36** South Carolina Dept. of Health and Environmental Control

| Number | Pollutant | CAS. No | RGV (mg/kg) | C_2_ | C_3_ | C_4_ | C_5_ |
| --- | --- | --- | --- | --- | --- | --- | --- |
| 35 | Benzene | 71-43-2 | 1.20E+01 | 1 | 1 | 1 | 1 |
| 36 | Toluene | 108-88-3 | 1.60E+04 | 2 | 2 | 1 | 1 |
| 44 | Ethylbenzene | 100-41-4 | 7.80E+03 | 3 | 3 | 1 | 1 |
| 46 | Xylenes | 1330-20-7 | 1.60E+05 | 3 | 3 | 1 | 1 |
| 55 | Benz(a)anthracene | 56-55-3 | 8.80E-01 | 0 | 0 | -1 | -1 |
| 57 | Benzo(b)fluoranthene | 205-99-2 | 8.80E-01 | 0 | 0 | -1 | -1 |
| 58 | Benzo(k)fluoranthene | 207-08-9 | 8.80E+00 | 0 | 0 | 1 | 1 |
| 59 | Chrysene | 218-01-9 | 8.80E+01 | 1 | 1 | 1 | 1 |
| 60 | Dibena(a,h)anthracene | 53-70-3 | 8.80E-02 | -1 | -1 | -1 | -1 |
| 62 | Naphthalene | 91-20-3 | 3.10E+03 | 2 | 3 | 1 | 1 |
| 100 | Methyl tert-butyl ether | 1634-04-4 | 3.90E+02 | 1 | 1 | 1 | 1 |
|  |  | Overall | C1 | C2 | C3 | C4 | C5 |
|  |  | Scores | 11 | 1.09 | 1.18 | 5 | 5 |
|  |  |  |  |  | No. >0 | 8 | 8 |
|  |  |  |  |  | No. <0 | 3 | 3 |

**Table S37** South Dakota Department of Environment & Natural Resources

| Number | Pollutant | CAS. No | RGV (mg/kg) | C_2_ | C_3_ | C_4_ | C_5_ |
| --- | --- | --- | --- | --- | --- | --- | --- |
| 35 | Benzene | 71-43-2 | 1.70E+01 | 2 | 1 | 1 | 1 |
| 36 | Toluene | 108-88-3 | 7.50E+02 | 1 | 1 | 1 | 1 |
| 44 | Ethylbenzene | 100-41-4 | 6.50E+02 | 2 | 2 | 1 | 1 |
| 46 | Xylenes | 1330-20-7 | 5.00E+02 | 1 | 1 | 1 | 1 |
| 62 | Naphthalene | 91-20-3 | 6.20E+02 | 2 | 3 | 1 | 1 |
| 100 | Methyl tert-butyl ether | 1634-04-4 | 2.00E+02 | 1 | 1 | 1 | 1 |
|  |  | Overall | C1 | C2 | C3 | C4 | C5 |
|  |  | Scores | 6 | 1.5 | 1.5 | 6 | 6 |
|  |  |  |  |  | No. >0 | 6 | 6 |
|  |  |  |  |  | No. <0 | 0 | 0 |

**Table S38a** Texas Commission on Environmental Quality - Combined, 0.5-acre source

| Number | Pollutant | CAS. No | RGV (mg/kg) | C_2_ | C_3_ | C_4_ | C_5_ |
| --- | --- | --- | --- | --- | --- | --- | --- |
| 1 | Antimony | 7440-36-0 | 1.50E+01 | 0 | 0 | -1 | -1 |
| 2 | Arsenic | 7440-38-2 | 2.40E+01 | 0 | 1 | 1 | 1 |
| 3 | Barium | 7440-39-3 | 8.10E+03 | 1 | 1 | 1 | 1 |
| 4 | Beryllium | 7440-41-7 | 3.80E+01 | 1 | 0 | 1 | 1 |
| 5 | Cadmium | 7440-43-9 | 5.20E+01 | 1 | 1 | 1 | 1 |
| 6 | Chromium (III) | 16065-83-1 | 3.30E+04 | 2 | 2 | 1 | 1 |
| 7 | Chromium (VI) | 18540-29-9 | 1.20E+02 | 0 | 1 | 1 | 1 |
| 8 | Cobalt | 7440-48-4 | 4.00E+02 | 1 | 1 | 1 | 1 |
| 9 | Copper | 7440-50-8 | 1.30E+03 | 1 | 1 | 1 | 1 |
| 12 | Mercury | 7439-97-6 | 3.60E+00 | 0 | 0 | 1 | 1 |
| 13 | Molybdenum | 7439-98-7 | 1.60E+02 | 1 | 1 | 1 | 1 |
| 14 | Nickel | 7440-02-0 | 8.40E+02 | 1 | 1 | 1 | 1 |
| 15 | Selenium | 7782-49-2 | 3.10E+02 | 1 | 1 | 1 | 1 |
| 16 | Silver | 7440-22-4 | 9.70E+01 | 1 | 1 | 1 | 1 |
| 17 | Thallium | 7440-28-0 | 6.30E+00 | 1 | 1 | 1 | 1 |
| 18 | Tin | 7440-31-5 | 3.50E+04 | 2 | 2 | 1 | 1 |
| 19 | Vanadium | 7440-62-2 | 7.60E+01 | 0 | 0 | -1 | -1 |
| 20 | Zinc | 7440-66-6 | 9.90E+03 | 1 | 1 | 1 | 1 |
| 21 | Cyanide | 57-12-5 | 4.50E+01 | 0 | 0 | -1 | 1 |
| 22 | Acrylonitril | 107-13-1 | 3.60E+00 | 0 | 0 | 1 | 1 |
| 23 | Dichloromethane | 75-09-2 | 1.60E+03 | 2 | 2 | 1 | 1 |
| 24 | Trichloromethane | 67-66-3 | 1.60E+01 | 1 | 1 | 1 | 1 |
| 25 | Tetrachloromethane | 56-23-5 | 3.50E+01 | 2 | 2 | 1 | 1 |
| 26 | Bromoform | 75-25-2 | 4.00E+02 | 1 | 1 | 1 | 1 |
| 27 | Bromomethane | 74-83-9 | 4.60E+01 | 1 | 1 | 1 | 1 |
| 28 | 1,2-Dichloroethane | 107-06-2 | 1.10E+01 | 1 | 1 | 1 | 1 |
| 29 | 1,1,1-Trichloroethane | 71-55-6 | 5.30E+04 | 2 | 3 | 1 | 1 |
| 30 | 1,1,2-Trichloroethane | 79-00-5 | 1.80E+01 | 1 | 1 | 1 | 1 |
| 31 | Chloroethene | 75-01-4 | 3.70E+00 | 2 | 1 | 1 | 1 |
| 32 | 1,1-Dichloroethene | 75-35-4 | 2.30E+03 | 3 | 3 | 1 | 1 |
| 33 | Trichloroethene | 79-01-6 | 1.80E+01 | 1 | 1 | 1 | 1 |
| 34 | Tetrachloroethene | 127-18-4 | 7.10E+02 | 3 | 3 | 1 | 1 |
| 35 | Benzene | 71-43-2 | 1.20E+02 | 2 | 2 | 1 | 1 |
| 36 | Toluene | 108-88-3 | 5.90E+03 | 2 | 2 | 1 | 1 |
| 37 | Chlorobenzene | 108-90-7 | 5.20E+02 | 1 | 2 | 1 | 1 |
| 38 | 1,2-Dichlorobenzene | 95-50-1 | 7.20E+02 | 1 | 1 | 1 | 1 |
| 39 | 1,3-Dichlorobenzene | 541-73-1 | 1.20E+02 | 1 | 1 | 1 | 1 |
| 40 | 1,4-Dichlorobenzene | 106-46-7 | 2.50E+02 | 2 | 2 | 1 | 1 |
| 41 | 1,2,4Trichlorobenzene | 120-82-1 | 1.20E+02 | 1 | 1 | 1 | 1 |
| 42 | Hexachlorobenzene | 118-74-1 | 1.10E+00 | 0 | 0 | 1 | 1 |
| 43 | Nitrobenzene | 98-95-3 | 6.60E+01 | 1 | 1 | 1 | 1 |
| 44 | Ethylbenzene | 100-41-4 | 6.40E+03 | 3 | 3 | 1 | 1 |
| 45 | Styrene | 100-42-5 | 6.70E+03 | 2 | 2 | 1 | 1 |
| 46 | Xylenes | 1330-20-7 | 6.00E+03 | 2 | 2 | 1 | 1 |
| 47 | Cresol | 1319-77-3 | 3.30E+03 | 2 | 2 | 1 | 1 |
| 48 | Phenol | 108-95-2 | 1.80E+03 | 1 | 1 | 1 | 1 |
| 49 | 2-Chlorophenol | 95-57-8 | 4.10E+02 | 1 | 2 | 1 | 1 |
| 50 | 2,4-Dichlorophenol | 120-83-2 | 2.00E+02 | 1 | 2 | 1 | 1 |
| 51 | 2,4,5-Trichlorophenol | 95-95-4 | 6.70E+03 | 1 | 2 | 1 | 1 |
| 52 | 2,4,6-Trichlorophenol | 88-06-2 | 6.70E+01 | 1 | 1 | 1 | 1 |
| 53 | 2,3,4,6-Tetrahlorophenol | 58-90-2 | 1.80E+02 | 0 | 1 | -1 | 1 |
| 54 | Pentachlorophenol | 87-86-5 | 7.30E-01 | -1 | -1 | -1 | -1 |
| 55 | Benz(a)anthracene | 56-55-3 | 5.70E+00 | 1 | 1 | 1 | 1 |
| 56 | Benzo(a)pyrene | 50-32-8 | 5.60E-01 | 0 | 0 | -1 | -1 |
| 57 | Benzo(b)fluoranthene | 205-99-2 | 5.70E+00 | 1 | 1 | 1 | 1 |
| 58 | Benzo(k)fluoranthene | 207-08-9 | 5.70E+01 | 1 | 1 | 1 | 1 |
| 59 | Chrysene | 218-01-9 | 5.60E+02 | 1 | 2 | 1 | 1 |
| 60 | Dibena(a,h)anthracene | 53-70-3 | 5.50E-01 | 0 | 0 | -1 | -1 |
| 61 | Indeno(1,2,3-c,d)pyrebe | 193-39-5 | 5.70E+00 | 1 | 0 | 1 | 1 |
| 62 | Naphthalene | 91-20-3 | 2.20E+02 | 1 | 2 | 1 | 1 |
| 63 | Acenaphehene | 83-32-9 | 3.00E+03 | 2 | 2 | 1 | 1 |
| 64 | Acenaphthylene | 208-96-8 | 3.80E+03 | 2 | 2 | 1 | 1 |
| 65 | Anthracene | 120-12-7 | 1.80E+04 | 2 | 2 | 1 | 1 |
| 66 | Benzo(g,h,i) perylene | 191-24-2 | 1.80E+03 | 2 | 2 | 1 | 1 |
| 67 | Flouranthene | 206-44-0 | 2.30E+03 | 3 | 2 | 1 | 1 |
| 68 | Flouorene | 86-73-7 | 2.30E+03 | 1 | 1 | 1 | 1 |
| 69 | Phenanthrene | 85-01-8 | 1.70E+03 | 2 | 2 | 1 | 1 |
| 70 | Pyrene | 129-00-0 | 8.20E+01 | 0 | 0 | -1 | -1 |
| 71 | Aldrin | 309-00-2 | 5.00E-02 | 0 | 0 | -1 | -1 |
| 72 | Chlordane | 57-74-9 | 6.00E+00 | 1 | 1 | 1 | 1 |
| 73 | DDT | 50-29-3 | 5.40E+00 | 1 | 0 | 1 | 1 |
| 74 | Dieldrin | 60-57-1 | 1.50E-01 | 0 | 0 | -1 | -1 |
| 75 | Endosulfan | 115-29-7 | 4.00E+02 | 1 | 2 | 1 | 1 |
| 76 | Endrin | 72-20-8 | 9.00E+00 | 1 | 1 | 1 | 1 |
| 77 | Heptachlor | 76-44-8 | 1.30E-01 | 0 | 0 | -1 | -1 |
| 78 | Lindane | 58-89-9 | 1.10E+00 | 1 | 1 | 1 | 1 |
| 79 | Toxaphene | 8001-35-2 | 1.20E+00 | 1 | 0 | 1 | -1 |
| 80 | 2,4-D | 94-75-7 | 7.30E+02 | 0 | 1 | 1 | 1 |
| 81 | Atrazine | 1912-24-9 | 2.10E+01 | 1 | 1 | 1 | 1 |
| 82 | Carbaryl | 63-25-2 | 6.70E+03 | 0 | 2 | 1 | 1 |
| 83 | Carbofuran | 1563-66-2 | 3.30E+02 | 0 | 1 | 1 | 1 |
| 84 | Chloryrifos | 2921-88-2 | 1.30E+02 | 0 | 0 | 1 | 1 |
| 85 | Diuron | 330-54-1 | 1.30E+02 | 0 | 1 | 1 | 1 |
| 86 | Gylphosate | 1071-83-6 | 6.70E+03 | 0 | 1 | 1 | 1 |
| 87 | Malathion | 121-75-5 | 1.70E+02 | -1 | 0 | -1 | -1 |
| 88 | MCPA | 94-74-6 | 3.30E+01 | 0 | 0 | 1 | 1 |
| 89 | Picloram | 1918-02-1 | 4.70E+03 | 0 | 1 | 1 | 1 |
| 90 | Simazine | 122-34-9 | 3.90E+01 | 1 | 1 | 1 | 1 |
| 91 | Trifluralin | 1582-09-8 | 2.70E+02 | 1 | 1 | 1 | 1 |
| 92 | Total PCB | 1336-36-3 | 1.10E+00 | 0 | 0 | 1 | 1 |
| 95 | Methyl ethyl ketone | 78-93-3 | 4.00E+04 | 1 | 2 | 1 | 1 |
| 96 | Methyl isobutyl ketone | 108-10-1 | 5.90E+03 | 0 | 1 | 1 | 1 |
| 97 | Dibutyl phthalate | 84-74-2 | 6.20E+03 | 0 | 1 | 1 | 1 |
| 98 | DEHP | 117-81-7 | 4.30E+01 | 0 | 0 | -1 | -1 |
| 99 | Hexachlorobutadine | 87-68-3 | 2.00E+01 | 1 | 1 | 1 | 1 |
| 100 | Methyl tert-butyl ether | 1634-04-4 | 8.00E+02 | 1 | 2 | 1 | 1 |
|  |  | Overall | C1 | C2 | C3 | C4 | C5 |
|  |  | Scores | 96 | 0.94 | 1.11 | 70 | 72 |
|  |  |  |  |  | No. >0 | 83 | 84 |
|  |  |  |  |  | No. <0 | 13 | 12 |

**Table S38b** Texas Commission on Environmental Quality - Combined, 30-acre source

| Number | Pollutant | CAS. No | RGV (mg/kg) | C_2_ | C_3_ | C_4_ | C_5_ |
| --- | --- | --- | --- | --- | --- | --- | --- |
| 1 | Antimony | 7440-36-0 | 1.50E+01 | 0 | 0 | -1 | -1 |
| 2 | Arsenic | 7440-38-2 | 2.40E+01 | 0 | 1 | 1 | 1 |
| 3 | Barium | 7440-39-3 | 8.10E+03 | 1 | 1 | 1 | 1 |
| 4 | Beryllium | 7440-41-7 | 3.80E+01 | 1 | 0 | 1 | 1 |
| 5 | Cadmium | 7440-43-9 | 5.10E+01 | 1 | 1 | 1 | 1 |
| 6 | Chromium (III) | 16065-83-1 | 2.70E+04 | 2 | 2 | 1 | 1 |
| 7 | Chromium (VI) | 18540-29-9 | 1.20E+02 | 0 | 1 | 1 | 1 |
| 8 | Cobalt | 7440-48-4 | 3.70E+02 | 1 | 1 | 1 | 1 |
| 9 | Copper | 7440-50-8 | 1.30E+03 | 1 | 1 | 1 | 1 |
| 12 | Mercury | 7439-97-6 | 2.10E+00 | 0 | 0 | -1 | -1 |
| 13 | Molybdenum | 7439-98-7 | 1.60E+02 | 1 | 1 | 1 | 1 |
| 14 | Nickel | 7440-02-0 | 8.40E+02 | 1 | 1 | 1 | 1 |
| 15 | Selenium | 7782-49-2 | 3.10E+02 | 1 | 1 | 1 | 1 |
| 16 | Silver | 7440-22-4 | 9.70E+01 | 1 | 1 | 1 | 1 |
| 17 | Thallium | 7440-28-0 | 6.30E+00 | 1 | 1 | 1 | 1 |
| 18 | Tin | 7440-31-5 | 3.50E+04 | 2 | 2 | 1 | 1 |
| 19 | Vanadium | 7440-62-2 | 7.50E+01 | 0 | 0 | -1 | -1 |
| 20 | Zinc | 7440-66-6 | 9.90E+03 | 1 | 1 | 1 | 1 |
| 21 | Cyanide | 57-12-5 | 4.30E+01 | 0 | 0 | -1 | 1 |
| 22 | Acrylonitril | 107-13-1 | 2.20E+00 | 0 | 0 | 1 | 1 |
| 23 | Dichloromethane | 75-09-2 | 1.50E+03 | 2 | 2 | 1 | 1 |
| 24 | Trichloromethane | 67-66-3 | 8.00E+00 | 1 | 1 | 1 | 1 |
| 25 | Tetrachloromethane | 56-23-5 | 2.30E+01 | 2 | 2 | 1 | 1 |
| 26 | Bromoform | 75-25-2 | 2.80E+02 | 1 | 1 | 1 | 1 |
| 27 | Bromomethane | 74-83-9 | 2.90E+01 | 1 | 1 | 1 | 1 |
| 28 | 1,2-Dichloroethane | 107-06-2 | 6.40E+00 | 1 | 1 | 1 | 1 |
| 29 | 1,1,1-Trichloroethane | 71-55-6 | 3.20E+04 | 2 | 2 | 1 | 1 |
| 30 | 1,1,2-Trichloroethane | 79-00-5 | 1.00E+01 | 1 | 1 | 1 | 1 |
| 31 | Chloroethene | 75-01-4 | 3.40E+00 | 2 | 1 | 1 | 1 |
| 32 | 1,1-Dichloroethene | 75-35-4 | 1.60E+03 | 2 | 2 | 1 | 1 |
| 33 | Trichloroethene | 79-01-6 | 1.10E+01 | 1 | 1 | 1 | 1 |
| 34 | Tetrachloroethene | 127-18-4 | 4.20E+02 | 2 | 2 | 1 | 1 |
| 35 | Benzene | 71-43-2 | 6.90E+01 | 2 | 2 | 1 | 1 |
| 36 | Toluene | 108-88-3 | 5.40E+03 | 2 | 2 | 1 | 1 |
| 37 | Chlorobenzene | 108-90-7 | 3.20E+02 | 1 | 1 | 1 | 1 |
| 38 | 1,2-Dichlorobenzene | 95-50-1 | 3.90E+02 | 1 | 1 | 1 | 1 |
| 39 | 1,3-Dichlorobenzene | 541-73-1 | 6.20E+01 | 1 | 1 | 1 | 1 |
| 40 | 1,4-Dichlorobenzene | 106-46-7 | 2.50E+02 | 2 | 2 | 1 | 1 |
| 41 | 1,2,4Trichlorobenzene | 120-82-1 | 7.00E+01 | 1 | 1 | 1 | 1 |
| 42 | Hexachlorobenzene | 118-74-1 | 1.00E+00 | 0 | 0 | 1 | 1 |
| 43 | Nitrobenzene | 98-95-3 | 3.40E+01 | 1 | 1 | 1 | 1 |
| 44 | Ethylbenzene | 100-41-4 | 5.30E+03 | 3 | 2 | 1 | 1 |
| 45 | Styrene | 100-42-5 | 4.30E+03 | 2 | 2 | 1 | 1 |
| 46 | Xylenes | 1330-20-7 | 3.70E+03 | 2 | 2 | 1 | 1 |
| 47 | Cresol | 1319-77-3 | 3.30E+03 | 2 | 2 | 1 | 1 |
| 48 | Phenol | 108-95-2 | 9.50E+02 | 1 | 1 | 1 | 1 |
| 49 | 2-Chlorophenol | 95-57-8 | 4.10E+02 | 1 | 2 | 1 | 1 |
| 50 | 2,4-Dichlorophenol | 120-83-2 | 2.00E+02 | 1 | 2 | 1 | 1 |
| 51 | 2,4,5-Trichlorophenol | 95-95-4 | 6.70E+03 | 1 | 2 | 1 | 1 |
| 52 | 2,4,6-Trichlorophenol | 88-06-2 | 6.70E+01 | 1 | 1 | 1 | 1 |
| 53 | 2,3,4,6-Tetrahlorophenol | 58-90-2 | 1.80E+02 | 0 | 1 | -1 | 1 |
| 54 | Pentachlorophenol | 87-86-5 | 7.30E-01 | -1 | -1 | -1 | -1 |
| 55 | Benz(a)anthracene | 56-55-3 | 5.60E+00 | 1 | 1 | 1 | 1 |
| 56 | Benzo(a)pyrene | 50-32-8 | 5.60E-01 | 0 | 0 | -1 | -1 |
| 57 | Benzo(b)fluoranthene | 205-99-2 | 5.70E+00 | 1 | 1 | 1 | 1 |
| 58 | Benzo(k)fluoranthene | 207-08-9 | 5.70E+01 | 1 | 1 | 1 | 1 |
| 59 | Chrysene | 218-01-9 | 5.60E+02 | 1 | 2 | 1 | 1 |
| 60 | Dibena(a,h)anthracene | 53-70-3 | 5.50E-01 | 0 | 0 | -1 | -1 |
| 61 | Indeno(1,2,3-c,d)pyrebe | 193-39-5 | 5.70E+00 | 1 | 0 | 1 | 1 |
| 62 | Naphthalene | 91-20-3 | 1.20E+02 | 1 | 2 | 1 | 1 |
| 63 | Acenaphehene | 83-32-9 | 3.00E+03 | 2 | 2 | 1 | 1 |
| 64 | Acenaphthylene | 208-96-8 | 3.80E+03 | 2 | 2 | 1 | 1 |
| 65 | Anthracene | 120-12-7 | 1.80E+04 | 2 | 2 | 1 | 1 |
| 66 | Benzo(g,h,i) perylene | 191-24-2 | 1.80E+03 | 2 | 2 | 1 | 1 |
| 67 | Flouranthene | 206-44-0 | 2.30E+03 | 3 | 2 | 1 | 1 |
| 68 | Flouorene | 86-73-7 | 2.30E+03 | 1 | 1 | 1 | 1 |
| 69 | Phenanthrene | 85-01-8 | 1.70E+03 | 2 | 2 | 1 | 1 |
| 70 | Pyrene | 129-00-0 | 8.20E+01 | 0 | 0 | -1 | -1 |
| 71 | Aldrin | 309-00-2 | 5.00E-02 | 0 | 0 | -1 | -1 |
| 72 | Chlordane | 57-74-9 | 5.90E+00 | 1 | 1 | 1 | 1 |
| 73 | DDT | 50-29-3 | 5.40E+00 | 1 | 0 | 1 | 1 |
| 74 | Dieldrin | 60-57-1 | 1.50E-01 | 0 | 0 | -1 | -1 |
| 75 | Endosulfan | 115-29-7 | 4.00E+02 | 1 | 2 | 1 | 1 |
| 76 | Endrin | 72-20-8 | 9.00E+00 | 1 | 1 | 1 | 1 |
| 77 | Heptachlor | 76-44-8 | 1.30E-01 | 0 | 0 | -1 | -1 |
| 78 | Lindane | 58-89-9 | 1.10E+00 | 1 | 1 | 1 | 1 |
| 79 | Toxaphene | 8001-35-2 | 1.20E+00 | 1 | 0 | 1 | -1 |
| 80 | 2,4-D | 94-75-7 | 7.30E+02 | 0 | 1 | 1 | 1 |
| 81 | Atrazine | 1912-24-9 | 2.10E+01 | 1 | 1 | 1 | 1 |
| 82 | Carbaryl | 63-25-2 | 6.70E+03 | 0 | 2 | 1 | 1 |
| 83 | Carbofuran | 1563-66-2 | 3.30E+02 | 0 | 1 | 1 | 1 |
| 84 | Chloryrifos | 2921-88-2 | 1.30E+02 | 0 | 0 | 1 | 1 |
| 85 | Diuron | 330-54-1 | 1.30E+02 | 0 | 1 | 1 | 1 |
| 86 | Gylphosate | 1071-83-6 | 6.70E+03 | 0 | 1 | 1 | 1 |
| 87 | Malathion | 121-75-5 | 9.60E+01 | -1 | 0 | -1 | -1 |
| 88 | MCPA | 94-74-6 | 3.30E+01 | 0 | 0 | 1 | 1 |
| 89 | Picloram | 1918-02-1 | 4.70E+03 | 0 | 1 | 1 | 1 |
| 90 | Simazine | 122-34-9 | 3.90E+01 | 1 | 1 | 1 | 1 |
| 91 | Trifluralin | 1582-09-8 | 2.70E+02 | 1 | 1 | 1 | 1 |
| 92 | Total PCB | 1336-36-3 | 1.10E+00 | 0 | 0 | 1 | 1 |
| 95 | Methyl ethyl ketone | 78-93-3 | 3.30E+04 | 1 | 2 | 1 | 1 |
| 96 | Methyl isobutyl ketone | 108-10-1 | 5.40E+03 | 0 | 1 | 1 | 1 |
| 97 | Dibutyl phthalate | 84-74-2 | 6.20E+03 | 0 | 1 | 1 | 1 |
| 98 | DEHP | 117-81-7 | 4.30E+01 | 0 | 0 | -1 | -1 |
| 99 | Hexachlorobutadine | 87-68-3 | 1.20E+01 | 1 | 1 | 1 | 1 |
| 100 | Methyl tert-butyl ether | 1634-04-4 | 5.90E+02 | 1 | 1 | 1 | 1 |
|  |  | Overall | C1 | C2 | C3 | C4 | C5 |
|  |  | Scores | 96 | 0.92 | 1.05 | 68 | 70 |
|  |  |  |  |  | No. >0 | 82 | 83 |
|  |  |  |  |  | No. <0 | 14 | 13 |

**Table S39** Utah Department of Environmental Quality

| Number | Pollutant | CAS. No | RGV (mg/kg) | C_2_ | C_3_ | C_4_ | C_5_ |
| --- | --- | --- | --- | --- | --- | --- | --- |
| 35 | Benzene | 71-43-2 | 2.00E-01 | 0 | 0 | -1 | -1 |
| 36 | Toluene | 108-88-3 | 9.00E+00 | -1 | -1 | -1 | -1 |
| 44 | Ethylbenzene | 100-41-4 | 5.00E+00 | 0 | -1 | -1 | -1 |
| 46 | Xylenes | 1330-20-7 | 1.42E+02 | 0 | 0 | -1 | 1 |
| 62 | Naphthalene | 91-20-3 | 5.10E+01 | 1 | 1 | 1 | 1 |
| 100 | Methyl tert-butyl ether | 1634-04-4 | 3.00E-01 | -2 | -2 | -1 | -1 |
|  |  | Overall | C1 | C2 | C3 | C4 | C5 |
|  |  | Scores | 6 | -0.33 | -0.5 | -4 | -2 |
|  |  |  |  |  | No. >0 | 1 | 2 |
|  |  |  |  |  | No. <0 | 5 | 4 |

**Table S40** Vermont Department of Environment

| Number | Pollutant | CAS. No | RGV (mg/kg) | C_2_ | C_3_ | C_4_ | C_5_ |
| --- | --- | --- | --- | --- | --- | --- | --- |
| 1 | Antimony | 7440-36-0 | 3.10E+01 | 0 | 1 | 1 | 1 |
| 2 | Arsenic | 7440-38-2 | 6.10E-01 | -1 | -1 | -1 | -1 |
| 3 | Barium | 7440-39-3 | 1.50E+04 | 1 | 1 | 1 | 1 |
| 4 | Beryllium | 7440-41-7 | 1.60E+02 | 1 | 1 | 1 | 1 |
| 5 | Cadmium | 7440-43-9 | 7.00E+01 | 1 | 1 | 1 | 1 |
| 6 | Chromium (III) | 16065-83-1 | 1.20E+05 | 3 | 2 | 1 | 1 |
| 7 | Chromium (VI) | 18540-29-9 | 2.90E-01 | -2 | -2 | -1 | -1 |
| 8 | Cobalt | 7440-48-4 | 2.30E+01 | 0 | 0 | -1 | -1 |
| 9 | Copper | 7440-50-8 | 3.10E+03 | 1 | 1 | 1 | 1 |
| 10 | Lead | 7439-92-1 | 4.00E+02 | 0 | 1 | 1 | 1 |
| 11 | Manganese | 7439-96-5 | 1.80E+03 | 0 | 0 | 1 | 1 |
| 12 | Mercury | 7439-97-6 | 1.00E+01 | 1 | 1 | 1 | 1 |
| 13 | Molybdenum | 7439-98-7 | 3.90E+02 | 1 | 1 | 1 | 1 |
| 14 | Nickel | 7440-02-0 | 1.50E+03 | 1 | 1 | 1 | 1 |
| 15 | Selenium | 7782-49-2 | 3.90E+02 | 1 | 1 | 1 | 1 |
| 16 | Silver | 7440-22-4 | 3.90E+02 | 1 | 1 | 1 | 1 |
| 17 | Thallium | 7440-28-0 | 7.80E-01 | 0 | 0 | -1 | -1 |
| 18 | Tin | 7440-31-5 | 4.70E+04 | 3 | 2 | 1 | 1 |
| 19 | Vanadium | 7440-62-2 | 3.90E+02 | 1 | 1 | 1 | 1 |
| 20 | Zinc | 7440-66-6 | 2.30E+04 | 2 | 2 | 1 | 1 |
| 21 | Cyanide | 57-12-5 | 2.20E+01 | 0 | 0 | -1 | -1 |
| 22 | Acrylonitril | 107-13-1 | 2.40E-01 | -1 | -1 | -1 | -1 |
| 23 | Dichloromethane | 75-09-2 | 5.60E+01 | 1 | 1 | 1 | 1 |
| 24 | Trichloromethane | 67-66-3 | 2.90E-01 | 0 | -1 | -1 | -1 |
| 25 | Tetrachloromethane | 56-23-5 | 6.10E-01 | 0 | 0 | -1 | -1 |
| 26 | Bromoform | 75-25-2 | 6.20E+01 | 0 | 0 | -1 | 1 |
| 27 | Bromomethane | 74-83-9 | 7.30E+00 | 0 | 0 | -1 | 1 |
| 28 | 1,2-Dichloroethane | 107-06-2 | 4.30E-01 | 0 | 0 | -1 | -1 |
| 29 | 1,1,1-Trichloroethane | 71-55-6 | 8.70E+03 | 1 | 2 | 1 | 1 |
| 30 | 1,1,2-Trichloroethane | 79-00-5 | 1.10E+00 | 0 | 0 | -1 | -1 |
| 31 | Chloroethene | 75-01-4 | 6.00E-02 | 0 | 0 | -1 | -1 |
| 32 | 1,1-Dichloroethene | 75-35-4 | 2.40E+02 | 2 | 2 | 1 | 1 |
| 33 | Trichloroethene | 79-01-6 | 9.10E-01 | 0 | 0 | -1 | -1 |
| 34 | Tetrachloroethene | 127-18-4 | 2.20E+01 | 1 | 1 | 1 | 1 |
| 35 | Benzene | 71-43-2 | 1.10E+00 | 0 | 0 | 1 | 1 |
| 36 | Toluene | 108-88-3 | 5.00E+03 | 2 | 2 | 1 | 1 |
| 37 | Chlorobenzene | 108-90-7 | 2.90E+02 | 1 | 1 | 1 | 1 |
| 38 | 1,2-Dichlorobenzene | 95-50-1 | 1.90E+03 | 1 | 2 | 1 | 1 |
| 40 | 1,4-Dichlorobenzene | 106-46-7 | 2.40E+00 | 0 | 0 | -1 | -1 |
| 41 | 1,2,4Trichlorobenzene | 120-82-1 | 2.20E+01 | 0 | 0 | -1 | -1 |
| 42 | Hexachlorobenzene | 118-74-1 | 3.00E-01 | 0 | 0 | -1 | -1 |
| 43 | Nitrobenzene | 98-95-3 | 4.80E+00 | 0 | 0 | -1 | -1 |
| 44 | Ethylbenzene | 100-41-4 | 5.40E+00 | 0 | -1 | -1 | -1 |
| 45 | Styrene | 100-42-5 | 6.30E+03 | 2 | 2 | 1 | 1 |
| 46 | Xylenes | 1330-20-7 | 6.30E+02 | 1 | 1 | 1 | 1 |
| 47 | Cresol | 1319-77-3 | 6.10E+03 | 2 | 2 | 1 | 1 |
| 48 | Phenol | 108-95-2 | 1.80E+04 | 2 | 2 | 1 | 1 |
| 49 | 2-Chlorophenol | 95-57-8 | 3.90E+02 | 1 | 1 | 1 | 1 |
| 50 | 2,4-Dichlorophenol | 120-83-2 | 1.80E+02 | 1 | 1 | 1 | 1 |
| 51 | 2,4,5-Trichlorophenol | 95-95-4 | 6.10E+03 | 1 | 2 | 1 | 1 |
| 52 | 2,4,6-Trichlorophenol | 88-06-2 | 4.40E+01 | 1 | 1 | 1 | 1 |
| 53 | 2,3,4,6-Tetrahlorophenol | 58-90-2 | 1.80E+03 | 1 | 2 | 1 | 1 |
| 54 | Pentachlorophenol | 87-86-5 | 8.90E-01 | -1 | 0 | -1 | -1 |
| 55 | Benz(a)anthracene | 56-55-3 | 1.50E-01 | -1 | -1 | -1 | -1 |
| 56 | Benzo(a)pyrene | 50-32-8 | 1.50E-02 | -1 | -1 | -1 | -1 |
| 57 | Benzo(b)fluoranthene | 205-99-2 | 1.50E-01 | -1 | -1 | -1 | -1 |
| 58 | Benzo(k)fluoranthene | 207-08-9 | 1.50E+00 | -1 | 0 | -1 | -1 |
| 59 | Chrysene | 218-01-9 | 1.50E+01 | 0 | 0 | -1 | -1 |
| 60 | Dibena(a,h)anthracene | 53-70-3 | 1.50E-02 | -1 | -1 | -1 | -1 |
| 61 | Indeno(1,2,3-c,d)pyrebe | 193-39-5 | 1.50E-01 | -1 | -1 | -1 | -1 |
| 62 | Naphthalene | 91-20-3 | 3.60E+00 | -1 | 0 | -1 | 1 |
| 63 | Acenaphehene | 83-32-9 | 3.40E+03 | 2 | 2 | 1 | 1 |
| 65 | Anthracene | 120-12-7 | 1.70E+04 | 2 | 2 | 1 | 1 |
| 67 | Flouranthene | 206-44-0 | 2.30E+03 | 3 | 2 | 1 | 1 |
| 68 | Flouorene | 86-73-7 | 2.30E+03 | 1 | 1 | 1 | 1 |
| 70 | Pyrene | 129-00-0 | 1.70E+03 | 1 | 1 | 1 | 1 |
| 71 | Aldrin | 309-00-2 | 2.90E-02 | 0 | -1 | -1 | -1 |
| 72 | Chlordane | 57-74-9 | 1.60E+00 | 0 | 0 | -1 | -1 |
| 73 | DDT | 50-29-3 | 1.70E+00 | 0 | 0 | -1 | -1 |
| 74 | Dieldrin | 60-57-1 | 3.00E-02 | 0 | -1 | -1 | -1 |
| 75 | Endosulfan | 115-29-7 | 3.70E+02 | 1 | 2 | 1 | 1 |
| 76 | Endrin | 72-20-8 | 1.80E+01 | 1 | 1 | 1 | 1 |
| 77 | Heptachlor | 76-44-8 | 1.10E-01 | 0 | 0 | -1 | -1 |
| 78 | Lindane | 58-89-9 | 5.20E-01 | 0 | 0 | -1 | 1 |
| 79 | Toxaphene | 8001-35-2 | 4.40E-01 | 0 | 0 | -1 | -1 |
| 80 | 2,4-D | 94-75-7 | 6.90E+02 | 0 | 1 | -1 | 1 |
| 81 | Atrazine | 1912-24-9 | 2.10E+00 | 0 | 0 | -1 | -1 |
| 82 | Carbaryl | 63-25-2 | 6.10E+03 | 0 | 2 | 1 | 1 |
| 83 | Carbofuran | 1563-66-2 | 3.10E+02 | 0 | 1 | 1 | 1 |
| 84 | Chloryrifos | 2921-88-2 | 6.10E+01 | 0 | 0 | -1 | -1 |
| 85 | Diuron | 330-54-1 | 1.20E+02 | 0 | 1 | -1 | 1 |
| 86 | Gylphosate | 1071-83-6 | 6.10E+03 | 0 | 1 | -1 | 1 |
| 87 | Malathion | 121-75-5 | 1.20E+03 | 0 | 1 | -1 | 1 |
| 88 | MCPA | 94-74-6 | 3.10E+01 | 0 | 0 | -1 | 1 |
| 89 | Picloram | 1918-02-1 | 4.30E+03 | 0 | 1 | -1 | 1 |
| 90 | Simazine | 122-34-9 | 4.10E+00 | 0 | 0 | -1 | -1 |
| 91 | Trifluralin | 1582-09-8 | 6.30E+01 | 0 | 0 | -1 | -1 |
| 92 | Total PCB | 1336-36-3 | 2.20E-01 | 0 | 0 | -1 | -1 |
| 93 | PCB 118 | 31508-00-6 | 1.10E-01 | 0 | 0 | -1 | -1 |
| 94 | Total PCDD/PCDF | 1746-01-6 | 4.50E-06 | -1 | -1 | -1 | -1 |
| 95 | Methyl ethyl ketone | 78-93-3 | 2.80E+04 | 1 | 2 | 1 | 1 |
| 96 | Methyl isobutyl ketone | 108-10-1 | 5.30E+03 | 0 | 1 | 1 | 1 |
| 97 | Dibutyl phthalate | 84-74-2 | 6.10E+03 | 0 | 1 | -1 | 1 |
| 98 | DEHP | 117-81-7 | 3.50E+01 | 0 | 0 | -1 | -1 |
| 99 | Hexachlorobutadine | 87-68-3 | 6.20E+00 | 0 | 0 | -1 | 1 |
| 100 | Methyl tert-butyl ether | 1634-04-4 | 4.30E+01 | 0 | 0 | -1 | 1 |
|  |  | Overall | C1 | C2 | C3 | C4 | C5 |
|  |  | Scores | 96 | 0.40 | 0.54 | -8 | 18 |
|  |  |  |  |  | No. >0 | 44 | 57 |
|  |  |  |  |  | No. <0 | 52 | 39 |

**Table S41a** Virginia Dept. of Environmental Quality - RSL

| Number | Pollutant | CAS. No | RGV | RGV (mg/kg) | C_2_ | C_3_ | C_4_ |
| --- | --- | --- | --- | --- | --- | --- | --- |
| 1 | Antimony | 7440-36-0 | 3.10E+00 | -1 | 0 | -1 | -1 |
| 2 | Arsenic | 7440-38-2 | 3.40E+00 | -1 | 0 | -1 | -1 |
| 3 | Barium | 7440-39-3 | 1.50E+03 | 0 | 0 | 1 | 1 |
| 4 | Beryllium | 7440-41-7 | 1.60E+01 | 0 | 0 | -1 | -1 |
| 5 | Cadmium | 7440-43-9 | 7.00E+00 | 0 | 0 | -1 | -1 |
| 6 | Chromium (III) | 16065-83-1 | 3.00E+00 | -2 | -2 | -1 | -1 |
| 7 | Chromium (VI) | 18540-29-9 | 3.00E+00 | -1 | -1 | -1 | -1 |
| 8 | Cobalt | 7440-48-4 | 2.30E+00 | -1 | -1 | -1 | -1 |
| 9 | Copper | 7440-50-8 | 3.10E+02 | 0 | 0 | 1 | 1 |
| 10 | Lead | 7439-92-1 | 4.00E+02 | 0 | 1 | 1 | 1 |
| 11 | Manganese | 7439-96-5 | 1.80E+02 | -1 | -1 | -1 | -1 |
| 12 | Mercury | 7439-97-6 | 9.40E-01 | 0 | 0 | -1 | -1 |
| 14 | Nickel | 7440-02-0 | 1.50E+02 | 0 | 0 | 1 | -1 |
| 15 | Selenium | 7782-49-2 | 3.90E+01 | 0 | 0 | 1 | 1 |
| 16 | Silver | 7440-22-4 | 3.90E+01 | 0 | 0 | -1 | -1 |
| 17 | Thallium | 7440-28-0 | 7.80E-02 | -1 | -1 | -1 | -1 |
| 19 | Vanadium | 7440-62-2 | 3.90E+01 | 0 | 0 | -1 | -1 |
| 20 | Zinc | 7440-66-6 | 2.30E+03 | 1 | 1 | 1 | 1 |
| 21 | Cyanide | 57-12-5 | 2.10E+00 | -1 | -1 | -1 | -1 |
| 23 | Dichloromethane | 75-09-2 | 3.50E+01 | 1 | 1 | 1 | 1 |
| 24 | Trichloromethane | 67-66-3 | 3.20E+00 | 1 | 0 | 1 | 1 |
| 25 | Tetrachloromethane | 56-23-5 | 6.50E+00 | 1 | 1 | 1 | 1 |
| 26 | Bromoform | 75-25-2 | 1.20E+02 | 0 | 1 | 1 | 1 |
| 27 | Bromomethane | 74-83-9 | 6.80E-01 | -1 | -1 | -1 | -1 |
| 28 | 1,2-Dichloroethane | 107-06-2 | 3.10E+00 | 0 | 1 | 1 | 1 |
| 31 | Chloroethene | 75-01-4 | 5.90E-01 | 1 | 1 | 1 | 1 |
| 32 | 1,1-Dichloroethene | 75-35-4 | 2.30E+01 | 1 | 1 | 1 | 1 |
| 33 | Trichloroethene | 79-01-6 | 4.10E-01 | -1 | -1 | -1 | -1 |
| 34 | Tetrachloroethene | 127-18-4 | 8.10E+00 | 1 | 1 | 1 | 1 |
| 35 | Benzene | 71-43-2 | 8.20E+00 | 1 | 1 | 1 | 1 |
| 36 | Toluene | 108-88-3 | 4.90E+02 | 1 | 1 | 1 | 1 |
| 37 | Chlorobenzene | 108-90-7 | 2.80E+01 | 0 | 0 | -1 | 1 |
| 38 | 1,2-Dichlorobenzene | 95-50-1 | 1.80E+02 | 0 | 1 | -1 | 1 |
| 40 | 1,4-Dichlorobenzene | 106-46-7 | 2.60E+01 | 1 | 1 | 1 | 1 |
| 41 | 1,2,4Trichlorobenzene | 120-82-1 | 5.80E+00 | 0 | 0 | -1 | -1 |
| 42 | Hexachlorobenzene | 118-74-1 | 3.30E+00 | 1 | 1 | 1 | 1 |
| 43 | Nitrobenzene | 98-95-3 | 1.30E+01 | 0 | 0 | -1 | 1 |
| 44 | Ethylbenzene | 100-41-4 | 5.80E+01 | 1 | 1 | 1 | 1 |
| 45 | Styrene | 100-42-5 | 6.00E+02 | 1 | 1 | 1 | 1 |
| 46 | Xylenes | 1330-20-7 | 5.80E+01 | 0 | 0 | -1 | -1 |
| 47 | Cresol | 1319-77-3 | 3.10E+02 | 0 | 1 | 1 | 1 |
| 48 | Phenol | 108-95-2 | 1.80E+03 | 1 | 1 | 1 | 1 |
| 49 | 2-Chlorophenol | 95-57-8 | 3.90E+01 | 0 | 0 | -1 | 1 |
| 50 | 2,4-Dichlorophenol | 120-83-2 | 1.80E+01 | 0 | 0 | -1 | 1 |
| 51 | 2,4,5-Trichlorophenol | 95-95-4 | 6.20E+02 | 0 | 1 | 1 | 1 |
| 52 | 2,4,6-Trichlorophenol | 88-06-2 | 6.20E+00 | 0 | 0 | -1 | -1 |
| 53 | 2,3,4,6-Tetrahlorophenol | 58-90-2 | 1.80E+02 | 0 | 1 | -1 | 1 |
| 54 | Pentachlorophenol | 87-86-5 | 9.90E+00 | 1 | 1 | 1 | 1 |
| 59 | Chrysene | 218-01-9 | 1.50E+02 | 1 | 1 | 1 | 1 |
| 60 | Dibena(a,h)anthracene | 53-70-3 | 1.50E-01 | 0 | 0 | -1 | -1 |
| 61 | Indeno(1,2,3-c,d)pyrebe | 193-39-5 | 1.50E+00 | 0 | 0 | -1 | -1 |
| 62 | Naphthalene | 91-20-3 | 1.30E+01 | 0 | 1 | -1 | 1 |
| 63 | Acenaphehene | 83-32-9 | 3.50E+02 | 1 | 1 | 1 | 1 |
| 64 | Acenaphthylene | 208-96-8 | 1.70E+02 | 1 | 1 | 1 | 1 |
| 65 | Anthracene | 120-12-7 | 1.70E+03 | 1 | 1 | 1 | 1 |
| 66 | Benzo(g,h,i) perylene | 191-24-2 | 1.70E+02 | 1 | 1 | 1 | 1 |
| 67 | Flouranthene | 206-44-0 | 2.30E+02 | 2 | 1 | 1 | 1 |
| 68 | Flouorene | 86-73-7 | 2.30E+02 | 0 | 0 | 1 | 1 |
| 69 | Phenanthrene | 85-01-8 | 1.70E+02 | 1 | 1 | 1 | 1 |
| 70 | Pyrene | 129-00-0 | 1.70E+02 | 0 | 0 | -1 | 1 |
| 71 | Aldrin | 309-00-2 | 1.80E-01 | 0 | 0 | 1 | -1 |
| 72 | Chlordane | 57-74-9 | 3.50E+00 | 0 | 0 | 1 | 1 |
| 73 | DDT | 50-29-3 | 3.60E+00 | 0 | 0 | 1 | 1 |
| 74 | Dieldrin | 60-57-1 | 3.10E-01 | 1 | 0 | 1 | 1 |
| 75 | Endosulfan | 115-29-7 | 3.70E+01 | 0 | 1 | -1 | 1 |
| 76 | Endrin | 72-20-8 | 1.80E+00 | 0 | 0 | -1 | -1 |
| 77 | Heptachlor | 76-44-8 | 1.20E+00 | 1 | 1 | 1 | 1 |
| 78 | Lindane | 58-89-9 | 2.10E+00 | 1 | 1 | 1 | 1 |
| 79 | Toxaphene | 8001-35-2 | 4.80E+00 | 1 | 1 | 1 | 1 |
| 94 | Total PCDD/PCDF | 1746-01-6 | 5.10E-06 | -1 | -1 | -1 | -1 |
| 95 | Methyl ethyl ketone | 78-93-3 | 2.70E+03 | 0 | 1 | -1 | 1 |
| 96 | Methyl isobutyl ketone | 108-10-1 | 5.30E+02 | -1 | 0 | -1 | -1 |
| 97 | Dibutyl phthalate | 84-74-2 | 6.20E+02 | -1 | 0 | -1 | -1 |
| 98 | DEHP | 117-81-7 | 1.20E+02 | 0 | 0 | 1 | -1 |
| 99 | Hexachlorobutadine | 87-68-3 | 6.20E+00 | 0 | 0 | -1 | 1 |
| 100 | Methyl tert-butyl ether | 1634-04-4 | 4.70E+02 | 1 | 1 | 1 | 1 |
|  |  | Overall | C1 | C2 | C3 | C4 | C5 |
|  |  | Scores | 76 | 0.18 | 0.33 | 6 | 22 |
|  |  |  |  |  | No. >0 | 41 | 49 |
|  |  |  |  |  | No. <0 | 35 | 27 |

**Table S41b** Virginia Dept. of Environmental Quality - VRP

| Number | Pollutant | CAS. No | RGV (mg/kg) | C_2_ | C_3_ | C_4_ | C_5_ |
| --- | --- | --- | --- | --- | --- | --- | --- |
| 1 | Antimony | 7440-36-0 | 3.10E+00 | -1 | 0 | -1 | -1 |
| 2 | Arsenic | 7440-38-2 | 3.40E+00 | -1 | 0 | -1 | -1 |
| 3 | Barium | 7440-39-3 | 1.50E+03 | 0 | 0 | 1 | 1 |
| 4 | Beryllium | 7440-41-7 | 1.60E+01 | 0 | 0 | -1 | -1 |
| 5 | Cadmium | 7440-43-9 | 7.00E+00 | 0 | 0 | -1 | -1 |
| 6 | Chromium (III) | 16065-83-1 | 3.00E+00 | -2 | -2 | -1 | -1 |
| 7 | Chromium (VI) | 18540-29-9 | 3.00E+00 | -1 | -1 | -1 | -1 |
| 8 | Cobalt | 7440-48-4 | 5.41E-01 | -2 | -2 | -1 | -1 |
| 9 | Copper | 7440-50-8 | 3.10E+02 | 0 | 0 | 1 | 1 |
| 10 | Lead | 7439-92-1 | 2.70E+02 | 0 | 0 | 1 | 1 |
| 11 | Manganese | 7439-96-5 | 5.60E+01 | -1 | -1 | -1 | -1 |
| 12 | Mercury | 7439-97-6 | 9.40E-01 | 0 | 0 | -1 | -1 |
| 14 | Nickel | 7440-02-0 | 5.08E+01 | 0 | 0 | -1 | -1 |
| 15 | Selenium | 7782-49-2 | 5.10E+00 | -1 | 0 | -1 | -1 |
| 16 | Silver | 7440-22-4 | 1.58E+00 | -1 | -1 | -1 | -1 |
| 17 | Thallium | 7440-28-0 | 7.80E-02 | -1 | -1 | -1 | -1 |
| 19 | Vanadium | 7440-62-2 | 3.90E+01 | 0 | 0 | -1 | -1 |
| 20 | Zinc | 7440-66-6 | 7.45E+02 | 0 | 0 | 1 | -1 |
| 21 | Cyanide | 57-12-5 | 2.10E+00 | -1 | -1 | -1 | -1 |
| 23 | Dichloromethane | 75-09-2 | 2.31E-02 | -2 | -2 | -1 | -1 |
| 24 | Trichloromethane | 67-66-3 | 5.11E-01 | 0 | 0 | -1 | -1 |
| 25 | Tetrachloromethane | 56-23-5 | 5.77E-02 | -1 | -1 | -1 | -1 |
| 26 | Bromoform | 75-25-2 | 4.72E-01 | -2 | -2 | -1 | -1 |
| 27 | Bromomethane | 74-83-9 | 1.12E-02 | -3 | -3 | -1 | -1 |
| 28 | 1,2-Dichloroethane | 107-06-2 | 3.47E-02 | -2 | -1 | -1 | -1 |
| 31 | Chloroethene | 75-01-4 | 1.78E-02 | -1 | -1 | -1 | -1 |
| 32 | 1,1-Dichloroethene | 75-35-4 | 6.90E-02 | -2 | -2 | -1 | -1 |
| 33 | Trichloroethene | 79-01-6 | 5.40E-02 | -2 | -2 | -1 | -1 |
| 34 | Tetrachloroethene | 127-18-4 | 8.07E-02 | -1 | -1 | -1 | -1 |
| 35 | Benzene | 71-43-2 | 9.77E-02 | -1 | -1 | -1 | -1 |
| 36 | Toluene | 108-88-3 | 3.11E+01 | 0 | 0 | -1 | -1 |
| 37 | Chlorobenzene | 108-90-7 | 3.05E+00 | -1 | -1 | -1 | -1 |
| 38 | 1,2-Dichlorobenzene | 95-50-1 | 2.89E+01 | -1 | 0 | -1 | -1 |
| 39 | 1,3-Dichlorobenzene | 541-73-1 | 7.83E+00 | 0 | 0 | -1 | -1 |
| 40 | 1,4-Dichlorobenzene | 106-46-7 | 3.56E+00 | 0 | 0 | -1 | -1 |
| 41 | 1,2,4Trichlorobenzene | 120-82-1 | 5.80E+00 | 0 | 0 | -1 | -1 |
| 42 | Hexachlorobenzene | 118-74-1 | 7.46E-01 | 0 | 0 | 1 | -1 |
| 43 | Nitrobenzene | 98-95-3 | 3.79E-02 | -2 | -2 | -1 | -1 |
| 44 | Ethylbenzene | 100-41-4 | 3.97E+01 | 0 | 0 | 1 | 1 |
| 45 | Styrene | 100-42-5 | 5.60E+00 | -1 | -1 | -1 | -1 |
| 46 | Xylenes | 1330-20-7 | 5.80E+01 | 0 | 0 | -1 | -1 |
| 47 | Cresol | 1319-77-3 | 3.54E+02 | 1 | 1 | 1 | 1 |
| 48 | Phenol | 108-95-2 | 1.42E+01 | -1 | -1 | -1 | -1 |
| 49 | 2-Chlorophenol | 95-57-8 | 3.53E-01 | -2 | -2 | -1 | -1 |
| 50 | 2,4-Dichlorophenol | 120-83-2 | 2.81E-01 | -2 | -1 | -1 | -1 |
| 51 | 2,4,5-Trichlorophenol | 95-95-4 | 2.58E+01 | -1 | -1 | -1 | -1 |
| 52 | 2,4,6-Trichlorophenol | 88-06-2 | 2.58E-01 | -1 | -1 | -1 | -1 |
| 53 | 2,3,4,6-Tetrahlorophenol | 58-90-2 | 8.60E+00 | -1 | -1 | -1 | -1 |
| 54 | Pentachlorophenol | 87-86-5 | 5.97E-01 | -1 | -1 | -1 | -1 |
| 59 | Chrysene | 218-01-9 | 1.50E+02 | 1 | 1 | 1 | 1 |
| 60 | Dibena(a,h)anthracene | 53-70-3 | 1.50E-01 | 0 | 0 | -1 | -1 |
| 61 | Indeno(1,2,3-c,d)pyrebe | 193-39-5 | 1.50E+00 | 0 | 0 | -1 | -1 |
| 62 | Naphthalene | 91-20-3 | 1.14E-01 | -2 | -1 | -1 | -1 |
| 63 | Acenaphehene | 83-32-9 | 3.21E+01 | 0 | 0 | -1 | -1 |
| 64 | Acenaphthylene | 208-96-8 | 1.45E+01 | 0 | 0 | -1 | -1 |
| 65 | Anthracene | 120-12-7 | 3.54E+02 | 1 | 0 | 1 | 1 |
| 66 | Benzo(g,h,i) perylene | 191-24-2 | 1.70E+02 | 1 | 1 | 1 | 1 |
| 67 | Flouranthene | 206-44-0 | 2.30E+02 | 2 | 1 | 1 | 1 |
| 68 | Flouorene | 86-73-7 | 3.19E+01 | 0 | 0 | -1 | -1 |
| 69 | Phenanthrene | 85-01-8 | 3.49E+01 | 0 | 0 | -1 | -1 |
| 70 | Pyrene | 129-00-0 | 9.03E+01 | 0 | 0 | -1 | -1 |
| 71 | Aldrin | 309-00-2 | 1.80E-01 | 0 | 0 | 1 | -1 |
| 72 | Chlordane | 57-74-9 | 3.50E+00 | 0 | 0 | 1 | 1 |
| 73 | DDT | 50-29-3 | 3.60E+00 | 0 | 0 | 1 | 1 |
| 74 | Dieldrin | 60-57-1 | 4.10E-02 | 0 | -1 | -1 | -1 |
| 75 | Endosulfan | 115-29-7 | 8.13E+00 | 0 | 0 | -1 | -1 |
| 76 | Endrin | 72-20-8 | 1.80E+00 | 0 | 0 | -1 | -1 |
| 77 | Heptachlor | 76-44-8 | 1.20E+00 | 1 | 1 | 1 | 1 |
| 78 | Lindane | 58-89-9 | 6.78E-02 | -1 | -1 | -1 | -1 |
| 79 | Toxaphene | 8001-35-2 | 4.80E+00 | 1 | 1 | 1 | 1 |
| 94 | Total PCDD/PCDF | 1746-01-6 | 5.10E-06 | -1 | -1 | -1 | -1 |
| 95 | Methyl ethyl ketone | 78-93-3 | 1.43E+00 | -3 | -3 | -1 | -1 |
| 96 | Methyl isobutyl ketone | 108-10-1 | 4.24E-01 | -4 | -3 | -1 | -1 |
| 97 | Dibutyl phthalate | 84-74-2 | 1.27E+01 | -4 | -3 | -1 | -1 |
| 98 | DEHP | 117-81-7 | 8.61E+01 | 0 | 0 | 1 | -1 |
| 99 | Hexachlorobutadine | 87-68-3 | 6.83E-02 | -2 | -2 | -1 | -1 |
| 100 | Methyl tert-butyl ether | 1634-04-4 | 4.87E-01 | -2 | -2 | -1 | -1 |
|  |  | Overall | C1 | C2 | C3 | C4 | C5 |
|  |  | Scores | 77 | -0.70 | -0.62 | -43 | -51 |
|  |  |  |  |  | No. >0 | 17 | 13 |
|  |  |  |  |  | No. <0 | 60 | 64 |
